# Verdazyl Radical Organocatalysis Overcomes Chain-Propagation Limitations in Borylation of Arenediazonium Salts

**DOI:** 10.1021/acs.joc.6c00165

**Published:** 2026-05-01

**Authors:** Shrouq Mujahed, Jaysan Janabel, Kundan Shaw, Tynchtyk Amatov

**Affiliations:** 167632 New York University Abu Dhabi, Abu Dhabi 129188, United Arab Emirates

## Abstract

We report an organocatalytic borylation of arenediazonium salts enabled by the bench-stable, nitrogen-centered persistent radical 1,3,5-triphenylverdazyl (TPV). Although this transformation is well precedented, limitations associated with its underlying chain-transfer mechanism have remained largely overlooked. We demonstrate that TPV functions as an efficient redox catalyst that outcompetes rapid radical chain propagation steps while also enabling catalytic chain repair, thereby mitigating inhibition and chain-termination processes that limit purely chain-driven reactions. Comparative studies with common organic electron donors reveal the unique efficacy of electron-rich verdazyl radicals, and contrast with the poor performance of conventional organic electron donors, which are prone to self-inhibition. DFT and Marcus theory calculations indicate lower activation barriers for electron transfer in the TPV-catalyzed pathway, particularly for challenging electron-rich arenediazonium substrates. The reaction proceeds under mild conditions without the need for visible light or mechanoredox mediators, is compatible with a broad range of functional groups, and enables late-stage borylation of aniline-containing drug molecules as well as one-pot telescoped applications of arylpinacolboronate products.

## Graphical abstract

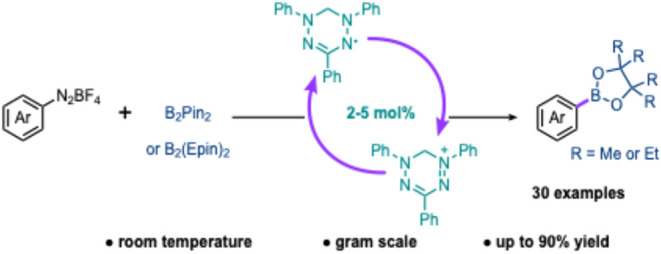

## Introduction

Arylboronic acid derivatives are central to modern cross-coupling technologies.^1–3^ In addition to their low toxicity, they are practical to handle and store due to their stability and tolerance of functional groups. Consequently, boronic acid derivatives are among the privileged building blocks in organic synthesis as they enable programmable synthesis of a variety of complex molecules, and their versatility has driven extensive research into their preparation methods and applications.^4^ Synthetic approaches to boronic acids and their derivatives can be classified into three major categories. The traditional polar approach generally involves nucleophilic attack by organolithium and organomagnesium compounds on electrophilic boron species.^5^ Transition-metal-catalyzed C–X^6^ and C–H^7,8^ borylation represents another common approach. The homolytic C–B bond-forming reactions of transient C-centered radicals with radicalophilic boron reagents are an emerging approach that offers a more sustainable route to organoboronates.^9–11^

In 2010, Wang and co-workers reported a direct conversion of anilines to aryl pinacol boronic esters (arylpinacolboronates) via the reaction of in situ generated arenediazonium salts with bis­(pinacolato)­diboron (B_2_Pin_2_) in the presence of a radical initiator, benzoyl peroxide (BPO).^12^ Later, the Wang team also reported that the borylation scope could be expanded under thermal conditions by conducting reactions at 80 °C without a radical initiator (Figure 1A).^13^ This reaction was proposed to proceed through a classical Sandmeyer-type chemistry via arenediazonium salts, from which aryl radicals are produced by single electron transfer (SET) process and ensuing borylative chain-transfer. Following Wang’s report, a variety of modifications were developed, either via in situ generation of arenediazonium salts from arylamines and aryltriazenes or using presynthesized arenediazonium salts. These modifications can be grouped according to the conditions that favor the generation of aryl radicals (Figure 1B).

**Figure 1:**
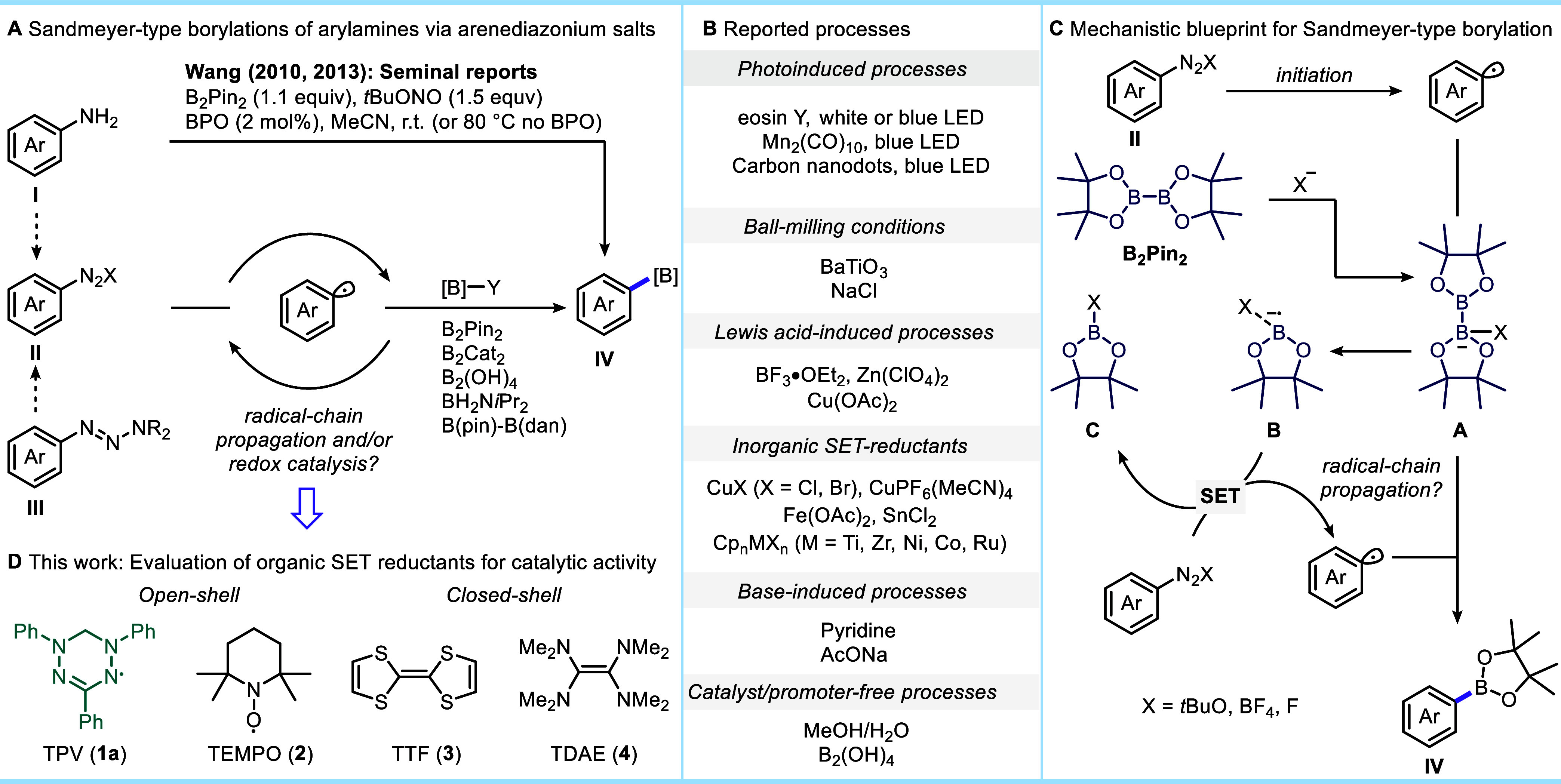
(A) Sandmeyer-type borylation of arenediazonium salts and their derivatives. (B) Previously reported processes. (C) Mechanistic blueprint for borylation. (D) This work: Evaluation of organic SET reductants identifies unique catalytic activity of 1,3,5-triphenylverdazyl (TPV, **1a**).

Yan and co-workers reported the first photoinduced borylation of arenediazonium salts using the organic dye eosin Y as a photoredox catalyst under white- and blue-LED irradiation.^14^ Following this, borylation of arenediazonium salts in water under visible-light irradiation in the presence of carbon nanodots,^15^ as well as light- and manganese-initiated borylation of arenediazonium salts, were disclosed.^16,17^ Borylation protocols under ball milling conditions were also reported using the piezoelectric material BaTiO_3_,^18^ and recently, ball milling in the presence of simple NaCl.^19^ While simple bases were also reported to initiate the borylation of arenediazonium salts,^20^ borylation of aryltriazenes, on the contrary, requires Lewis acid mediators or catalysts that promote the generation of arenediazonium salts from the latter.^21,22^ Hence, in some instances, both ionic and radical mechanisms were postulated for the borylation of arenediazonium salts.^23–25^ Paralleling classical Sandmeyer-type chemistry, inorganic SET reductants, such as Cu­(I) salts,^22^ metallocene complexes,^26^ and tetrabutyl ammonium iodide,^27^ have also been shown to promote the borylation of arenediazonium salts at catalytic loadings. Catalyst- or promoter-free borylation of arenediazonium salts were also disclosed when tetrahydroxydiboron was used,^28^ under aqueous acetone conditions without any added radical initiators or SET reagents.^29^ Nonarenediazonium approaches to borylation of anilines via aryl radicals have also been described.^30–36^

Given that borylation of arenediazonium salts proceeds under a variety of conditions favoring aryl radical generation,^12,37^ it is clear that radical-chain propagation can be the dominant mechanism in Wang-Sandmeyer borylation. On the other hand, quantum yield measurements by Majek and von Wangelin showed that photoinduced borylation using eosin Y exhibited a quantum yield of 0.60,^38^ suggesting only moderate efficiency of the radical-chain propagation mechanism and opportunities for catalysis. In the commonly accepted radical-chain propagation mechanism (Figure 1C), the reaction is initiated by the generation of aryl radicals from arenediazonium salts **II** which attacks anionic species **A** formed by the Lewis acid–base interaction between B_2_Pin_2_ and anions **X**. Product formation is accompanied by the generation of ligated anion radical **B**, which transfers a single electron to arenediazonium salt **II**, which continues the chain process. The nature of X in the boron-containing byproduct **C** can influence the equilibrium concentration of **A**, as species **C** can compete with B_2_Pin_2_ toward X and potentially determine the efficiency of chain-transfer. The described mechanistic blueprint for the borylation of arenediazonium salts with B_2_Pin_2_ offers opportunities for organocatalytic intervention, provided that organic redox-active molecules can compete with the radical-chain-propagation pathway. Ideally, such an organic redox catalyst will also be capable of catalytic reinitiation to repair terminated chains to sustain high yields.

Recently, we showed that Kuhn-verdazyls,^39^ electron-rich nitrogen-centered persistent radicals with stronger reducing power than nitroxides and even ferrocene, function as effective and tunable ground-state redox catalysts in C–H arylation and C–H trifluoromethylation of (hetero)­arenes.^40^ Herein, we apply their ability to emulate single electron shuttling, analogous to transition-metal and photoredox catalysts, to ground-state organocatalytic Wang-Sandmeyer borylation of arenediazonium salts. We demonstrate that 1,3,5-triphenylverdazyl (TPV, **1a**) consistently affords arylpinacolboronates in high yields under mild conditions, without heating or photoirradiation. Comparative evaluation against established organic electron donors reveals that TPV uniquely delivers high and substrate-independent yields for both electron-rich and electron-deficient arenediazonium salts, underscoring its distinct catalytic activity (Figure 1D).

## Results and Discussion

Initially, we evaluated the background reactivity in the presence of base, as well as the efficiency of the base-induced chain reaction,^41^ by incubating 4-methoxybenzenediazonium tetrafluoroborate (**5a**) with two equivalents of B_2_Pin_2_ and NaHCO_3_ in acetonitrile, as it is well-known that the presence of certain weak bases initiates SET-induced reactions of arenediazonium salts. Under these conditions, less than 10% arylpinacolboronate **6a** was formed by NMR analysis (Table 1, entry 1) indicating either very slow initiation or inefficient radical-chain propagation according to the mechanistic blueprint in Figure 1. Keeping this stoichiometry as the standard condition, we next tested the effect of organic SET reductants **1a-4** in 5 mol % catalytic loading. TTF (**3**), which has a moderate reducing ability, has been shown by Murphy and co-workers to be an efficient SET reductant to arenediazonium salts.^42–47^ Indeed, when 5 mol % TTF was added, the yield of **6a** increased to 68% (Table 1, entry 2). TDAE (**4**), a prototypical organic super electron donor (SED), also provided **6a** in 68% yield under the same conditions (Table 1, entry 3). On the other hand, the addition of 5 mol % TEMPO (**2**), which is also known to induce SET reduction of arenediazonium salts,^48,49^ surprisingly provided 50% yield (Table 1, entry 4). Under identical conditions, when 5 mol % of TPV (**1a**) was used, the arylpinacolboronate **6a** was formed in a substantially higher isolated yield of 88% (92% NMR yield; Table 1, entry 5). The addition of base was shown to be critical in these reactions. TPV, as well as TEMPO, are known to rapidly disproportionate in the presence of acids.^50,51^ At the same time, TTF and TDAE can be protonated,^52^ and as the reaction presumably generates acidic boron-derived byproduct species, such as **C** (see Figure 1), or HBF_4_ from incipient moisture, they can also induce protodeborylation of the product. For example, omitting NaHCO_3_ in the presence of TPV and two equivalents of B_2_Pin_2_ provided **6a** in only 14% yield (Table 1, entry 6). Adding one equivalent of NaHCO_3_ restored the reaction and increased the yield to 80% (Table 1, entry 7). We also performed the reaction in the presence of one equivalent of B_2_Pin_2_ and 5 mol % TPV. However, the yield decreased to 56% in this case (Table 1, entry 8). Collectively, these results demonstrate the unique effect of l TPV on the efficiency of Wang-Sandmeyer borylation. Monitoring reaction yield by all tested OEDs over 24 h indicates that comparably rapid initiation takes place in the presence of TPV, TTF, and TDAE. At the same time, the weakest SET reductant, TEMPO, exhibits slow initiation. Surprisingly, however, the TPV-mediated reaction is significantly accelerated after a comparable initial period with TTF and TDAE, yielding **6a** at 90%, while the yield of **6a** plateaued at 68% with TTF and TDAE. The yield with TEMPO was the lowest, and stalled at 50%.

**Table 1:**
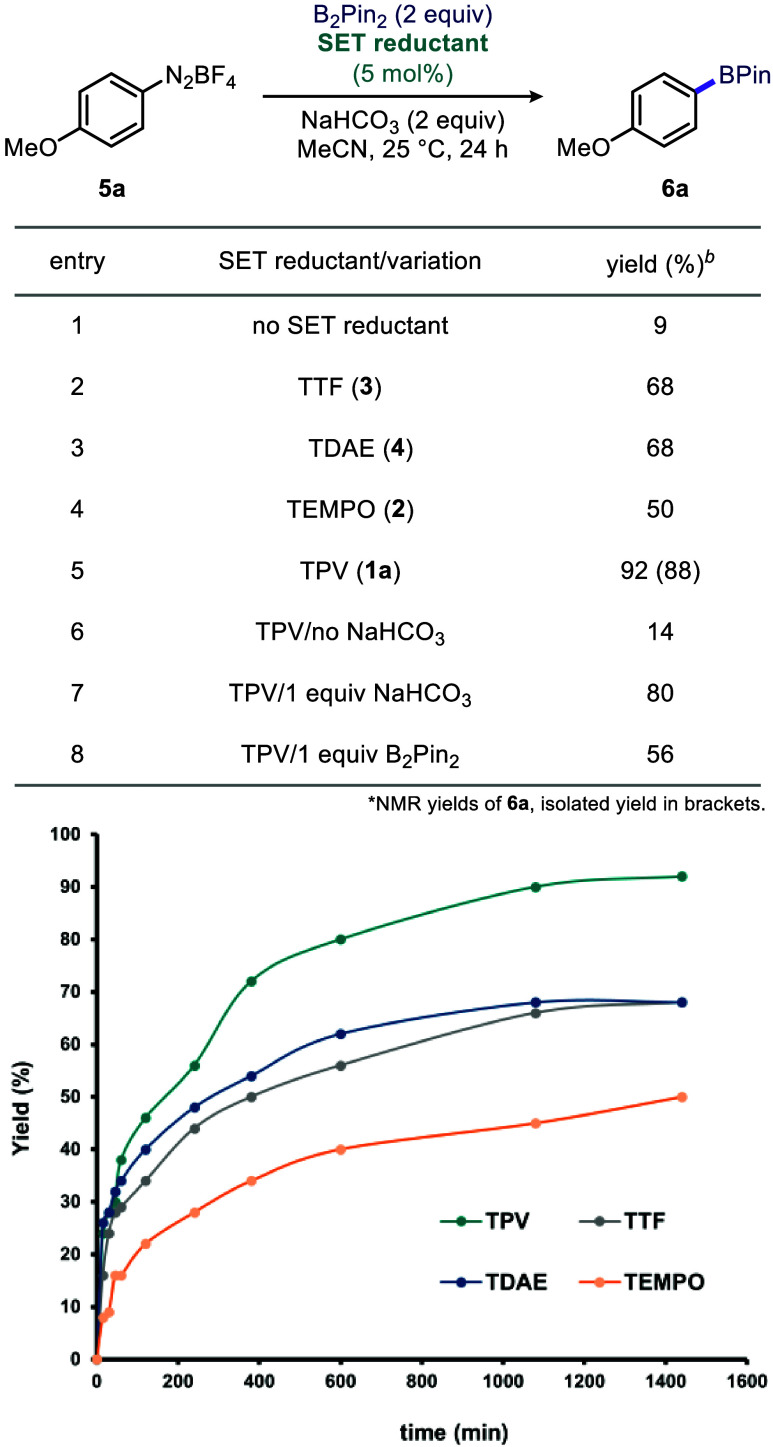
Comparison of Organic SET Reductants **1-4** ^a^

a Reactions were performed on a 0.2 mmol scale in acetonitrile (0.2 M) under N_2_ atmosphere for 24 h.

Under optimized conditions with TPV at 5 mol % catalytic loading, we next explored the substrate scope of our organocatalytic borylation of arenediazonium salts (Scheme 1). A wide variety of arenediazonium tetrafluoroborate salts afforded the arylpinacolboronate products in good yields, as indicated by ^1^H NMR analysis of the crude reaction mixtures and by the isolated yields. In a few cases, a significant loss of yield occurred due to their known instability on silica gel during column chromatography purification.^53^ Arylpinacolboronate products can also be isolated as aryltrifluoroborates without using column chromatography by treating the crude reaction mixture with potassium bifluoride (Supporting Information, p. S21). The borylation worked well with both electron-rich and electron-deficient arenediazonium salts, except for simple phenyl-, 3,5-dimethylbenzene-, and 2,4,6-trimethylbenzenediazonium tetrafluoroborate salts (**5l**, **5j**, and **5k**, respectively). Interestingly, Majek and Sebesta also observed a low yield of the desired borylation of the phenyldiazonium tetrafluoroborate salt in their mechanochemical protocol and reported that a 1,3-diboronated side-product was observed in up to 18% yield, which is in accordance with our results (Supporting Information, p. S6). In addition to fluoro-, chloro-, and bromoarendiazonium salts, the iodoarenediazonium salt reacted equally well (**6m**–**6r**). Other electron-withdrawing groups, such as carboxylates (**6s** and **6t**), trifluoromethyl (**6u**), nitro (**6v**) as well as both *o*- and *p*-cyano (**6w** and **6x**) and ketones (**6y** and **6z**) are well tolerated . The latter example demonstrates that diborylation can also be performed with practical yields. Most importantly, our method is well-compatible with drug-derived arenediazonium salts. Arenediazonium salts derived from drug molecules such as aminoglutethimide and benzocaine were borylated in moderate and good yields (**6f** and **6t**, respectively). Since TEMPO was also shown to promote borylation in reasonable yields, we compared its performance in the borylation of several additional arenediazonium salts.

**Scheme 1:**
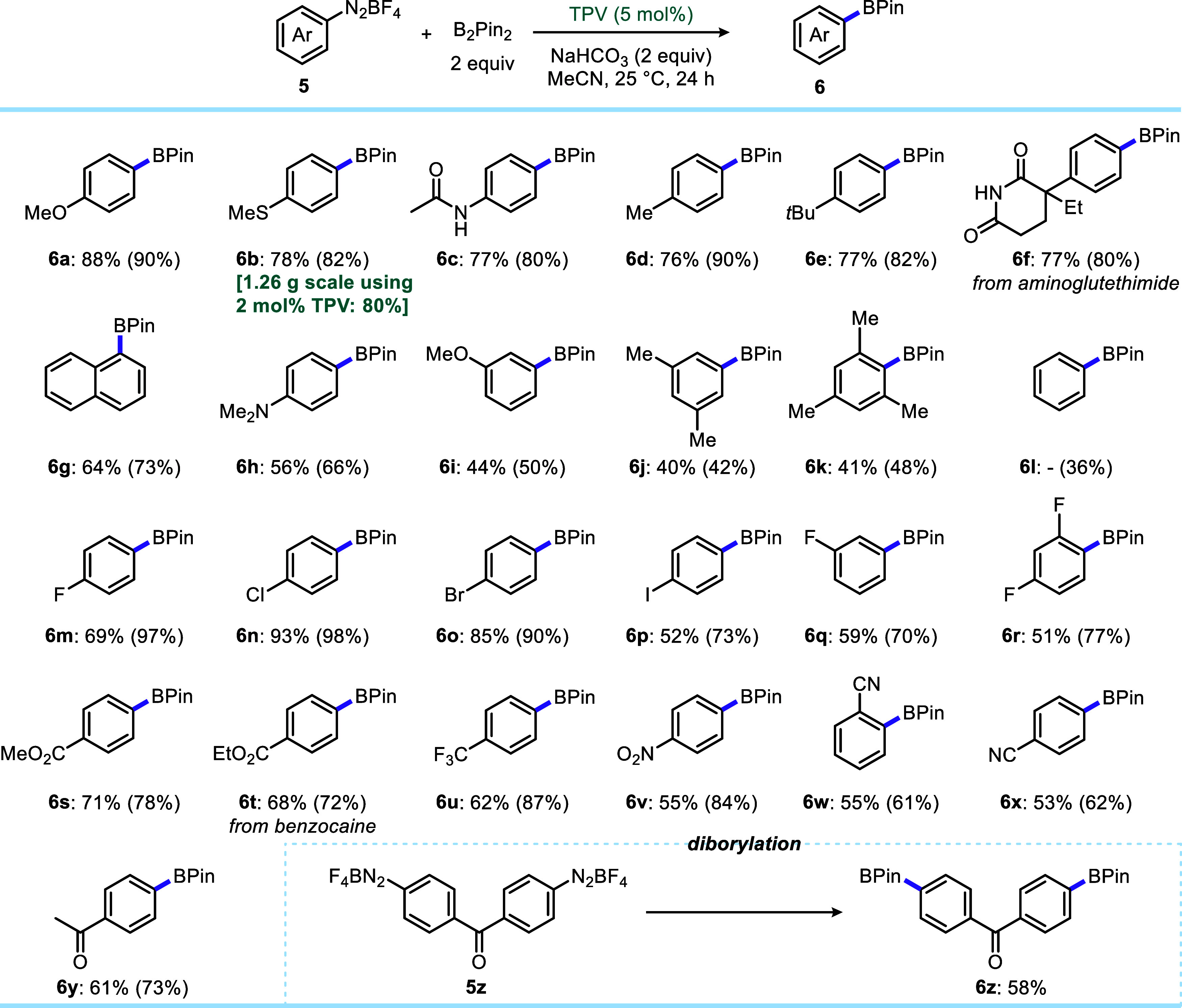
Scope of Verdazyl-Catalyzed Borylation of Arenediazonium Salts^a^

a Reactions were performed on a 0.4 mmol scale in acetonitrile (0.2 M) under N_2_ atmosphere for 24 h. NMR yields determined by crude ^1^H NMR analysis are reported in brackets.

In most cases, the ^1^H NMR yields with TEMPO were 20–40% lower than those with TPV. The most electron-rich *p*-dimethylaminoarenediazonium tetrafluoroborate **5h** gave no observable product formation with TEMPO, while TPV provided 66% of the corresponding arenediazonium salt (Supporting Information, Table S2). Finally, the TPV loading can be reduced to 2 mol % without compromising the yield, as demonstrated by a gram-scale synthesis of **6b**. Given the safety concerns with handling arenediazonium salts,^54^ arylamino-containing drug molecules would be best modified without isolating them (Scheme 2). Especially, this would be attractive for the late-stage modification of drug molecules and drug candidates. We have performed in situ diazotization/verdazyl-catalyzed borylation without isolating the arenediazonium procaine hydrochloride, a widely used local anesthetic, which furnished 56% yield of procaine-pinacol boronate **8** (Scheme 2A). Significantly, this protocol does not need NaHCO_3_ as the *tert*-butoxide counterion serves as the base to sequester Brønsted and Lewis acidic byproducts. As the TPV-catalyzed borylation does not involve any transition-metal catalysts or promoters, the arylpinacolboron esters can be telescoped in the follow-up Suzuki-Miyaura coupling in the same pot. We found that yields in this one-pot borylation/Suzuki coupling could be increased by decomposing excess B_2_Pin_2_ with *N*-methylmorpholine N-oxide, as reported by Lakshman.^55^ Using this approach, nonsteroidal anti-inflammatory drug felbinac (**10**) was obtained in 63% overall yield from 4-(carboxymethyl)­benzenediazonium tetrafluoroborate salt **9** (Scheme 2B). Additionally, an efficient one-pot borylation/Petasis reaction was performed in a transition-metal-free setting to provide amino acid derivative **11** in 55% overall yield over two steps (Scheme 2C).^56^ Such telescoped transformations are valuable because transition-metal residues from metal-mediate borylation step can interfere with downstream reactions or are undesirable altogether in medicinal chemistry. The compatibility of our method with other diboron reagents was tested using the more bulky bis­(1,1,2,2-tetraethylethylene glycolato)­diboron (B_2_Epin_2_) and more Lewis acidic bis­(catecholato)­diboron (B_2_Cat_2_). Borylation using B_2_Epin_2_ proceeded with even higher yields than with B_2_Pin_2_ (Scheme 2D). For example, the borylation of problematic phenyldiazonium tetrafluoroborate (**5l**) was improved (from 36% NMR yield using B_2_Pin_2_) to a more practical 60% isolated yield of PhBEpin **12**, while the borylation of 4-methylbenzenediazonium tetrafluoroborate **5d** also proceeded with significantly improved isolated yields (75% **6d** using B_2_Pin_2_ vs 90% **13** using B_2_Epin_2_). These results agree with Akai’s and Ikawa’s reports on the improved stability of ArBEpin esters compared to on silica gel.^53^ Unfortunately, the borylation using B_2_Cat_2_ failed even when attempted isolation of organoboron products via conversion to aryltrifluoroborate salts.

**Scheme 2:**
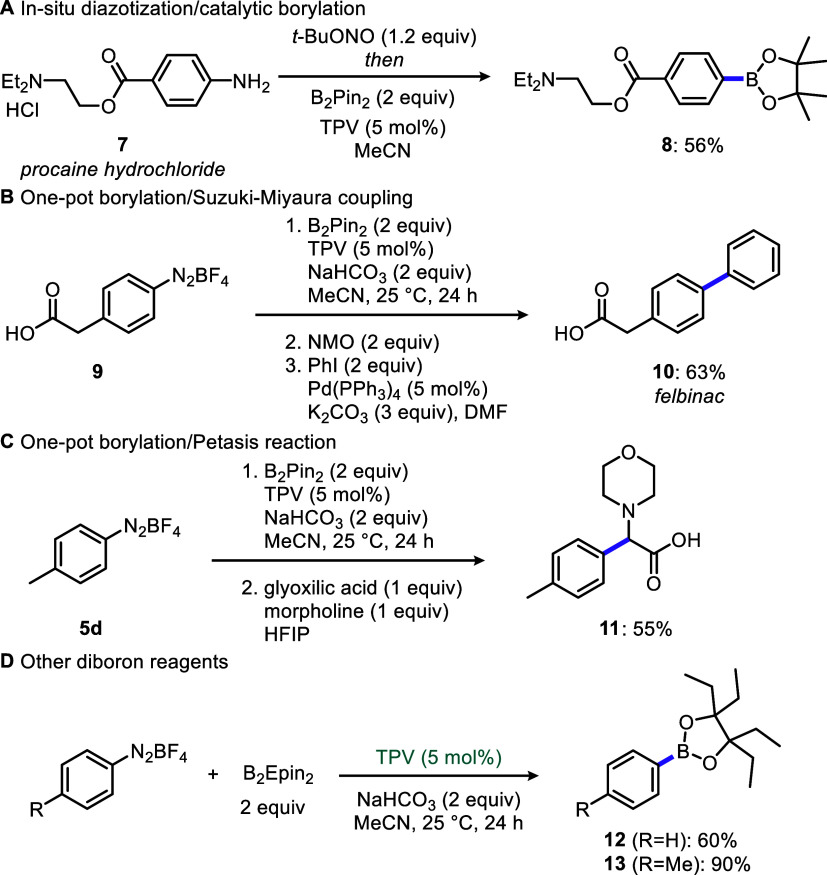
Applications in Telescoped Transformations and with a Bulky Diboron Reagent B_2_Epin_2_

### Mechanistic Studies

Finally, we carried out experimental and computational studies to understand TPV’s unique performance in Wang-Sandmeyer borylation compared to the other three tested organic electron donors, TEMPO, TTF, and TDAE. The significance of radical chain-propagation in the transformations of arenediazonium salts is widely acknowledged.^38^ However, the efficiency of chain-propagation might be highly dependent on the reaction type and conditions. Distinguishing between a chain-transfer mechanism and true redox catalysis, or estimating the relative contributions of each mechanism in SET-induced radical reactions, is often challenging.^12,57^ In a system governed by relatively efficient chain propagation, the overall reaction efficiency should be largely independent of the SET reductant used to initiate radical formation. In such cases, the chain process sustains itself after SET initiation, making the nature of the SET reductant irrelevant to the reaction outcome, unless a self-inhibition mechanism is present. The relevance of each mechanism can, however, be probed by comparing the performance of SET reductants of differing redox potentials. In other words, in the redox catalysis scenario, the choice of SET initiator matters in the reaction outcome, while in the chain transfer scenario it does not.

Expanding on these points, we conducted competition experiments with two different arenediazonium salts using various SET reductants (Figure 2A). Subjecting a 1:1 mixture of electron-rich 4-methylbenzenediazonium tetrafluoroborate (**5d**, *E*_p_^red^ = −0.565 V versus Fc^+^/Fc)^58^ and electron-deficient 4-chlorobenzenediazonium tetrafluoroborate (**5n**, *E*_p_^red^ = +0.01 V versus Fc^+^/Fc)^59^ to the borylation conditions with one equivalent of B_2_Pin_2_ using 5 mol % SET reductants provided mixtures of arylpinacolboronates **6d** and **6n**, favoring the formation of **6n** over **6d**, consistent with the pronouncedly easy reduction of **6n**. When TPV (**1a**) was used, arylpinacolboronates **6d** and **6n** were formed in a 1:8 ratio in an overall 36% yield. The more strongly reducing triarylverdyazyl, bearing two 4-methoxyphenyl and one 4-methylphenyl substituents,^60^ provided a 1:6.8 ratio of **6d** and **6n**, in a slightly higher 39% total yield, consistent with its more negative oxidation potential. Surprisingly, in the presence of 5 mol % TEMPO, this particular combination of arenediazonium substrates provided slightly higher total yield of products compared to TTF and TDAE (31, 26 and 20%, respectively). The ratios of **6d** and **6n** decreased in going from TEMPO to TTF and TDAE (1:6.8, 1:5.5, and 1.4, respectively). Because the results of competition experiments in the presence of TEMPO, TTF, and TDAE did not yield a straightforward correlation, we were also eager to compare cobaltocene’s performance. This organometallic SET reductant is even more strongly reducing (*E*_1/2_^ox^ = −1.33 V versus Fc^+^/Fc) than TDAE.^61,62^

**Figure 2:**
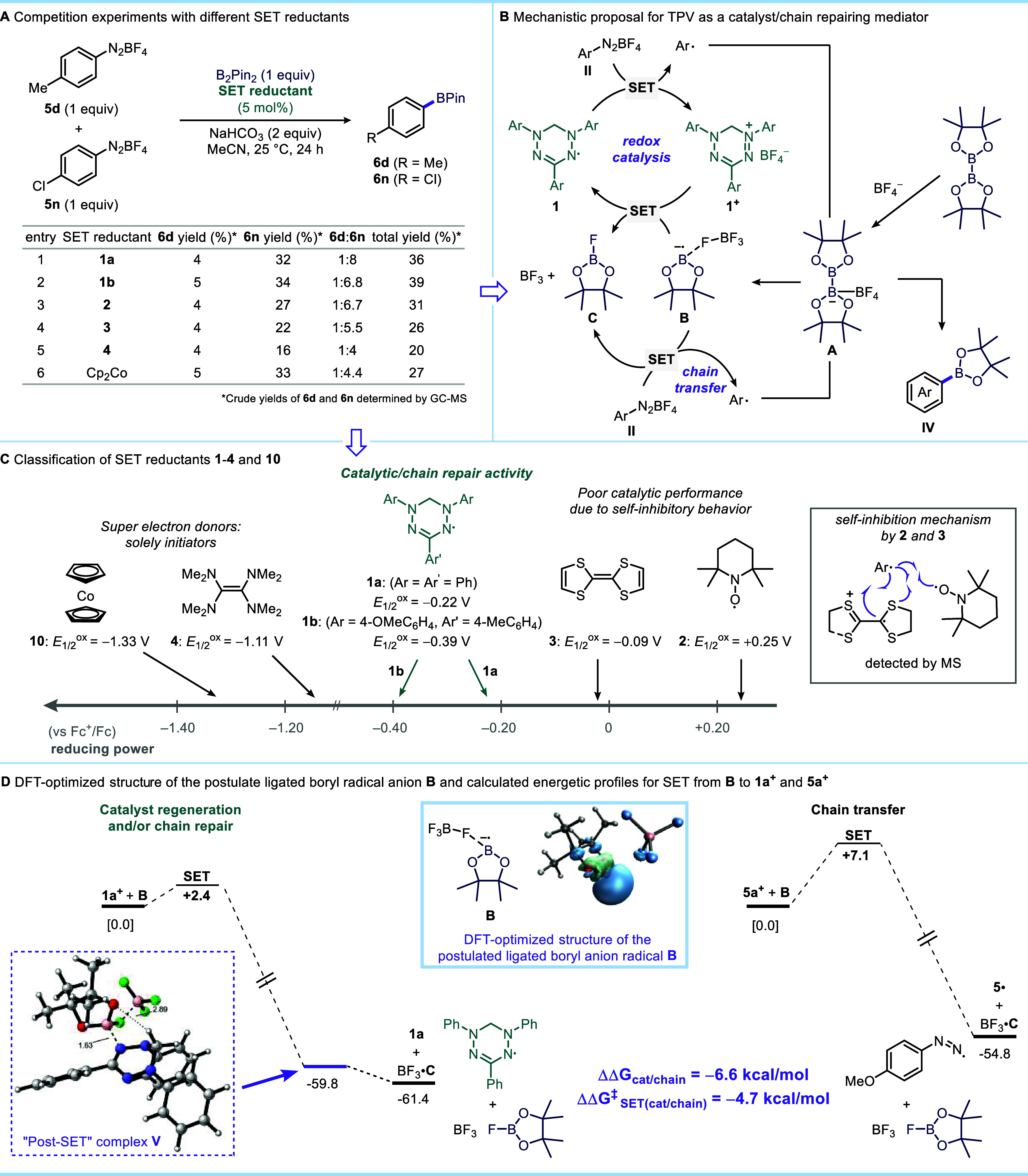
Mechanistic studies. (A) Competitive borylation experiments with different SET reductants. (B) Proposed catalytic cycle for verdazyl-catalyzed borylation. (C) Classification of SET reductants according to their performance. (D) Theoretical calculations. Calculations were performed at uM06–2*X*/6–311++g­(d,p)­SMD­(MeCN)//uM06–2*X*/6–311+g­(d,p) level of theory.

Interestingly, cobaltocene provided **6d** and **6n** in a 1:4.4 ratio, which is close to the product ratio obtained with TDAE. Based on the product distributions from competition experiments and the results in Table 1, we suggest that organic SET reductants **1–4** can be classified into three categories (Figure 2C). While weakly reducing TEMPO (*E*_1/2_^ox^ = +0.25 V versus Fc^+^/Fc) and TTF (*E*_1/2_^ox^ = −0.09 V versus Fc^+^/Fc) can, in principle, act as redox catalysts, both exhibit self-inhibition properties (Figure 2C). Because TEMPO reduces the arenediazonium salts only slowly, it will coexist with transient aryl radicals, eventually irreversibly trapping them.^63,64^ The cation radical of TTF is also known to trap C-centered radicals via coupling at the S atom^42–47^ Super electron donor TDAE (*E*_1/2_^ox^ = −1.11 V versus Fc^+^/Fc)^65^ and its analogs also generate cation radicals upon SET reduction of substrates, which are potentially capable of trapping C-centered radical species.^66^ However, unlike the cation radical derived from TTF, the SET reduction of TDAE^+•^ cation radical to neutral TDAE by boryl anion radical species **B** might not be thermodynamically feasible and rather it can undergo further SET to arenediazonium salt, generating a second equivalent of aryl radical and dicationic TDAE^2+^.^67^ The mass spectrometric analysis of crude reaction mixtures from the borylation of **5a** with organic electron donors **1–4** indeed allowed the detection of aryl radical trapping products of TTF and TEMPO, while no such self-inhibition products due to trapping of aryl radical with TDAE were detected. As such, the organic super electron donor TDAE and the strongest electron donor cobaltocene act solely as initiators. No N-aryl coupling adducts with TPV were detected by mass spectrometric analysis of the crude reaction mixture. Instead, in addition to intact TPV, species corresponding to arylation of one of the three phenyl rings of TPV were observed (see Supporting Information, Figure S3). However, these are likely inconsequential for catalytic turnover (vide infra). Verdazyls **1a** and **1b** have optimal redox potentials that are in the redox potential window of typical arenediazonium salts. As a result, they are not only capable of rapidly reducing the arenediazonium salts, but also their corresponding oxidized verdazylium derivatives can compete with the arenediazonium chain acceptors. Based on these experimental results, we propose that Kuhn verdazyls **1a** and **1b** perform either as competent catalysts or catalytic chain repair agents (Figure 2B). Due to fast SET to arenediazonium salts,^68^ verdazyls avoid the self-inhibitory mechanism as the electron transfer and oxidation of verdazyl **1** to verdazylium cation **1** ^**+**^ occur concurrently, while the reverse process, the reduction of **1** ^**+**^ regenerating **1** occurs at similar potentials to the reduction potentials of typical arenediazonium salts. The catalytic chain repair ability of the TPV/TPV^+^ redox system was further probed experimentally using 5 mol % independently synthesized verdazylium tetrafluoroborate **1a** ^**+**^ **BF**_**4**_^**–**^ in place of TPV.^69^ Under these conditions, arylpinacolboronate **6a** was formed in a comparable yield (94% NMR yield) despite exhibiting an expected induction period. (Supporting Information, Scheme S1). These results support the in situ generation of TPV via SET reduction of TPV^+^ by reducing boryl species under the reaction conditions. The detection of the boryl radical anion **B** under catalytic conditions is particularly difficult, as this species is expected to undergo near-instantaneous electron transfer to either the arenediazonium salt or the verdazylium cation. Therefore, we were interested if alternatively generated ligated boryl radical species could induce the reduction of **1a** ^**+**^ **BF**_**4**_^**–**^ to neutral verdazyl **1a**. Recently, the pyridine boryl radicals, generated by ligation-mediated homolysis of diboron compounds emerged as unique catalytic SET reductants.^70,71^ Indeed, when purple **1a** ^**+**^ **BF**_**4**_^**–**^ was treated with excess B_2_Pin_2_ in the presence of 4-phenylpyridine, formation of the green verdazyl radical **1a** was observed. This experiment further supports the feasibility of a closed redox catalytic cycle, or chain repair via regeneration of the verdazyl radical from verdazylium via SET from boryl radicals in the absence of terminated aryl radicals (Supporting Information, Scheme S2 and Figure S4).

We also performed DFT calculations to compare the key steps in the competing mechanisms, namely the SET steps involved in closing the redox catalysis cycle and the chain transfer. As both steps involve electron transfer from the postulated ligated boryl anion radical species **B**, we optimized its structure which suggests significant spin density on the boron atom (Figure 2D, light blue box and Supporting Information, Figure S7). DFT and Marcus theory calculations suggest that SET from the boryl anion radical **B** to verdazylium cation **1a** ^**+**^ is kinetically more favorable, with the activation free energy for single electron transfer to **1a** ^**+**^ 4.7 kcal/mol lower compared to chain transfer to **5a** ^**+**^. Moreover, this process is also more exergonic by 6.6 kcal/mol compared to SET reduction of an electron-rich 4-methoxybenzenediazonium cation **5a** ^**+**^ (Figure 2C). Interestingly, our calculations identify a “post-SET” complex **V** with strongly stabilizing interactions between triphenylverdazyl and incipiently developing neutral boron associates (Figure 2D, purple dashed box). Complex **V** features noncovalent interactions such as C–H···O hydrogen bond interaction (2.89 Å)^72^ between the oxygen atom of the pinacolboron ring and the CH_2_ group in **1a**, as well as a short B–N contact (1.63 Å) between the boron and nitrogen atoms of anion radical **B** and the verdazyl **1a**. We suggest that, in the incipient charge-transfer state along the reaction coordinate,^73^ the Coulombic attraction as well as the above-mentioned noncovalent interactions between anion radical **B** and the verdazylium cation **1a** ^**+**^, further contribute to substantially lowering the electron transfer to close the catalytic cycle. The singly occupied molecular orbital (SOMO) of “post-SET” complex **V** is almost entirely delocalized on triphenylverdazyl, and Mulliken spin density calculations indicate that the majority of spin density resides on the verdazyl moeity (see Supporting Information, Figures S9,S10). Interestingly, no such “post-SET” complex formation following the electron transfer between 4-methoxybenzenediazonium cation **5a** ^**+**^ and anion radical **B** could be located in the radical chain propagation mechanism.

## Conclusions

In summary, we report an organocatalytic borylation of arenediazonium salts using the nitrogen-centered persistent radical 1,3,5-triphenylverdazyl (TPV), first introduced by Kuhn in 1963. The method produces arylpinacolboronates in high yields at room temperature under mild conditions without light, mechanochemical, or electrochemical stimuli under conventional settings and can be scaled up with low catalyst loadings without compromising reaction yields. Our experimental and computational results show that TPV uniquely promotes the Wang-Sandmeyer borylation by overcoming the self-inhibition mechanism or via chain repair, thereby decreasing the reaction’s reliance on the efficiency of chain propagation. Although reactions with efficient innate chain-transfer steps are inherently challenging to catalyze because the individual steps are fast, our work delineates a strategy to overcome this challenge. We demonstrate that even highly difficult-to-control innate chain reactions can be catalyzed when the catalyst engages in more favorable noncovalent interactions with chain-transfer species. Moreover, the mild reaction conditions are compatible with late-stage modification of bioactive arylamine molecules and enable one-pot telescoping.

## Methods

### General Procedure for TPV-Catalyzed Borylation Reactions

Under an Ar atmosphere inside a glovebox (or in a Schlenk flask under N_2_ outside glovebox), an oven-dried 8 mL vial was charged with a magnetic stir bar, sodium bicarbonate (0.4 mmol, 33.6 mg, 2.0 equiv), bis­(pinacolato)­diboron (0.4 mmol, 101.6 mg, 2.0 equiv), dry and degassed MeCN (0.2 M, 1 mL), and the corresponding arenediazonium salt (0.2 mmol, 1.0 equiv), followed by the addition of TPV (0.01 mmol, 3.1 mg, 5 mol %). The vial was sealed with a septum cap and parafilm. The reaction mixture was taken outside the glovebox and stirred at 25 °C for 24 h. The solvent was removed using a rotary evaporator, and the residue was purified by automated flash chromatography to afford the pure product.

## Supplementary Material



## Data Availability

The data underlying this study are available in the published article and its supporting information.

## Notes

Copies of ^1^H, ^13^C, ^13^C­{^1^H}, ^19^F, ^11^B NMR and HRMS (ESI) spectra; and computational details (PDF)
