# Supplementary material for: Verdazyl Radical Organocatalysis Overcomes Chain-Propagation Limitations in Borylation of Arenediazonium Salts
Source: J Org Chem. 2026 May 1;91(19):6623–32. doi: 10.1021/acs.joc.6c00165 (PMC13185099; doi:10.1021/acs.joc.6c00165)
Supplement: Supplementary file 1 [file jo6c00165_si_001.pdf]

Supporting Information

Verdazyl Radical Organocatalysis Overcomes Chain-Propagation Limitations in Borylation of Arenediazonium Salts

Shrouq Mujahed‡, Jaysan Janabel‡, Kundan Shaw and Tynchtyk Amatov*

[1]New York University Abu Dhabi, Abu Dhabi 129188, United Arab Emirates

*Email: ta2511@nyu.edu

Contents

General information S2

Safety notes S3

Arenediazonium salts used in this study and their preparation. S3

General reaction procedure for borylation reactions* S5

Comparison of TPV with different SET reductants on performance against a selected panel of substrates S5

Data for Table 1 .... S5

Comparison of TPV with different SET reductants in borylation of phenyldiazonium tetrafluoroborate S6

GC–MS Analysis of competition experiments S7

Detection of adducts of aryl radical with SET reductants using mass spectrometry S9

Probing the chain-repair catalysis scenario with verdazylium tetrafluoroborate ($TPV^+BF_4^-$) S10

Reduction of verdazylium tetrafluoroborate ($TPV^+BF_4^-$) by $^{4\text{-}Ph}$Pyridine/B_2Pin_2 adduct S11

Data for borylation reactions S12

Procedure for one-pot in-situ diazotization/catalytic borylation S20

Procedure for one-pot two-step synthesis of Ar-BF_3K salt S21

Procedure for one-pot borylation/Suzuki-Miyaura coupling S21

Procedure for one-pot borylation/Petasis reaction S22

Gram-scale synthesis of 6b using 2 mol% TPV S22

Borylation with B_2Epin_2 .... S23

Copies of NMR Spectra S25

DFT Calculations S76

Using Marcus Theory to estimate the activation barriers for SET S76

Molecular orbitals and spin density plots S78

Cartesian coordinates of the optimized structures S79

References S90

General information

Unless otherwise stated, all reactions were magnetically stirred and conducted in oven-dried (125 °C) or flame-dried glassware in anhydrous solvents under Ar or N_2, applying standard Schlenk techniques. Solvents, liquid reagents, and solutions of solid or liquid reagents were added via syringes, stainless steel or polyethylene cannulas through rubber septa or under a weak N_2 counter-flow. Solid reagents were added through a weak Ar counter-flow. Cooling baths were prepared in Dewar vessels, filled with ice/water (0 °C), chilled acetone/isopropanol (> –78 °C), or dry ice/acetone (–78 °C). Heated oil baths were used for reactions requiring elevated temperatures. Solvents were removed under reduced pressure at 40 °C using a rotary evaporator, and unless otherwise stated, the remaining compound was dried under high vacuum at ambient temperature. All given yields are isolated yields of chromatographically and NMR spectroscopically pure materials, unless otherwise stated.

Chemicals and solvents

Chemicals were purchased from commercial suppliers (including abcr, Ambeed, Acros, Alfa Aesar, Fluorochem, Merck, Sigma, and TCI) and used without further purification unless otherwise stated. DMSO, acetonitrile, and benzene were purchased from commercial suppliers, dried over 3 Å molecular sieves, and degassed before use. Other solvents were dried using the Innovative Technology solvent purification system PS-MD-7. Verdazyl **1b** was synthesized according to Gilroy, who reported its oxidation potential ($E_{1/2}^{ox}$ = -0.39 V vs Fc/Fc).[1]

Thin layer chromatography

Thin layer chromatography (TLC) analysis was performed by using silica gel precoated plates (0.25 mm) 60 (F-254), visualized by irradiation with UV lamp (λ = 254 or 366 nm), basic $KMnO_4$, and/or phosphomolybdic acid (PMA). PMA stain: PMA (20 g) in EtOH (200 mL), $KMnO_4$ stain: aq NaOH (10 wt%, 1.25 mL), $KMnO_4$ (1.5 g), K_2CO_3 (10 g) in H_2O (200 mL).

Automated column chromatography

All preparative chromatographic purifications were performed using a Biotage Select instrument with UV Detector, using pre-packed silica gel columns (Biotage Sfar Silica HC D – High Capacity Duo Samplet) purchased from Biotage. All fractions containing a desired substance were combined and concentrated in vacuo, then redissolved in an appropriate solvent and filtered through a cotton plug to remove silica residues.

Nomenclature

The nomenclature follows the suggestions proposed by the computer program ChemDraw Professional 15.0 of PerkinElmer®.

Nuclear magnetic resonance spectroscopy

^{1}H, ^{13}C, ^{19}F, ^{31}P Nuclear magnetic resonance (NMR) spectra were recorded on a Bruker Advance-III 500 MHz FT-NMR spectrometer in a suitable deuterated solvent. The solvent employed and respective measuring frequency are indicated for each experiment. Chemical shifts are reported with Me_4Si serving as a universal reference of all nuclides and with two or one digit after the comma. The resonance multiplicity is described as s (singlet), d (doublet), t (triplet), q (quadruplet), p (pentet), hept (heptet), m (multiplet), and br. (broad). All spectra were recorded at 298 K unless otherwise noted, processed with the program Bruker TopSpin 4.4.0 or MestReNova 15.0.1, and coupling constants are reported as observed. The residual deuterated solvent signal relative to tetramethylsilane (TMS) was used as the internal reference in ^{1}H NMR spectra ($CDCl_3$ δ 7.26, acetone-d_6 δ 2.05, CD_3OD δ 3.31), and are reported as follows: chemical shift δ in ppm (multiplicity, coupling constant *J* in Hz, number of protons). ^{13}C NMR spectra reported in ppm from tetramethylsilane (TMS) with the solvent resonance as the internal standard ($CDCl_3$ δ 77.16, acetone-d_6 δ 206.26, CD_3OD δ 49.00). All spectra are broadband decoupled unless otherwise noted.

Mass spectrometry

High-resolution mass spectrometry (ESI-HRMS) or (APCI-HRMS) was performed on Agilent LCMS QToF 6538 using methanol as solvent. The ionization method and detection mode employed are indicated for each experiment, and all masses are reported in atomic units per elementary charge (m/z), with intensities normalized to the most intense peak.

Gas chromatography–mass spectrometry (GC–MS)

GC–MS analyses were carried out on an Agilent 7000D GC–MS system equipped with a Gerstel autosampler. Samples were analyzed under electron ionization (EI) conditions. Compounds were identified by their

retention times and mass spectra, and relative quantification was performed based on integrated peak areas. All mass spectra are reported as mass-to-charge ratios (m/z) with intensities normalized to the base peak.

Safety notes

Arenediazonium salts have established explosion risk.[2] As the reaction produces gaseous byproducts N_2 and CO_2, even conducting reactions on a small scale in closed vials safety measures such as blast shields must be used. Although no explosions or violent decomposition occurred during these studies, we have observed that on a larger scale experiment, significant exotherm was observed and it is necessary to initiate the reaction by adding verdazyl catalyst to a cooled reaction mixture.

Arenediazonium salts used in this study and their preparation.

All arenediazonium salts used in this study are known compounds. 4-Nitrobenzenediazonium tetrafluoroborate **5v** was purchased from Sigma-Aldrich. The remaining arenediazonium salts were synthesized using general methods A-C and stored in a refrigerator. Since all arenediazonium salts except **5z** are known compounds, only the data for **5z** is provided. Alternatively, arenediazonium salts can be synthesized and used in situ according to general procedure **P3** or **P4.**

Table S1. List of arenediazonium salts used in this study

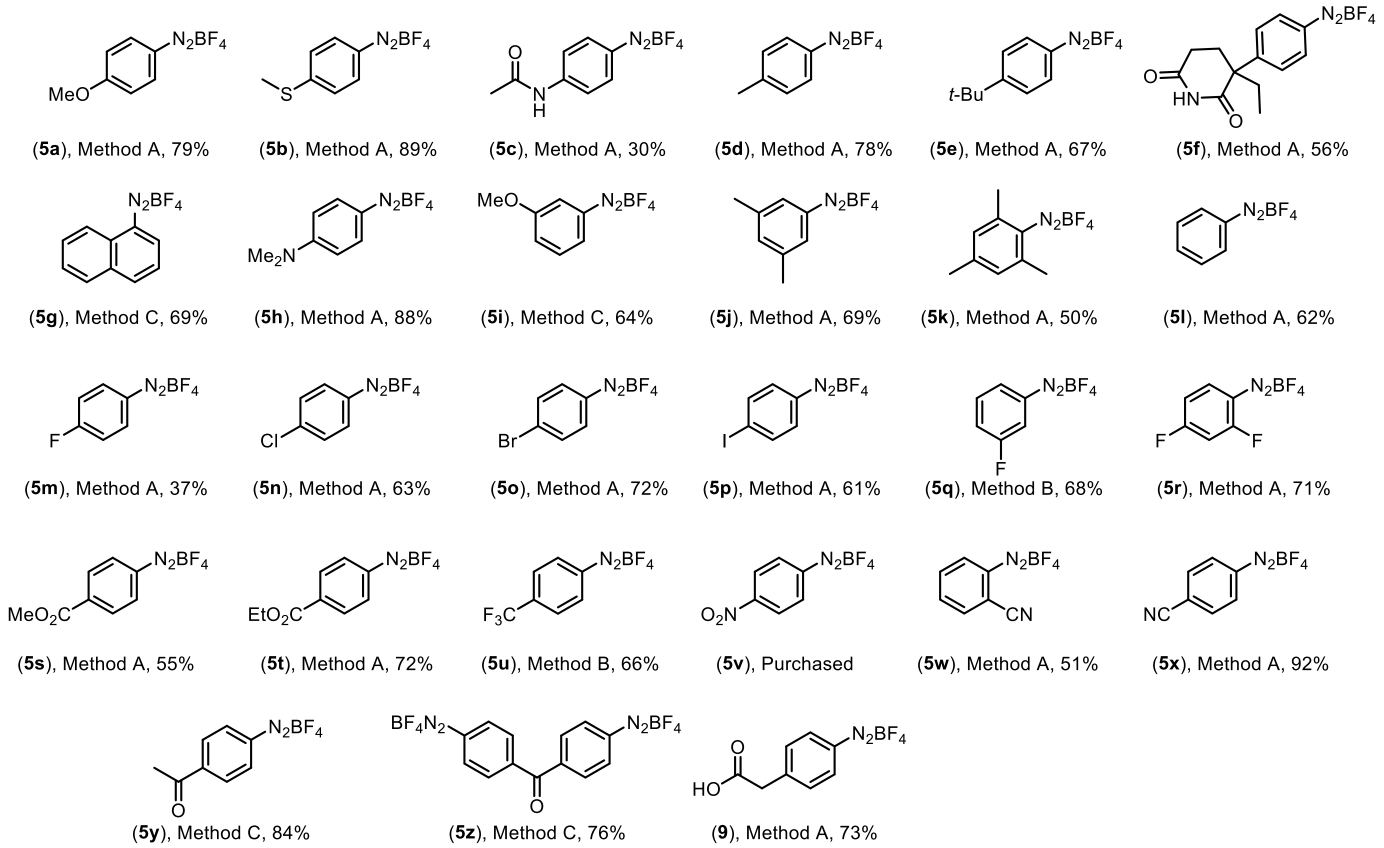

Method A: General procedure A using $NaNO_2/HBF_4$:

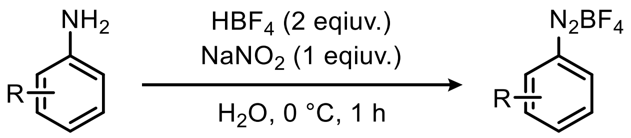

Arenediazonium salts **5a-5e**, **5h**, **5j-5o, 5r-5s** were synthesized according to the literature protocol.[3] The aniline (48.3 mmol) and deionized water (20 mL) were added in a 100 ml round-bottom flask, followed by

adding hydrofluoroboric acid (48 % wt. % in H_2O, 12.6 mL, 2 equiv.). The reaction mixture was dissolved and cooled to 0 °C, and then $NaNO_2$ solution (3.3 g, 48.3 mmol in 8 mL water) was added dropwise. It was left to stir at 0 °C for 1 h. The resulting precipitate was filtered and washed with cold water (2 mL x 3). If needed, the product was reprecipitated by dissolving in a minimum amount of acetone and then adding diethyl ether. The product was washed three times with the cold diethyl ether and dried under high vacuum overnight.

Method B: General procedure B using ^{t}BuONO/BF_3:

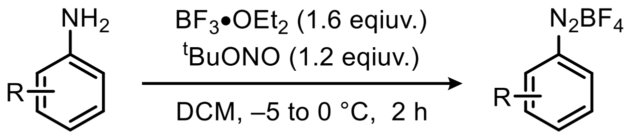

Arenediazonium salts **5g** and **5i** were synthesized according to the literature protocol,[4] with minor modification: the aniline (18 mmol, 1 equiv.) was added to an oven-dried 100 ml round bottom flask and dissolved in dry DCM (35 mL). The solution was cooled to −5 °C under a nitrogen atmosphere, BF_3•Et_2O (28.7 mmol, 3.6 mL, 1.6 equiv.) was added and stirred at this temperature for 15 minutes. ^{t}BuONO (21.5 mmol, 2.6 mL, 1.2 equiv) in DCM (5 mL) was added dropwise and the reaction mixture was stirred at −5 °C for 30 min and then at 0 °C for 1 h. The resulting precipitate was collected by filtration, washed with cold diethyl ether, and dried under a high vacuum overnight.

Method C: General procedure C using ^{t}BuONO/HBF_4:

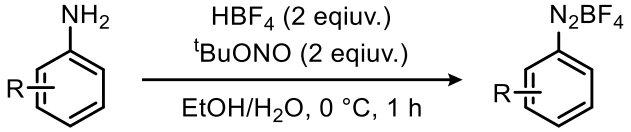

Arenediazonium salts **5p** and **5q** were synthesized according to the literature protocol.[5] The aniline (10.0 mmol) was dissolved in absolute ethanol (3.0 mL) in a 50 ml round-bottom flask, followed by adding hydrofluoroboric acid (48 % wt. % in H_2O, 2.5 mL, 2 equiv.). The reaction mixture was cooled to 0 °C, and then ^{t}BuONO (2.7 mL, 20.0 mmol, 2 equiv) was added dropwise. It was left to stir at 0 °C for 1 h. Diethyl ether (20mL) was added and the resulting precipitate was filtered and washed with ether. The product was dried under high vacuum overnight.

4,4'-Carbonyldibenzenediazonium ditetrafluoroborate (5z)

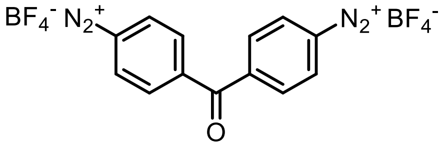

Synthesized using a modified general method C using acetone as a solvent. 4,4’-Diaminobenzophenone (1.06 g, 5.00 mmol, 1.0 equiv) was suspended in a mixture of acetone (15 mL) and hydrofluoroboric acid (48 % wt. in H_2O, 5.0 mL) and cooled to -10 °C using an ice-brine bath. While vigorously stirred, *t*-BuONO (3.57 mL, 30.0 mmol, 6.0 equiv) was added dropwise to observe precipitate formation. The slurry was stirred for 30 min at -10 °C, then filtered, washed with diethyl ether (2 × 10 mL), and air-dried for 5 min. The collected bis(diazonium) salt **5z**, as a beige solid, was further dried at high vacuum overnight.
^{1}H NMR (500 MHz, DMSO-d_6) δ 8.84 (d, J = 8.8 Hz, 4H), 8.26 (d, J = 8.8 Hz, 4H).
^{13}C{^{1}H} NMR (126 MHz, DMSO-d_6) δ 191.4, 144.2, 133.0, 131.8, 119.8.
^{11}B NMR (160 MHz, DMSO-d_6) δ 3.47.
^{19}F NMR (470 MHz, DMSO-d_6) δ -143.38.

General reaction procedure for borylation reactions*

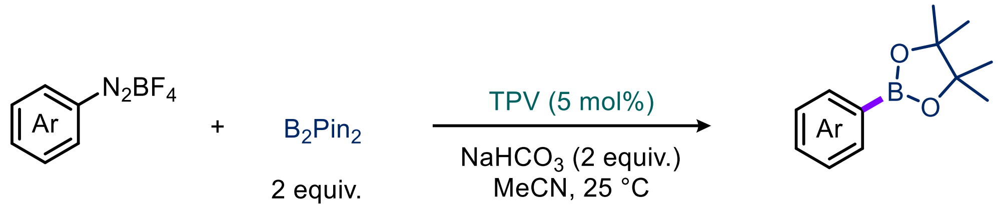

Under an Ar atmosphere inside a glovebox (or in a Schlenk flask under N_2 outside glovebox), an oven-dried 8 mL vial was charged with a magnetic stir bar, sodium bicarbonate (0.4 mmol, 33.6 mg, 2 equiv.), bis(pinacolato)diboron (0.4 mmol, 101.6 mg, 2 equiv.), dry and degassed MeCN (0.2 M), and the corresponding arenediazonium salt (0.2 mmol, 1 equiv.), followed by the addition of TPV (0.01 mmol, 3.1 mg, 0.05 equiv.). The vial was sealed with a septum cap and parafilm. The reaction mixture was taken outside the glovebox, and stirred at 25 °C for 24 h. The solvent was removed using a rotary evaporator, and the residue was purified by flash chromatography to afford the pure product.

**Reactions using other SET reductants were performed similarly by replacing TPV with the corresponding SET reductant.*

Comparison of TPV with different SET reductants on performance against a selected panel of substrates

Table S2. Selected substrate scope to compare different SET reductants*

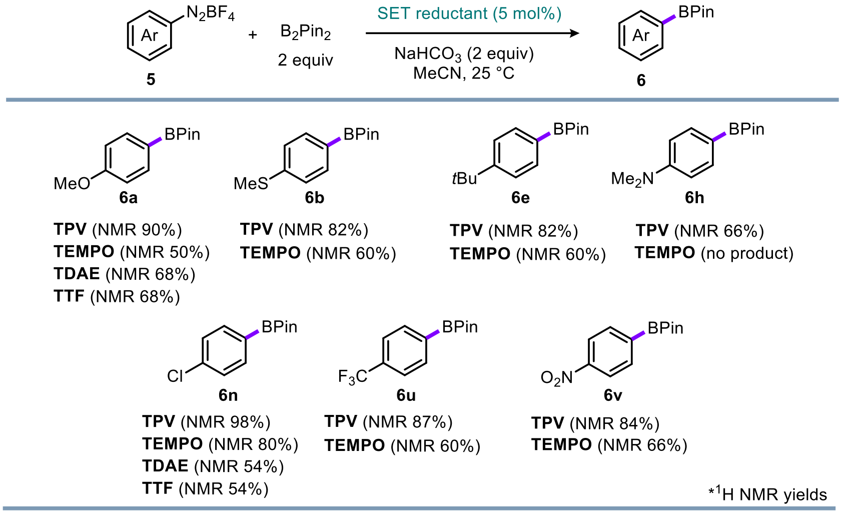

Data for Table 1

Table S3. Data for Table 1 in the manuscript

Time (min)	TPV (% Yield)	TTF (% Yield)	TDAE (% Yield)	TEMPO (% Yield)
**0**	0	0	0	0
**15**	24	16	26	8
**30**	28	24	28	9
**45**	30	28	32	16
**60**	38	29	34	16
**120**	46	34	40	22
**240**	56	44	48	28
**380**	72	50	54	34
**600**	80	56	62	40
**1080**	90	66	68	45
**1440**	92	68	68	50

Comparison of TPV with different SET reductants in borylation of phenyldiazonium tetrafluoroborate

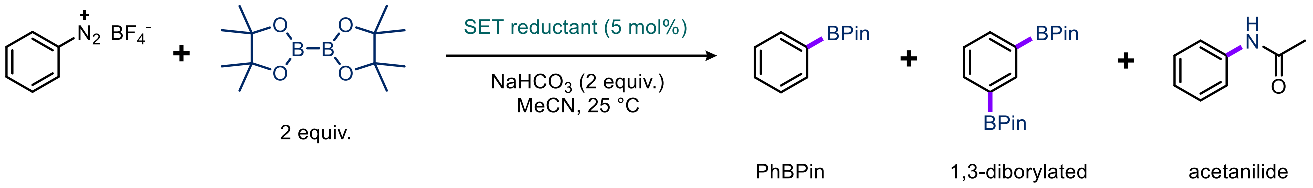

Following the general procedure for borylation, performed in a Schlenk flask under N_2 at 0.4 mmol and 5 mol% loading of initiator/catalyst. The reaction mixture was stirred at 25 °C for 16 h, the solvent was removed using a rotary evaporator, and the residue was directly analyzed by ^{1}H NMR using 1,2-DCE as internal standard. Analysis of crude reaction mixtures and isolation of the 1,3-diborylated byproduct confirmed its identity in agreement with the literature. Additionally, we discovered the formation of significant amounts of acetanilide in the case of TPV, TTF and TEMPO but not with TDAE. Such a discrepancy in product distribution with TDAE is also in agreement with the distinctly different course of reaction compared to other three organic electron donors.

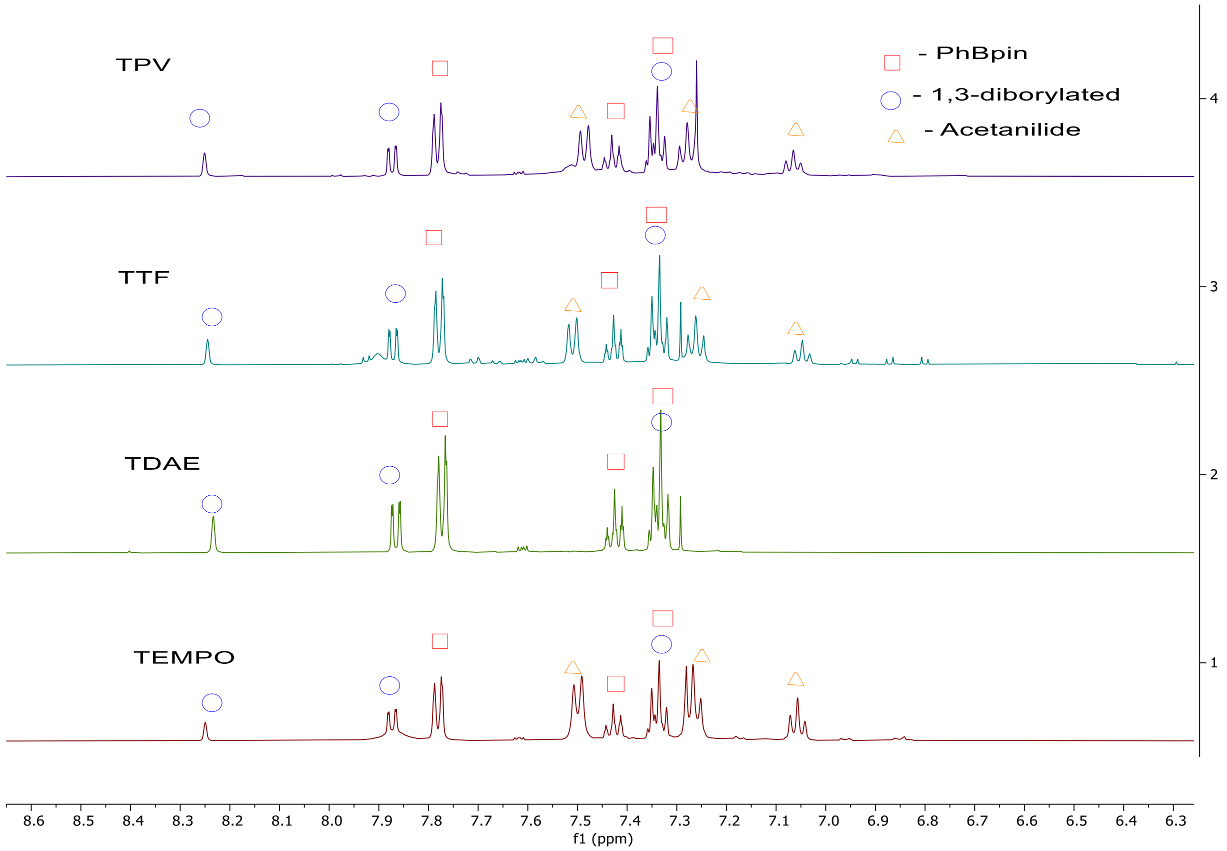

Figure S1. ^{1}H NMR (500 MHz, $CDCl_3$) analysis of product distribution in crude reactions mixtures from different SET reductants.

Table S4. NMR yields of the products from Figure S1.

Catalyst/Initiator	PhBPin (desired)	1,3-diborylated	Acetanilide
TPV	36%	15%	33%
TTF	40%	17%	27%
TDAE	61%	25%	n.d.
TEMPO	32%	19%	48%

1,3-Bis(4,4,5,5-tetramethyl-1,3,2-dioxaborolan-2-yl)benzene

Isolated from the reaction with TPV that was conducted according to the general procedure (**P1**) using diazonium salt PhN_2BF_4 at 0.4 mmol scale (76.8 mg). The crude product was purified by silica gel column chromatography (4% Et_2O, *n*-pentane, R_f: 0.16), the 1,3-diborylated product (15.3 mg, 11% yield) as a yellow oil (contains 10% 1,2-diborylated isomer as an inseparable mixture).

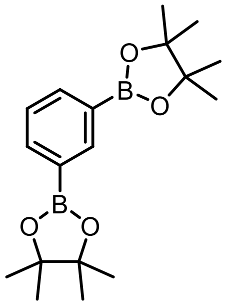

^{1}H NMR (500 MHz, $CDCl_3$) δ 8.28 (s, 1H), 7.90 (dd, *J* = 7.4, 1.3 Hz, 2H), 7.37 (td, *J* = 7.4, 0.5 Hz, 2H), 1.34 (s, 24H).

^{13}C{^{1}H} NMR (126 MHz, $CDCl_3$) δ 141.4, 137.8, 127.2, 83.9, 25.0. The carbon directly attached to the boron atom was not detected due to quadrupolar broadening.

^{11}B NMR (160 MHz, $CDCl_3$) δ 30.29.

ESI-HRMS (m/z): calculated for calculated for $C_{18}H_{29}B_2O_4^+$ ([M+H]$^+$) 331.2246, found: 331.2259.

The data matched the literature.[7]

GC–MS Analysis of competition experiments

GC–MS analysis was performed using DCM as the injection solvent to determine product ratios and approximate yields for compounds **6d** and **6n**. Each compound was first injected individually to establish its characteristic retention time. A 1:1 mixture of **6d**:**6n** was then analysed to determine their relative response factor, which was found to be 0.98 ~1.00. In a separate calibration experiment, a 1:1 mixture of B_2pin_2 and **6n** was injected, and the response factor was determined to be 1.58 (a separate ^{1}H NMR analysis of this mixture confirmed their 1:1 ratio). For reaction analysis, an aliquot of each crude reaction mixture was withdrawn, diluted with DCM, and injected under identical GC–MS conditions. Figure S2 displays the GC–MS chromatograms and peak integrations for each analysed sample. The resulting product ratios and approximate yields were calculated using the established response factors and the conversion of the limiting reagent B_2Pin_2 and are summarized in Table S5.

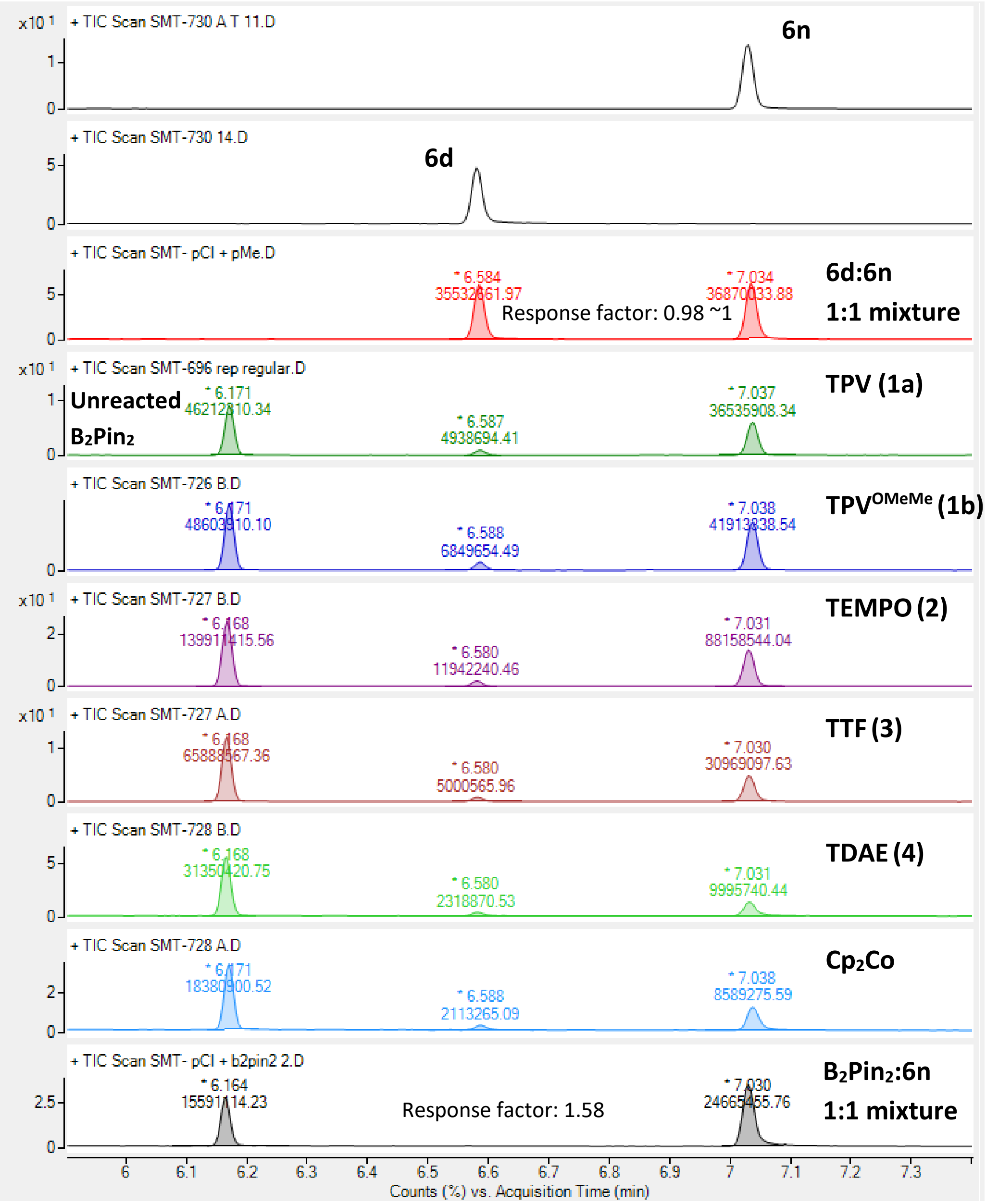

Figure S2. GC-MS analysis of competition experiments.

Table S5. Data for Figure S2

SET reductant	**6d** yield (%)	**6n** yield (%)	**6d:6n**	Total yield (%)
TPV **(1a)**	4	32	1:8	36
TPV^{OMeMe} **(1b)**	5	34	1:6.8	39
TEMPO **(2)**	4	27	1:6.7	31
TTF **(3)**	4	22	1:5.5	26
TDAE **(4)**	4	16	1:4	20
Cp_2Co	5	22	1:4.4	27

Detection of adducts of aryl radical with SET reductants using mass spectrometry

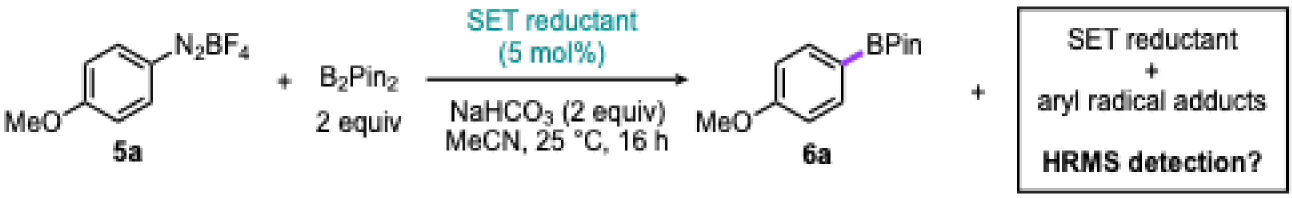

Following the general procedure for borylation, reactions were set up in an Ar filled glovebox using 8 mL reaction vials with diazonium salt **5a** (0.4 mmol) and 5 mol% loading of SET reductant. The vials were sealed, taken out of the glovebox and stirred at 25 °C for 16 h. The vials were opened to air, a small aliquot was filtered and diluted with MeCN for HRMS analysis (ESI-HRMS). The results are summarized in Figure S3 shown below.

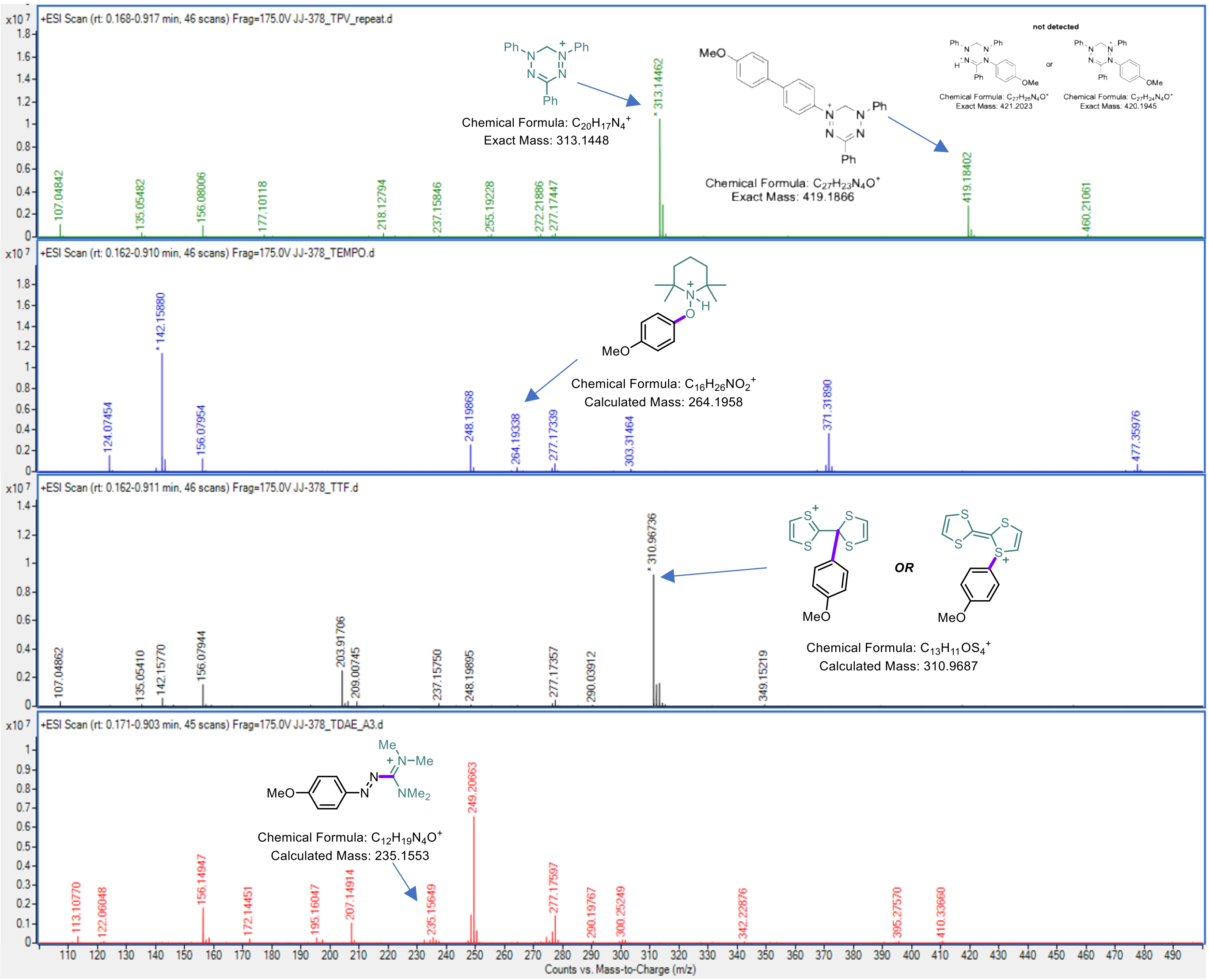

Figure S3. HRMS analysis of aryl radical adducts to SET reductants.

In the reaction with TPV, the expected N-coupled TPV-Ar adducts neither matching the formula $C_{27}H_{25}N_4O^+$ ($[M+H]^+$) 421.2023 or $C_{27}H_{25}N_4O^+$ ($[M]^+$) 420.1945 *are not detected*. Instead, along with unmodified TPV^+, a mass fragment was detected potentially matching the calculated formula $C_{27}H_{23}N_4O^+$ 419.1866, found: 419.1840 which is in agreement of phenyl-ring arylation of TPV. This is inconsequential for catalysis as this species can also participate in catalytic turnover along with TPV.

In the reaction with TDAE, instead of the covalent adduct, a possible product of TDAE addition to diazonium followed by fragmentation is observed. Calculated for $C_{12}H_{19}N_4O^+$ ([M]$^+$): 235.1553, found: 235.1565.
In the reaction with TEMPO, the covalent Ar–OTMP adduct was detected. Calculated for $C_{16}H_{26}NO_2^+$ ([M+H]$^+$): 264.1958, found: 264.1934.
In the reaction with TTF, the covalent adduct was detected. Calculated for $C_{13}H_{11}OS_4^+$ ([M]$^+$): 310.9687, found: 310.9673.

Probing the chain-repair catalysis scenario with verdazylium tetrafluoroborate ($TPV^+BF_4^-$)

The catalytic chain repair ability of the TPV/TPV$^+$ redox system experimentally probed using 5 mol% independently synthesized verdazylium tetrafluoroborate **1a**$^+$ **BF**$_4$ in place of TPV. Following the general procedure for borylation, reactions with diazonium salt **5a** (0.4 mmol) and 5 mol% of either TPV or TPVBF$_4$ (**1a**$^+$**BF**$_4^-$) and 1,2-dichloroethane (31.5 µL, 0.4 mmol, 1.0 equiv) as internal standard were set up in an Ar filled glovebox in 8 mL reaction vials. The vials were stirred at 25 °C with removal of *ca.* 100 µL aliquot for NMR analysis at specific time points. The results are plotted in Scheme S1.
Under these conditions, arylpinacolboronate **6a** was formed in a comparable yield (94% NMR yield) despite exhibiting an expected induction period. These results support the *in situ* generation of TPV via SET reduction of TPV$^+$ by reducing boryl species under the reaction conditions.

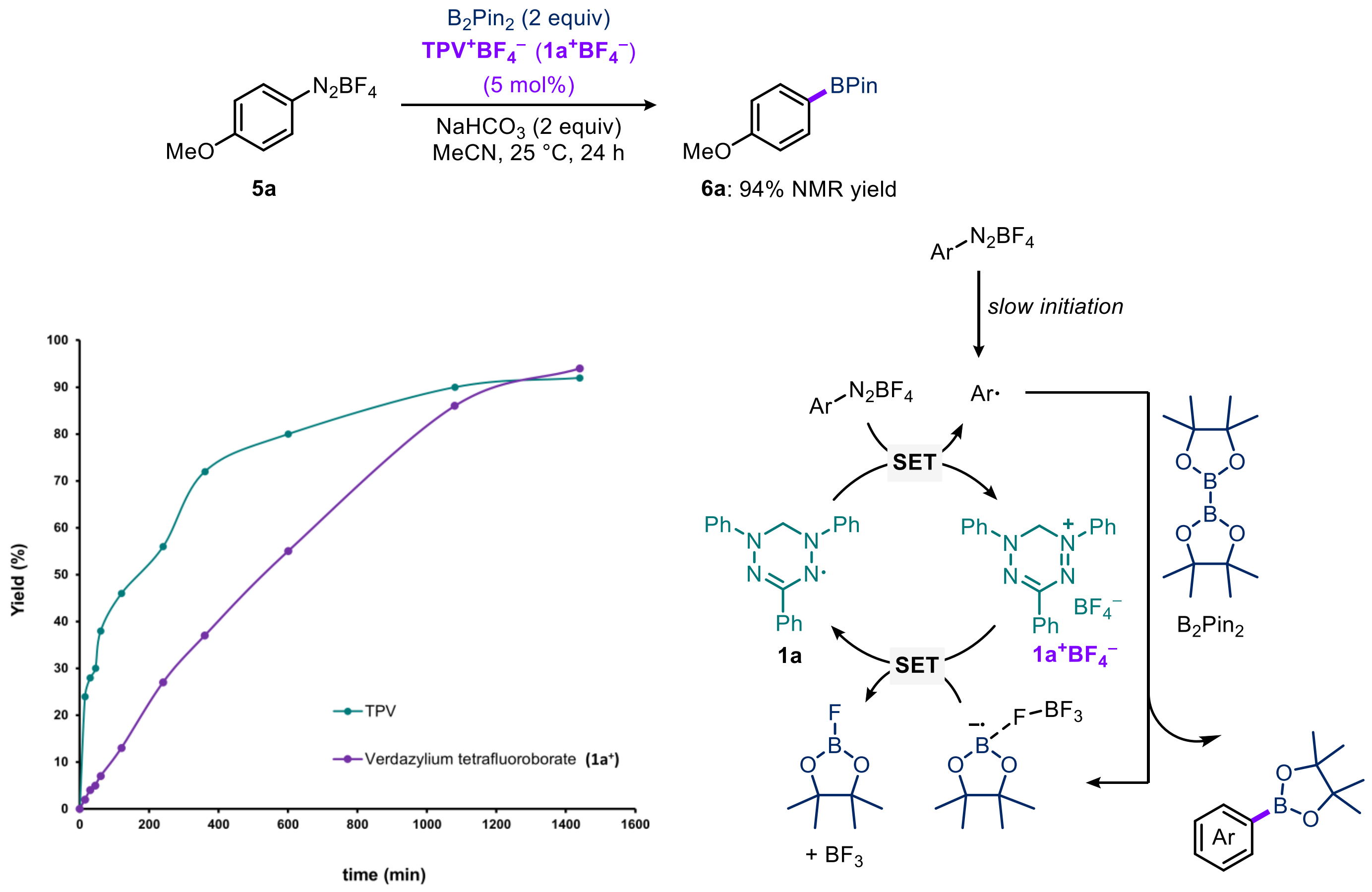

Scheme S1. Chain-repair catalysis scenario using verdazylium tetrafluoroborate ($TPV^+BF_4^-$)

Reduction of verdazylium tetrafluoroborate ($TPV^+BF_4^-$) by $^{4\text{-}Ph}$Pyridine/B_2Pin_2 adduct

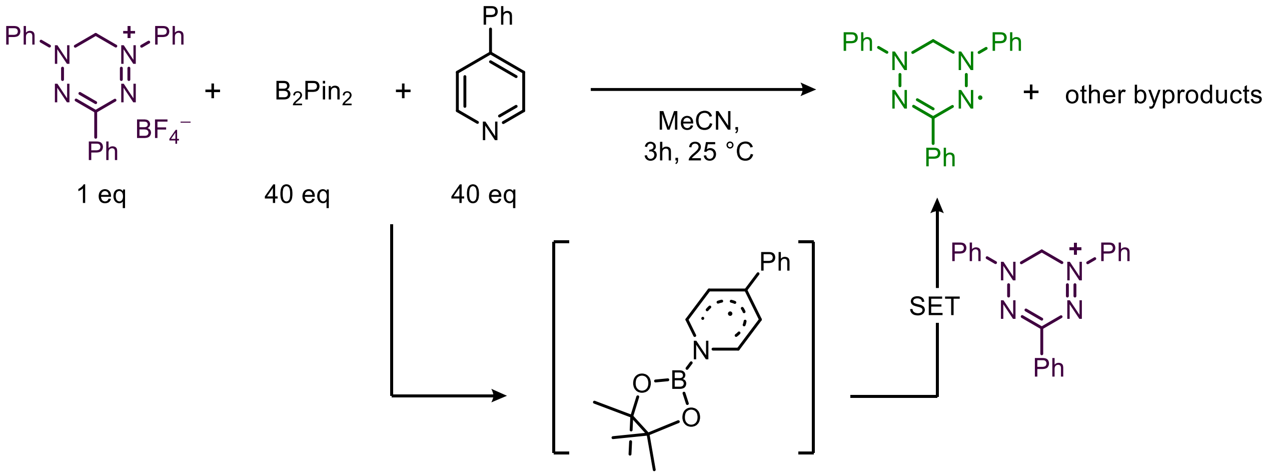

Scheme S2. Reduction of verdazylium tetrafluoroborate ($TPV^+BF_4^-$) with B_2Pin_2/4-phenylpyridine mixture

Imitating the reaction conditions, an oven-dried Schlenk flask was charged with $TPVBF_4$ (10 mg, 0.025 mmol, 1.0 equiv.) and 4-phenylpyridine (155mg, 1.00 mmol, 40 equiv), evacuated-backfilled with N_2 three times and finally degassed, dry MeCN (2 mL) was added. Stirred under inert atmosphere, the color change was monitored. At 3 h mark a complete transition from purple to green was observed (Figure S4).

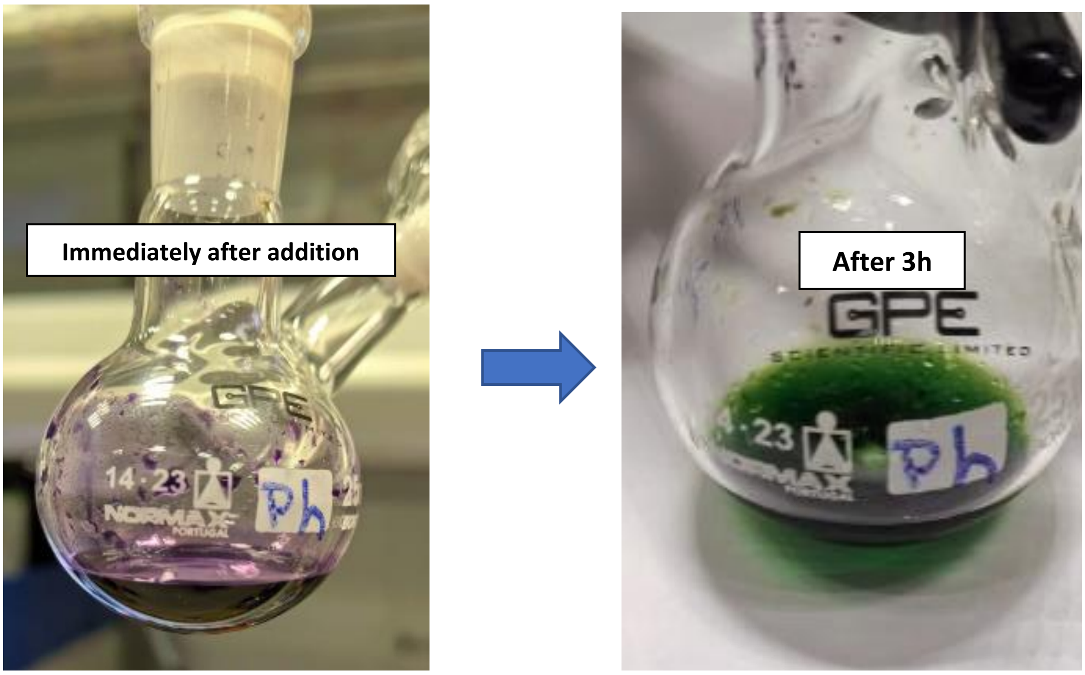

Figure S4. Color change observed during the reduction of verdazylium tetrafluoroborate ($TPV^+BF_4^-$) with B_2Pin_2/4-phenylpyridine mixture.

Data for borylation reactions

2-(4-Methoxyphenyl)-4,4,5,5-tetramethyl-1,3,2-dioxaborolane (6a)

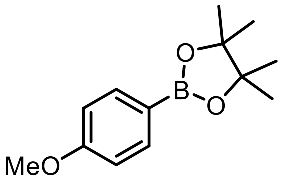

Synthesized according to the general procedure (**P1**) using diazonium salt **5a** (0.4 mmol scale, 89 mg). The crude product was purified by silica gel column chromatography (10% Et_2O, *n*-pentane, R_f: 0.28), affording **6a** (83 mg, 88%) as a white solid.

^{1}H NMR (500 MHz, $CDCl_3$) δ 7.76 (d, *J* = 8.7 Hz, 2H), 6.98 – 6.80 (m, 2H), 3.83 (s, 3H), 1.33 (s, 12H).

^{13}C{^{1}H} NMR (126 MHz, $CDCl_3$) δ 162.3, 136.6, 113.4, 83.7, 55.2, 25.0. The carbon directly attached to the boron atom was not detected due to quadrupolar broadening.

^{11}B NMR (160 MHz, $CDCl_3$) δ 30.57.

ESI-HRMS (m/z): calculated for $C_{13}H_{20}BO_3^+$ ($[M+H]^+$): 235.1500, found: 235.1505.

The data matched the literature.[6]

4,4,5,5-Tetramethyl-2-(4-(methylthio)phenyl)-1,3,2-dioxaborolane (6b)

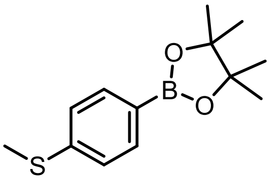

Synthesized according to the general procedure (**P1**) using diazonium salt **5b** (0.4 mmol scale, 95.2 mg). The crude product was purified by silica gel column chromatography (10% Et_2O, *n*-pentane, R_f: 0.45), affording **6b** (78 mg, 78%) as a yellow oil.

^{1}H NMR (500 MHz, $CDCl_3$) δ 7.71 (d, *J* = 8.4 Hz, 2H), 7.22 (d, *J* = 8.4 Hz, 2H), 2.49 (s, 3H), 1.34 (s, 12H).

^{13}C{^{1}H} NMR (126 MHz, $CDCl_3$) δ 142.7, 135.2, 125.1, 83.9, 25.0, 15.2. The carbon directly attached to the boron atom was not detected due to quadrupolar broadening.

^{11}B NMR (160 MHz, $CDCl_3$) δ 31.01.

ESI-HRMS (m/z): calculated for $C_{13}H_{20}BO_2S^+$ ($[M+H]^+$) 251.1272, found: 251.1246.

The data matched the literature.[7]

N-(4-(4,4,5,5-Tetramethyl-1,3,2-dioxaborolan-2-yl)phenyl)acetamide (6c)

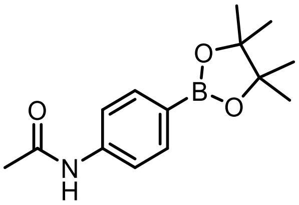

Synthesized according to the general procedure (**P1**) using diazonium salt **5c** (0.4 mmol scale, 99.6 mg). The crude product was purified by silica gel column chromatography (50% EtOAc, *n*-pentane, R_f: 0.34), affording **6c** (80 mg, 77%) as a white solid.

^{1}H NMR (500 MHz, $CDCl_3$) δ 7.76 (d, *J* = 8.4 Hz, 2H), 7.51 (d, *J* = 8.5 Hz, 2H), 2.18 (s, 3H), 1.33 (s, 12H).

^{13}C{^{1}H} NMR (126 MHz, $CDCl_3$) δ 168.5, 140.6, 135.9, 118.7, 83.9, 25.0, 24.7. The carbon directly attached to the boron atom was not detected due to quadrupolar broadening.

^{11}B NMR (160 MHz, $CDCl_3$) δ 31.02.

ESI-HRMS (m/z): calculated for $C_{14}H_{21}BNO_3^+$ $[M+H]^+$ 262.1609, found: 262.1635.

The data matched the literature.[7]

4,4,5,5-Tetramethyl-2-(p-tolyl)-1,3,2-dioxaborolane (6d)

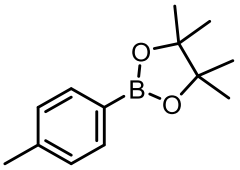

Synthesized according to the general procedure (**P1**) using diazonium salt **5d** (0.4 mmol scale, 82.4 mg). The crude product was purified by silica gel column chromatography (5% Et_2O, *n*-pentane, R_f: 0.62), affording **6d** (66 mg, 76%) as a white solid.
^{1}H NMR (500 MHz, $CDCl_3$) δ 7.74 – 7.66 (m, 2H), 7.21 – 7.15 (m, 2H), 2.36 (s, 3H), 1.34 (s, 12H).
^{13}C{^{1}H} NMR (126 MHz, $CDCl_3$) δ 141.5, 134.9, 128.7, 83.8, 25.0, 21.9. The carbon directly attached to the boron atom was not detected due to quadrupolar broadening.
^{11}B NMR (160 MHz, $CDCl_3$) δ 30.89.
ESI-HRMS (m/z): calculated for $C_{13}H_{20}BO_2^+$ ($[M+H]^+$) 219.1551, found: 219.1548.
The data matched the literature.[8]

2-(4-(*tert*-Butyl)phenyl)-4,4,5,5-tetramethyl-1,3,2-dioxaborolane (6e)

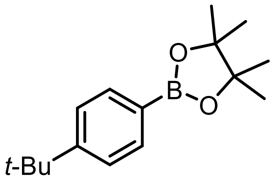

Synthesized according to the general procedure (**P1**) using diazonium salt **5e** (0.2 mmol scale, 49.6 mg). The crude product was purified by silica gel column chromatography (1% Et_2O, *n*-pentane, R_f: 0.4), affording **6e** (40.3 mg, 77%) as a yellow solid.
^{1}H NMR (500 MHz, $CDCl_3$) δ 7.77 (d, *J* = 8.3 Hz, 2H), 7.42 (d, *J* = 8.3 Hz, 2H), 1.35 (s, 12H), 1.34 (s, 9H).
^{13}C{^{1}H} NMR (126 MHz, $CDCl_3$) δ 154.6, 134.8, 124.8, 83.7, 35.0, 31.3, 25.0. The carbon directly attached to the boron atom was not detected due to quadrupolar broadening.
^{11}B NMR (160 MHz, $CDCl_3$) δ 30.92.
ESI-HRMS (m/z): calculated for $C_{16}H_{26}BO_2^+$ ($[M+H]^+$) 261.2020, found: 261.2010.
The data matched the literature.[9]

3-Ethyl-3-(4-(4,4,5,5-tetramethyl-1,3,2-dioxaborolan-2-yl)phenyl)piperidine-2,6-dione (6f)

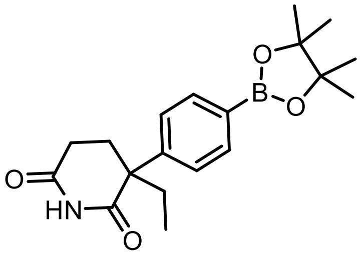

Synthesized according to the general procedure (**P1**) using diazonium salt **5f** (0.4 mmol scale, 132.4 mg). The crude product was purified by silica gel column chromatography (35% EtOAc, *n*-pentane, R_f: 0.2), affording **6f** (105 mg, 77%) as a white solid.
^{1}H NMR (500 MHz, $CDCl_3$) δ 7.84 (br. s, 1H), 7.81 (d, *J* = 8.4 Hz, 2H), 7.28 (d, *J* = 8.4 Hz, 1H), 2.61 – 2.55 (m, 1H), 2.42 – 2.33 (m, 2H), 2.24 (dd, *J* = 14.4, 3.3 Hz, 1H), 2.10 – 2.03 (m, 1H), 1.93 (dt, *J* = 14.0, 7.4 Hz, 1H), 1.34 (s, 12H), 0.87 (t, *J* = 7.4 Hz, 3H).
^{13}C{^{1}H} NMR (126 MHz, $CDCl_3$) δ 175.0, 172.2, 142.0, 135.6, 125.6, 84.1, 51.6, 32.9, 29.4, 27.2, 25.0, 9.2. The carbon directly attached to the boron atom was not detected due to quadrupolar broadening.
^{11}B NMR (160 MHz, $CDCl_3$) δ 30.63.

ESI-HRMS (m/z): calculated for $C_{19}H_{27}BNO_4^+$ ($[M+H]^+$) 344.2028, found: 344.2013.

4,4,5,5-Tetramethyl-2-(naphthalen-1-yl)-1,3,2-dioxaborolane (6g)

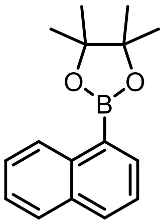

Synthesized according to the general procedure (**P1**) using diazonium salt **5g** (0.4 mmol scale, 96.8 mg). The crude product was purified by silica gel column chromatography (2% Et_2O, *n*-pentane, R_f: 0.6), affording **6g** (65.0 mg, 64%) as an orange solid.
^{1}H NMR (500 MHz, $CDCl_3$) δ 8.83 (d, *J* = 8.4 Hz, 1H), 8.14 (d, *J* = 6.2 Hz, 1H), 7.97 (d, *J* = 8.2 Hz, 1H), 7.87 (d, *J* = 8.1 Hz, 1H), 7.58 (t, *J* = 7.6 Hz, 1H), 7.55 – 7.45 (m, 2H), 1.46 (s, 12H).
^{13}C{^{1}H} NMR (126 MHz, $CDCl_3$) δ 137.0, 135.8, 133.3, 131.7, 128.54, 128.47, 126.5, 125.6, 125.1, 83.8, 25.1. The carbon directly attached to the boron atom was not detected due to quadrupolar broadening.
^{11}B NMR (160 MHz, $CDCl_3$) δ 31.59.
ESI-HRMS (m/z): calculated for $C_{16}H_{20}BO_2^+$ ($[M+H]^+$) 255.1551, found: 255.1538.
The data matched the literature.[10]

N,N-Dimethyl-4-(4,4,5,5-tetramethyl-1,3,2-dioxaborolan-2-yl)aniline (6h)

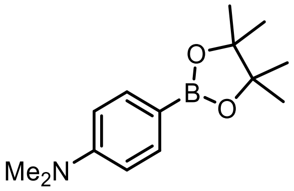

Synthesized according to the general procedure (**P1**) using diazonium salt **5h** (0.4 mmol scale, 94 mg. The crude product was purified by silica gel column chromatography (7% Et_2O, *n*-pentane, R_f: 0.3), affording **6h** (56 mg, 56%) as a white solid.
^{1}H NMR (500 MHz, $CDCl_3$) δ 7.70 (d, *J* = 8.3 Hz, 2H), 6.71 (d, *J* = 8.1 Hz, 2H), 2.99 (s, 6H), 1.33 (s 12H).
^{13}C{^{1}H} NMR (126 MHz, $CDCl_3$) δ 152.6, 136.3, 111.5, 83.3, 40.3, 25.0. The carbon directly attached to the boron atom was not detected due to quadrupolar broadening.
^{11}B NMR (160 MHz, $CDCl_3$) δ 30.85. The data matched those reported in the literature.[6]
ESI-HRMS (m/z): calculated for $C_{14}H_{23}BNO_2^+$ ($[M+H]^+$) 248.1816, found: 248.1827.
The data matched the literature.[6]

2-(3-Methoxyphenyl)-4,4,5,5-tetramethyl-1,3,2-dioxaborolane (6i)

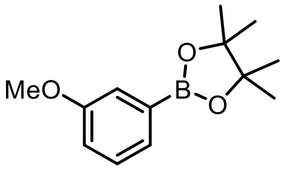

Synthesized according to the general procedure (**P1**) using diazonium salt **5i** (0.4 mmol scale, 88.8 mg). The crude product was purified by silica gel column chromatography (3% Et_2O, *n*-pentane, R_f: 0.4), affording **6i** (41.4 mg, 44%) as a colorless oil.
^{1}H NMR (500 MHz, $CDCl_3$) δ 7.40 (d, *J* = 7.2 Hz, 1H), 7.33 (d, *J* = 2.9 Hz, 1H), 7.31 – 7.27 (m, 1H), 7.01 (ddd, *J* = 8.2, 2.8, 1.1 Hz, 1H), 3.83 (s, 3H), 1.35 (s, 12H).

$^{13}C\{^1H\}$ NMR (126 MHz, $CDCl_3$) δ 159.2, 129.1, 127.3, 118.8, 118.1, 84.0, 55.4, 25.0. The carbon directly attached to the boron atom was not detected due to quadrupolar broadening.
^{11}B NMR (160 MHz, $CDCl_3$) δ 31.13.
ESI-HRMS (m/z): calculated for $C_{13}H_{20}BO_3^+$ ($[M+H]^+$) 235.1500, found: 235.1506.
The data matched the literature.[9, 11]

2-(3,5-Dimethylphenyl)-4,4,5,5-tetramethyl-1,3,2-dioxaborolane (6j)

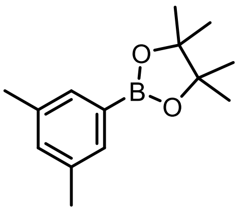

Synthesized according to the general procedure (**P1**) using diazonium salt **5j** (0.4 mmol scale, 88 mg. The crude product was purified by silica gel column chromatography (5% Et_2O, *n*-pentane, R_f: 0.69), affording **6j** (37 mg, 40%) as a colorless oil.
^1H NMR (500 MHz, $CDCl_3$) δ 7.46 – 7.42 (m, 2H), 7.10 (s, 1H), 2.32 (s, 6H), 1.34 (s, 12H).
$^{13}C\{^1H\}$ NMR (126 MHz, $CDCl_3$) δ 137.3, 133.1, 132.5, 83.8, 25.0, 21.3. The carbon directly attached to the boron atom was not detected due to quadrupolar broadening.
^{11}B NMR (160 MHz, $CDCl_3$) δ 31.27.
ESI-HRMS (m/z): calculated for $C_{14}H_{25}BNO_2^+$ ($[M+NH_4]^+$) 250.1973, found: 250.1948.
The data matched the literature.[12]

2-Mesityl-4,4,5,5-tetramethyl-1,3,2-dioxaborolane (6k)

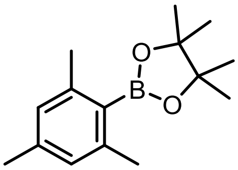

Synthesized according to the general procedure (**P1**) using diazonium salt **5k** (0.4 mmol scale, 137 mg). The crude product was purified by silica gel column chromatography (5% Et_2O, *n*-pentane, R_f: 0.69), affording **6k** (40 mg, 41%) as a colorless oil.
^1H NMR (500 MHz, $CDCl_3$) δ 6.77 (s, 2H), 2.36 (s, 6H), 2.24 (s, 3H), 1.37 (s, 12H).
$^{13}C\{^1H\}$ NMR (126 MHz, $CDCl_3$) δ 142.6, 140.0, 127.6, 83.6, 25.1, 22.3, 21.4. The carbon directly attached to the boron atom was not detected due to quadrupolar broadening.
^{11}B NMR (160 MHz, $CDCl_3$) δ 32.45.
ESI-HRMS (m/z): calculated for $C_{15}H_{24}BO_2^+$ ($[M+H]^+$) 247.1864, found: 247.1867.
The data matched the literature.[10]

2-(4-Fluorophenyl)-4,4,5,5-tetramethyl-1,3,2-dioxaborolane (6m)

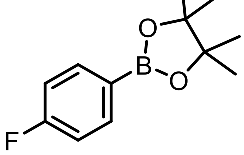

Synthesized according to the general procedure (**P1**) using diazonium salt **5l** (0.4 mmol scale, 84.0 mg). The crude product was purified by silica gel column chromatography (5% Et_2O, *n*-pentane, R_f: 0.62), affording **6l** (61 mg, 69%) as a colorless oil.
^1H NMR (500 MHz, $CDCl_3$) δ 7.79 (dd, *J* = 8.7, 6.3 Hz, 2H), 7.05 (t, *J* = 8.9 Hz, 2H), 1.34 (s, 13H).

^{13}C{^{1}H} NMR (126 MHz, $CDCl_3$) δ 165.2 (d, *J* = 250.2 Hz), 137.1 (d, *J* = 8.2 Hz), 115.0 (d, *J* = 20.0 Hz) 83.9, 24.9. The carbon directly attached to the boron atom was not detected due to quadrupolar broadening.
^{11}B NMR (160 MHz, $CDCl_3$) δ 30.72.
ESI-HRMS (m/z): calculated for $C_{12}H_{17}BFO_2^+$ ($[M+H]^+$) 223.1300, found: 223.1257.
The data matched the literature.[10]

2-(4-Chlorophenyl)-4,4,5,5-tetramethyl-1,3,2-dioxaborolane (6n)

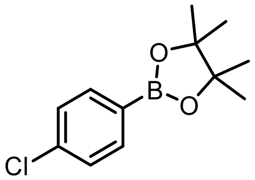

Synthesized according to the general procedure (**P1**) using diazonium salt **5n** (0.4 mmol scale, 90.5 mg). The crude product was purified by silica gel column chromatography (7% Et_2O, *n*-pentane, R_f: 0.46), affording **6n** (89 mg, 93%) as a white solid.
^{1}H NMR (500 MHz, $CDCl_3$) δ 7.72 (d, *J* = 8.3 Hz, 2H), 7.34 (d, *J* = 8.3 Hz, 2H), 1.34 (s, 12H).
^{13}C{^{1}H} NMR (126 MHz, $CDCl_3$) δ 137.7, 136.3, 128.2, 84.2, 25.0. The carbon directly attached to the boron atom was not detected due to quadrupolar broadening.
^{11}B NMR (160 MHz, $CDCl_3$) δ 30.48.
ESI-HRMS (m/z): calculated for $C_{12}H_{17}BClO_2^+$ ($[M+H]^+$): 239.1005, found: 239.0992.
The data matched the literature.[6]

2-(4-Bromophenyl)-4,4,5,5-tetramethyl-1,3,2-dioxaborolane (6o)

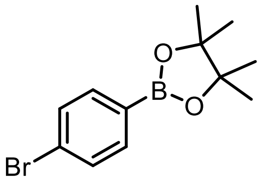

Synthesized according to the general procedure (**P1**) using diazonium salt **5o** (0.4 mmol scale, 108 mg). The crude product was purified by silica gel column chromatography (1% Et_2O, *n*-pentane, R_f: 0.4), affording **6o** (96 mg, 85%) as a white solid.
^{1}H NMR (500 MHz, $CDCl_3$) δ 7.66 (d, *J* = 8.3 Hz, 2H), 7.51 (d, *J* = 8.3 Hz, 2H), 1.34 (s, 12H).
^{13}C{^{1}H} NMR (126 MHz, $CDCl_3$) δ 136.4, 131.1, 126.4, 84.2, 25.0. The carbon directly attached to the boron atom was not detected due to quadrupolar broadening.
^{11}B NMR (160 MHz, $CDCl_3$) δ 30.91.
ESI-HRMS (m/z): calculated for $C_{12}H_{17}BBrO_2^+$ ($[M+H]^+$) 283.0499, found: 283.0487.
The data matched the literature.[9]

2-(4-Iodophenyl)-4,4,5,5-tetramethyl-1,3,2-dioxaborolane (6p)

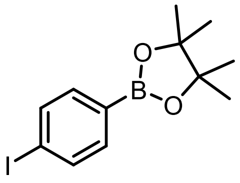

Synthesized according to the general procedure (**P1**) using diazonium salt **5p** (0.4 mmol scale, 68.8 mg). The crude product was purified by silica gel column chromatography (1% Et_2O, *n*-pentane, R_f: 0.4), affording **6p** (68.8 mg, 52%) as a yellow solid.
^{1}H NMR (500 MHz, $CDCl_3$) δ 7.72 (d, *J* = 8.1 Hz, 2H), 7.51 (d, *J* = 8.1 Hz, 2H), 1.33 (s, 12H).

$^{13}C\{^1H\}$ **NMR** (126 MHz, $CDCl_3$) δ 137.0, 136.4, 99.0, 84.2, 25.0. The carbon directly attached to the boron atom was not detected due to quadrupolar broadening.
^{11}B **NMR** (160 MHz, $CDCl_3$) δ 30.92.
ESI-HRMS (m/z): calculated for $C_{12}H_{17}BIO_2^+$ $[M+H]^+$ 331.0361, found: 331.0342.
The data matched the literature.[8, 9]

2-(3-Fluorophenyl)-4,4,5,5-tetramethyl-1,3,2-dioxaborolane (6q)

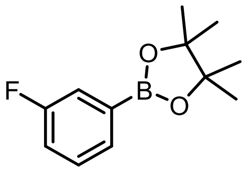

Synthesized according to the general procedure (**P1**) using diazonium salt **5q** (0.4 mmol scale, 84 mg). The crude product was purified by silica gel column chromatography (5% Et_2O, *n*-pentane, R_f: 0.6), affording **6q** (52 mg, 59%) as a colorless liquid.
^1H **NMR** (500 MHz, $CDCl_3$) δ 7.57 (d, J = 7.3 Hz, 1H), 7.48 (dd, J = 9.2, 2.8 Hz, 1H), 7.38 – 7.30 (m, 1H), 7.14 (m, 1H), 1.35 (s, 12H).
$^{13}C\{^1H\}$ **NMR** (126 MHz, $CDCl_3$) δ 162.6 (d, J = 246.4 Hz), 130.4 (d, J = 3.0 Hz), 129.6 (d, J = 7.0 Hz), 121.1 (d, J = 19 Hz), 118.3 (d, J = 21.1 Hz), 84.2, 25.0. The carbon directly attached to the boron atom was not detected due to quadrupolar broadening.
^{11}B **NMR** (160 MHz, $CDCl_3$) δ 30.45.
^{19}F **NMR** (470 MHz, $CDCl_3$) δ -114.2 (m, 1F).
ESI-HRMS (m/z): calculated for $C_{12}H_{20}BFNO_2^+$ ($[M+NH_4]^+$) 240.1566, found: 240.1533.
The data matched the literature.[8, 9]

2-(2,4-Difluorophenyl)-4,4,5,5-tetramethyl-1,3,2-dioxaborolane (6r)

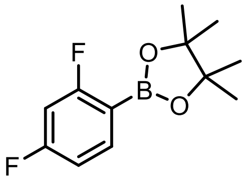

Synthesized according to the general procedure (**P1**) using diazonium salt **5r** (0.4 mmol scale, 91.2 mg). The crude product was purified by silica gel column chromatography (4% Et_2O, *n*-pentane, R_f: 0.5), affording **6r** (48.8 mg, 51%) as an orange oil.
^1H **NMR** (500 MHz, $CDCl_3$) δ 7.73 (dt, J = 8.5, 7.0 Hz, 1H), 6.86 (td, J = 8.3, 2.3 Hz, 1H), 6.76 (td, J = 9.5, 2.3 Hz, 1H), 1.35 (s, 12H).
$^{13}C\{^1H\}$ **NMR** (126 MHz, $CDCl_3$) δ 169.0 (d, J = 12.0 Hz), 166.9 (dd, J = 35.4, 12.3 Hz), 164.7 (d, J = 12.4 Hz), 138.3 (t, J = 10.1 Hz), 111.3 (dd, J = 20.2, 3.5 Hz), 103.8 (dd, J = 27.9, 24.3 Hz), 84.1, 25.0. ^{11}B NMR (160 MHz, $CDCl_3$) δ 30.07.
^{19}F **NMR** (470 MHz, $CDCl_3$) δ -98.68 (td, J = 10.2, 6.8 Hz, 1F), -105.15 (dt, J = 18.3, 8.5 Hz, 1F).
ESI-HRMS (m/z): calculated for $C_{12}H_{16}BF_2O_2^+$ ($[M+H]^+$) 241.1206, found: 241.1220.
The data matched the literature.[9, 10]

Methyl 4-(4,4,5,5-tetramethyl-1,3,2-dioxaborolan-2-yl)benzoate (6s)

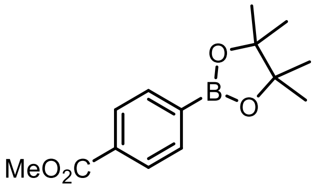

Synthesized according to the general procedure (**P1**) using diazonium salt **5s** (0.4 mmol scale, 100 mg). The crude product was purified by silica gel column chromatography (7% Et_2O, *n*-pentane, R_f: 0.4), affording **6s** (75 mg, 71%) as a white solid.
^{1}H NMR (500 MHz, $CDCl_3$) δ 8.02 (d, *J* = 8.4 Hz, 1H), 7.87 (d, *J* = 8.4 Hz, 1H), 3.92 (s, 2H), 1.35 (s, 8H).
^{13}C NMR (126 MHz, $CDCl_3$) δ 167.3, 134.8, 132.4, 128.7, 84.3, 53.3 – 48.8 (m), 25.0 (q, *J* = 39.3 Hz). The carbon directly attached to the boron atom was not detected due to quadrupolar broadening.
^{11}B NMR (160 MHz, $CDCl_3$) δ 30.90.
ESI-HRMS (m/z): calculated for $C_{14}H_{20}BO_4^+$ ($[M+H]^+$): 263.1449, found: 263.1461.
The data matched the literature.[13]

Ethyl 4-(4,4,5,5-tetramethyl-1,3,2-dioxaborolan-2-yl)benzoate (6t)

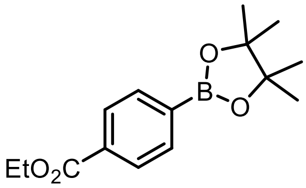

Synthesized according to the general procedure (**P1**) using diazonium salt **5t** (0.4 mmol scale, 105 mg). The crude product was purified by silica gel column chromatography (7% Et_2O, *n*-pentane, R_f: 0.4), affording **6t** (61mg, 55%) as a colorless liquid.
^{1}H NMR (500 MHz, $CDCl_3$): δ 8.02 (d, *J* = 8.3 Hz, 2H), 7.86 (d, *J* = 8.4 Hz, 2H), 4.38 (q, *J* = 7.1 Hz, 2H), 1.40 (t, *J* = 7.1 Hz, 3H), 1.36 (s, 12H).
^{13}C{^{1}H} NMR (125 MHz, $CDCl_3$): δ 166.8, 134.8, 132.8, 128.7, 84.3, 61.2, 25.0, 14.5. The carbon directly attached to the boron atom was not detected due to quadrupolar broadening.
^{11}B NMR (160 MHz, $CDCl_3$): δ 31.03.
ESI-HRMS (m/z): calculated for $C_{15}H_{22}BO_4^+$ ($[M+H]^+$): 277.1606, found: 277.1600.
The data matched the literature.[14]

4,4,5,5-Tetramethyl-2-(4-(trifluoromethyl)phenyl)-1,3,2-dioxaborolane (6u)

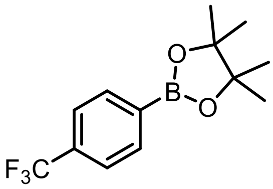

Synthesized according to the general procedure (**P1**) using diazonium salt **5u** (0.4 mmol scale, 104 mg). The crude product was purified by silica gel column chromatography (5% Et_2O, *n*-pentane, R_f: 0.6), affording **6u** (68 mg, 62%) as a colorless oil.
^{1}H NMR (500 MHz, $CDCl_3$) δ 7.91 (d, *J* = 7.5 Hz, 2H), 7.61 (d, *J* = 7.6 Hz, 2H), 1.36 (s, 12H).
^{13}C{^{1}H} NMR (126 MHz, $CDCl_3$) δ 135.1, 132.9 (q, *J* = 31 Hz), 124.2 (q, *J* = 272 Hz), 124.4 (q, *J* = 3.9 Hz), 84.4, 25.0. The carbon directly attached to the boron atom was not detected due to quadrupolar broadening.
^{19}F NMR (470 MHz, $CDCl_3$) δ -63.04.
^{11}B NMR (160 MHz, $CDCl_3$) δ 30.78.
ESI-HRMS (m/z): calculated for $C_{13}H_{17}BF_3O_2^+$ ($[M+H]^+$): 273.1268, found: 273.1254.
The data matched the literature. [6]

4,4,5,5-Tetramethyl-2-(4-nitrophenyl)-1,3,2-dioxaborolane (6v)

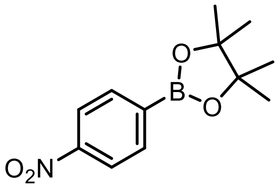

Synthesized according to the general procedure (**P1**) using diazonium salt **5v** (0.4 mmol scale, 94.8 mg). The crude product was purified by silica gel column chromatography (10% Et_2O, *n*-pentane, R_f: 0.49), affording **6v** (55 mg, 55%) as a yellow solid.

^{1}H NMR (500 MHz, $CDCl_3$) δ 8.19 (d, *J* = 8.7 Hz, 2H), 7.96 (d, *J* = 8.7 Hz, 2H), 1.36 (s, 12H).

^{13}C{^{1}H} NMR (126 MHz, $CDCl_3$) δ 150.0, 135.8, 122.6, 84.8, 25.0. The carbon directly attached to the boron atom was not detected due to quadrupolar broadening.

^{11}B NMR (160 MHz, $CDCl_3$) δ 30.49.

ESI-HRMS (m/z): calculated for $C_{12}H_{17}BNO_4^+$ ($[M+H]^+$) 250.1245, found: 250.1260.

The data matched the literature.[8]

2-(4,4,5,5-Tetramethyl-1,3,2-dioxaborolan-2-yl)benzonitrile (6w)

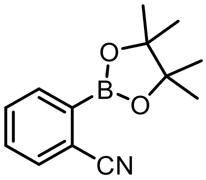

Synthesized according to the general procedure (**P1**) using diazonium salt **5w** (0.4 mmol scale, 86.8 mg). The crude product was purified by silica gel column chromatography (5% Et_2O, *n*-pentane, R_f: 0.6), affording **6w** (50.8 mg, 55%) as a colorless liquid.

^{1}H NMR (500 MHz, $CDCl_3$) δ 7.87 (d, *J* = 7.4 Hz, 1H), 7.69 (d, *J* = 7.6 Hz, 1H), 7.56 (td, *J* = 7.5, 1.4 Hz, 1H), 7.52 (td, *J* = 7.6, 1.6 Hz, 1H), 1.38 (s, 12H).

^{13}C{^{1}H} NMR (126 MHz, $CDCl_3$) δ 136.0, 133.5, 131.7, 131.2, 119.1, 117.4, 84.9, 24.9. The carbon directly attached to the boron atom was not detected due to quadrupolar broadening.

^{11}B NMR (160 MHz, $CDCl_3$) δ 30.33.

ESI-HRMS (m/z): calculated for $C_{13}H_{17}BNO_2^+$ ($[M+H]^+$) 230.1347, found: 230.1337.

The data matched the literature.[9, 11]

4-(4,4,5,5-Tetramethyl-1,3,2-dioxaborolan-2-yl)benzonitrile (6x)

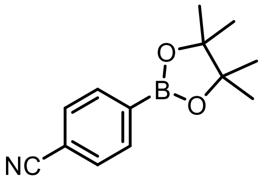

Synthesized according to the general procedure (**P1**) using diazonium salt **5x** (0.4 mmol scale, 86.8 mg). The crude product was purified by silica gel column chromatography (10% Et_2O, *n*-pentane, R_f: 0.45), affording **6x** (49 mg, 53%) as colorless oil.

^{1}H NMR (500 MHz, $CDCl_3$) δ 7.87 (d, *J* = 8.2 Hz, 2H), 7.63 (d, *J* = 8.2 Hz, 2H), 1.34 (s, 12H).

^{13}C{^{1}H} NMR (126 MHz, CDCl3) δ 135.2, 131.2, 119.0, 114.6, 83.6, 25.1 The carbon directly attached to the boron atom was not detected due to quadrupolar broadening.

^{11}B NMR (160 MHz, $CDCl_3$) δ 30.29.

ESI-HRMS (m/z): calculated for $C_{13}H_{20}BN_2O_2^+$ ($[M+NH_4]^+$) 247.1612, found: 247.1619.

The data matched the literature.[8]

1-(4-(4,4,5,5-Tetramethyl-1,3,2-dioxaborolan-2-yl)phenyl)ethan-1-one (6y)

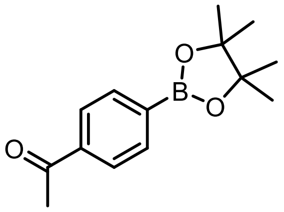

According to the general procedure (**P1**), using diazonium salt 4-acetylbenzenediazonium tetrafluoroborate (0.4 mmol scale, 93.6 mg). The crude product was purified by silica gel column chromatography (10% EtOAc, *n*-hexane, R_f: 0.33), affording **6y** (60.5 mg, 61%) as a colorless oil.
^{1}H NMR (500 MHz, $CDCl_3$) δ 7.93 (d, *J* = 8.3 Hz, 2H), 7.89 (d, *J* = 8.3 Hz, 2H), 2.62 (s, 3H), 1.36 (s, 12H).
^{13}C{^{1}H} NMR (126 MHz, $CDCl_3$) δ 198.7, 139.1, 135.1, 127.4, 84.4, 27.0, 25.0. The carbon directly attached to the boron atom was not detected due to quadrupolar broadening.
^{11}B NMR (160 MHz, $CDCl_3$) δ 30.90.
ESI-HRMS (m/z): calculated for $C_{14}H_{20}BO_3^+$ ($[M+H]^+$) 247.1500, found: 247.1486.
The data matched the literature.[7]

Bis(4-(4,4,5,5-tetramethyl-1,3,2-dioxaborolan-2-yl)phenyl)methanone

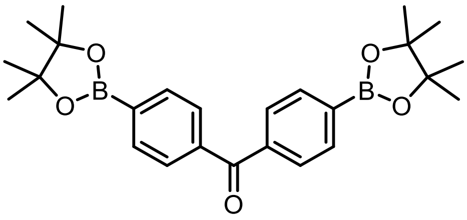

Synthesized according to the general procedure (**P1**) using bis(arenediazonium) salt **5z** (0.4 mmol scale, 82.4 mg). The crude product was purified by silica gel column chromatography (5% Et_2O, *n*-pentane, R_f: 0.45), affording **6z** (101 mg, 58%) as an amorphous white solid.
^{1}H NMR (500 MHz, $CDCl_3$) δ 7.91 (d, *J* = 8.2 Hz, 4H), 7.76 (d, *J* = 8.3 Hz, 4H), 1.37 (s, 24H).
^{13}C{^{1}H} NMR (126 MHz, $CDCl_3$) δ 197.3, 139.7, 134.7, 129.2, 84.4, 25.0. The carbon directly attached to the boron atom was not detected due to quadrupolar broadening.
^{11}B NMR (160 MHz, $CDCl_3$) δ 30.60.
ESI-HRMS (m/z): calculated for $C_{25}H_{33}B_2O_5^+$ ($[M+H]^+$) 435.2509, found: 435.2489.

Procedure for one-pot in-situ diazotization/catalytic borylation

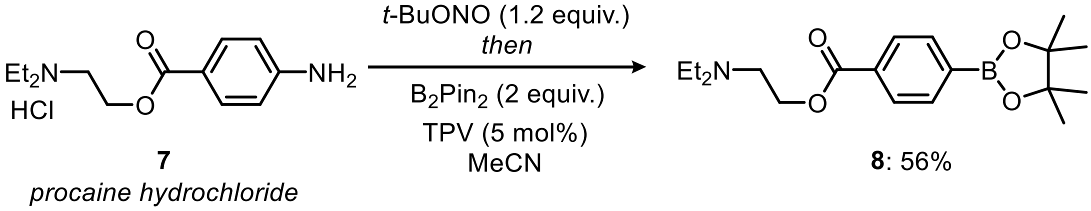

Under Ar atmosphere inside the glovebox, an oven-dried 8 mL vial was charged with a magnetic stir bar, procaine*HCl (0.4 mmol, 109.1 mg, 1 equiv.), dry and degassed MeCN (0.2 M), followed by the slow addition of tert-butyl nitrite. The mixture was stirred for 30 minutes inside the glovebox. Bis(pinacolato)diboron (0.8 mmol, 203.2 mg, 2 equiv.) followed by TPV (0.02 mmol, 6.3 mg, 0.05 equiv.) were added, and the vial was sealed with a septum cap and parafilm. The reaction mixture was stirred at 25 °C for 24 h. The reaction mixture was evaporated using a rotary evaporator and the residue was purified by flash chromatography (25% EtOAc, 5% TEA, n-hexane, R_f: 0.25), affording **6y** (77 mg, 56%) as a white solid.

^{1}H NMR (500 MHz, $CDCl_3$) δ 8.00 (d, *J* = 8.2 Hz, 2H), 7.87 (d, *J* = 8.2 Hz, 2H), 4.51 (t, *J* = 6.3 Hz, 2H), 3.04 – 2.95 (m, 2H), 2.84 – 2.72 (m, 4H), 1.35 (s, 12H), 1.15 (t, *J* = 7.2 Hz, 6H).
^{13}C{^{1}H} NMR (126 MHz, $CDCl_3$) δ 166.6, 134.8, 132.2, 128.8, 84.3, 50.8, 47.8, 25.2, 24.9, 11.5.
^{11}B NMR (160 MHz, $CDCl_3$) δ 31.13.
ESI-HRMS (m/z): calculated for $C_{19}H_{31}BNO_4^+$ $[M+H]^+$ 348.2341, found: 348.2372.

Procedure for one-pot two-step synthesis of Ar-BF_3K salt

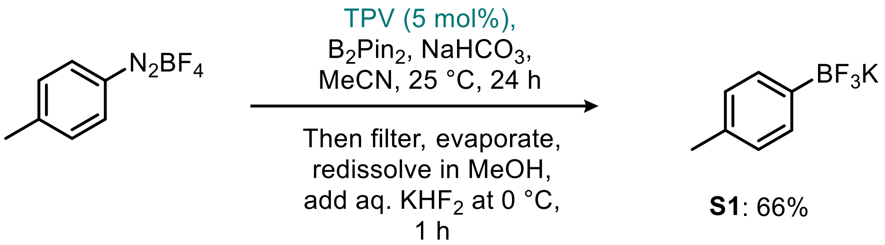

Under Ar atmosphere inside the glovebox, an oven-dried 8 mL vial was charged with a magnetic stir bar, sodium bicarbonate (0.8 mmol, 67.2 mg, 2.0 equiv.), bis(pinacolato)diboron (0.8 mmol, 203.1 mg, 2.0 equiv.), dry and degassed MeCN (0.2 M), and the arenediazonium salt **5d** (82.4 mg, 0.4 mmol, 1.0 equiv.), followed by the addition of TPV (0.02 mmol, 6.2 mg, 0.05 equiv.). The vial was sealed with a septum cap and parafilm. The reaction was taken out of the glovebox and stirred at 25 °C for 24 h; the solids were filtered off using a short silica plug, and the solvent was evaporated. The crude mixture was redissolved in MeOH, cooled to 0 °C, and a solution of KHF_2 (125.0 mg, 1.6 mmol, 4.0 equiv.) in water (0.36 mL, 4.5 M) was added dropwise to form a precipitate. The mixture was stirred for 30 minutes, allowing it to warm to 25 °C. The product was filtered, washed with cold water and ether, dried under high vacuum overnight to give **S1** as a white solid (52.8 mg, 66% yield).
^{1}H NMR (500 MHz, acetone-d_6) δ 7.36 (d, *J* = 7.4 Hz, 2H), 6.91 (d, *J* = 7.3 Hz, 2H), 2.21 (s, 3H).
^{13}C{^{1}H} NMR (126 MHz, acetone-d_6) δ 134.3, 132.5 (q, *J* = 1.9 Hz), 127.7, 21.3. The carbon directly attached to the boron atom was not detected due to quadrupolar broadening.
^{11}B NMR (160 MHz, acetone-d_6) δ 3.70 (q, *J* = 55.7 Hz). ^{19}F NMR (470 MHz, acetone-d_6) δ -142.2 (q, *J* = 44.6 Hz).
ESI-HRMS (m/z): calculated for $C_7H_7BF_3^-$ $([M-K]^-)$ 159.0598, found: 159.0605.
The data matched the literature.[9, 15]

Procedure for one-pot borylation/Suzuki-Miyaura coupling

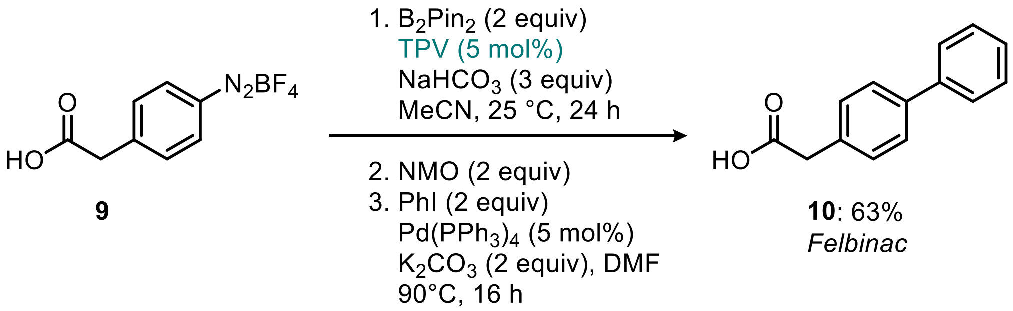

In a Schlenk tube under an Ar atmosphere inside the glovebox, an oven-dried 8 mL vial was charged with a magnetic stir bar, sodium bicarbonate (1.5 mmol, 126 mg, 3 equiv.), bis(pinacolato)diboron (1 mmol, 253.9 mg, 2 equiv.), dry and degassed MeCN (0.2 M), and the arenediazonium salt **9** (0.5 mmol, 125 mg, 1 equiv.), followed by the addition of TPV (0.025 mmol, 7.8 mg, 0.05 equiv.). The reaction mixture was taken outside

the glovebox and stirred at 25 °C for 24 h. After that, 4-methylmorpholine 4-oxide (NMO, one mmol, 117 mg, two equiv.) was added to the reaction mixture, and the mixture was stirred for an additional 30 min at room temperature. The solvent was removed using a rotary evaporator, and the crude reaction mixture was redissolved in dry and degassed DMF (0.25 M). Phenyl iodide (1 mmol, 204 mg, 2 equiv.), K_2CO_3 (1 mmol, 138 mg, 2 equiv.), $Pd(PPh_3)_4$ (0.025 mmol, 29 mg, 0.05 equiv.) were added and the reaction mixture was stirred at 90 °C for 16 h. The reaction mixture was extracted with 1 M HCl and ether (3x5 mL) and the combined organic layers were dried over an anhydrous sodium sulphate and concentrated. The residue was purified by flash chromatography (40% EtOAc, *n*-hexane) to afford **10** as a white solid (70 mg, 63% yield).
^{1}H NMR (500 MHz, DMSO-*d*$_6$) δ 12.35 (s, 1H), 7.65 (dd, *J* = 8.3, 1.3 Hz, 2H), 7.61 (d, *J* = 8.2 Hz, 2H), 7.46 (t, *J* = 7.7 Hz, 2H), 7.38 – 7.33 (m, 3H), 3.61 (s, 2H).
^{13}C{^{1}H} NMR (126 MHz, DMSO-*d*$_6$) δ 172.7, 140.0, 138.5, 134.3, 130.0, 129.4, 128.9, 128.2, 127.3, 126.6, 126.5, 24.7.
ESI-HRMS (m/z): calculated for $C_{14}H_{13}O_2^+$ ($[M+H]^+$) 213.0910, found: 213.0912.
The data matched the literature.[16]

Procedure for one-pot borylation/Petasis reaction

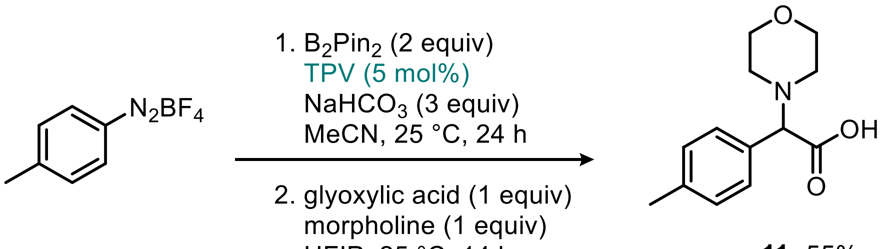

11: 55%

Under Ar atmosphere inside glovebox, an oven-dried 8 mL vial was charged with a magnetic stir bar, sodium bicarbonate (1 mmol, 84 mg, 2 equiv.), bis(pinacolato)diboron (1 mmol, 253.9 mg, 2 equiv.) dry and degassed MeCN (0.2 M), and the arenediazonium salt **5d** (0.5 mmol, 103 mg, 1 equiv.), followed by the addition of TPV (0.025 mmol, 7.8 mg, 0.05 equiv.). The vial was sealed with a septum cap and parafilm. The reaction mixture was taken outside the glovebox, and stirred at 25 °C for 24 h, the solvent was removed using rotary evaporator. After that, HFIP (1 M), glyoxylic acid monohydrate (0.5 mmol, 46 mg, 1 equiv.), morpholine (0.5 mmol, 43.1 µL, 1 equiv.) were added to the same reaction vial, and stirred for 14 h at rt. Upon completion, the solvent was removed under reduced pressure, and the crude material was triturated with Et_2O (10 mL). The resulting precipitate was collected by filtration and dissolved in aqueous HCl (1 M). The aqueous phase was washed with EtOAc (3×10 mL), then concentrated under reduced pressure to afford the product 11 as an off-white solid (141.5 mg, 55% yield).
^{1}H NMR (500 MHz, CD_3OD) δ 7.50 (d, *J* = 8.2 Hz, 2H), 7.38 (d, *J* = 8.2 Hz, 2H), 5.10 (s, 1H), 3.93 (s, 4H), 3.27 (d, *J* = 4.9 Hz, 5H), 2.43 (s, 3H).
^{13}C{^{1}H} NMR (126 MHz, CD_3OD) δ 172.3, 142.7, 131.4, 130.7, 126.9, 94.5, 72.9, 64.8, 44.6, 21.2.
ESI-HRMS (m/z): calculated for $C_{13}H_{18}NO_3^+$ ($[M+H]^+$) 236.1281, found: 236.1265.

Gram-scale synthesis of 6b using 2 mol% TPV

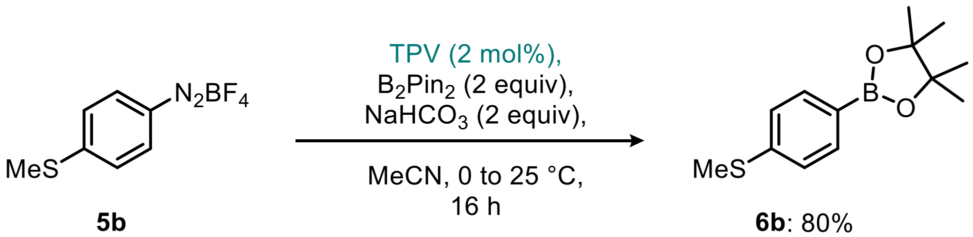

5b **6b**: 80%

To an oven-dried 100 mL Schlenk flask, B_2Pin_2 (3.20 g, 12.6 mmol, 2.00 equiv), $NaHCO_3$ (1.06 g, 12.6 mmol, 2.00 equiv), and TPV (40.0 mg, 0.126 mmol, 0.02 equiv) were added, evacuated, and back-filled with N_2 three times. Dry, degassed MeCN (31.5 mL, 0.2 M) was added, and the flask was cooled to 0 °C in an ice bath to minimize potential exotherm with a gram-scale arenediazonium reaction. Under vigorous stirring, the arenediazonium salt **5b** (1.50 g, 6.30 mmol, 1.00 equiv.) was added in portions under a weak flow of N_2. The ice bath was removed, and the flask was stirred at 25 °C for 16 h, during which the NMR aliquot (using DMSO-d_6) confirmed full conversion of the arenediazonium salt. The solvent was removed under a rotary evaporator, and the residue was purified by flash chromatography (10% Et_2O in *n*-pentane), affording **6b** (1.26 g, 80%) as a yellow oil.

Borylation with B_2Epin_2

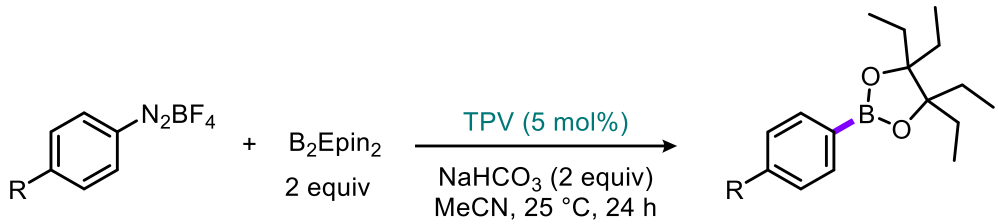

12 (R=H): 60%
13 (R=Me): 90%

4,4,5,5-Tetraethyl-2-phenyl-1,3,2-dioxaborolane (12)

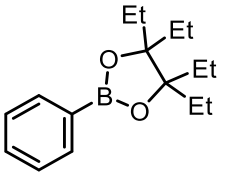

According to the general procedure (**P1**), using diazonium salt **5l** (0.4 mmol scale, 76.8 mg) and B_2Epin_2 (292.9 mg, 0.8 mmol, 2 equiv). The crude product was purified by silica gel column chromatography (2% Et_2O, *n*-pentane, R_f: 0.42), affording **10** (62 mg, 60%).

^{1}H NMR (500 MHz, $CDCl_3$) δ 7.83 (d, *J* = 6.6 Hz, 2H), 7.45 (t, *J* = 7.4 Hz, 1H), 7.36 (t, *J* = 7.3 Hz, 3H), 1.84 – 1.67 (m, 8H), 0.97 (t, *J* = 7.5 Hz, 12H).

^{13}C{^{1}H} NMR (126 MHz, $CDCl_3$) δ 134.8, 131.1, 127.6, 88.7, 26.4, 8.8. The carbon directly attached to the boron atom was not detected due to quadrupolar broadening.

^{11}B NMR (160 MHz, $CDCl_3$) δ 30.62.

ESI-HRMS (m/z): calculated for $C_{16}H_{26}BO_2^+$ ($[M+H]^+$) 261.2020, found: 261.2028.

4,4,5,5-Tetraethyl-2-(p-tolyl)-1,3,2-dioxaborolane (13)

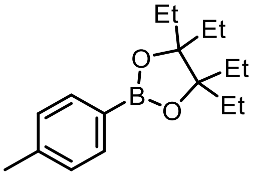

Synthesized according to the general procedure (**P1**) using diazonium salt **5d** (0.4 mmol scale, 82.4 mg) and $B_2(Epin)_2$ (292.9 mg, 0.8 mmol, 2 equiv). The crude product was purified by silica gel column chromatography (3% Et_2O, *n*-pentane, R_f: 0.60), affording **11** (99 mg, 90%) as a colorless oil.

^{1}H NMR (500 MHz, $CDCl_3$) δ 7.73 (d, *J* = 7.6 Hz, 2H), 7.19 (d, *J* = 7.6 Hz, 2H), 2.37 (s, 3H), 1.85 - 1.65 (m, 8H), 0.98 (t, *J* = 7.5 Hz, 12H).

$^{13}C\{^1H\}$ NMR (126 MHz, $CDCl_3$) δ 141.3, 135.0, 128.6, 88.7, 26.6, 21.9, 9.0. The carbon directly attached to the boron atom was not detected due to quadrupolar broadening.
^{11}B NMR (160 MHz, $CDCl_3$) δ 30.62.
ESI-HRMS (m/z): calculated for $C_{17}H_{31}BNO_2^+$ ($[M+NH_4]^+$) 292.2442, found: 292.2418.
The data matched the literature.[17]

Copies of NMR Spectra

^{1}H NMR (500 MHz, DMSO-d_6)

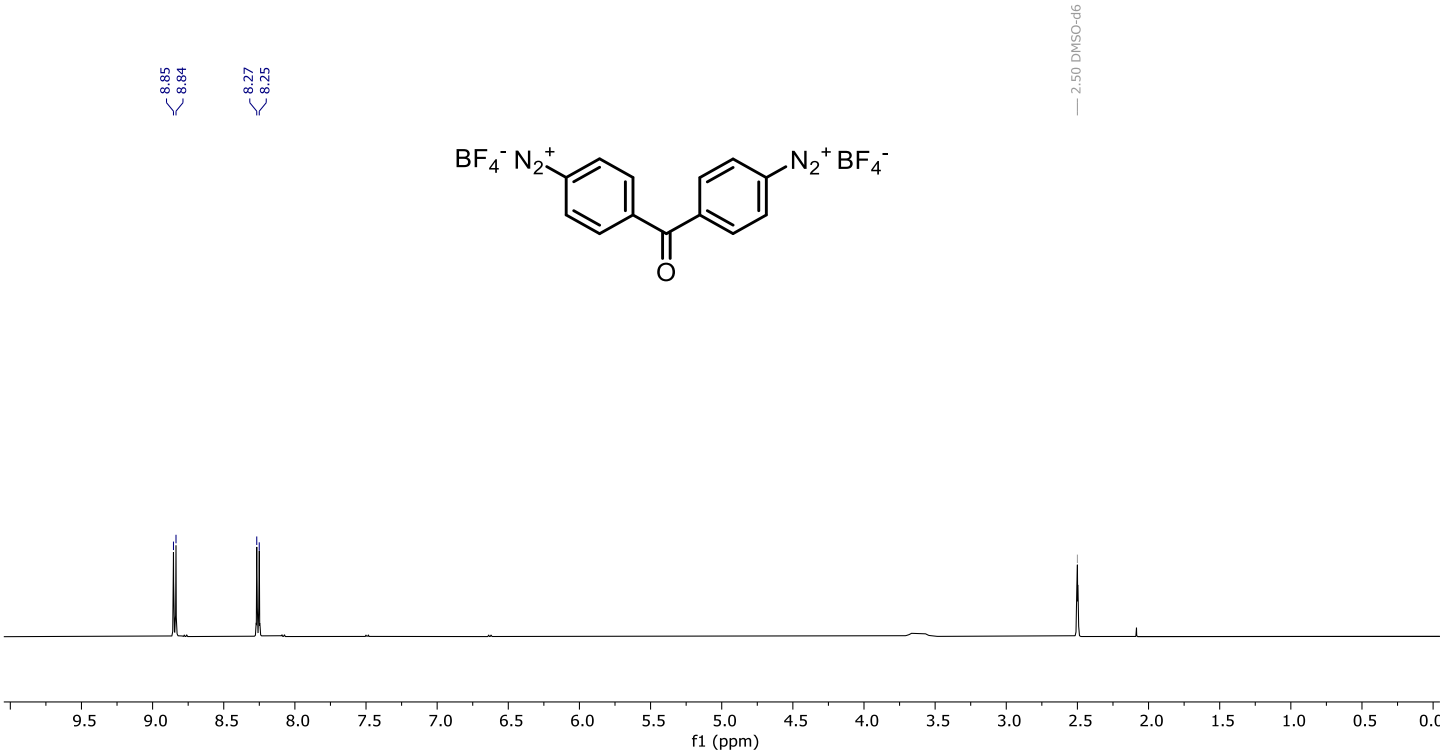

^{13}C{^{1}H} NMR (126 MHz, DMSO-d_6)

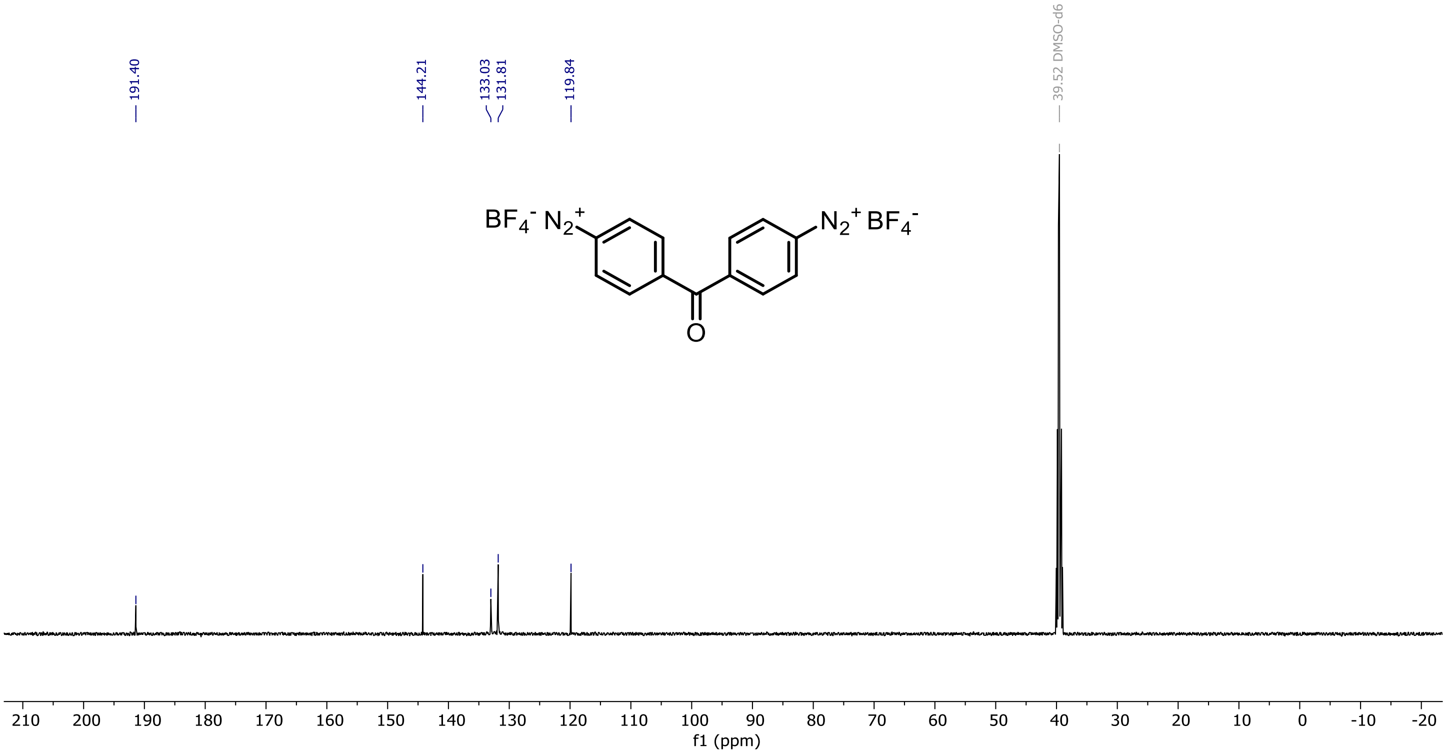

^{11}**B NMR** (160 MHz, DMSO-d_6)

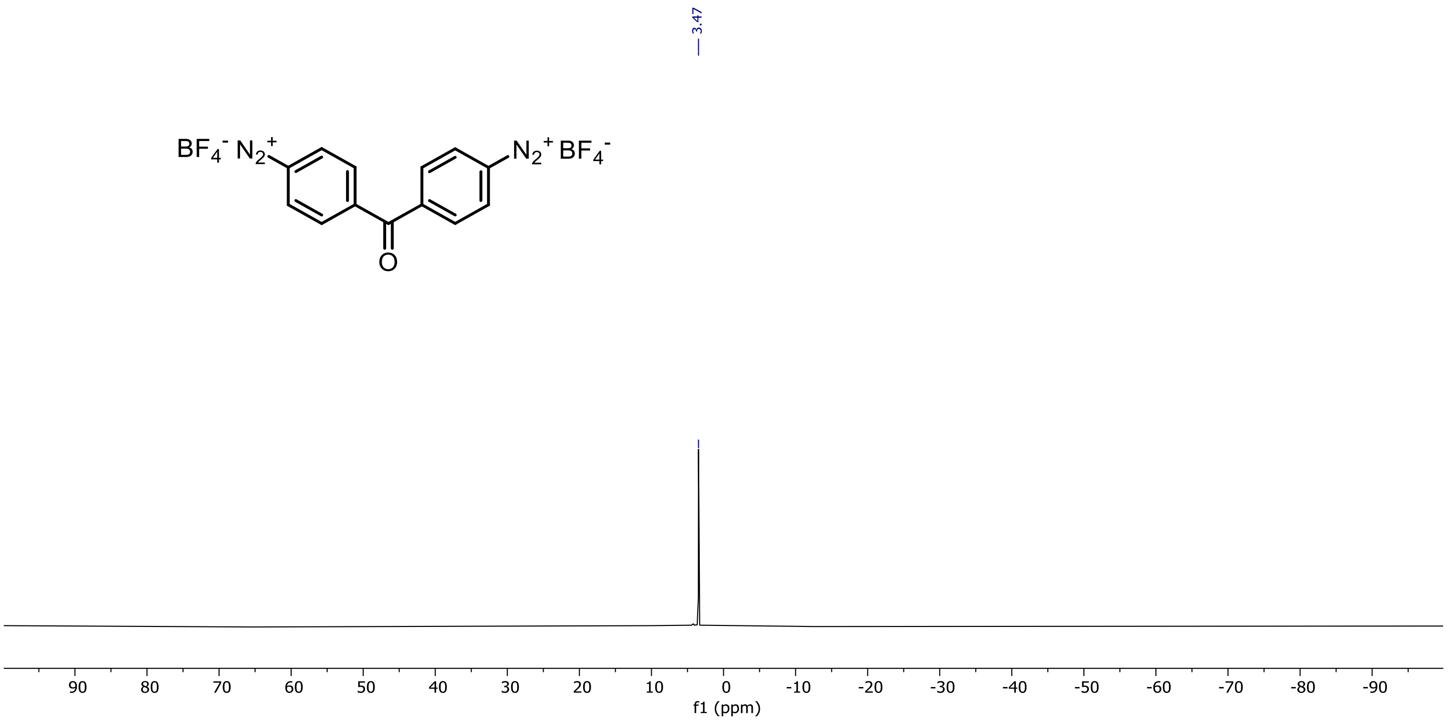

^{19}**F NMR** (470 MHz, DMSO-d_6)

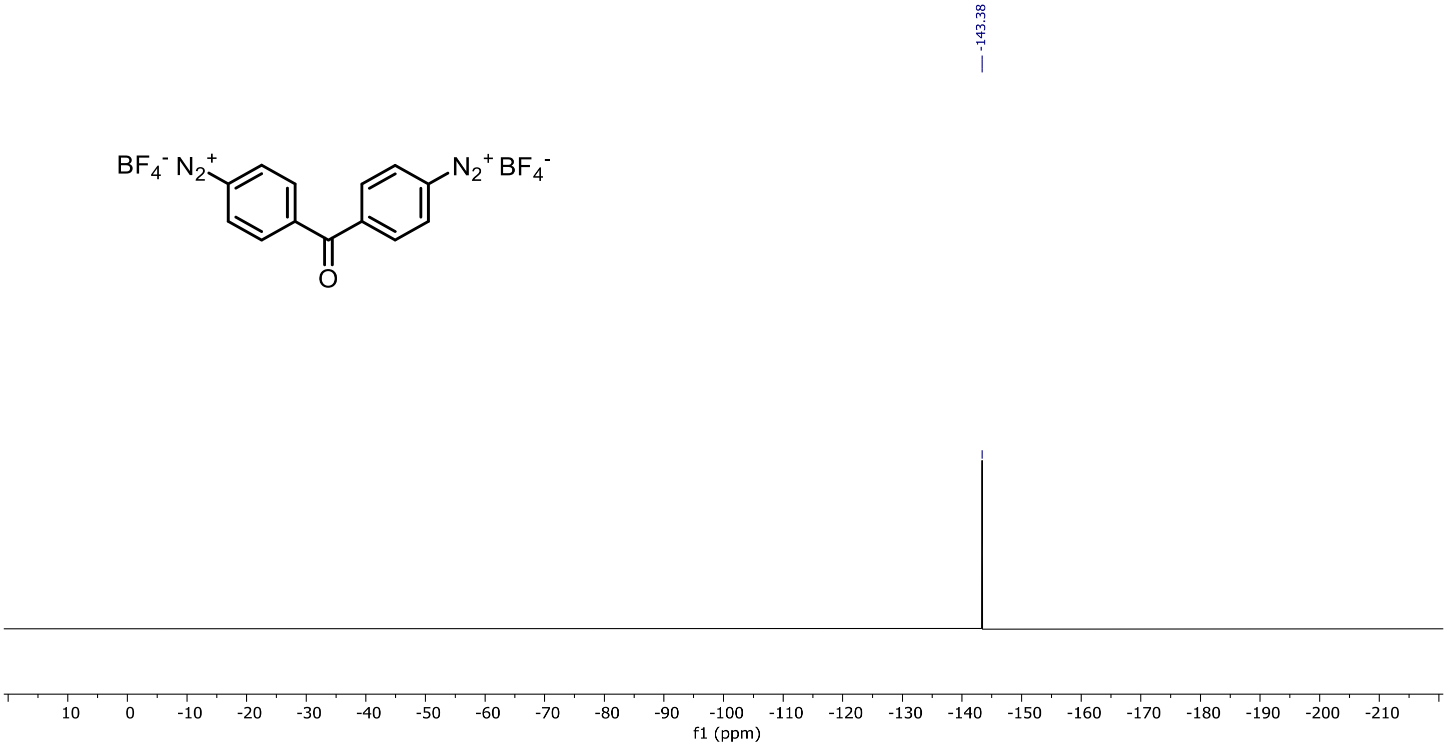

1,3-bis(4,4,5,5-tetramethyl-1,3,2-dioxaborolan-2-yl)benzene

^{1}H NMR (500 MHz, $CDCl_3$)

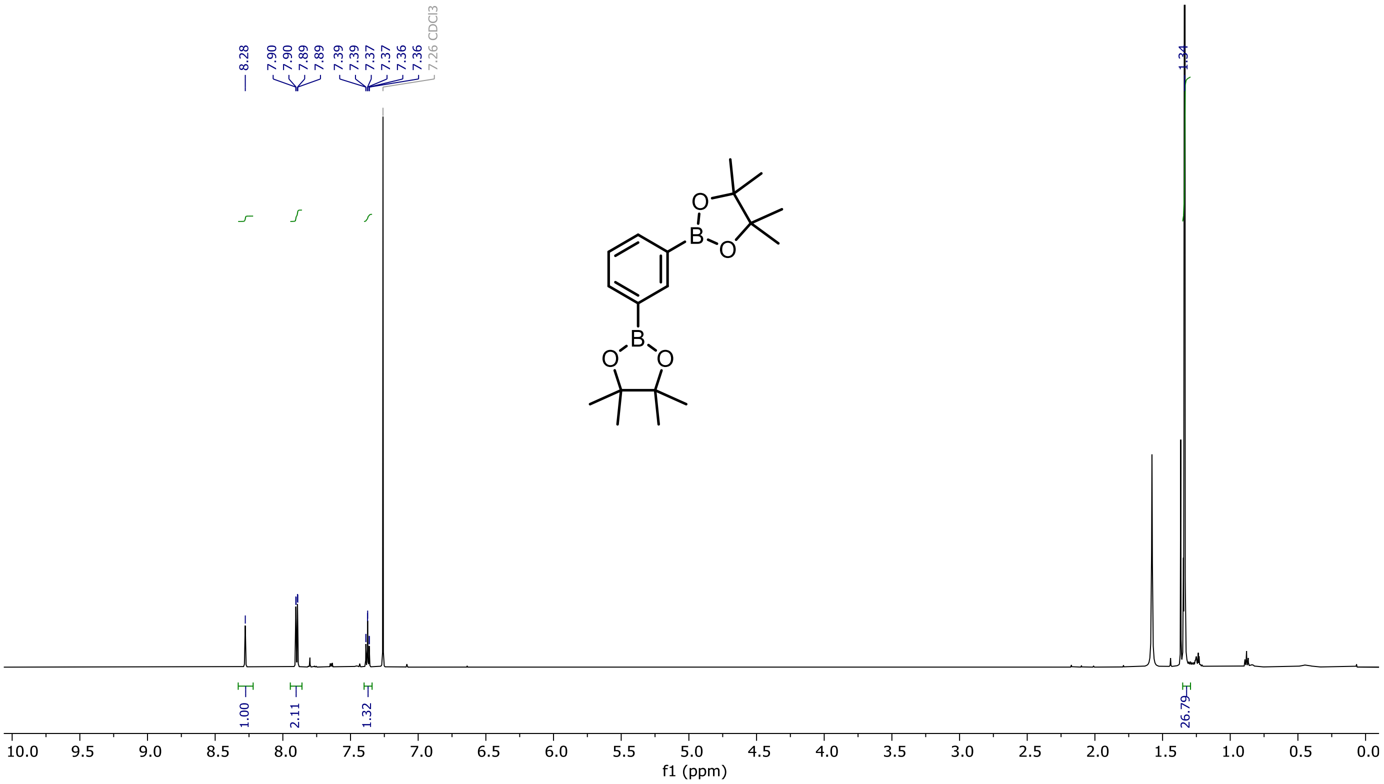

$^{13}C\{^{1}H\}$ NMR (126 MHz, $CDCl_3$)

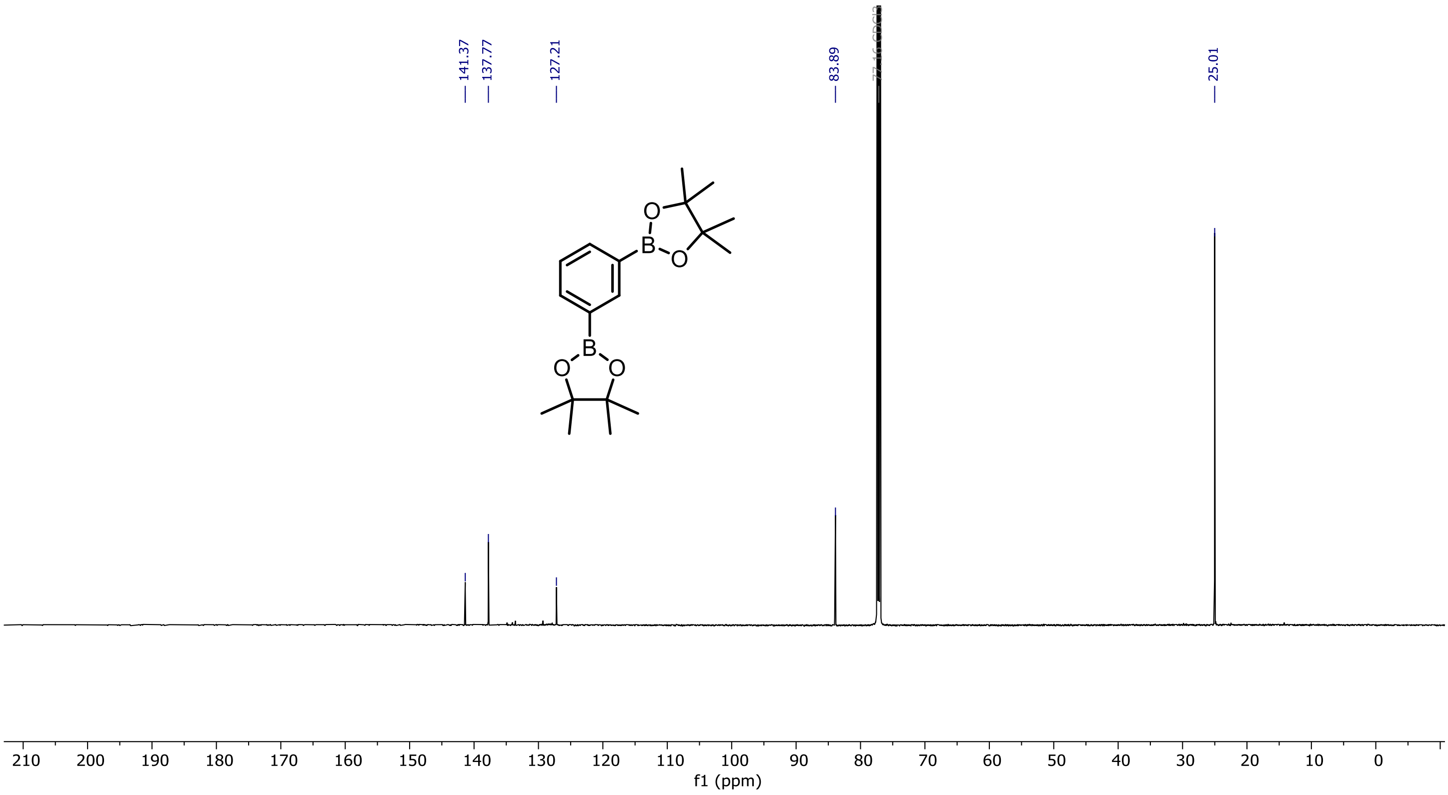

^{11}B NMR (160 MHz, $CDCl_3$)

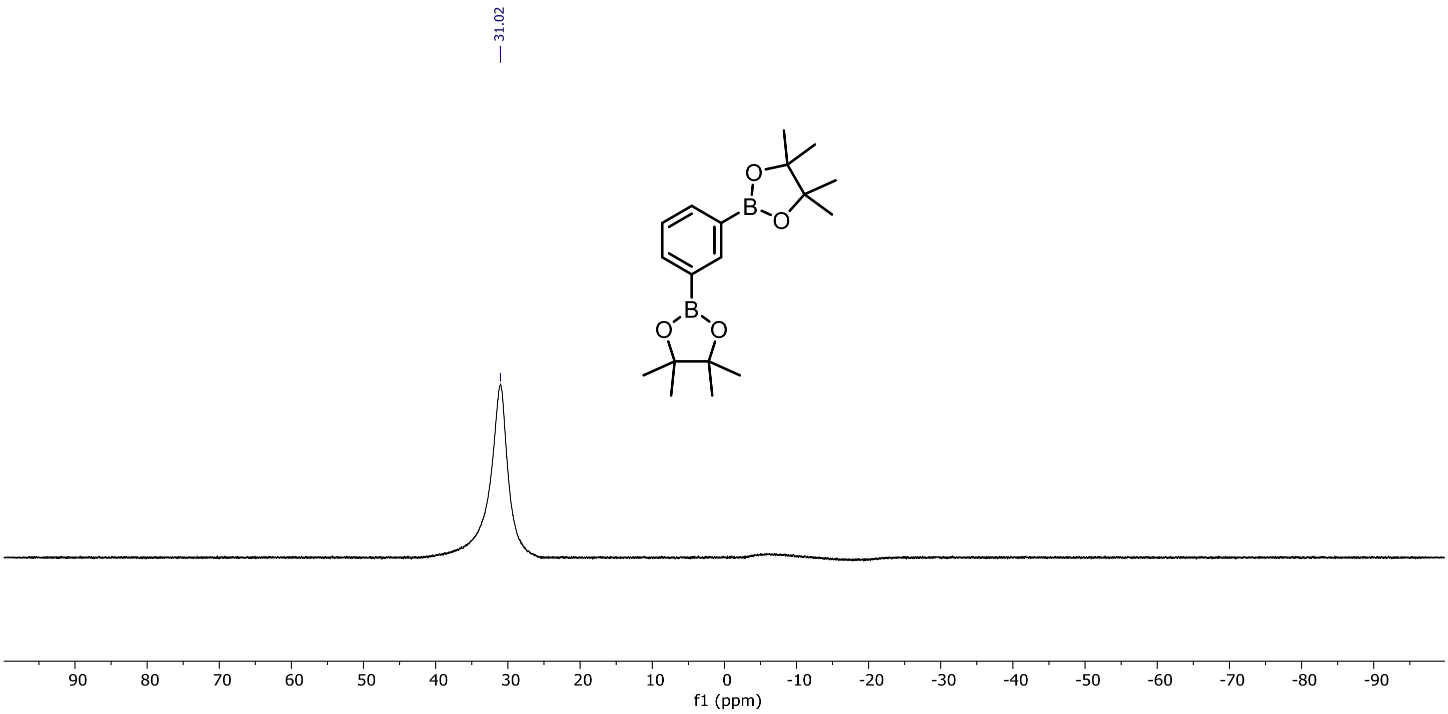

2-(4-Methoxyphenyl)-4,4,5,5-tetramethyl-1,3,2-dioxaborolane (6a)

^{1}H NMR (500 MHz, $CDCl_3$)

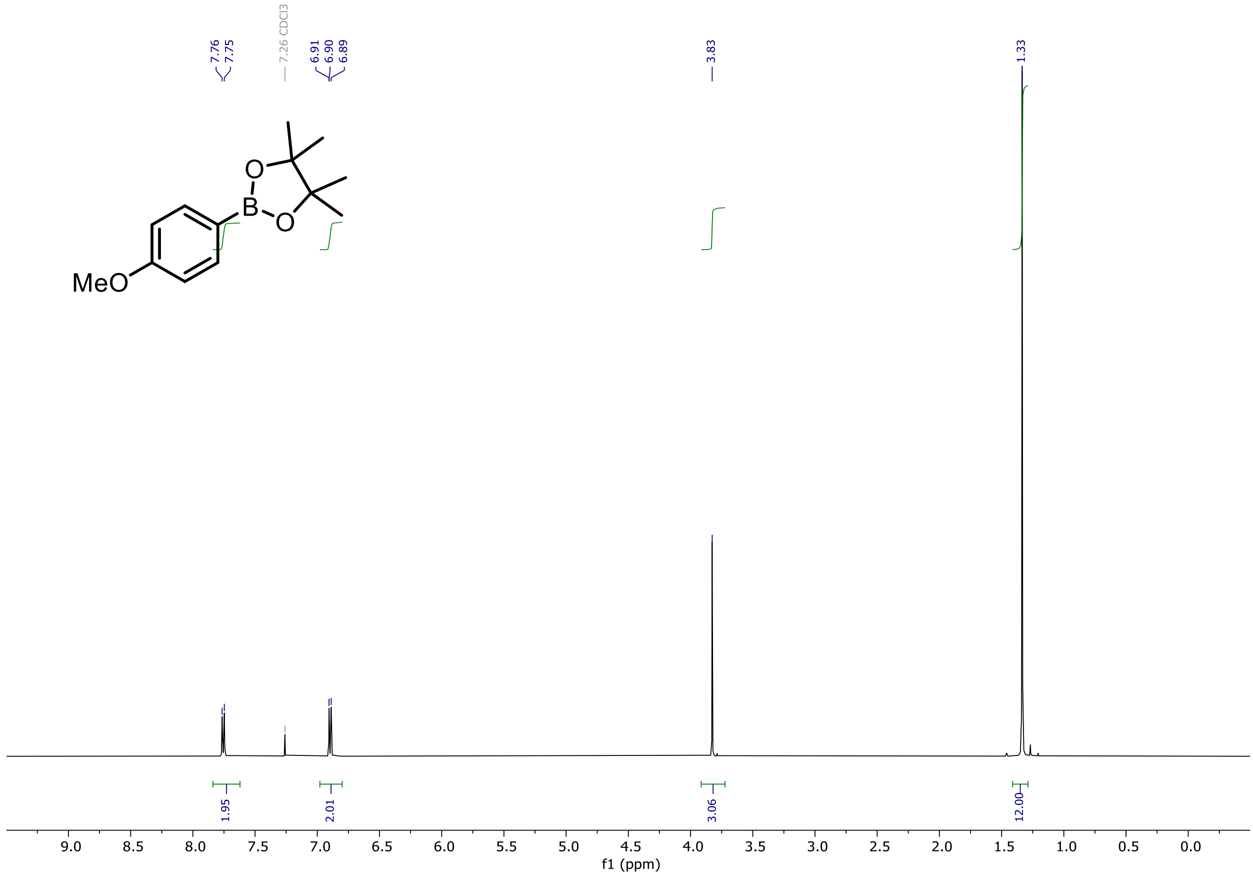

$^{13}C\{^{1}H\}$ NMR (126 MHz, $CDCl_3$)

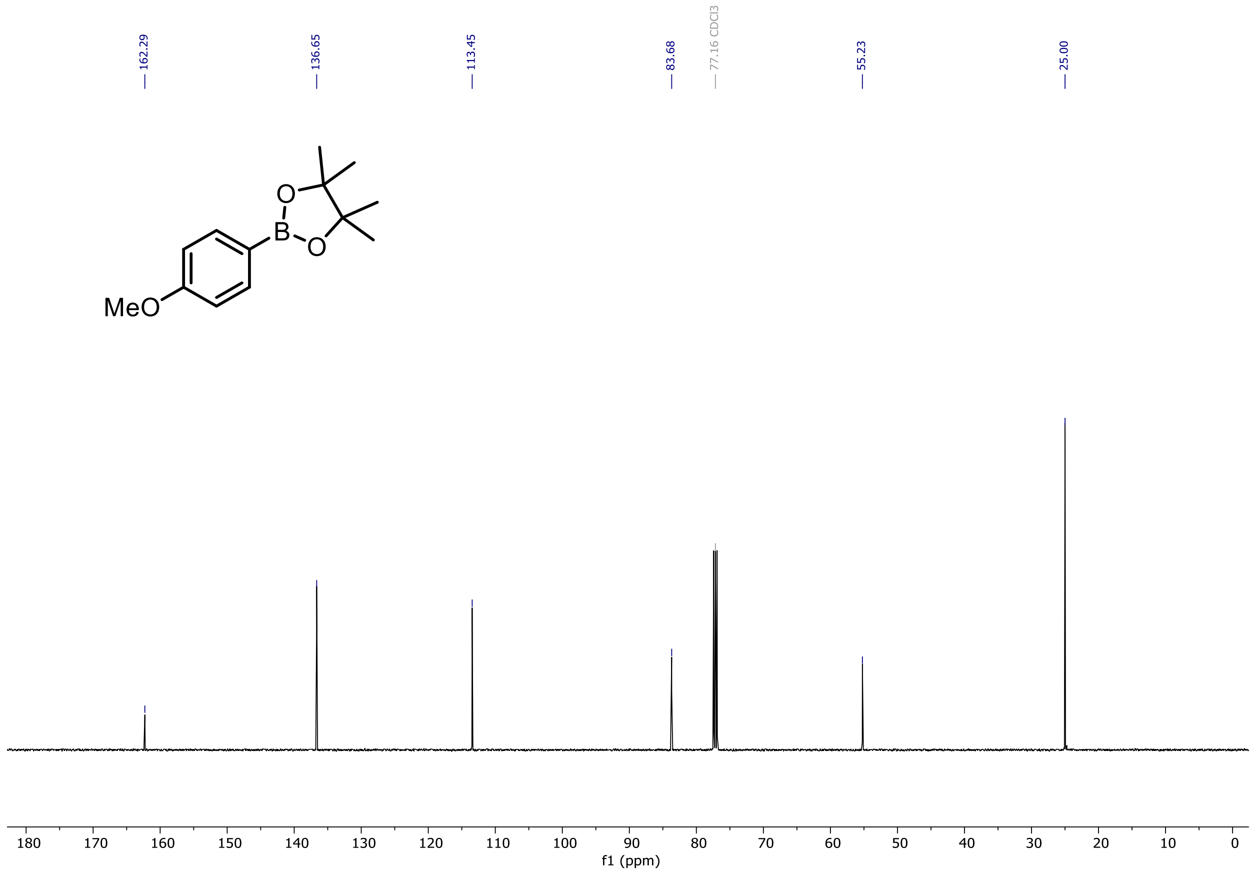

^{11}B NMR (160 MHz, $CDCl_3$)

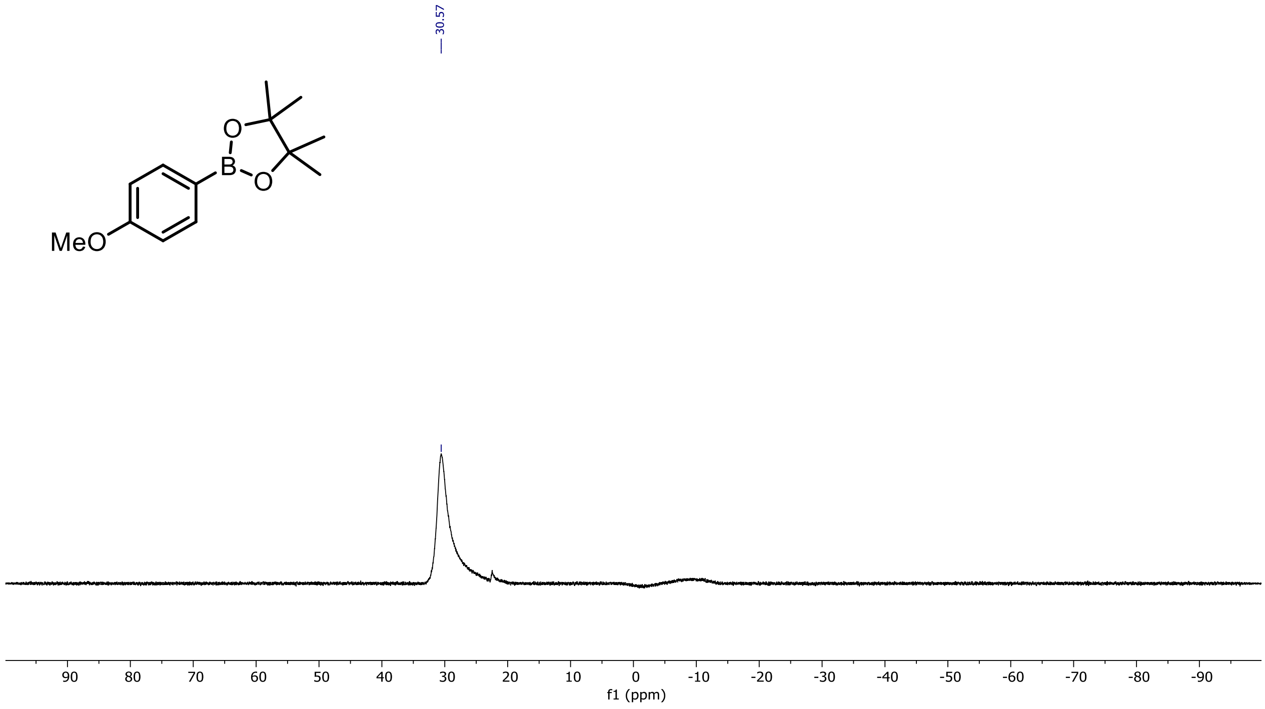

4,4,5,5-Tetramethyl-2-(4-(methylthio)phenyl)-1,3,2-dioxaborolane (6b)

^{1}H NMR (500 MHz, $CDCl_3$)

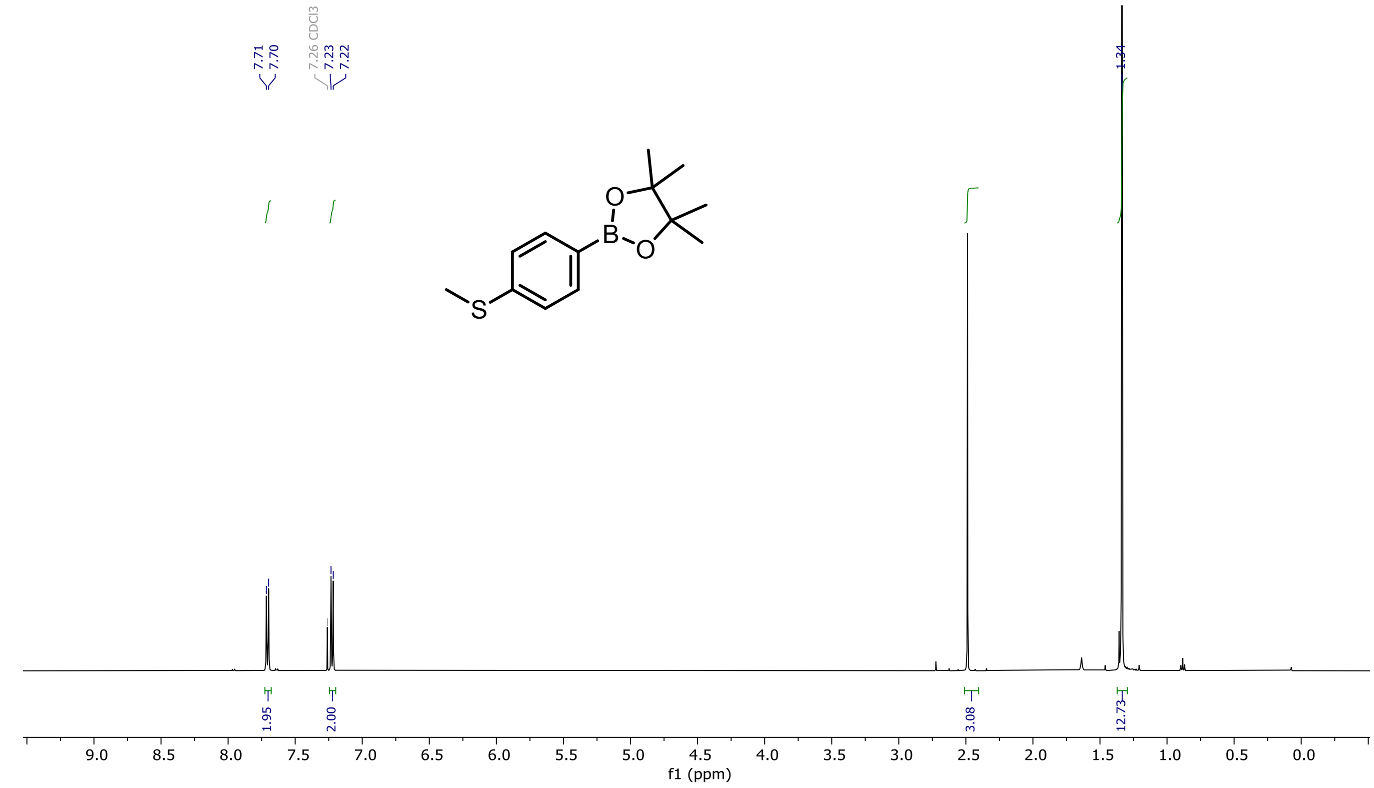

$^{13}C\{^{1}H\}$ NMR (126 MHz, $CDCl_3$)

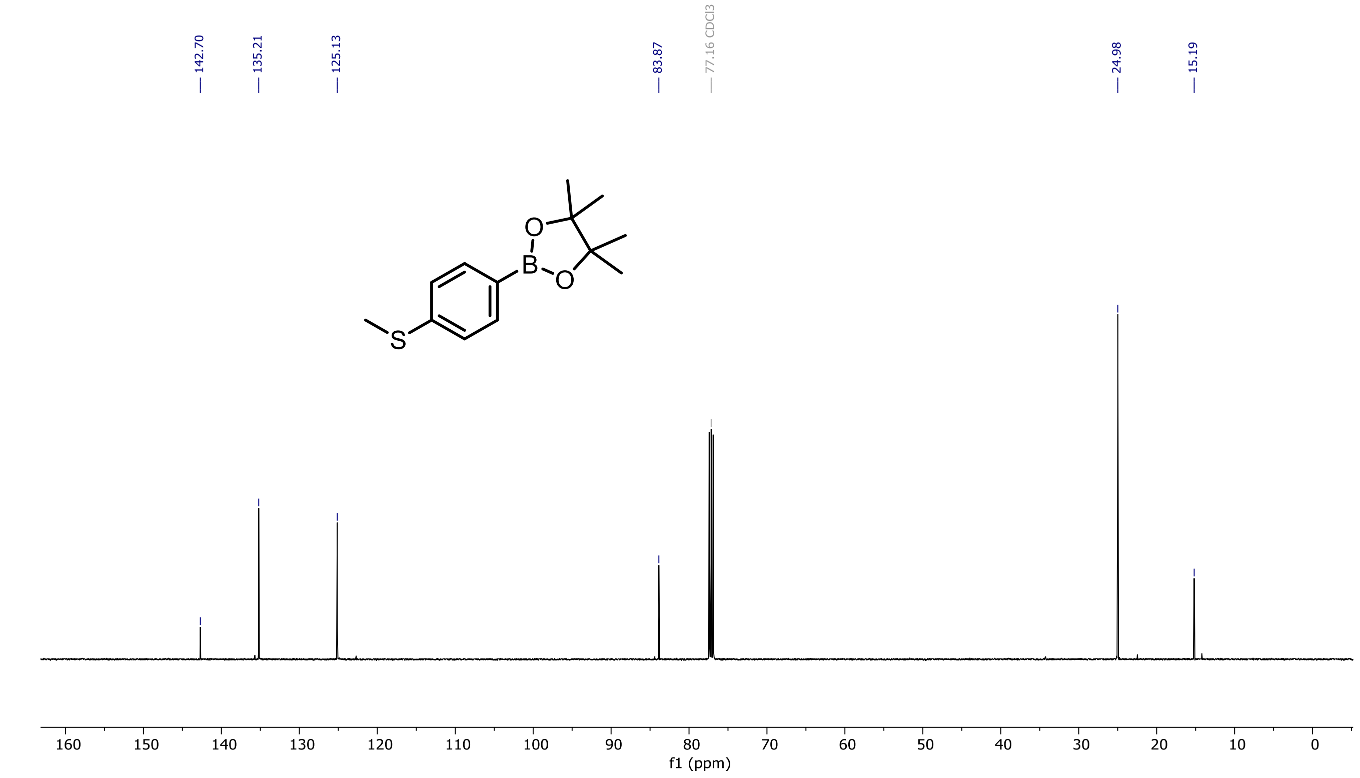

^{11}B NMR (160 MHz, $CDCl_3$)

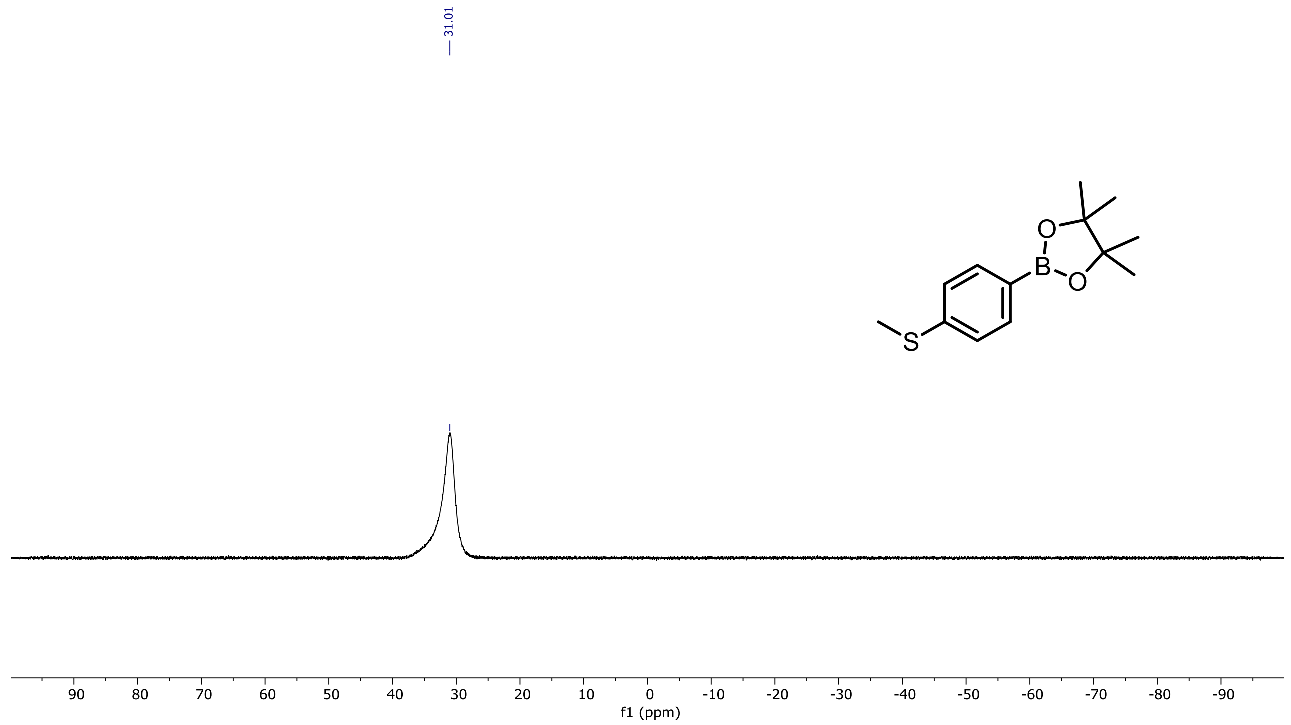

N-(4-(4,4,5,5-Tetramethyl-1,3,2-dioxaborolan-2-yl)phenyl)acetamide (6c)

^{1}H NMR (500 MHz, $CDCl_3$)

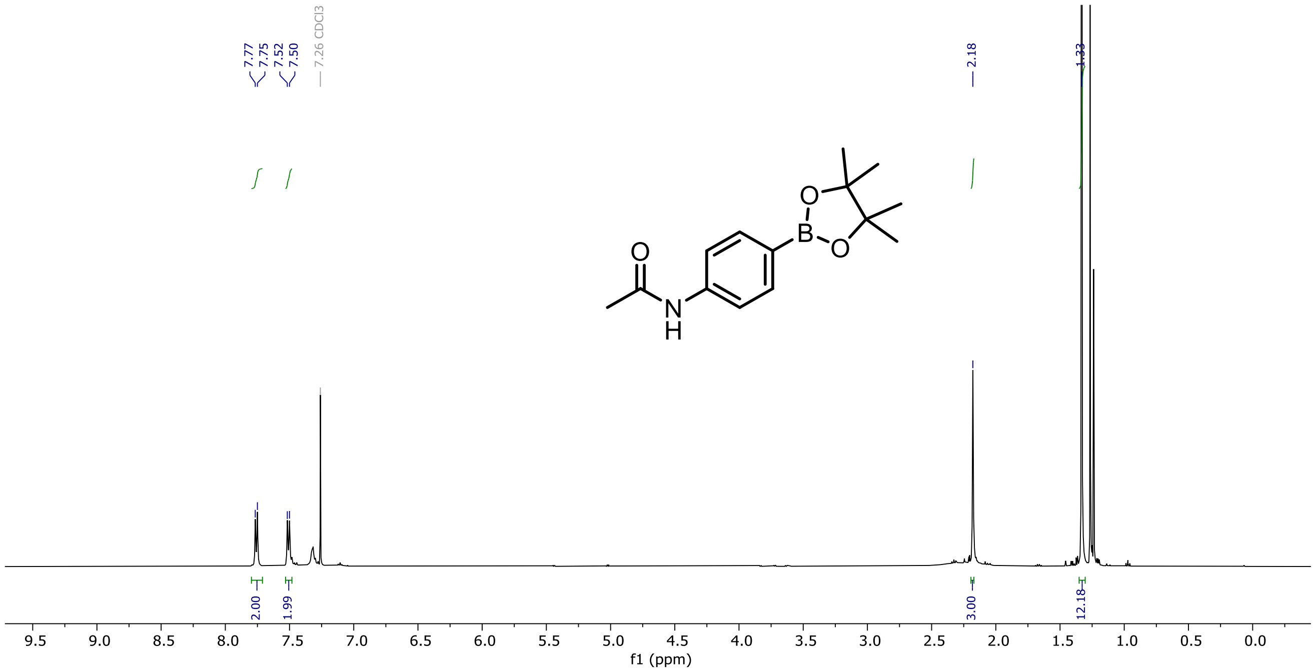

$^{13}C\{^{1}H\}$ NMR (126 MHz, $CDCl_3$)

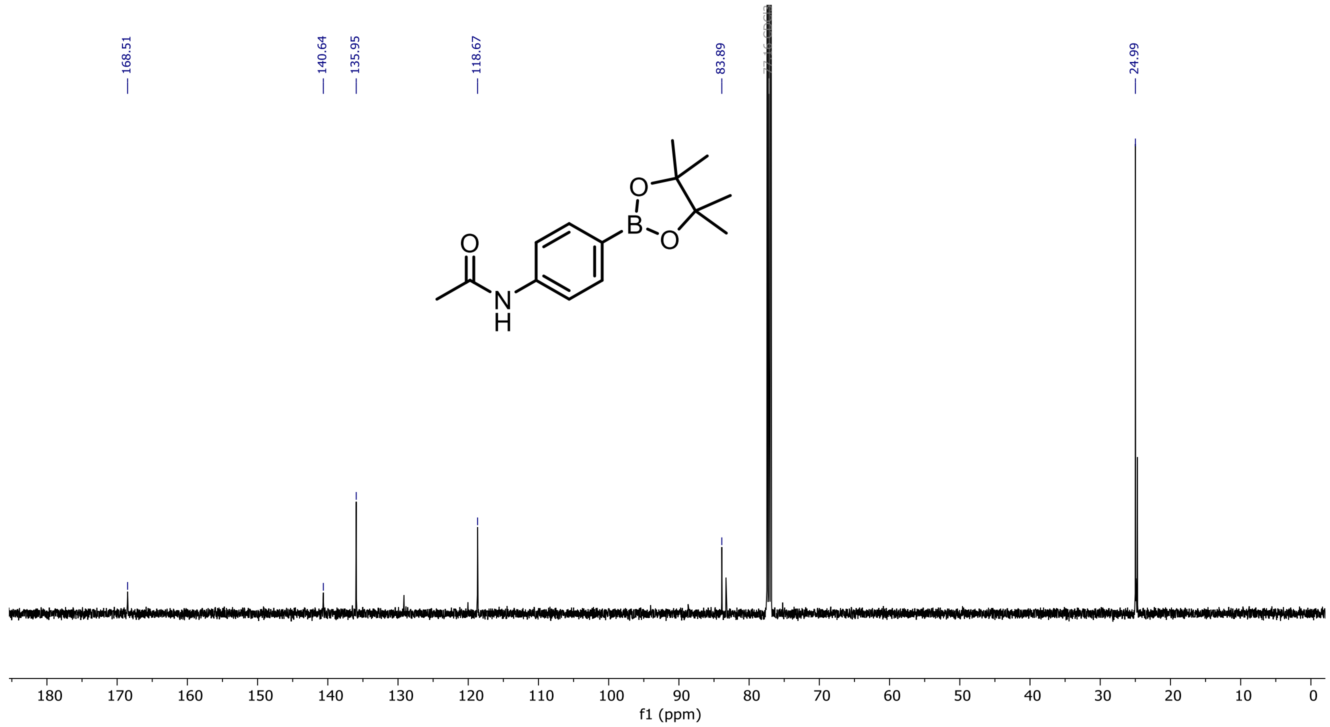

^{11}B NMR (160 MHz, $CDCl_3$)

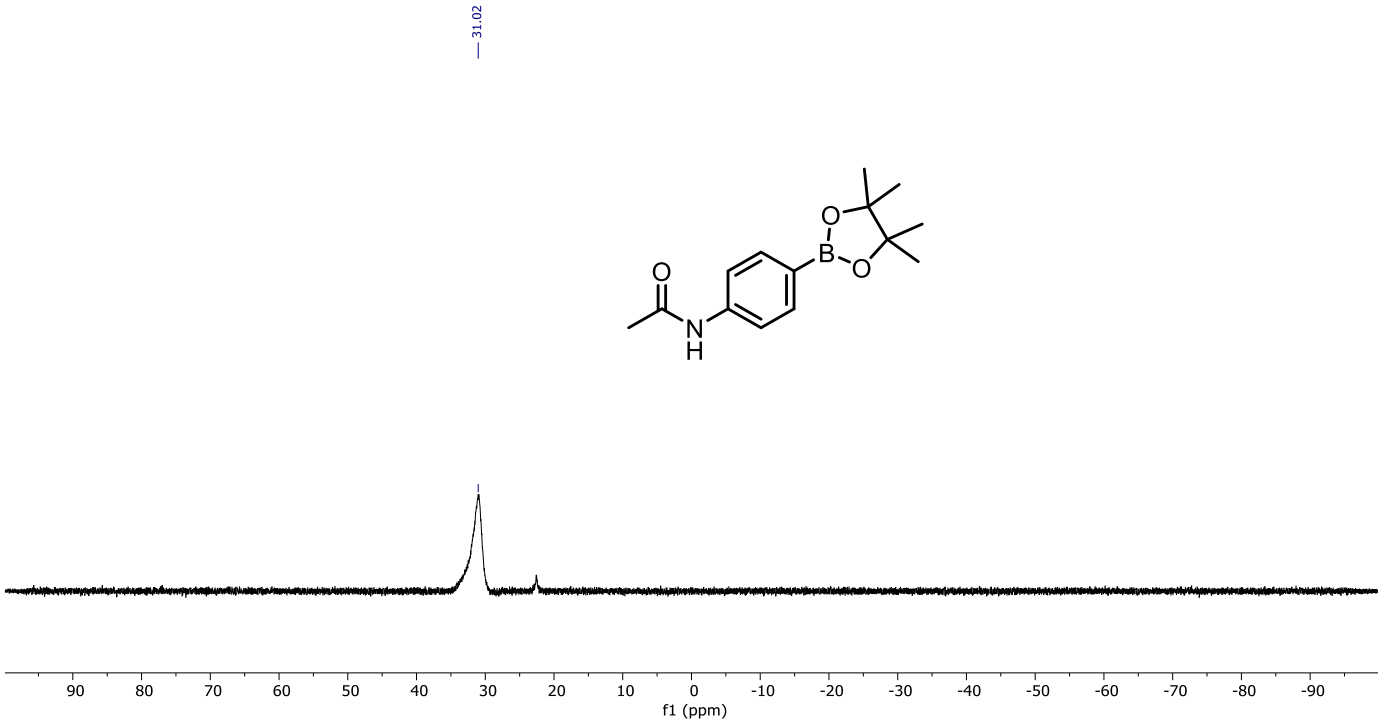

4,4,5,5-Tetramethyl-2-(p-tolyl)-1,3,2-dioxaborolane (6d)

^{1}H NMR (500 MHz, $CDCl_3$)

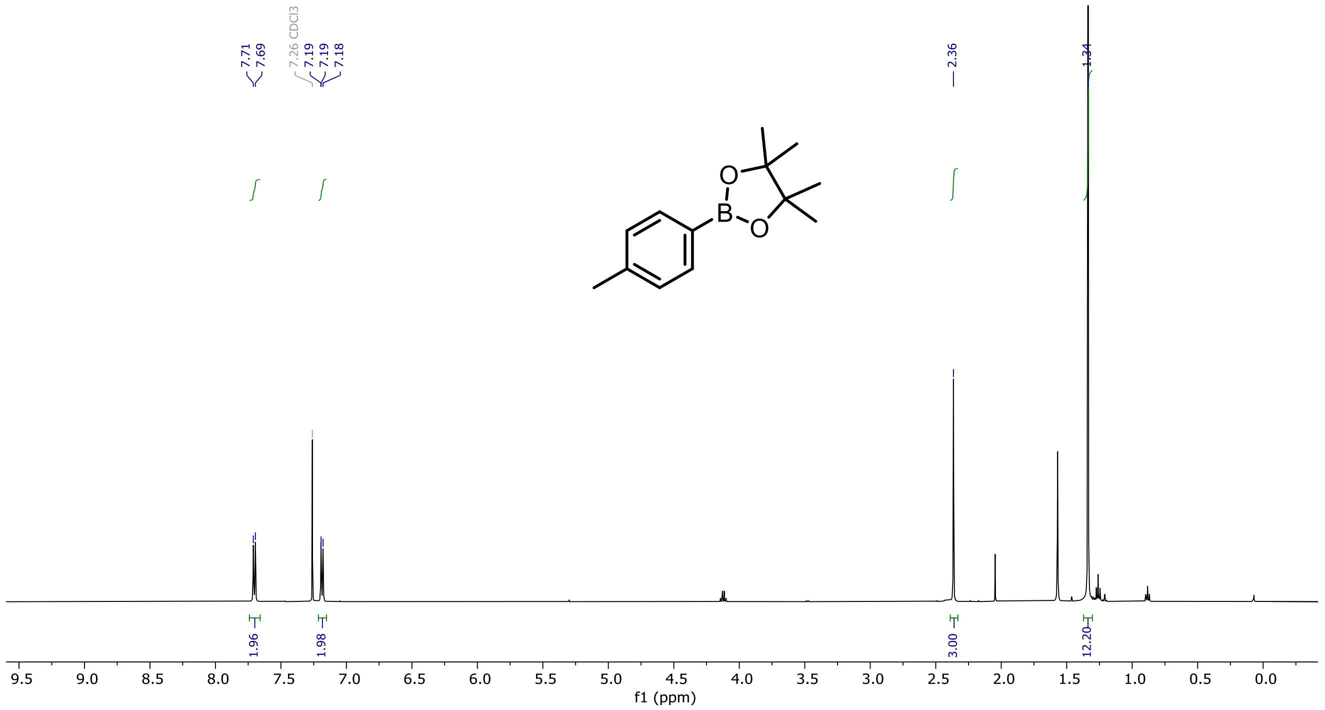

^{13}C{1H} NMR (126 MHz, $CDCl_3$)

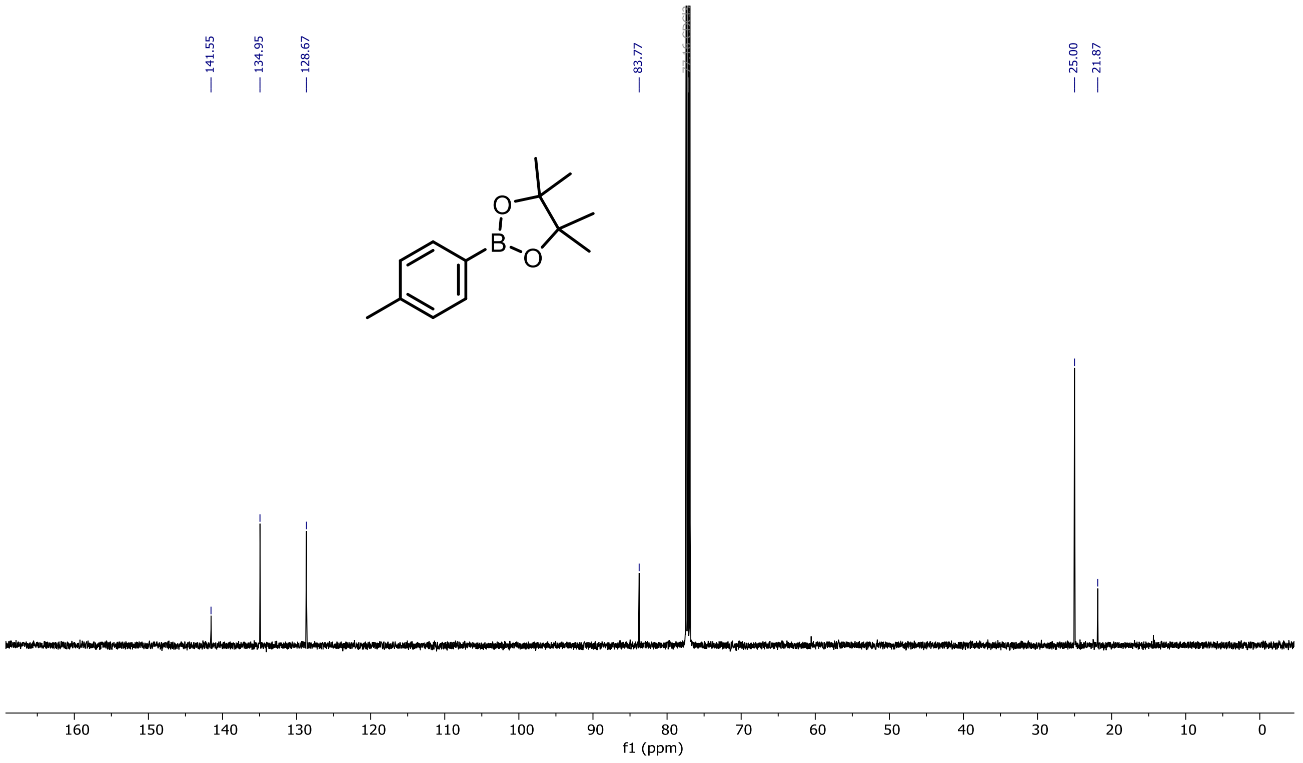

^{11}B NMR (160 MHz, $CDCl_3$)

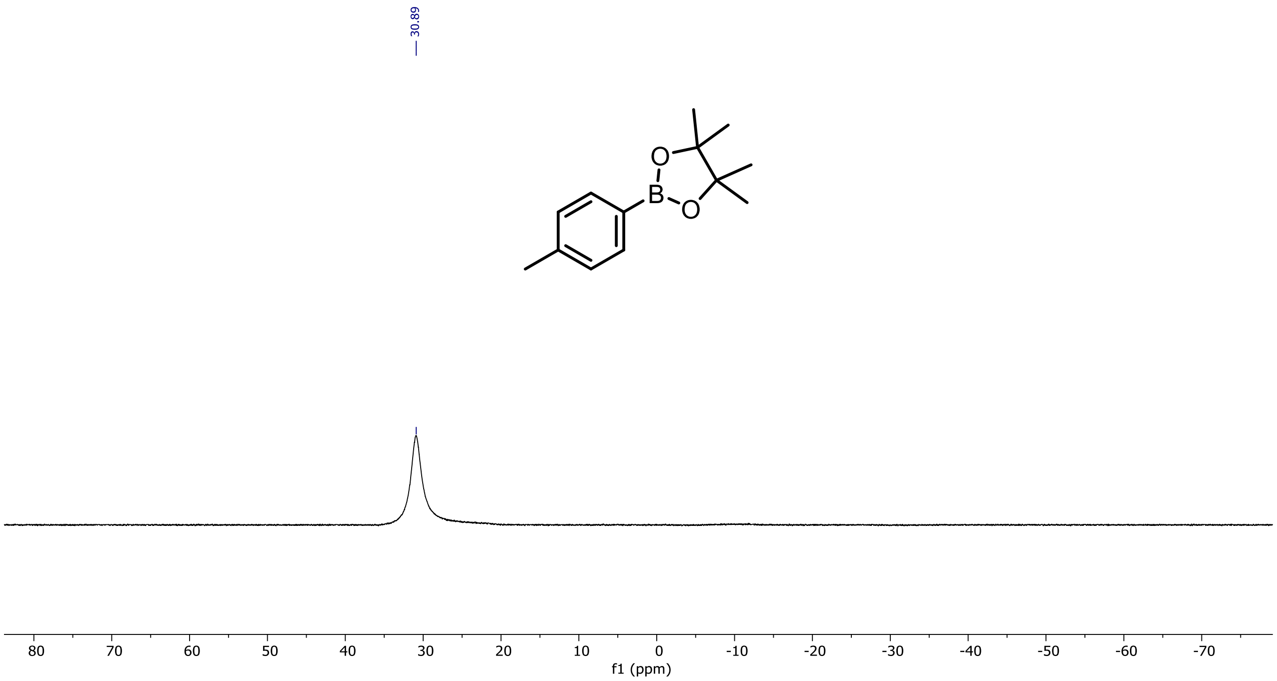

2-(4-(*tert*-Butyl)phenyl)-4,4,5,5-tetramethyl-1,3,2-dioxaborolane (6e)

^{1}H NMR (500 MHz, $CDCl_3$)

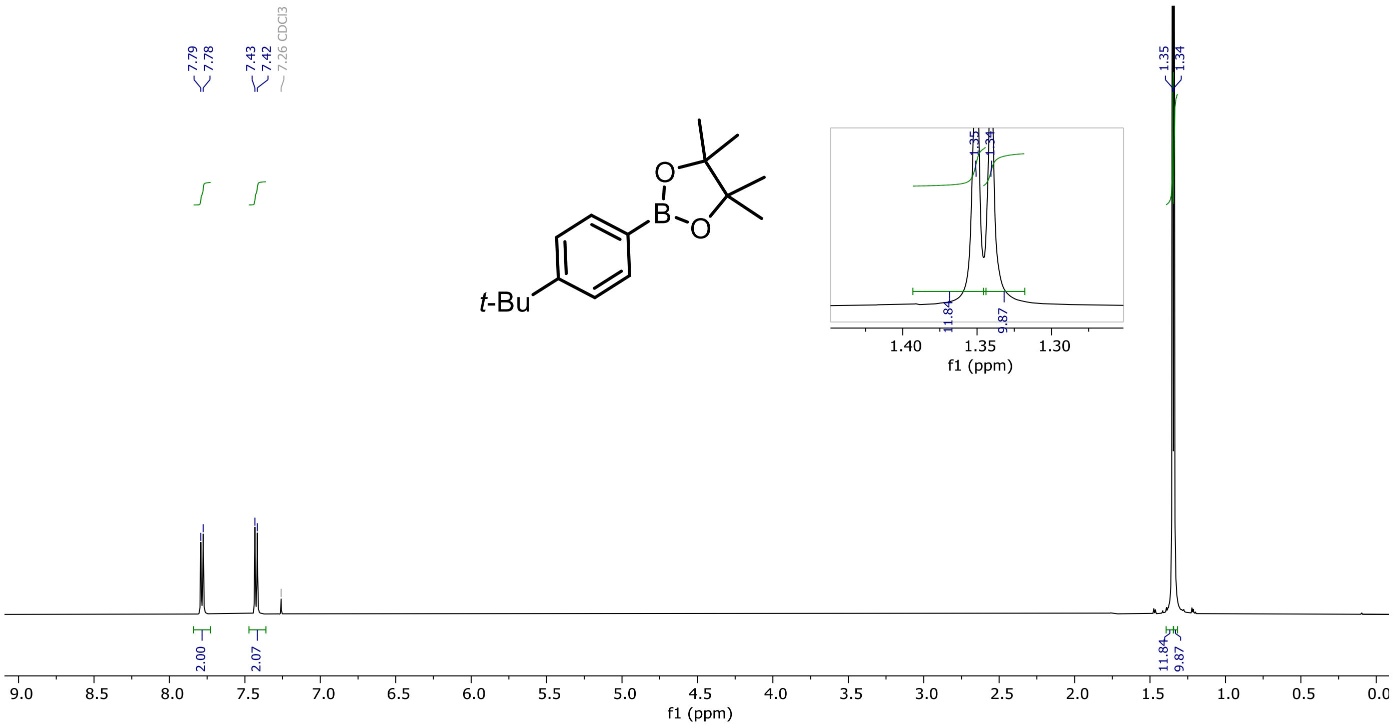

$^{13}C\{^{1}H\}$ NMR (126 MHz, $CDCl_3$)

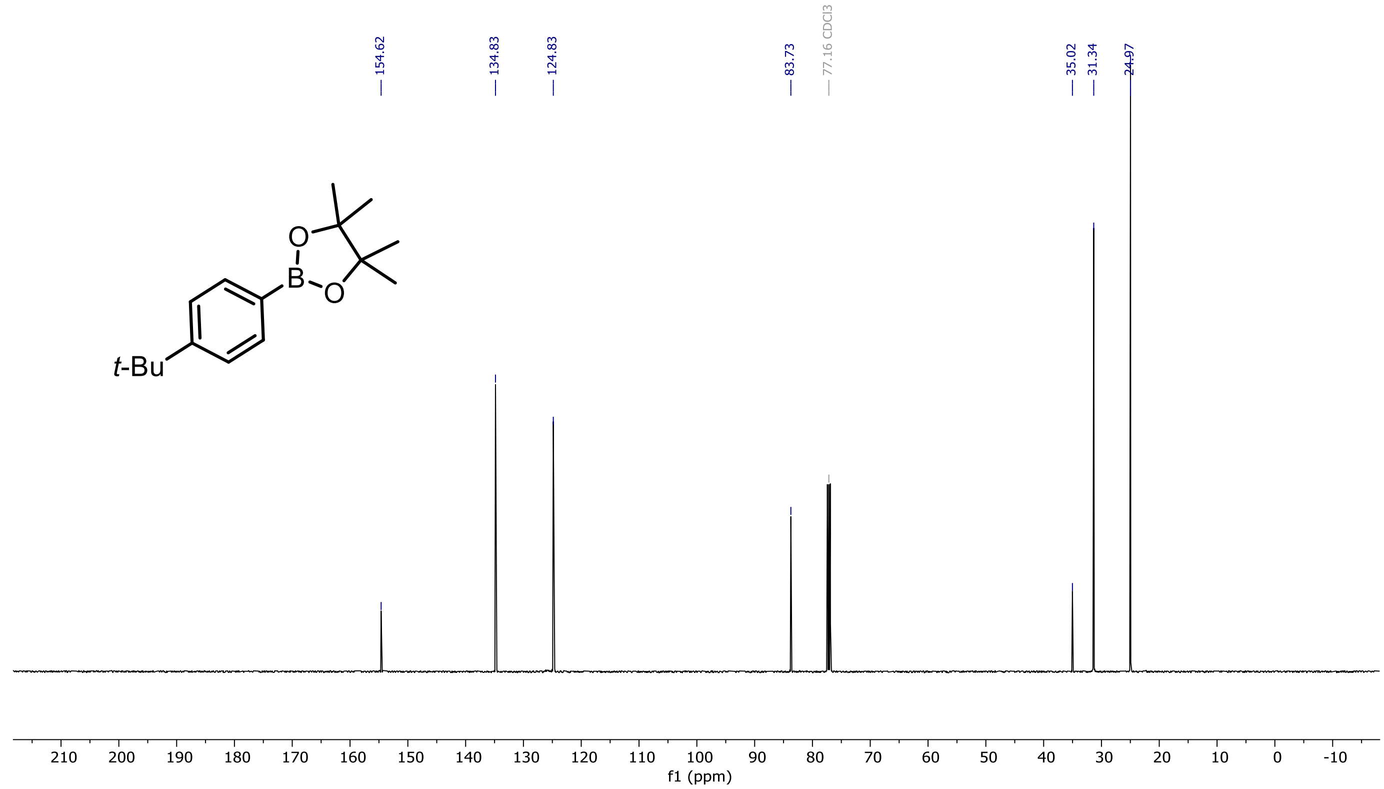

^{11}B NMR (160 MHz, $CDCl_3$)

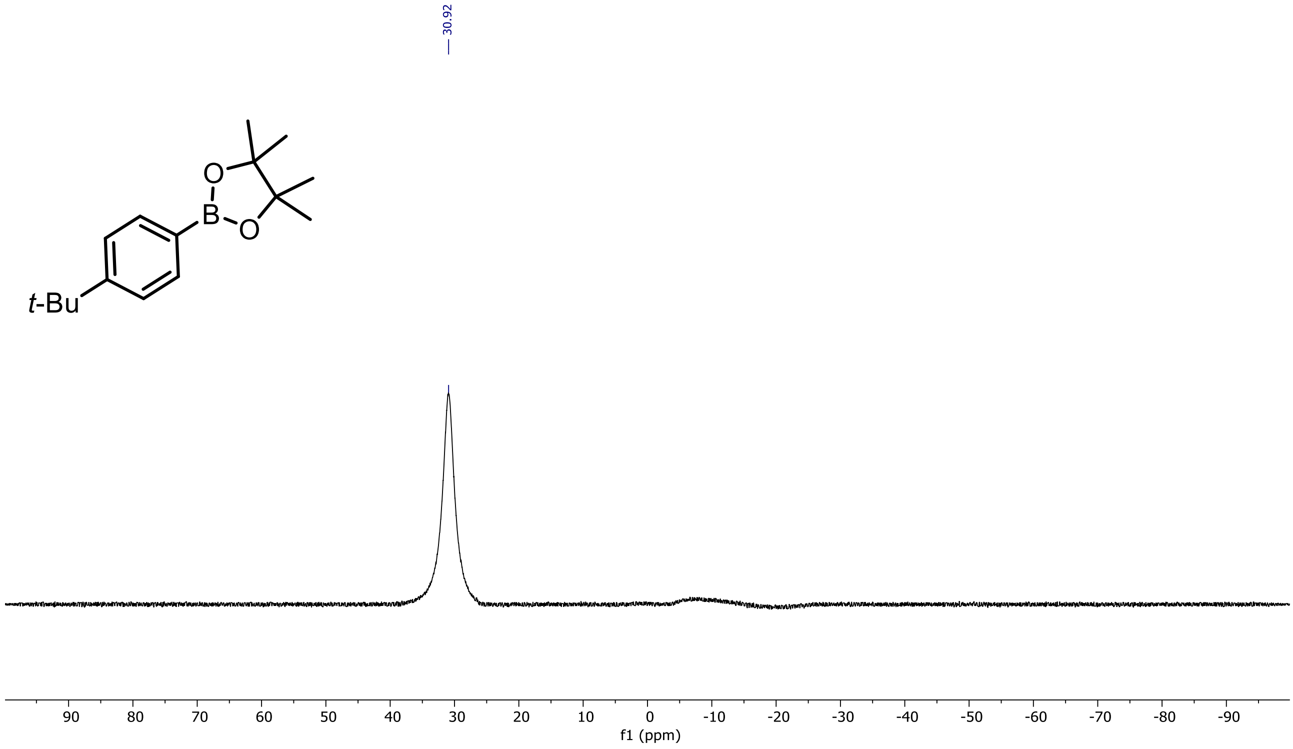

3-Ethyl-3-(4-(4,4,5,5-tetramethyl-1,3,2-dioxaborolan-2-yl)phenyl)piperidine-2,6-dione (6f)

^{1}H NMR (500 MHz, $CDCl_3$)

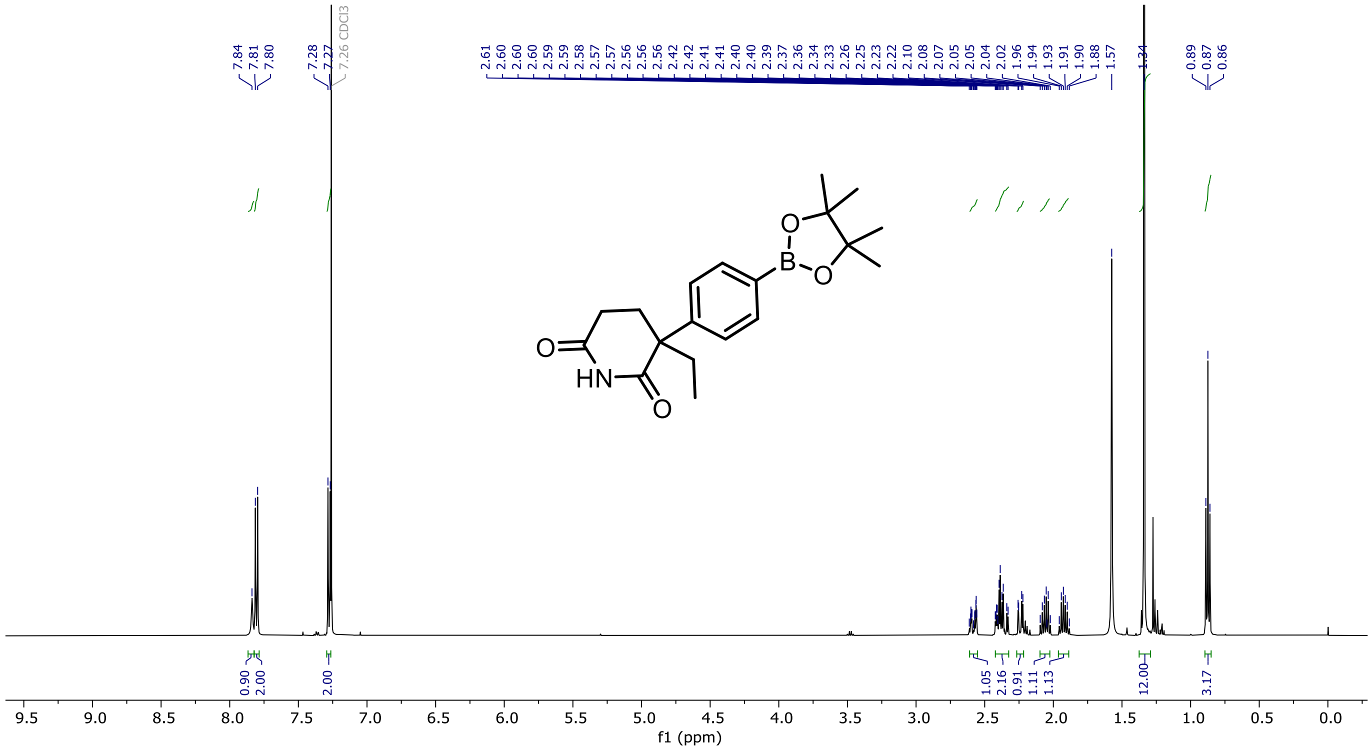

^{13}C{^{1}H} NMR (126 MHz, $CDCl_3$)

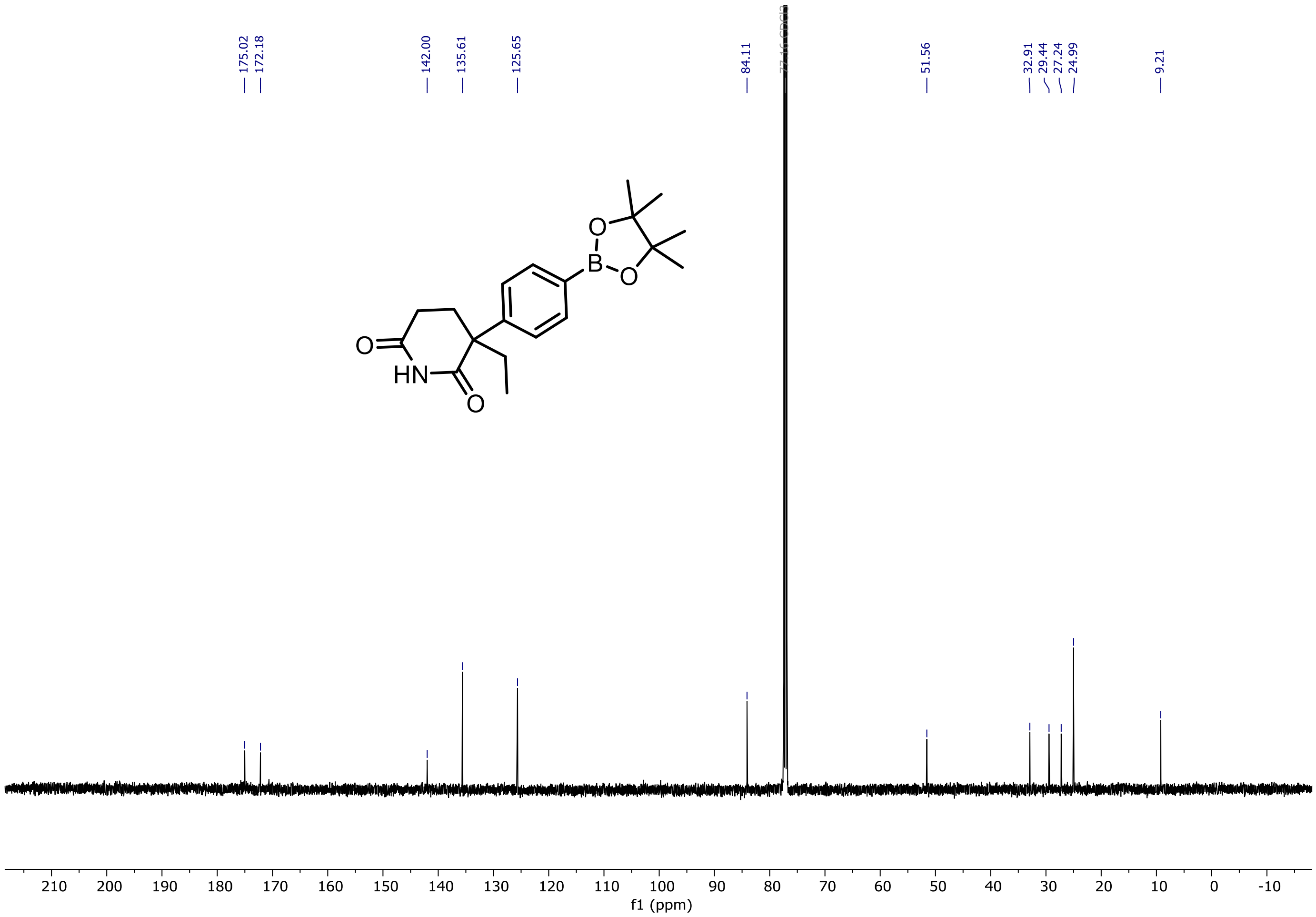

^{11}B NMR (160 MHz, $CDCl_3$)

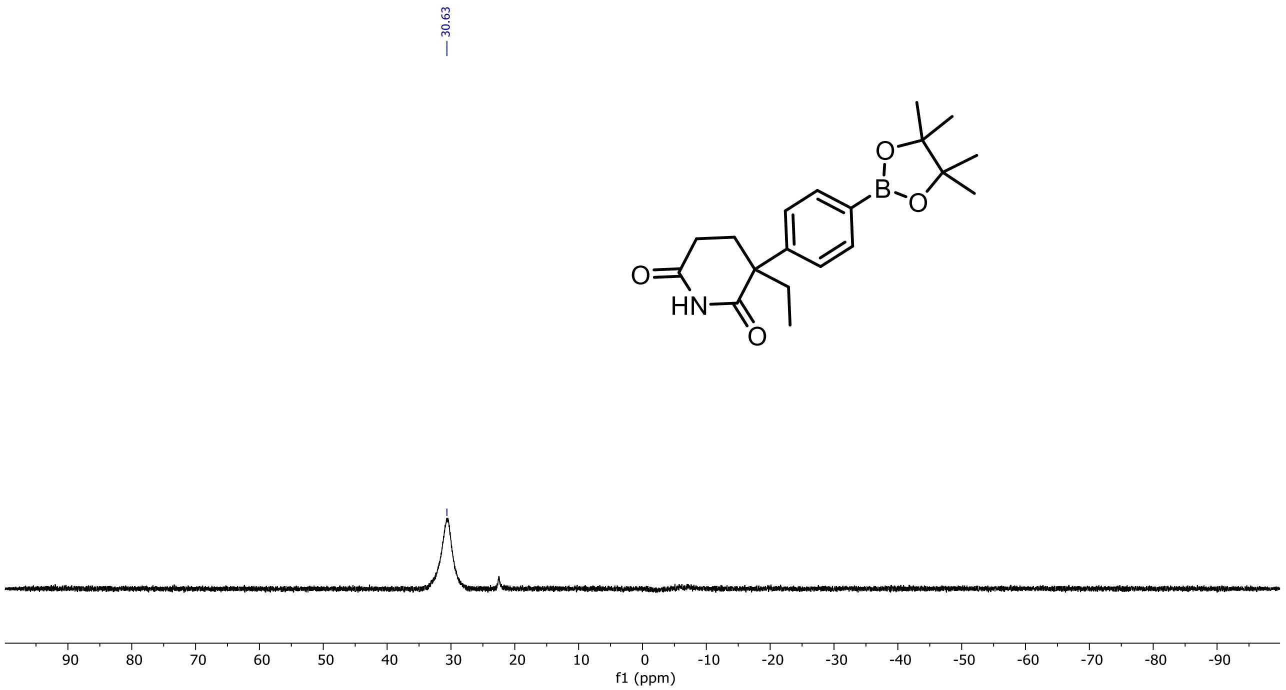

4,4,5,5-Tetramethyl-2-(naphthalen-1-yl)-1,3,2-dioxaborolane (6g)

^{1}H NMR (500 MHz, $CDCl_3$)

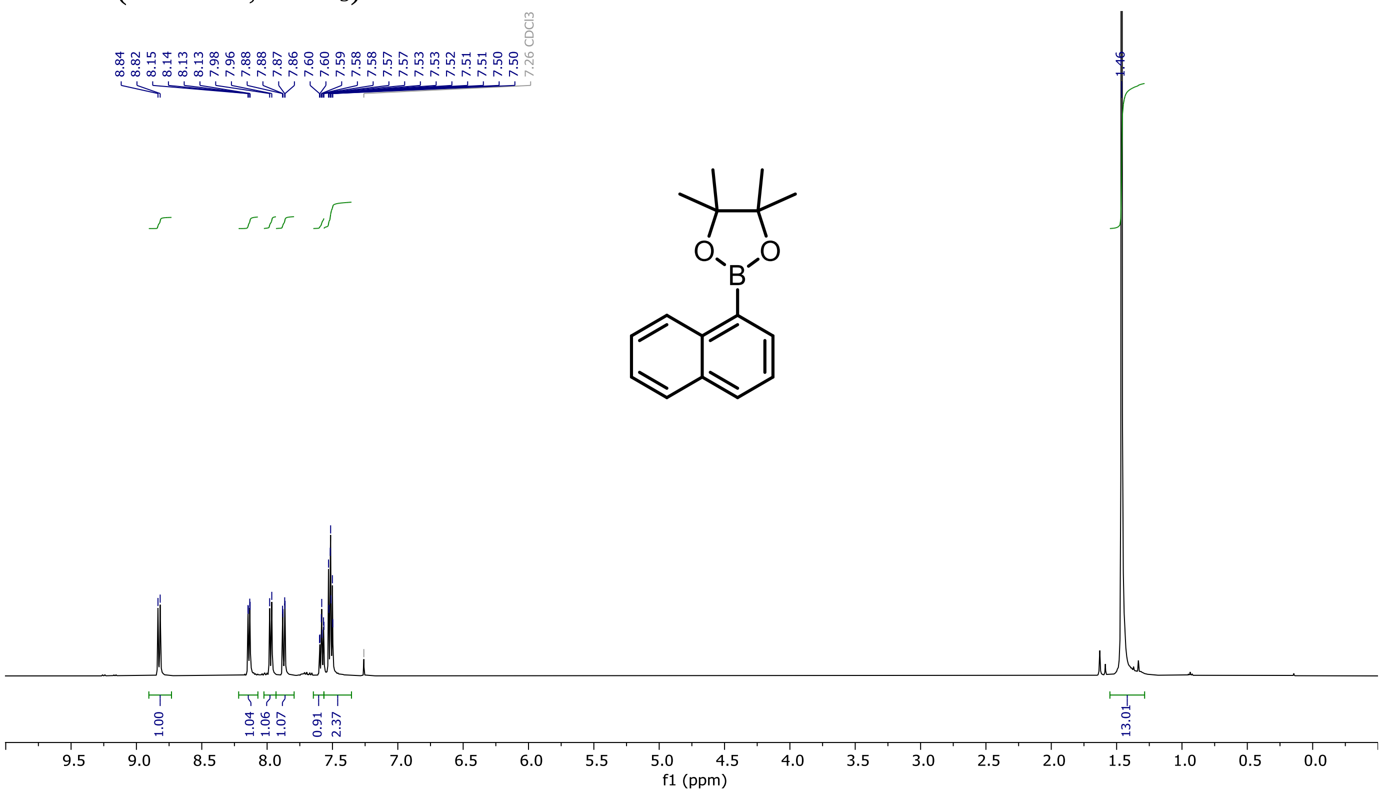

$^{13}C\{^{1}H\}$ NMR (126 MHz, $CDCl_3$)

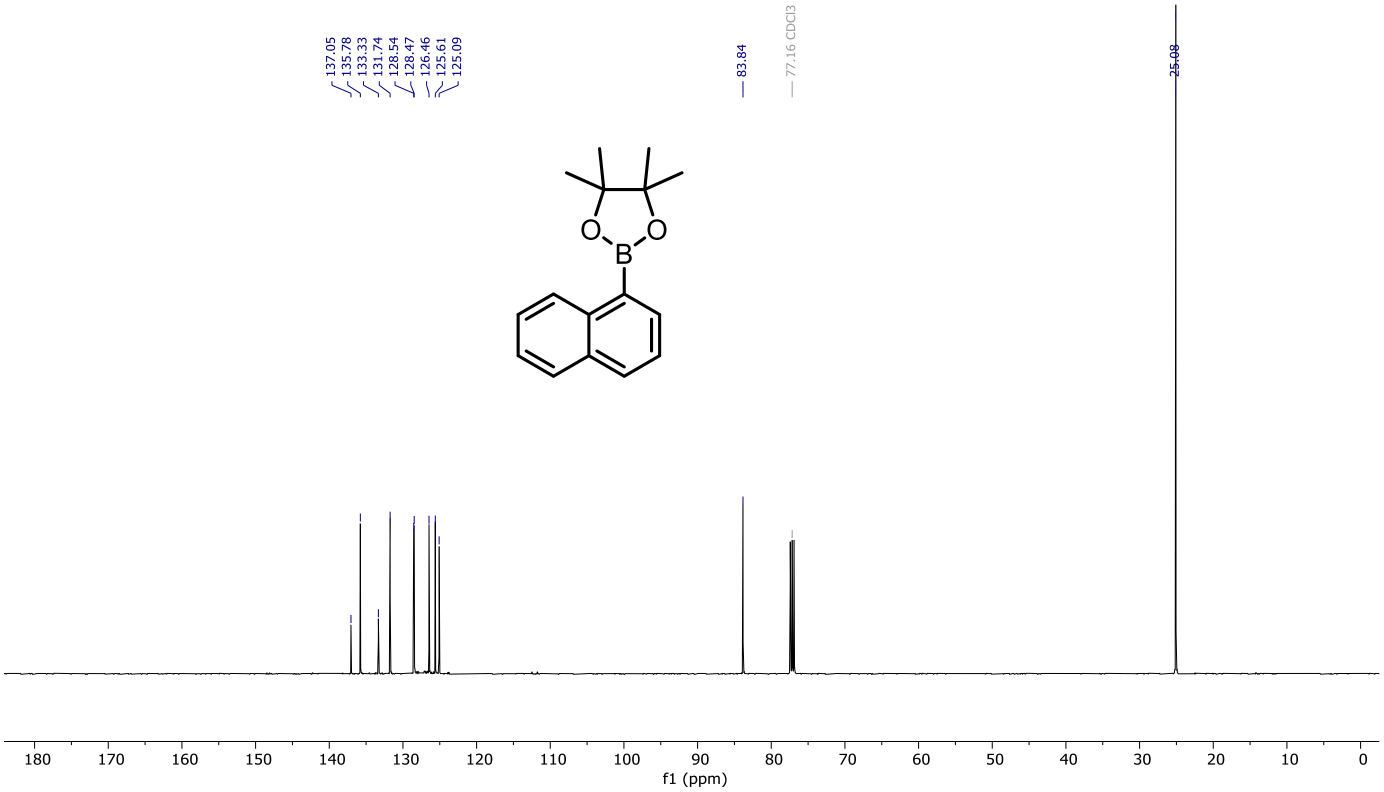

^{11}B NMR (160 MHz, $CDCl_3$)

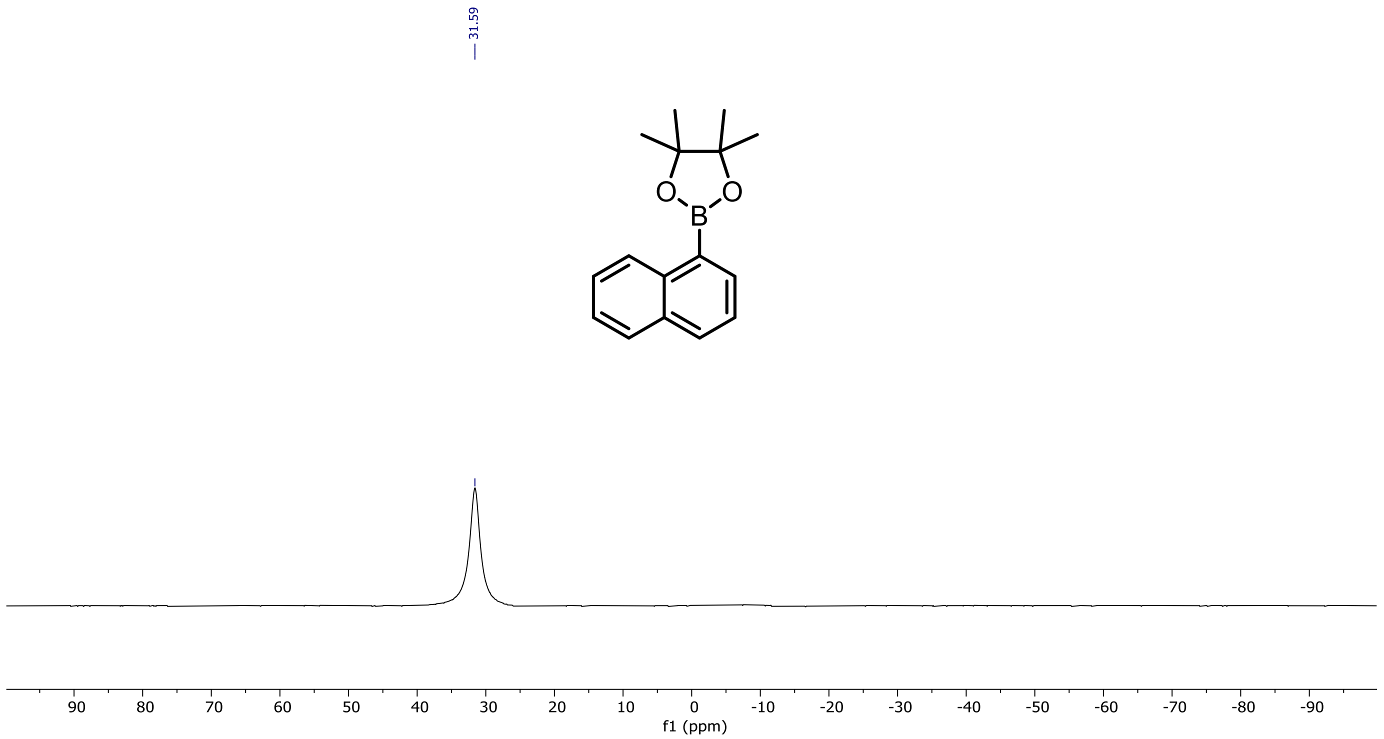

N,N-Dimethyl-4-(4,4,5,5-tetramethyl-1,3,2-dioxaborolan-2-yl)aniline (6h)

^{1}H NMR (500 MHz, $CDCl_3$)

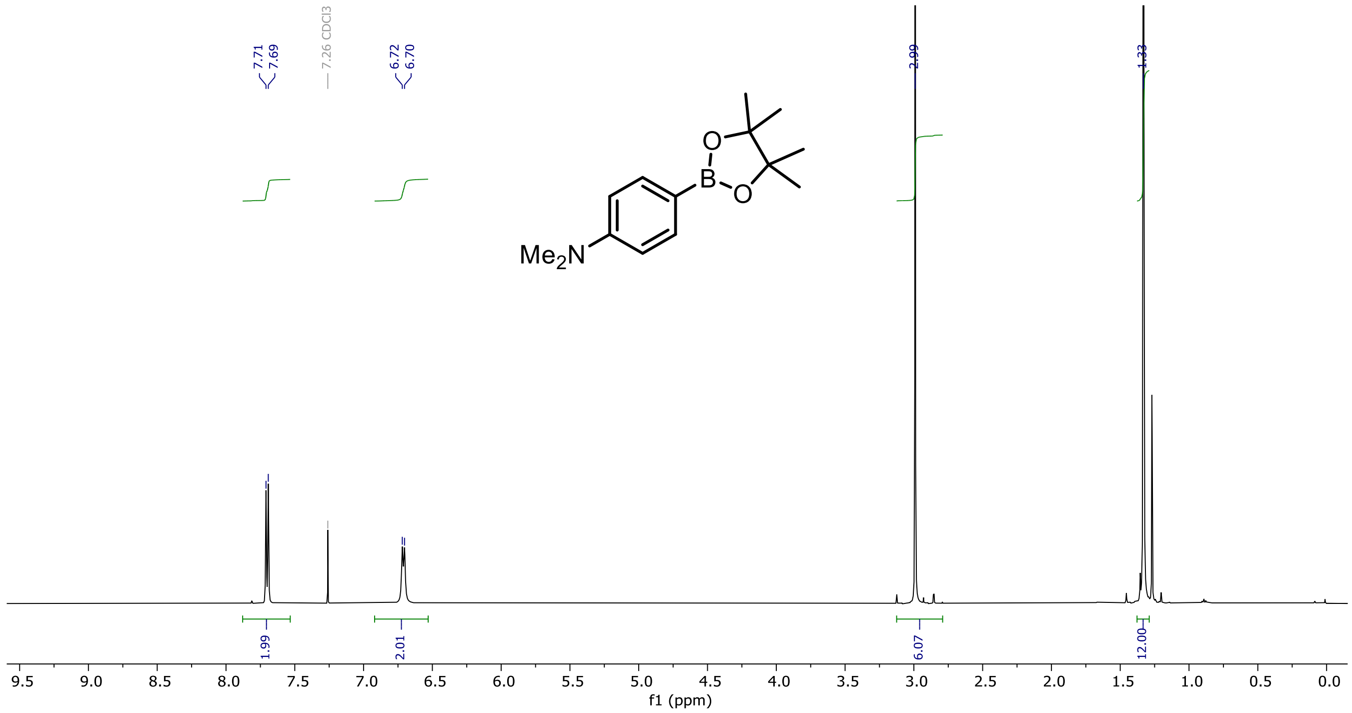

^{13}C{^{1}H} NMR (126 MHz, $CDCl_3$)

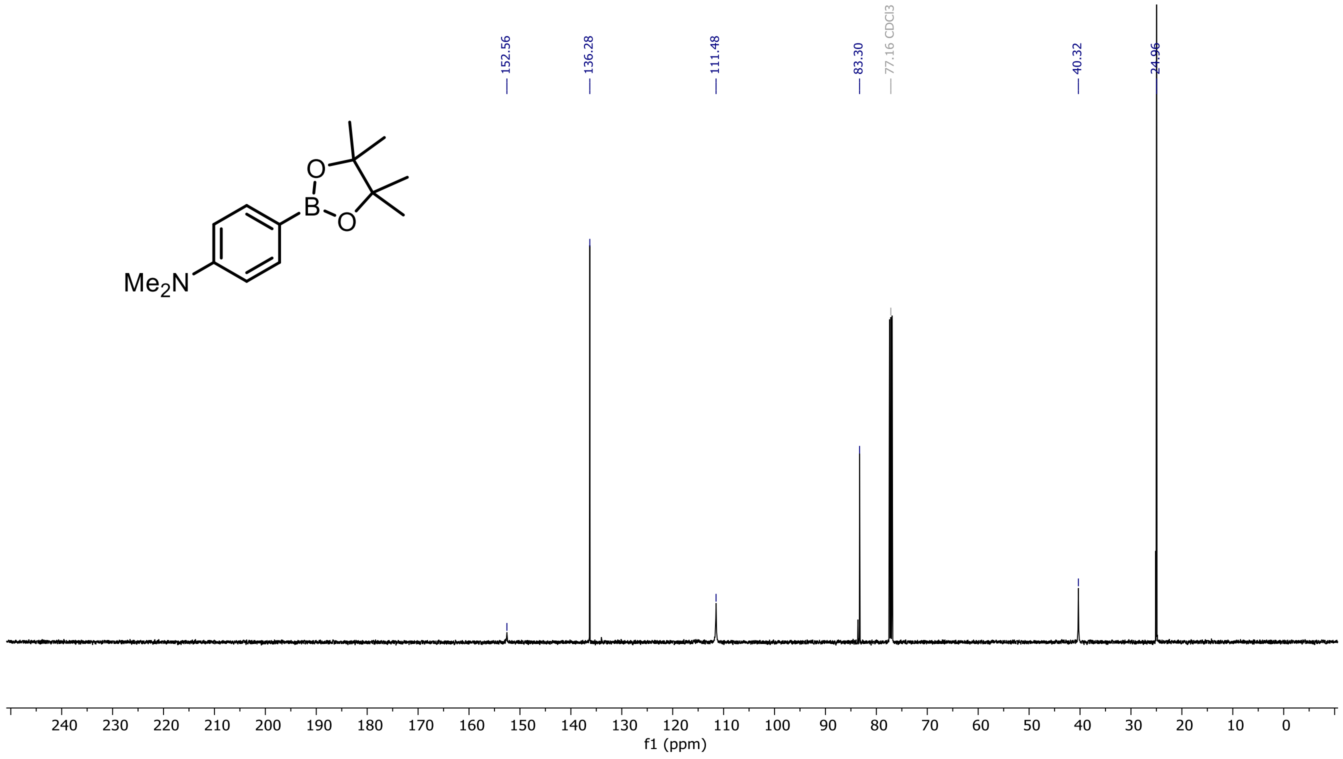

^{11}B NMR (160 MHz, $CDCl_3$)

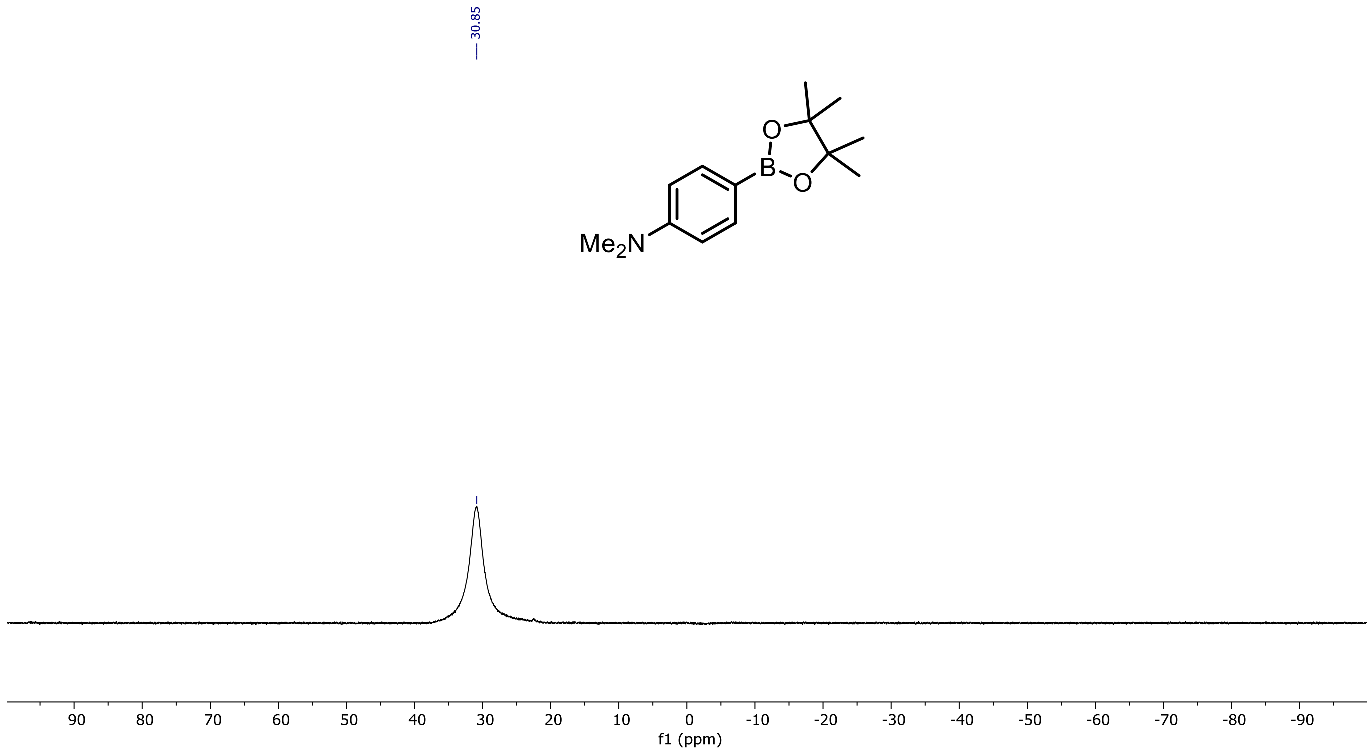

2-(3-Methoxyphenyl)-4,4,5,5-tetramethyl-1,3,2-dioxaborolane (6i)

^{1}H NMR (500 MHz, $CDCl_3$)

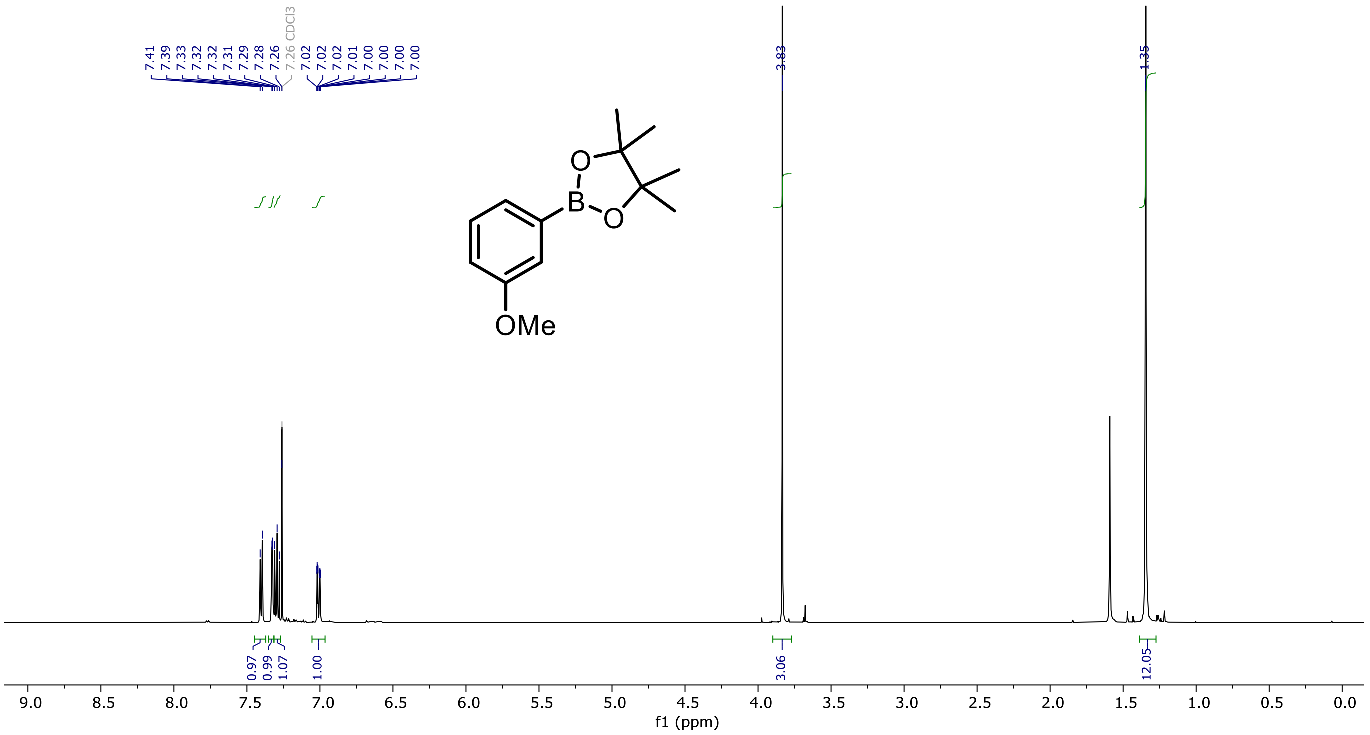

$^{13}C\{^{1}H\}$ NMR (126 MHz, $CDCl_3$)

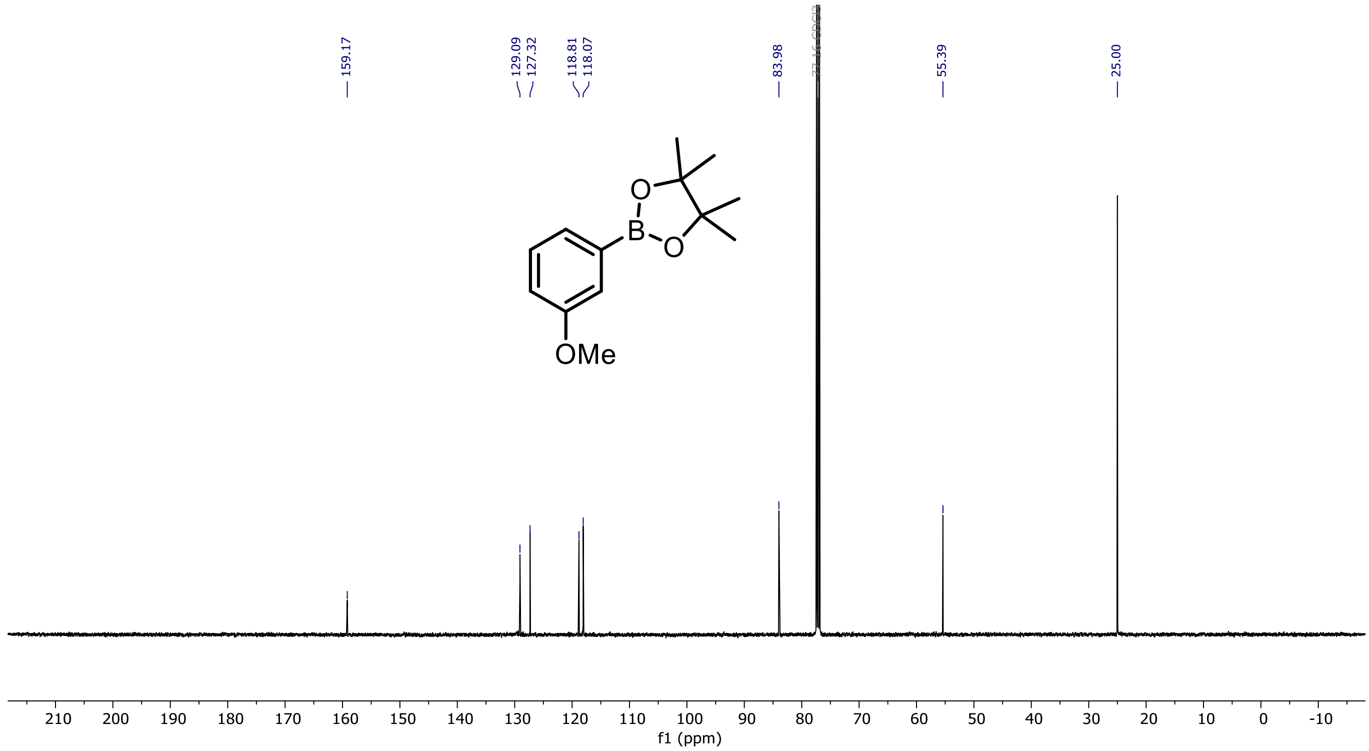

^{11}B NMR (160 MHz, $CDCl_3$)

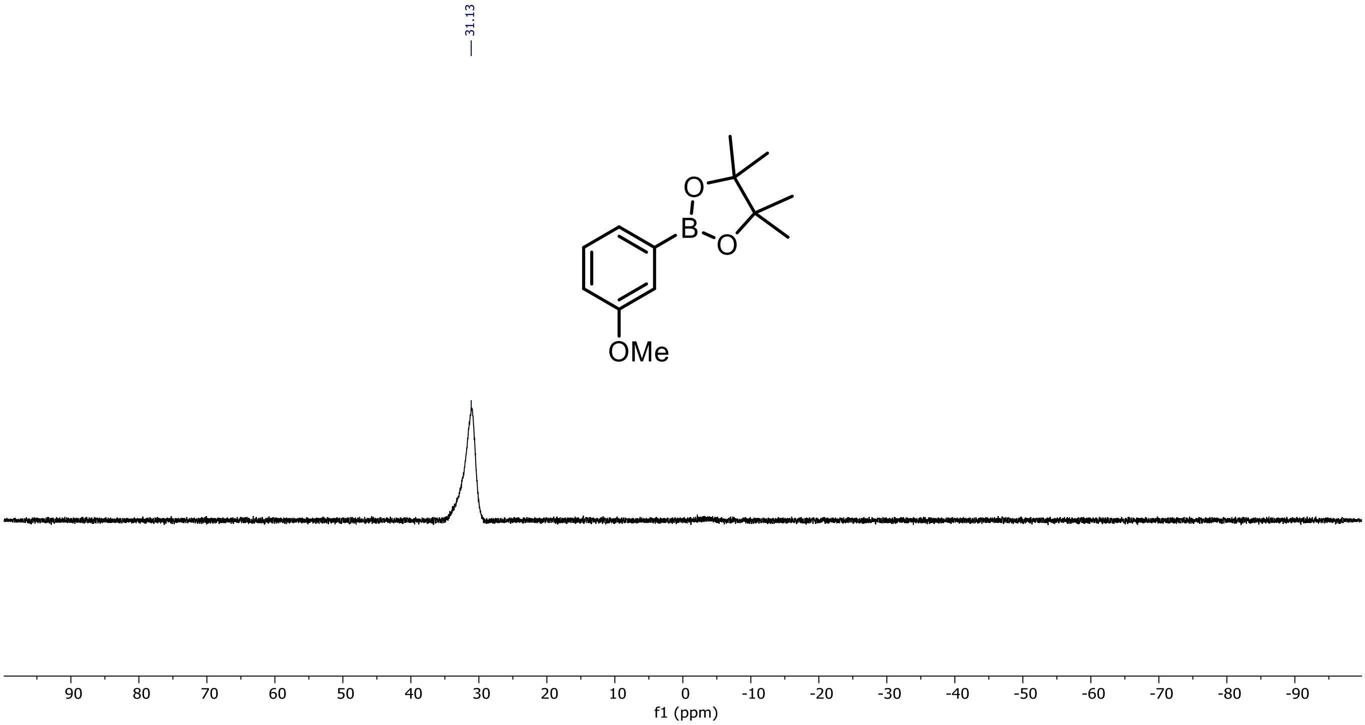

2-(3,5-Dimethylphenyl)-4,4,5,5-tetramethyl-1,3,2-dioxaborolane (6j)

^{1}H NMR (500 MHz, $CDCl_3$)

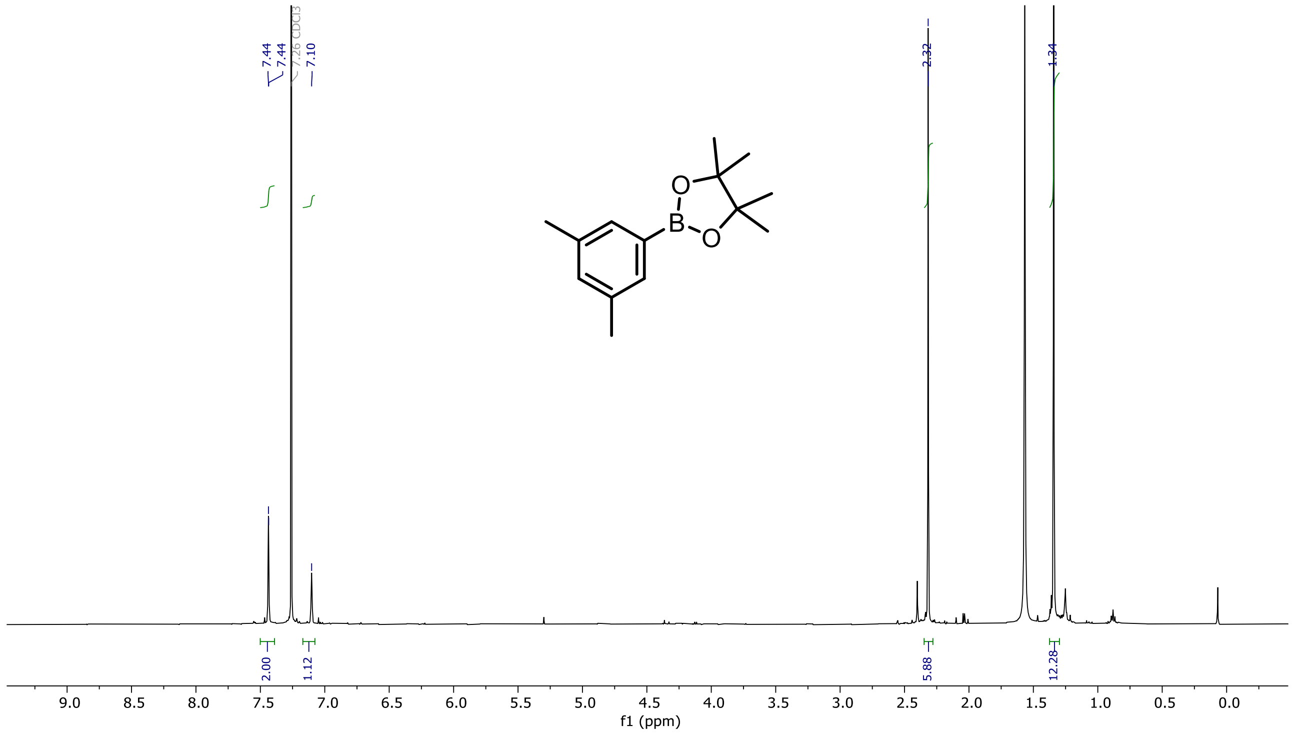

^{13}C{^{1}H} NMR (126 MHz, $CDCl_3$)

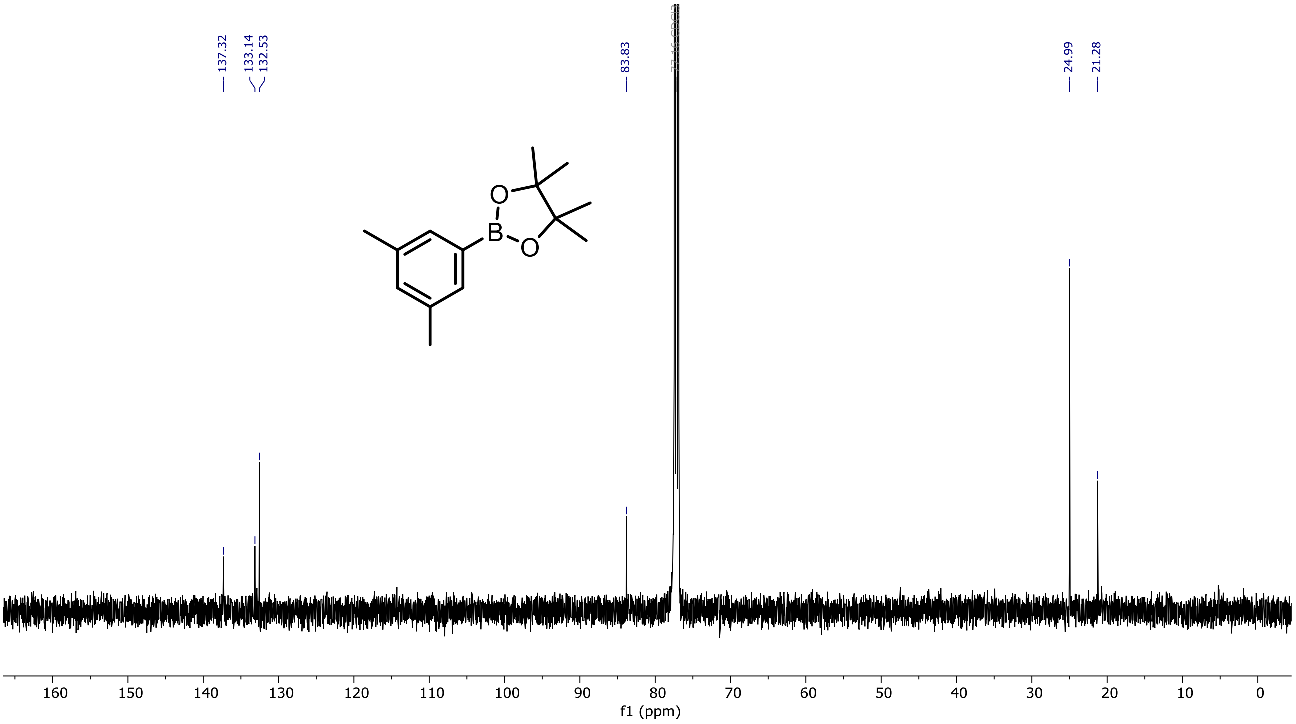

^{11}B NMR (160 MHz, $CDCl_3$)

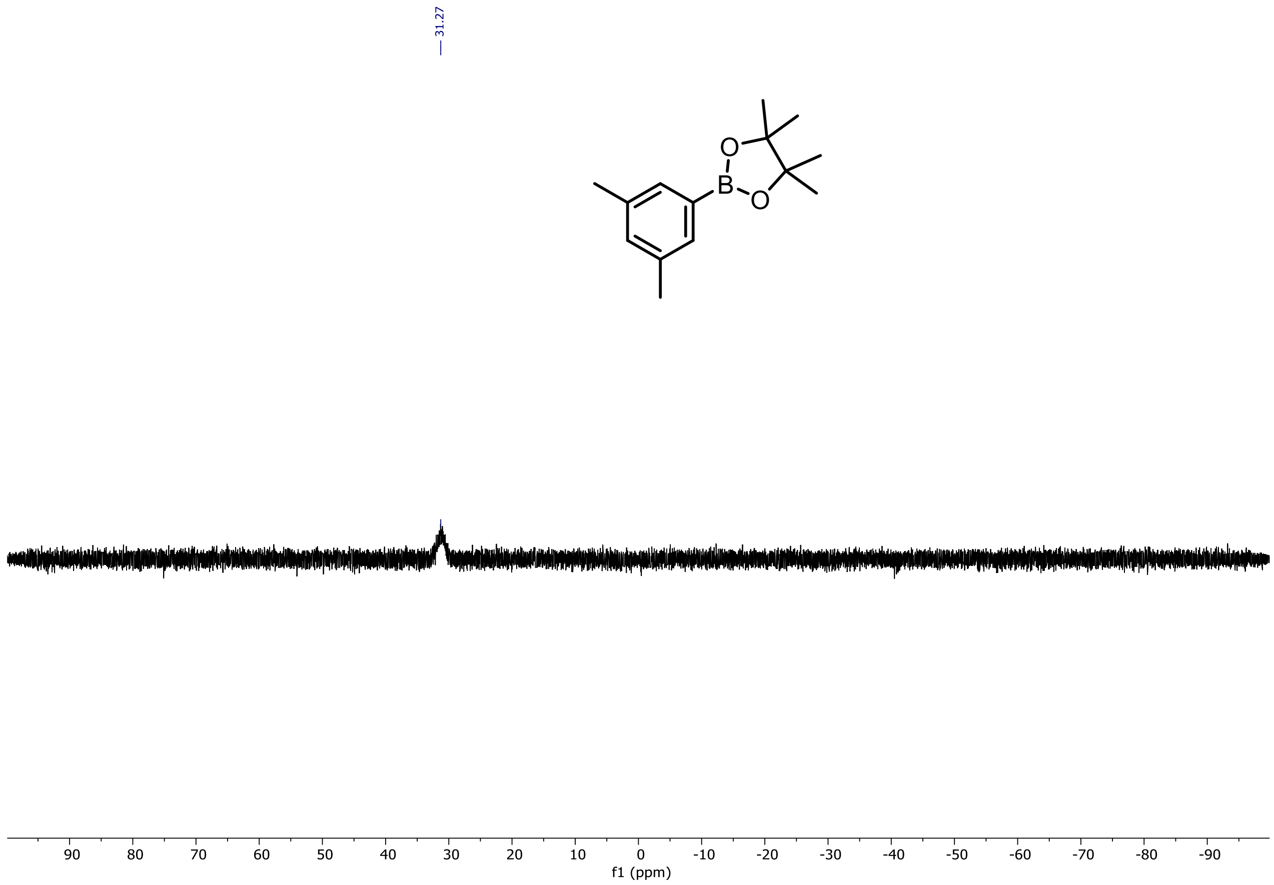

2-Mesityl-4,4,5,5-tetramethyl-1,3,2-dioxaborolane (6k)

^{1}H NMR (500 MHz, $CDCl_3$)

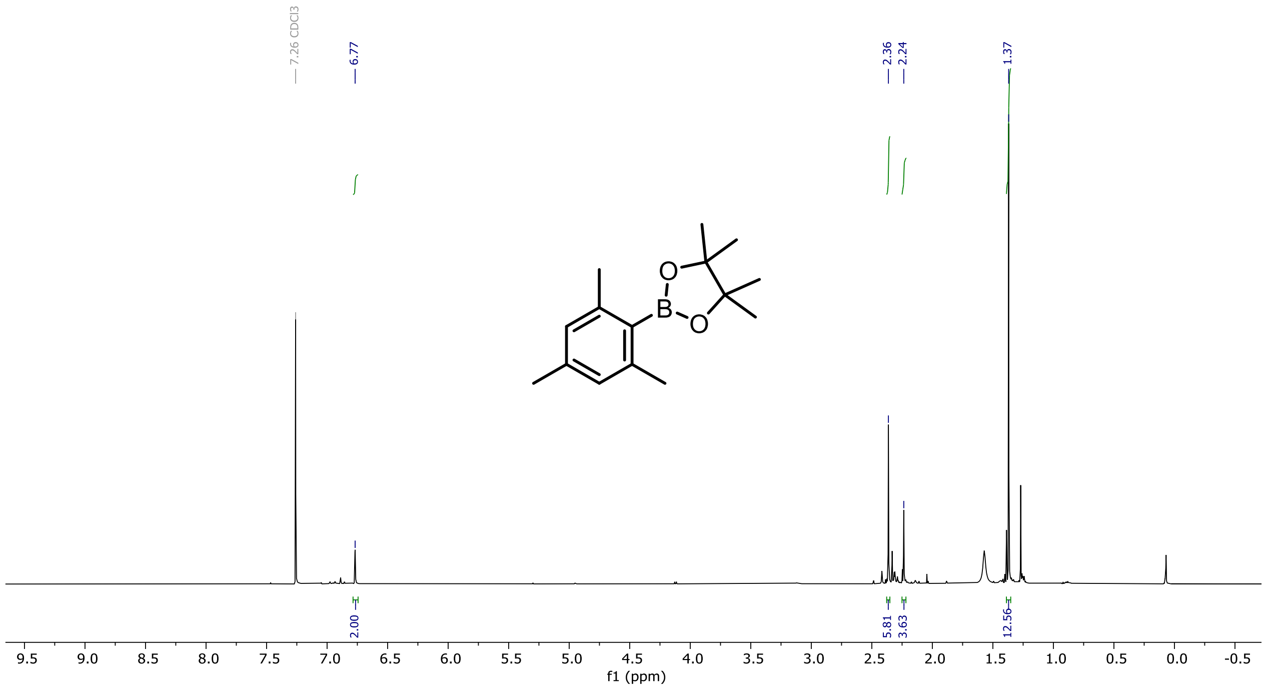

$^{13}C\{^{1}H\}$ NMR (126 MHz, $CDCl_3$)

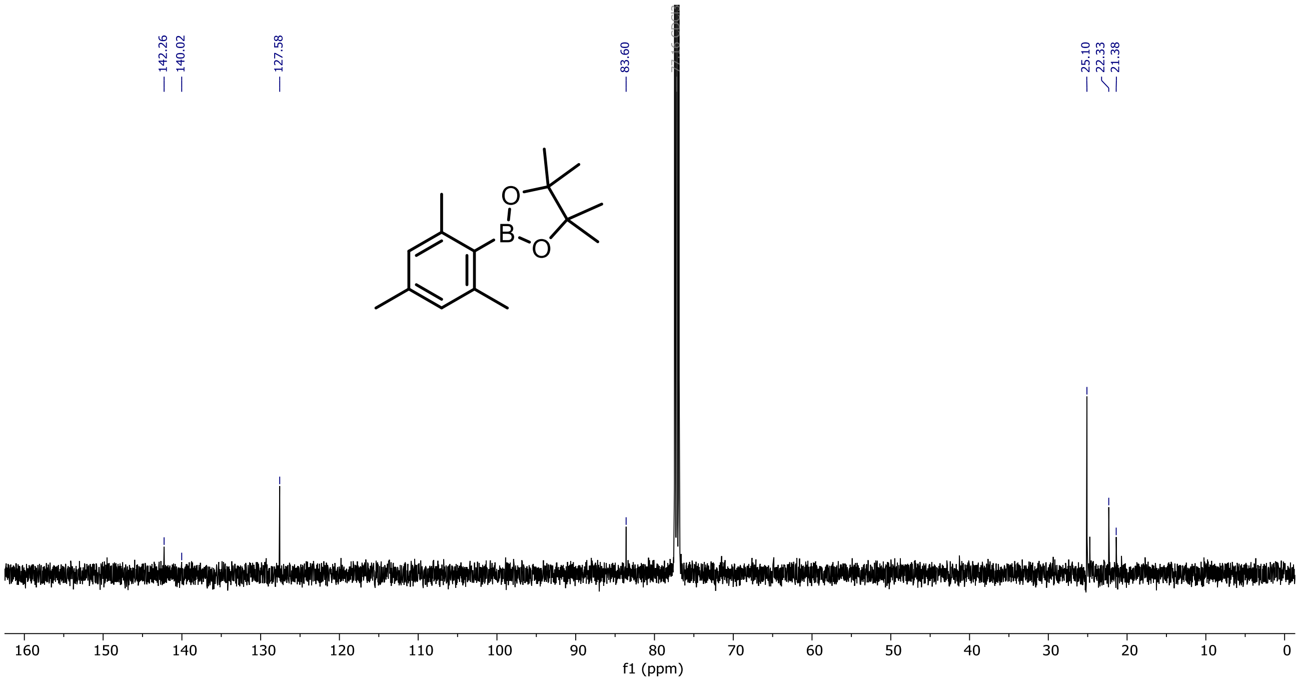

^{11}B NMR (160 MHz, $CDCl_3$)

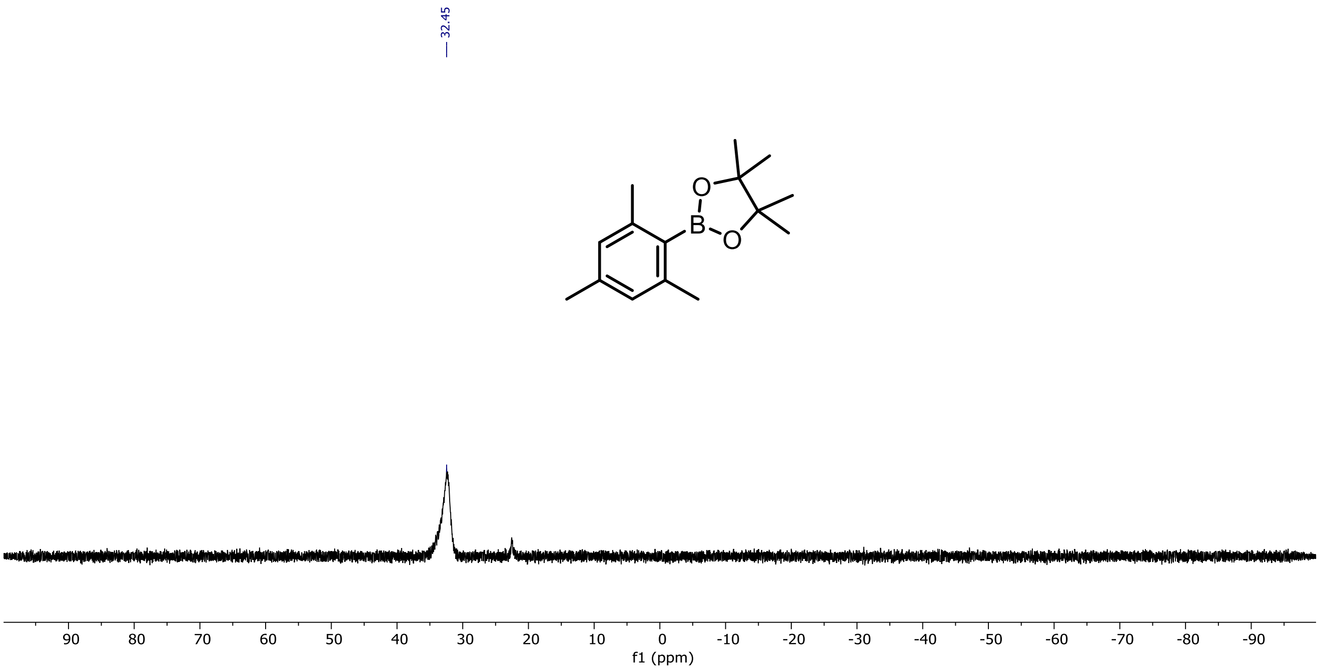

2-(4-Fluorophenyl)-4,4,5,5-tetramethyl-1,3,2-dioxaborolane (6m)

^{1}H NMR (500 MHz, CDCl$_3$)

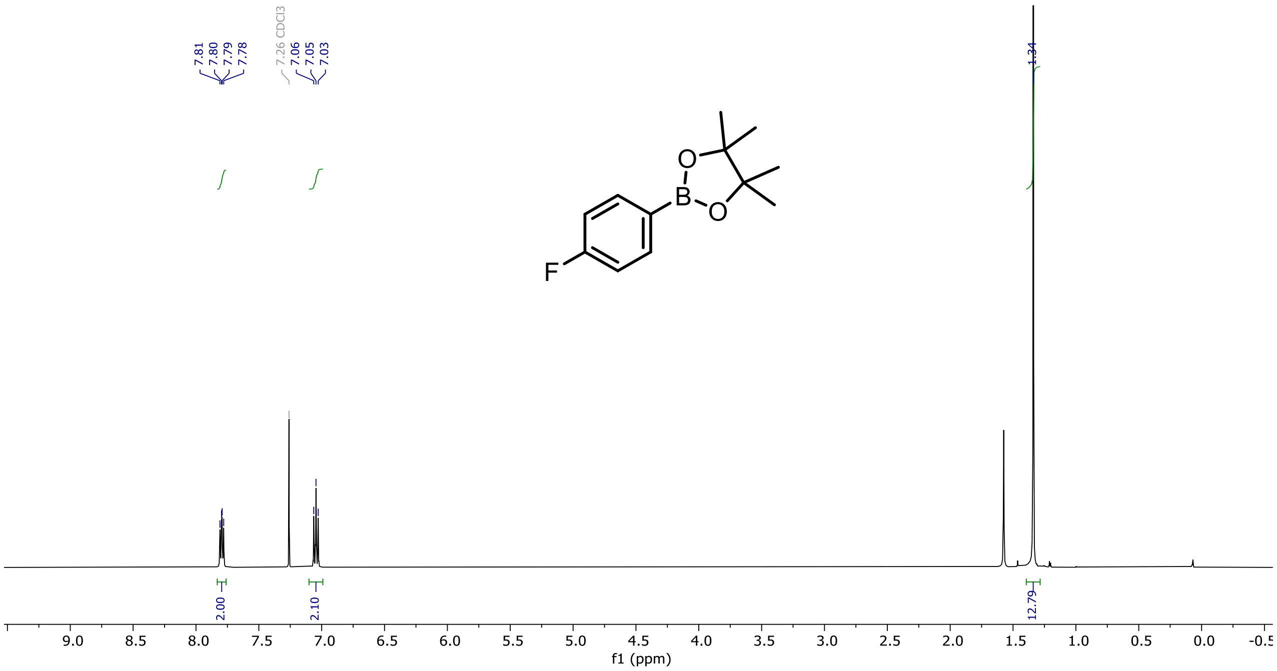

^{13}C{^{1}H} NMR (126 MHz, CDCl$_3$)

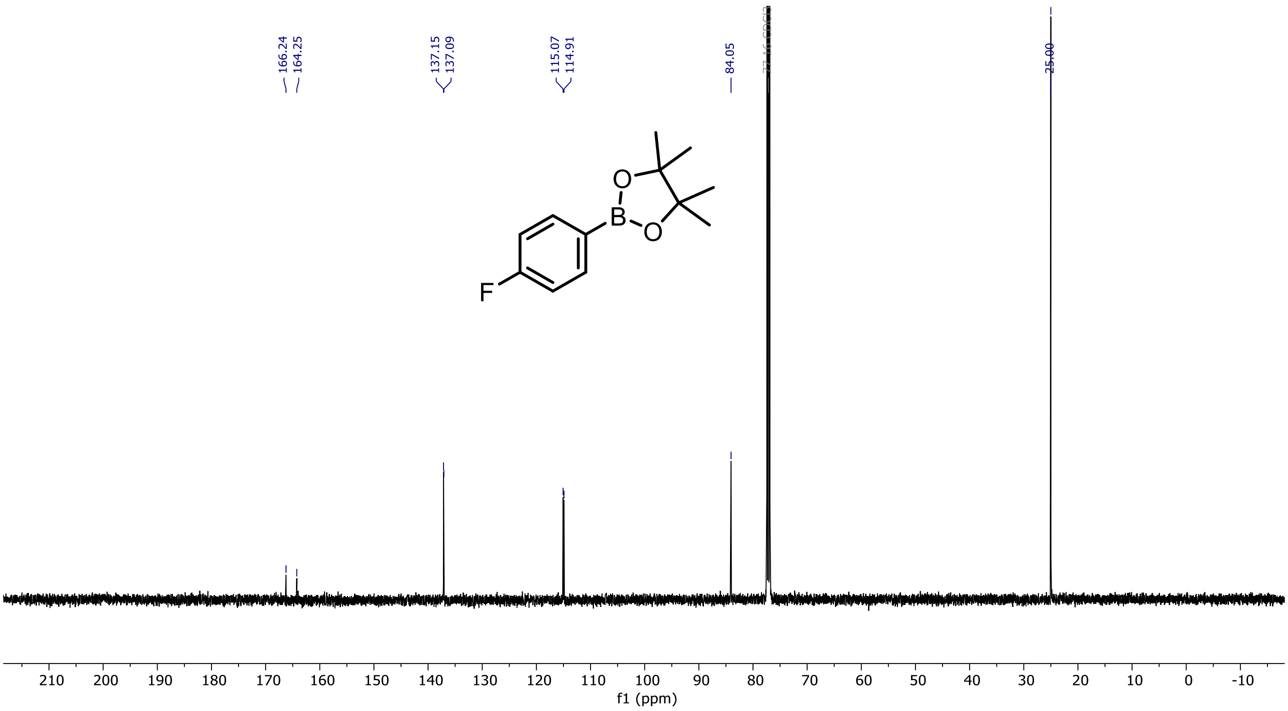

^{11}B NMR (160 MHz, $CDCl_3$)

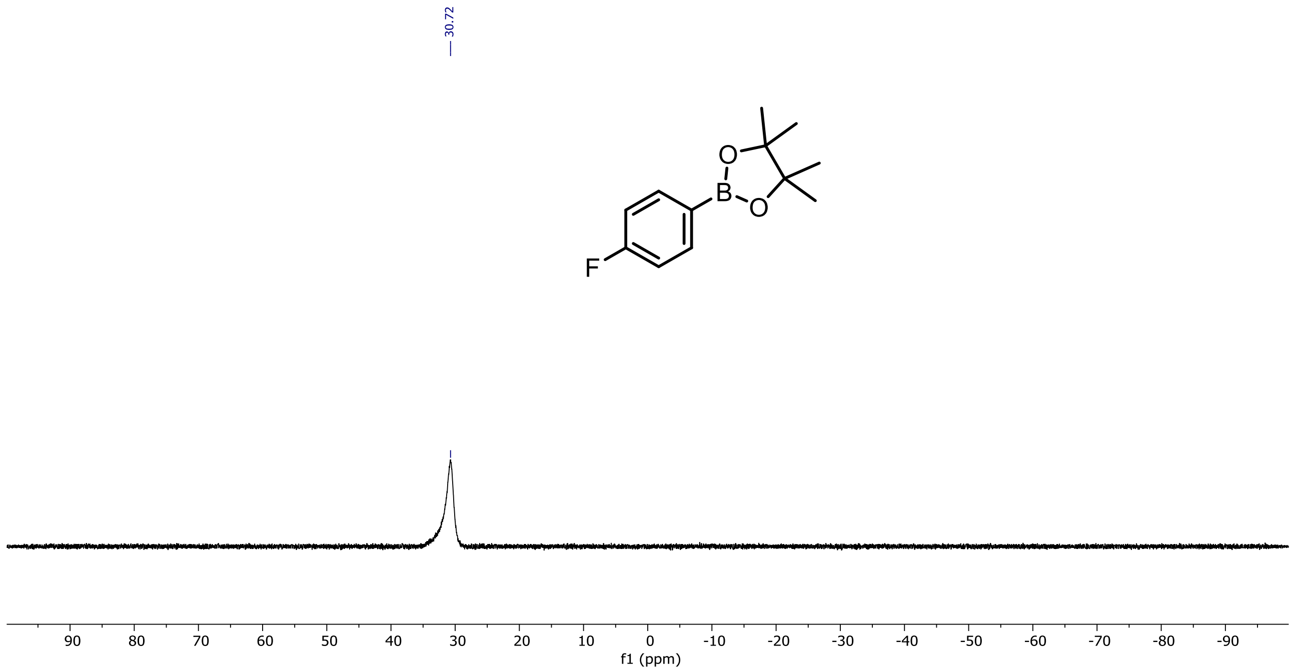

2-(4-Chlorophenyl)-4,4,5,5-tetramethyl-1,3,2-dioxaborolane (6n)

^{1}H NMR (500 MHz, $CDCl_3$)

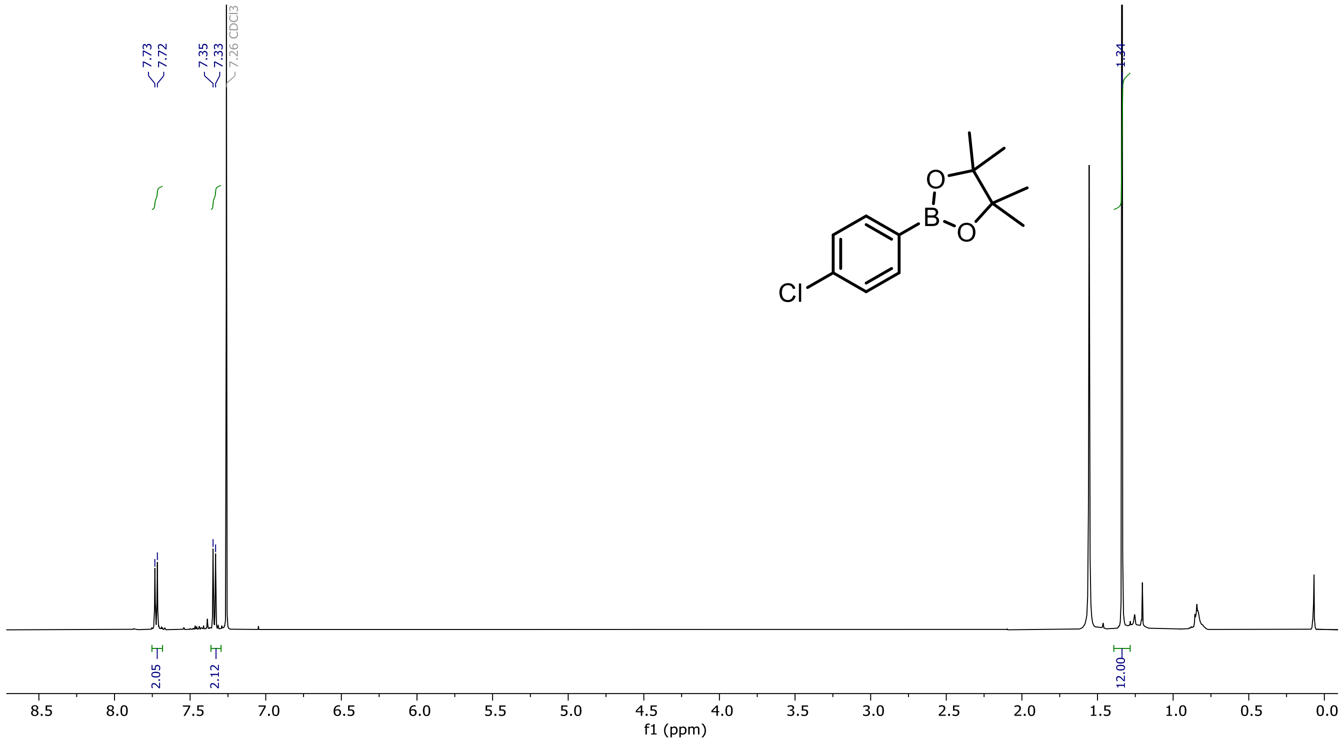

$^{13}C\{^{1}H\}$ NMR (126 MHz, $CDCl_3$)

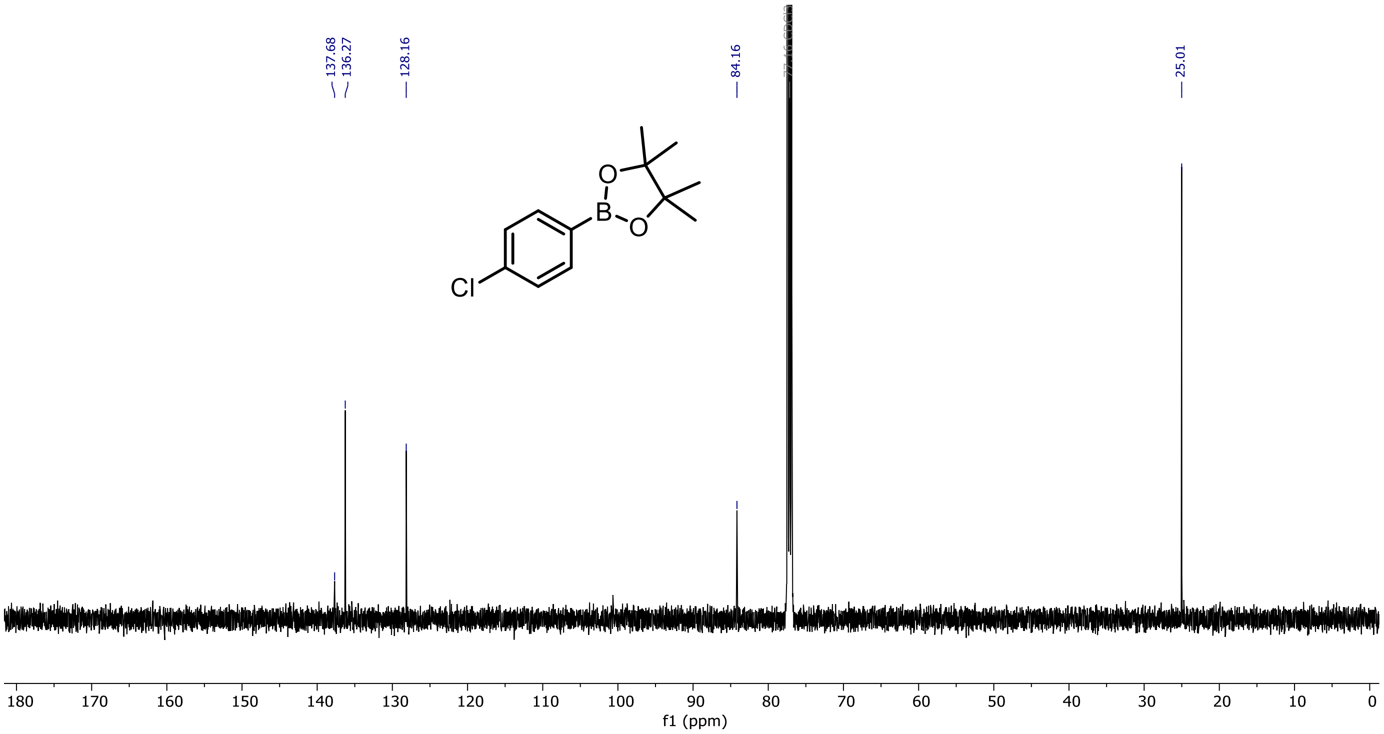

^{11}B NMR (160 MHz, $CDCl_3$)

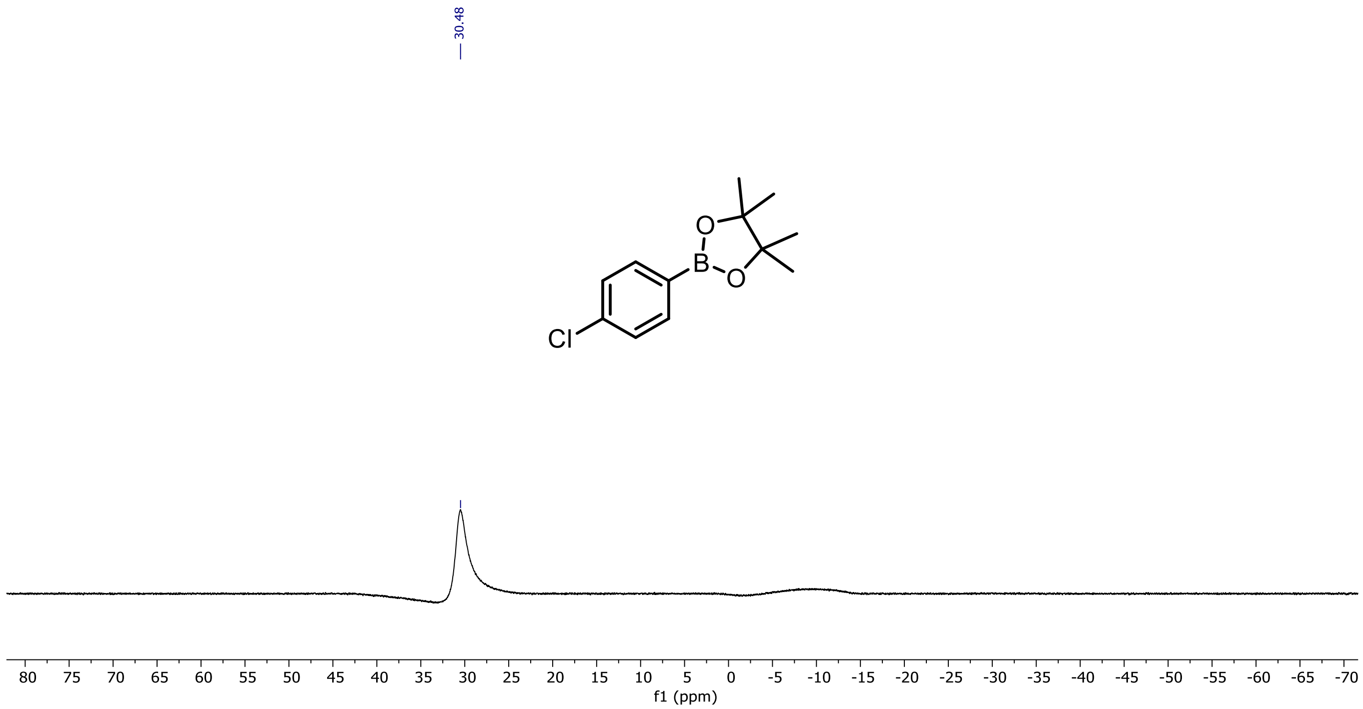

2-(4-Bromophenyl)-4,4,5,5-tetramethyl-1,3,2-dioxaborolane (6o)

^{1}H NMR (500 MHz, $CDCl_3$)

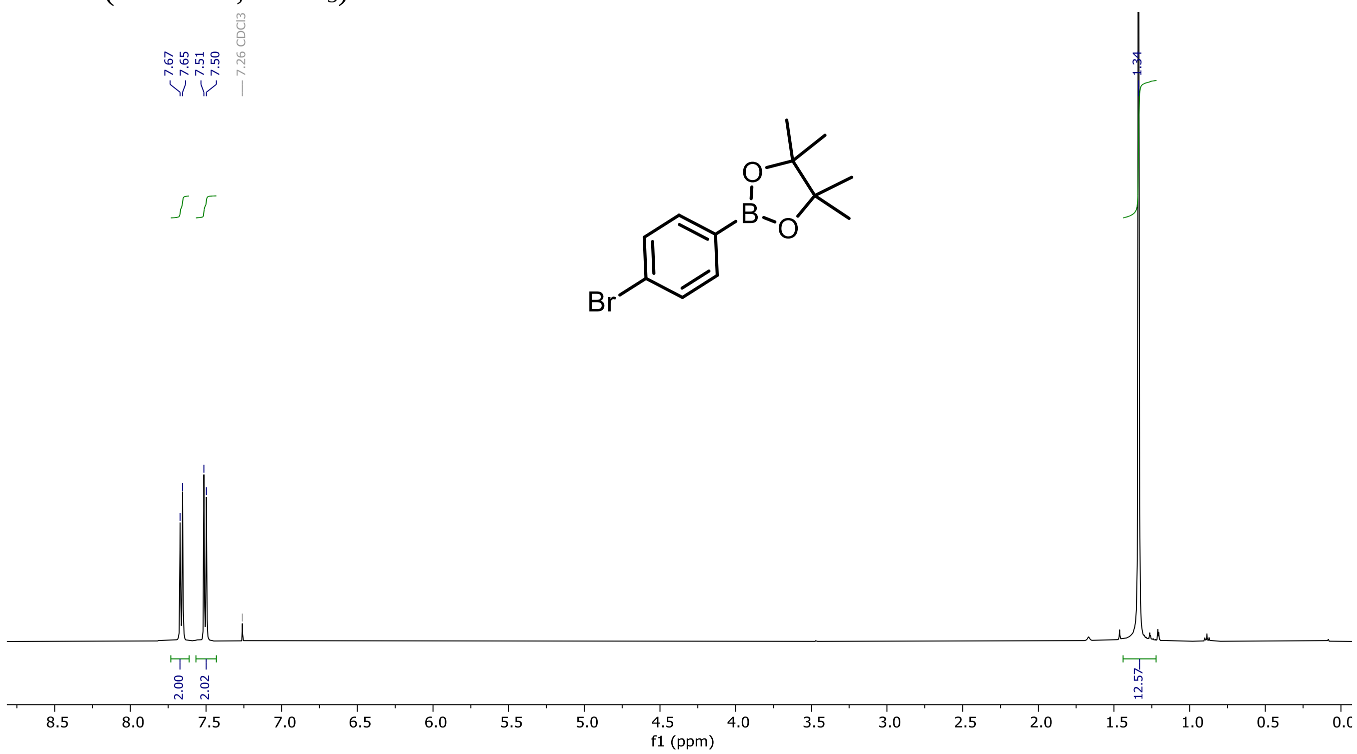

$^{13}C\{^{1}H\}$ NMR (126 MHz, $CDCl_3$)

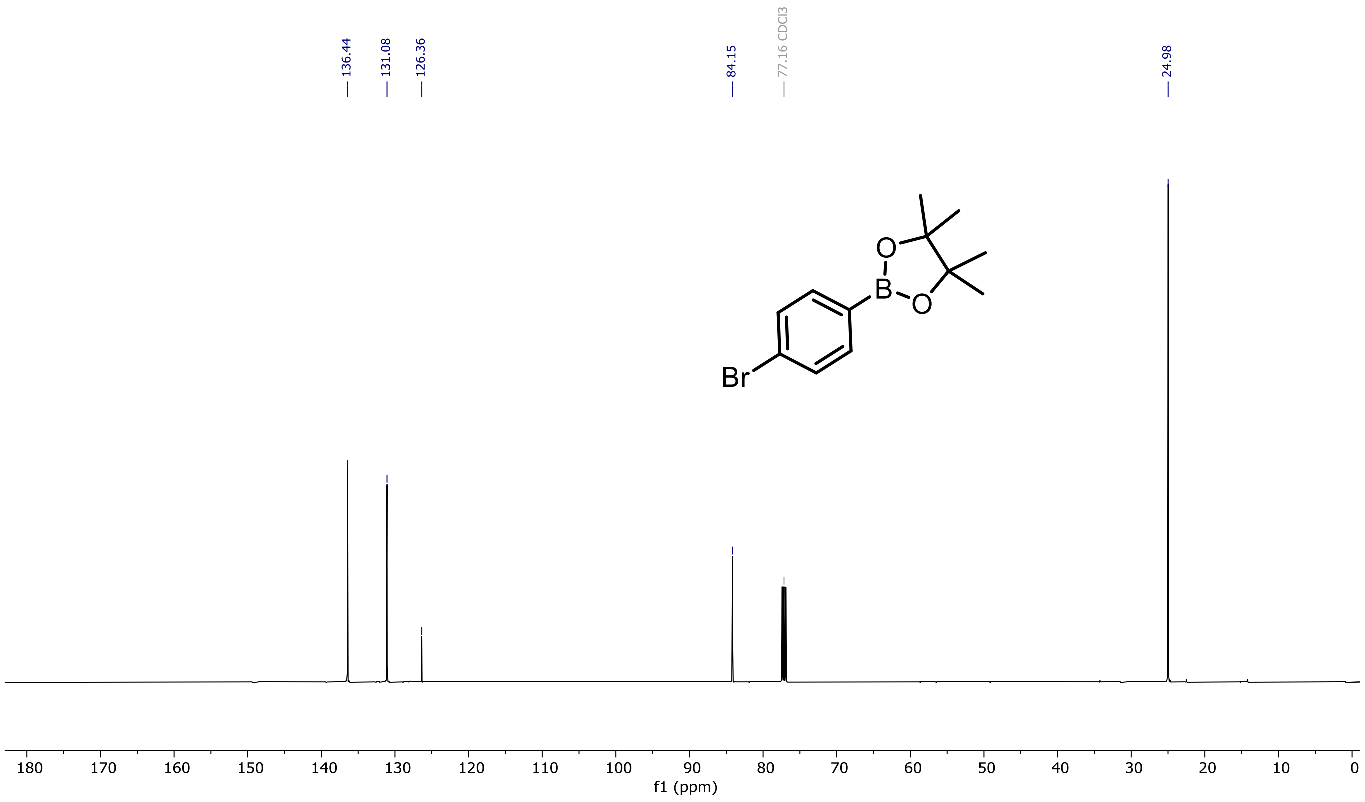

^{11}B NMR (160 MHz, $CDCl_3$)

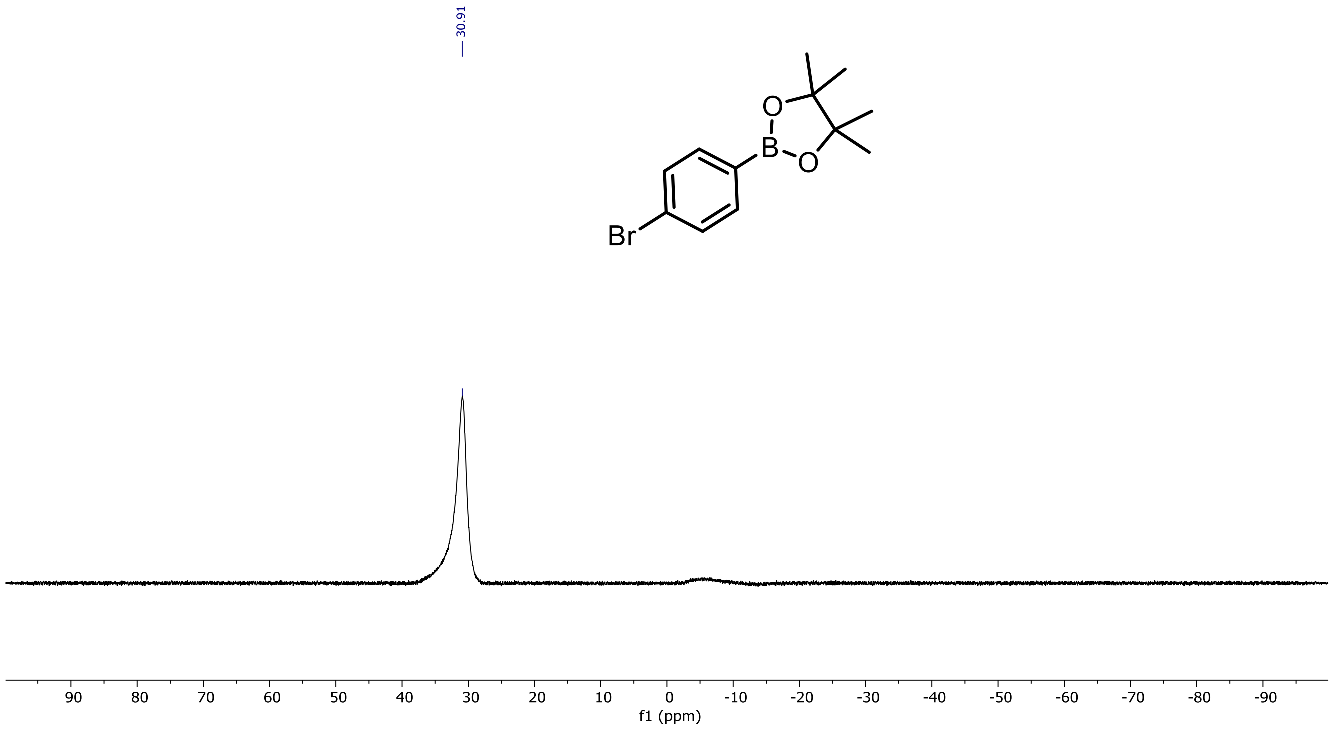

2-(4-Iodophenyl)-4,4,5,5-tetramethyl-1,3,2-dioxaborolane (6p)

^{1}H NMR (500 MHz, $CDCl_3$)

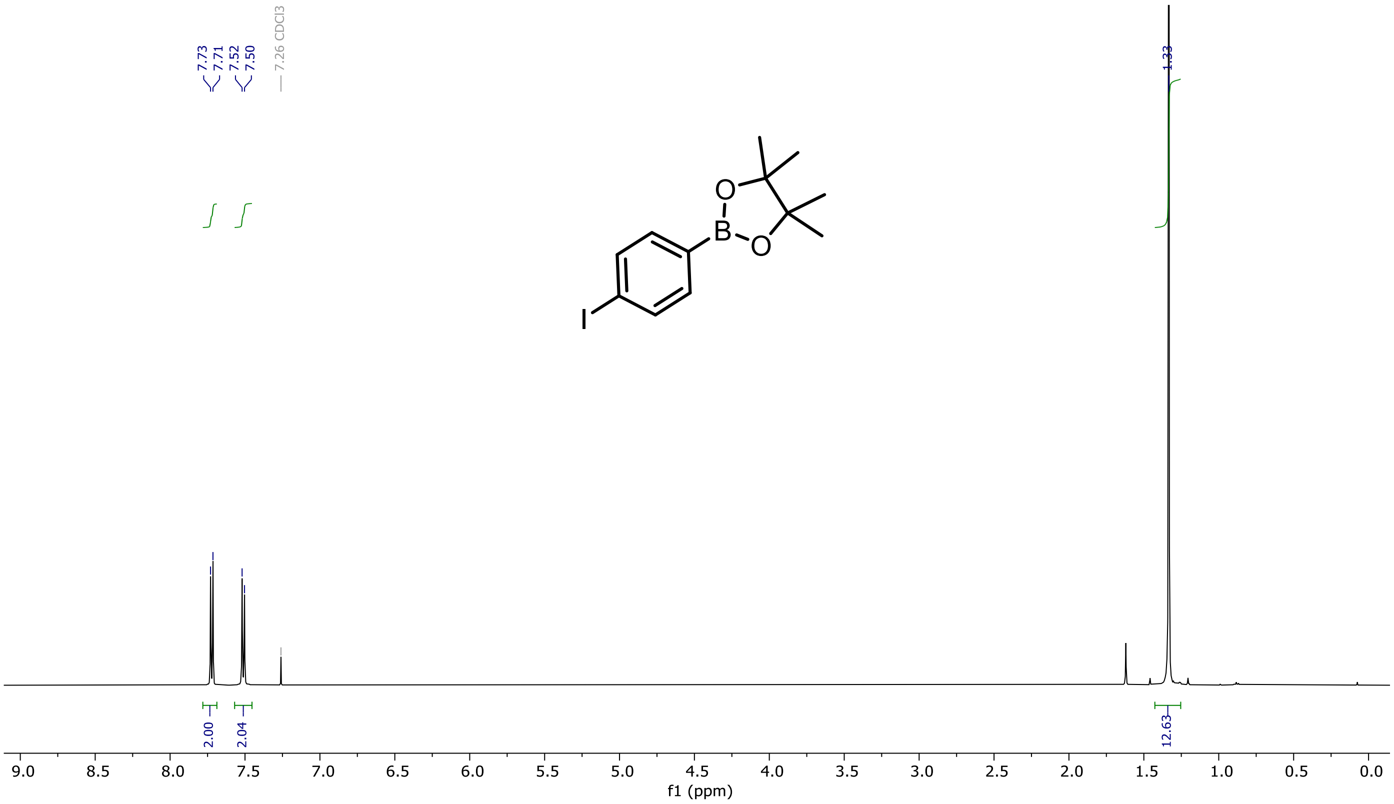

$^{13}C\{^{1}H\}$ NMR (126 MHz, $CDCl_3$)

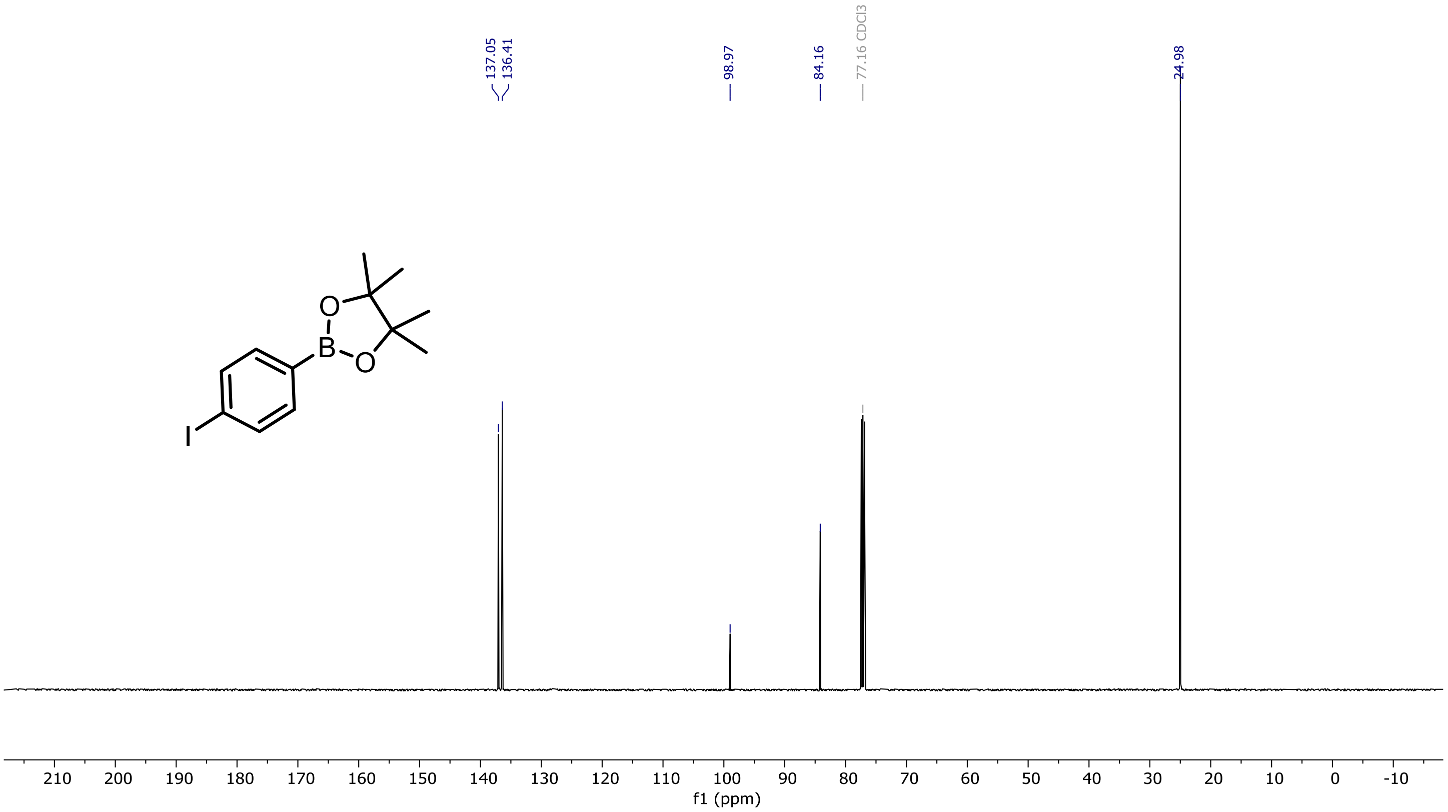

^{11}B NMR (160 MHz, $CDCl_3$)

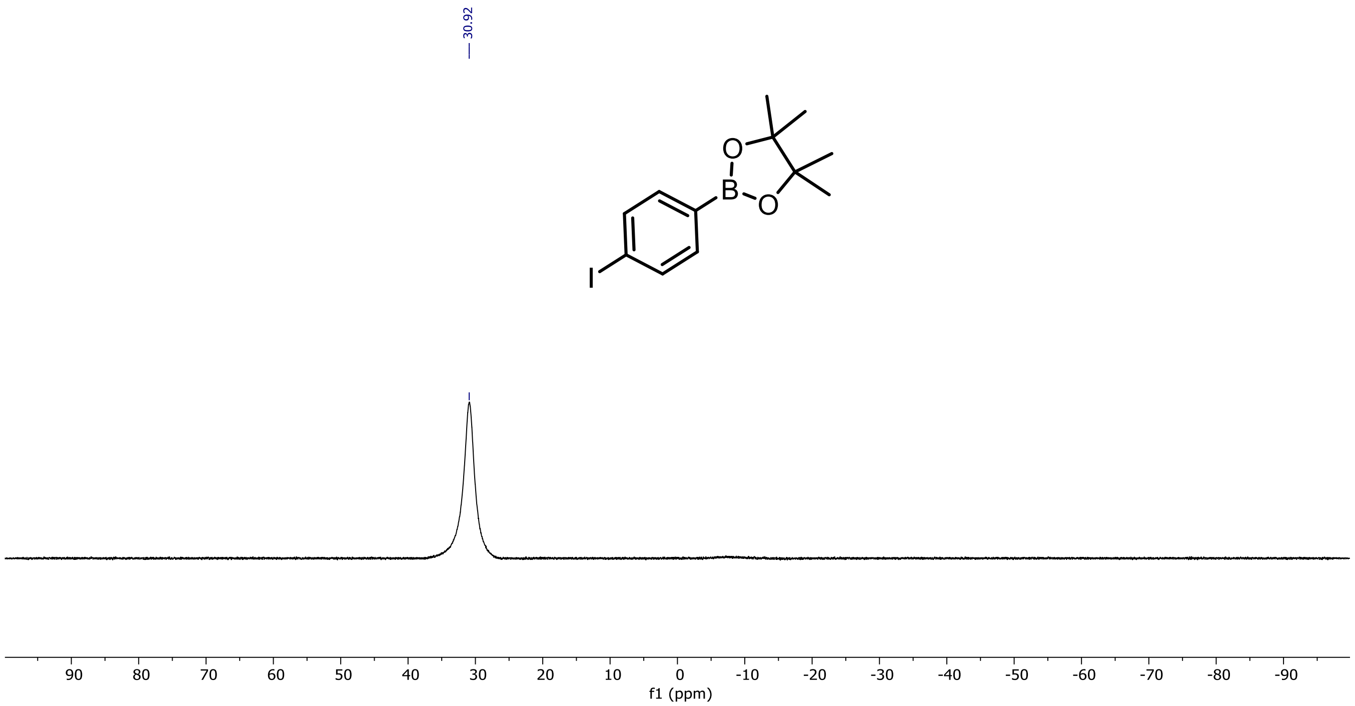

2-(3-Fluorophenyl)-4,4,5,5-tetramethyl-1,3,2-dioxaborolane (6q)

^{1}H NMR (500 MHz, $CDCl_3$)

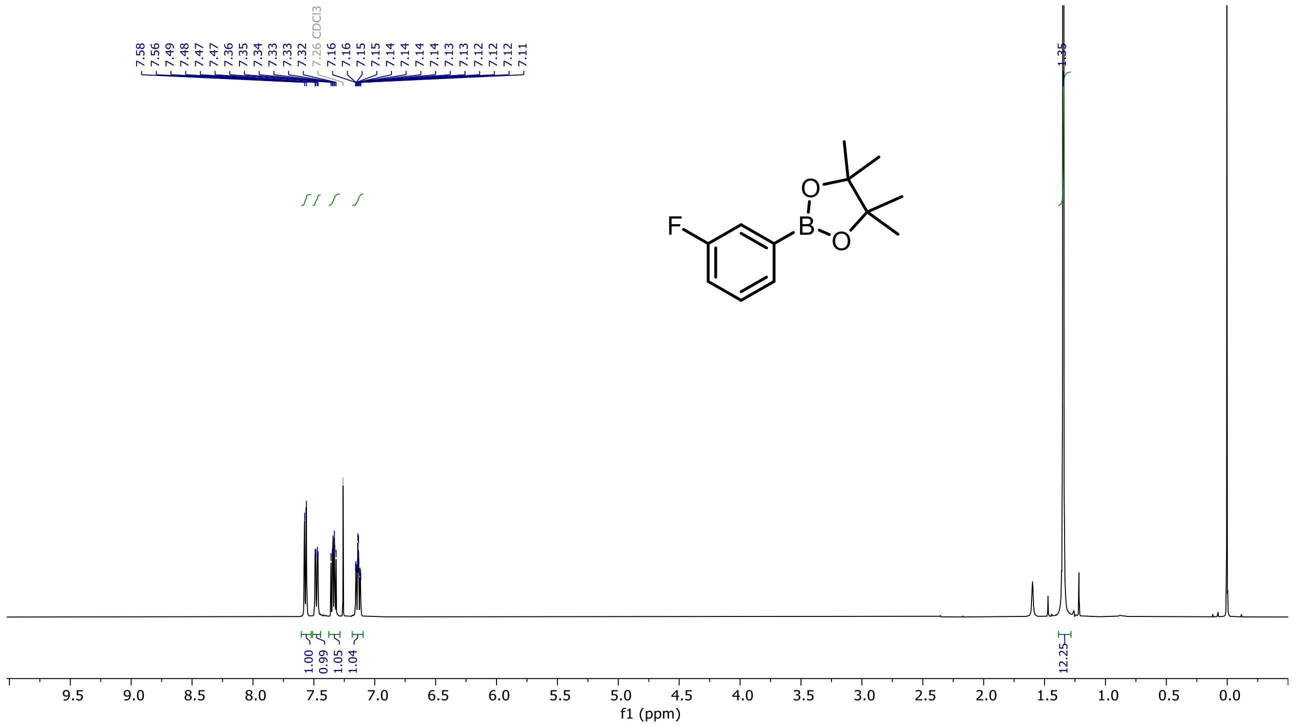

$^{13}C\{^{1}H\}$ NMR (126 MHz, $CDCl_3$)

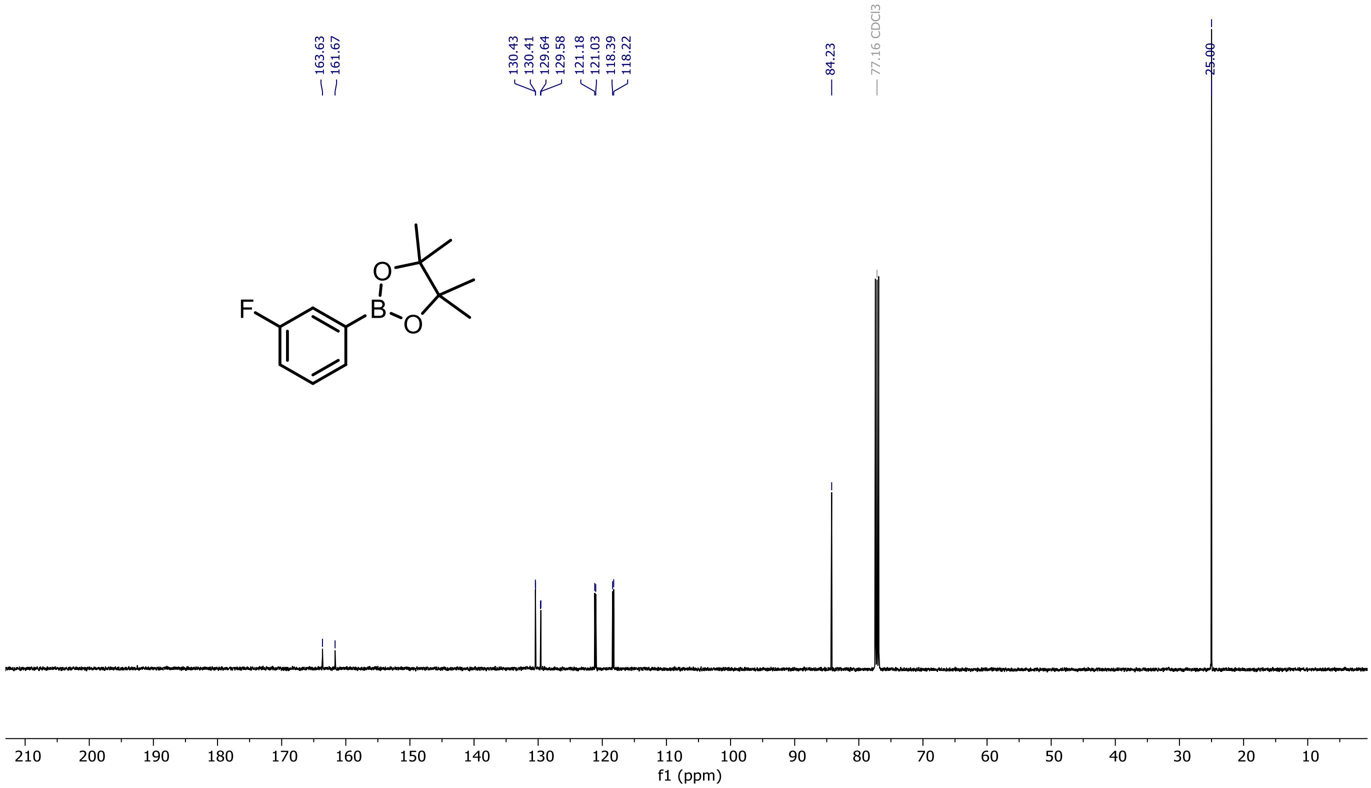

^{11}B NMR (160 MHz, $CDCl_3$)

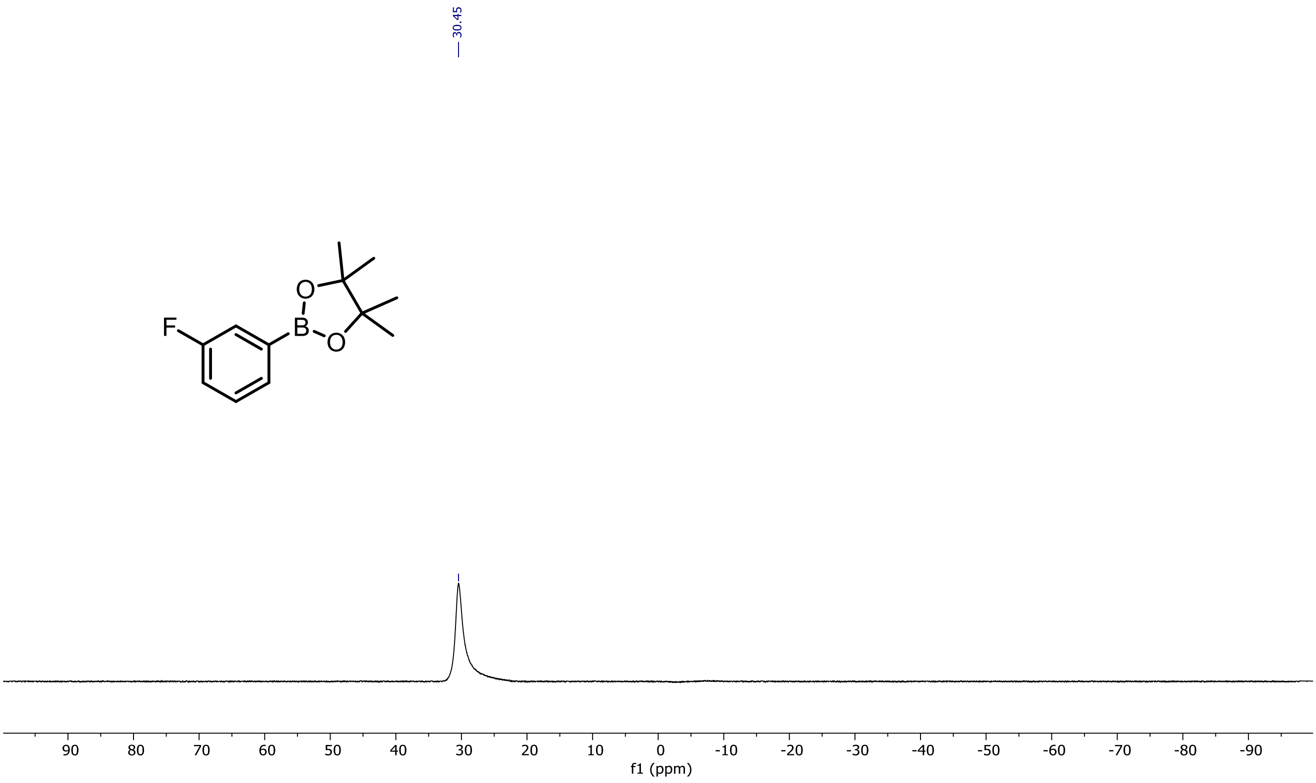

^{19}F NMR (470 MHz, $CDCl_3$)

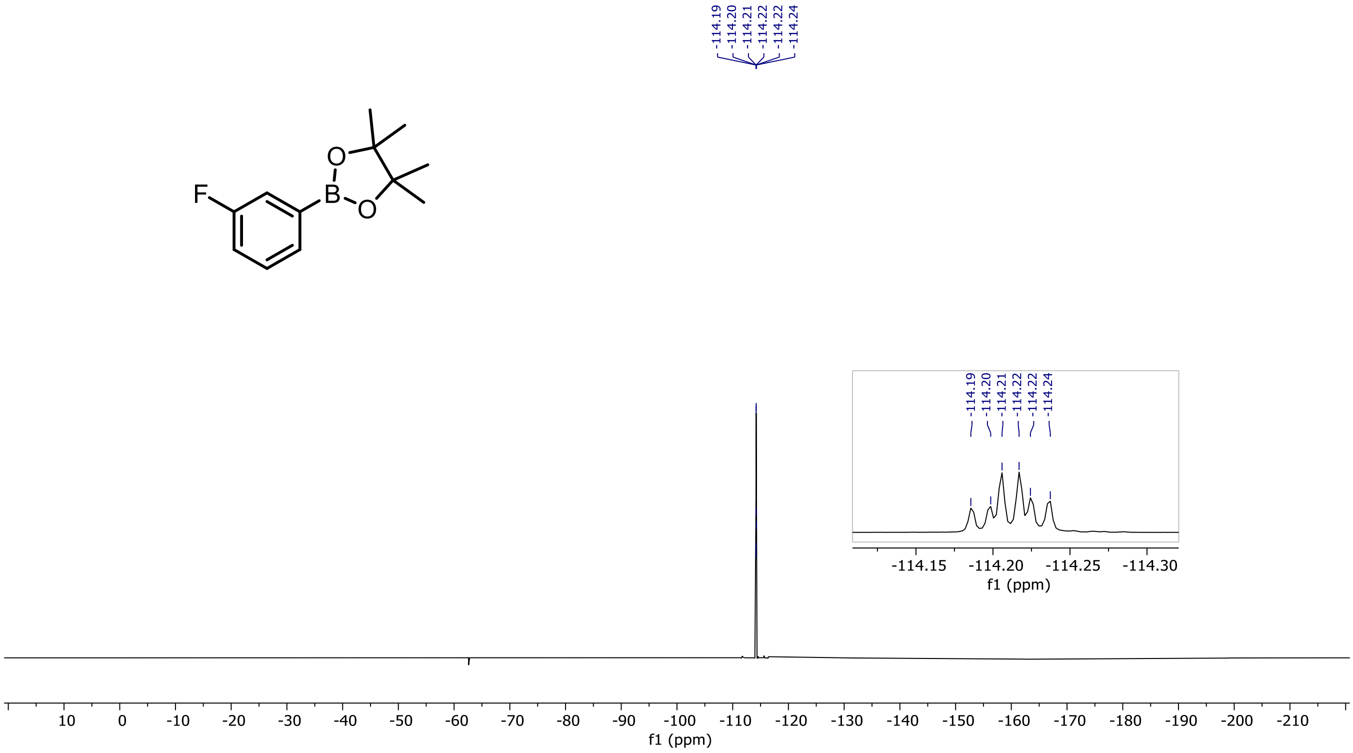

2-(2,4-Difluorophenyl)-4,4,5,5-tetramethyl-1,3,2-dioxaborolane (6r)

^{1}H NMR (500 MHz, $CDCl_3$)

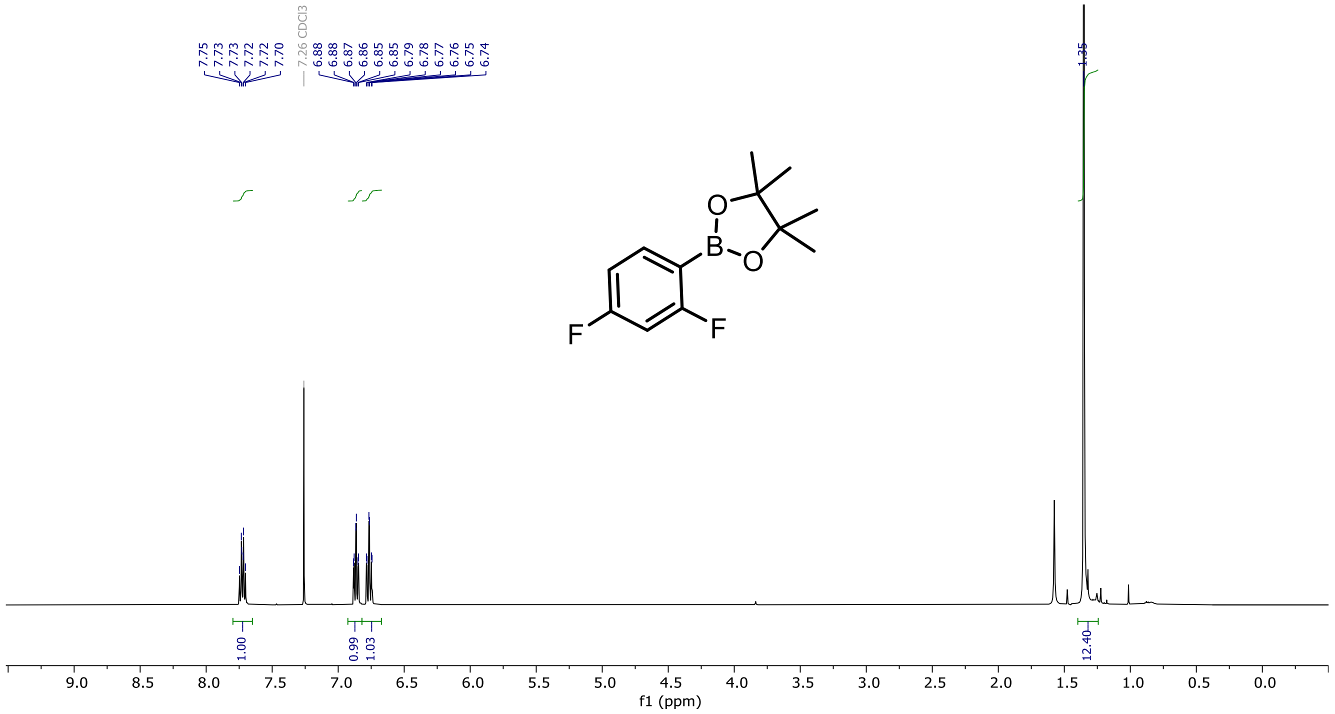

$^{13}C\{^{1}H\}$ NMR (126 MHz, $CDCl_3$)

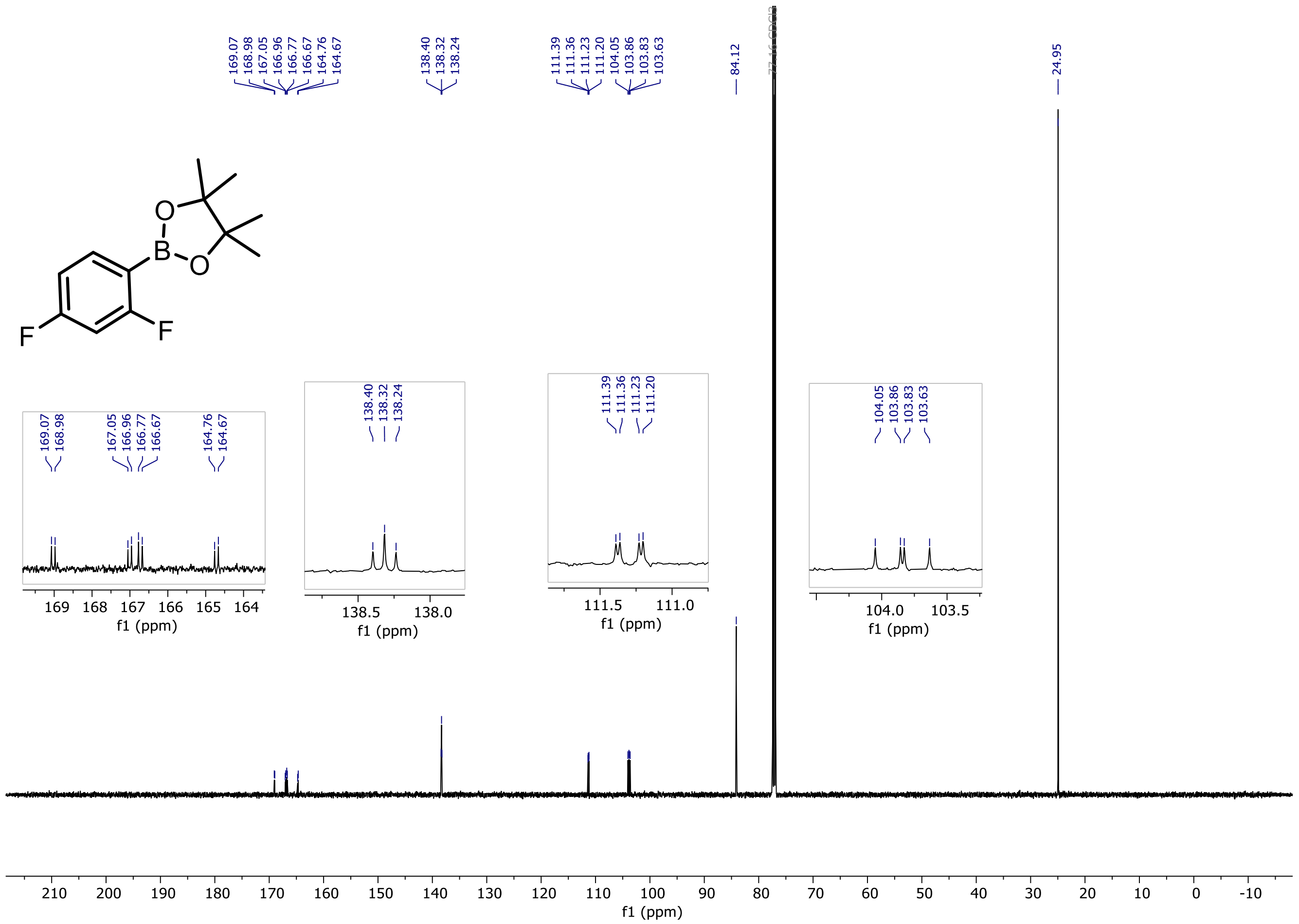

^{11}B NMR (160 MHz, $CDCl_3$)

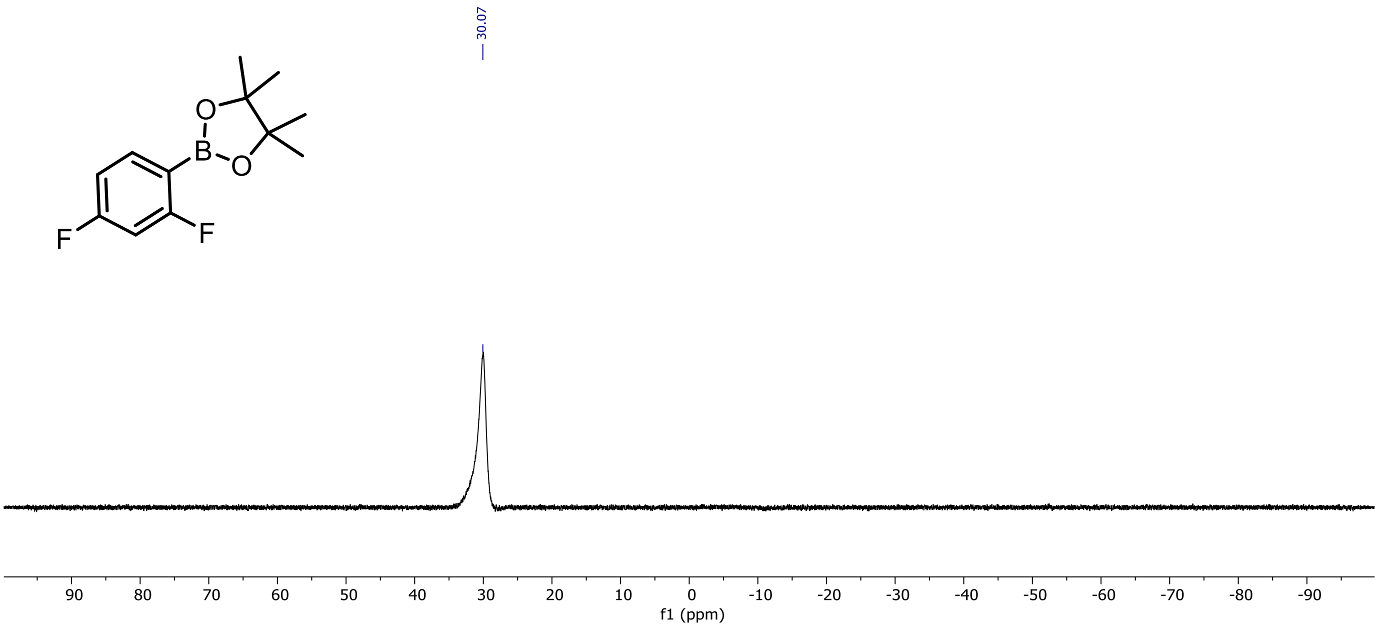

^{19}F NMR (470 MHz, $CDCl_3$)

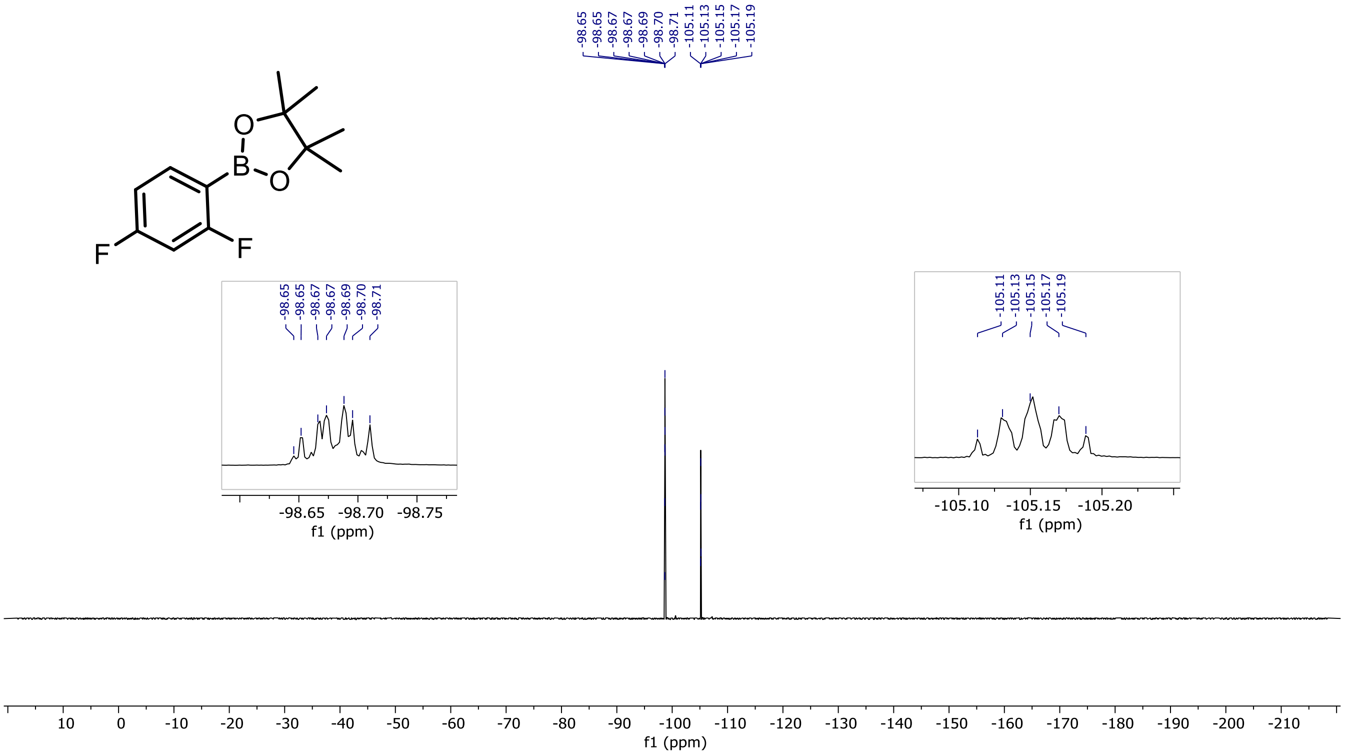

Methyl 4-(4,4,5,5-tetramethyl-1,3,2-dioxaborolan-2-yl)benzoate (6s)

^{1}H NMR (500 MHz, $CDCl_3$)

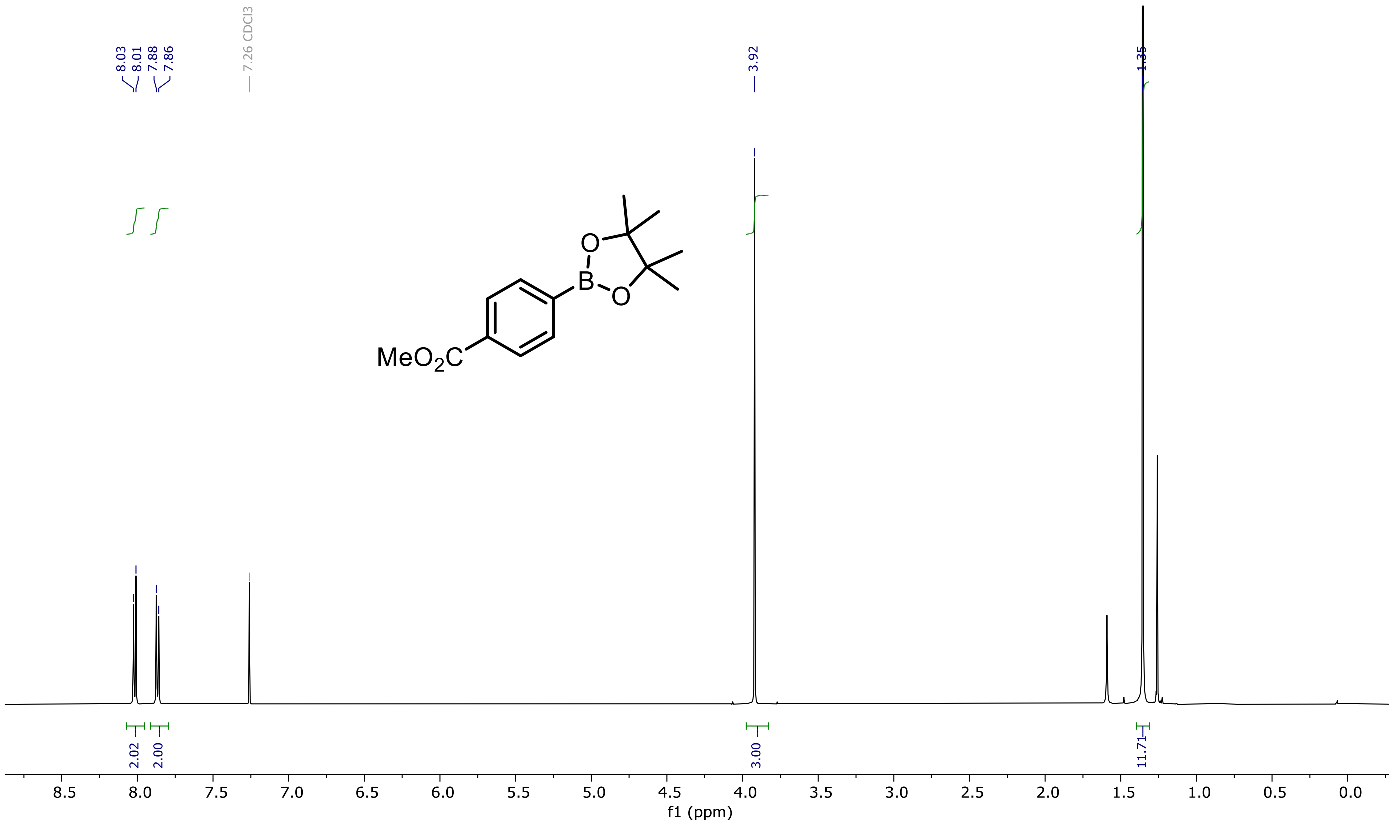

^{13}C NMR (126 MHz, $CDCl_3$)

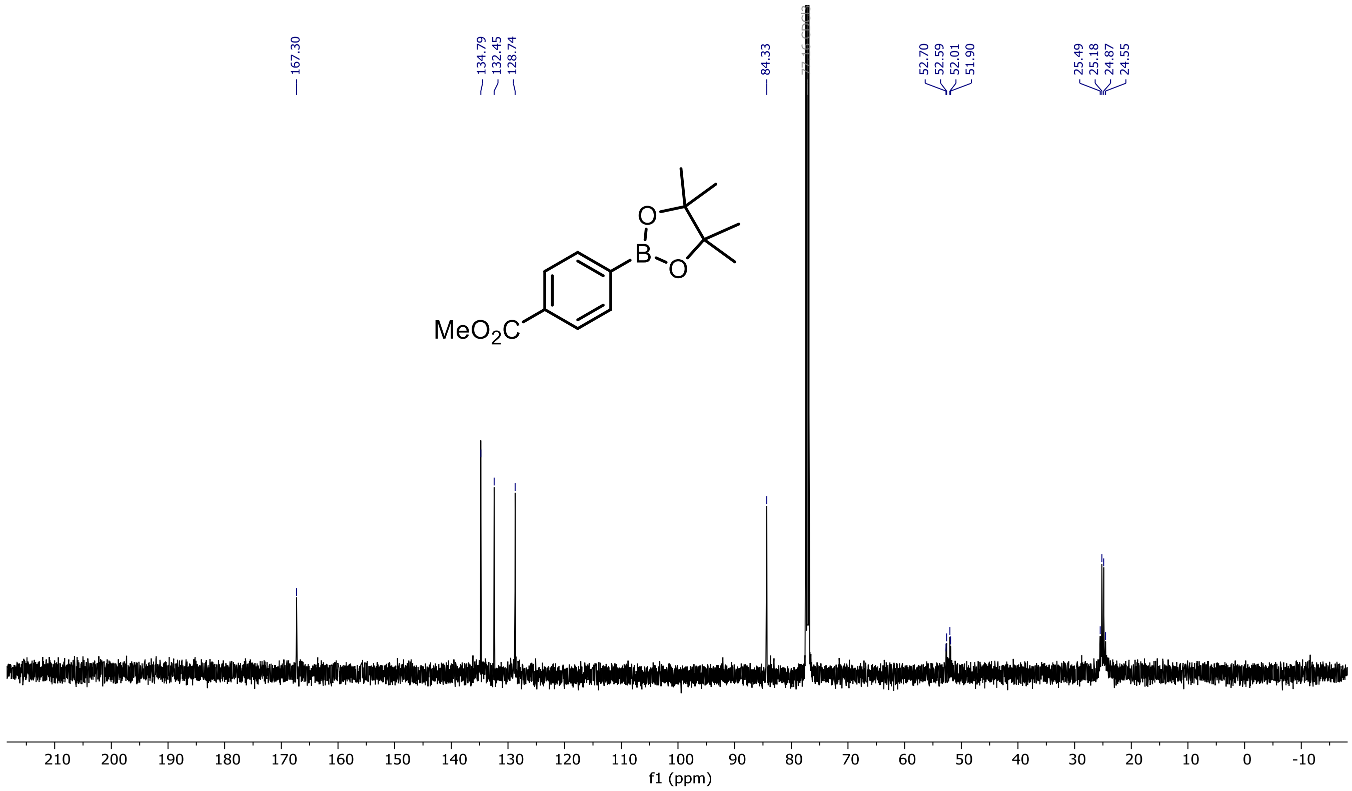

^{11}B NMR (160 MHz, $CDCl_3$)

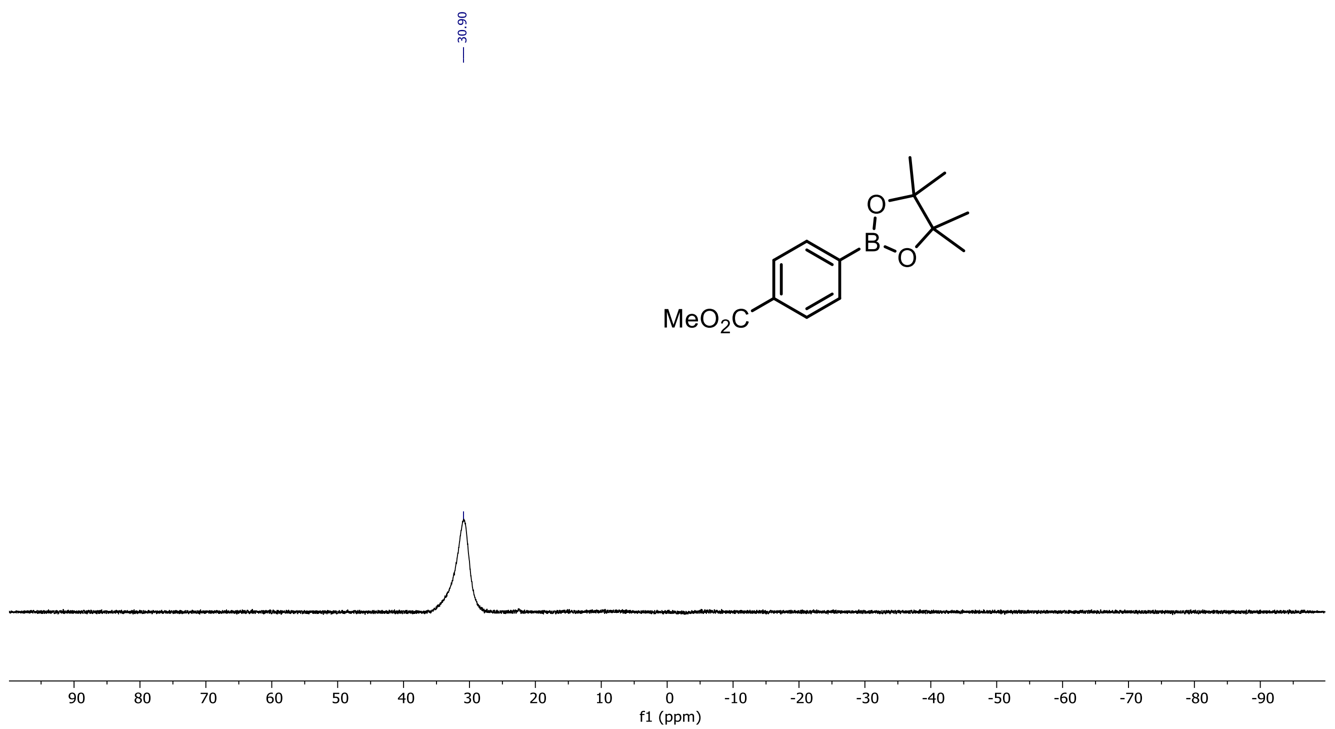

Ethyl 4-(4,4,5,5-tetramethyl-1,3,2-dioxaborolan-2-yl)benzoate (6t)

^{1}H NMR (500 MHz, $CDCl_3$)

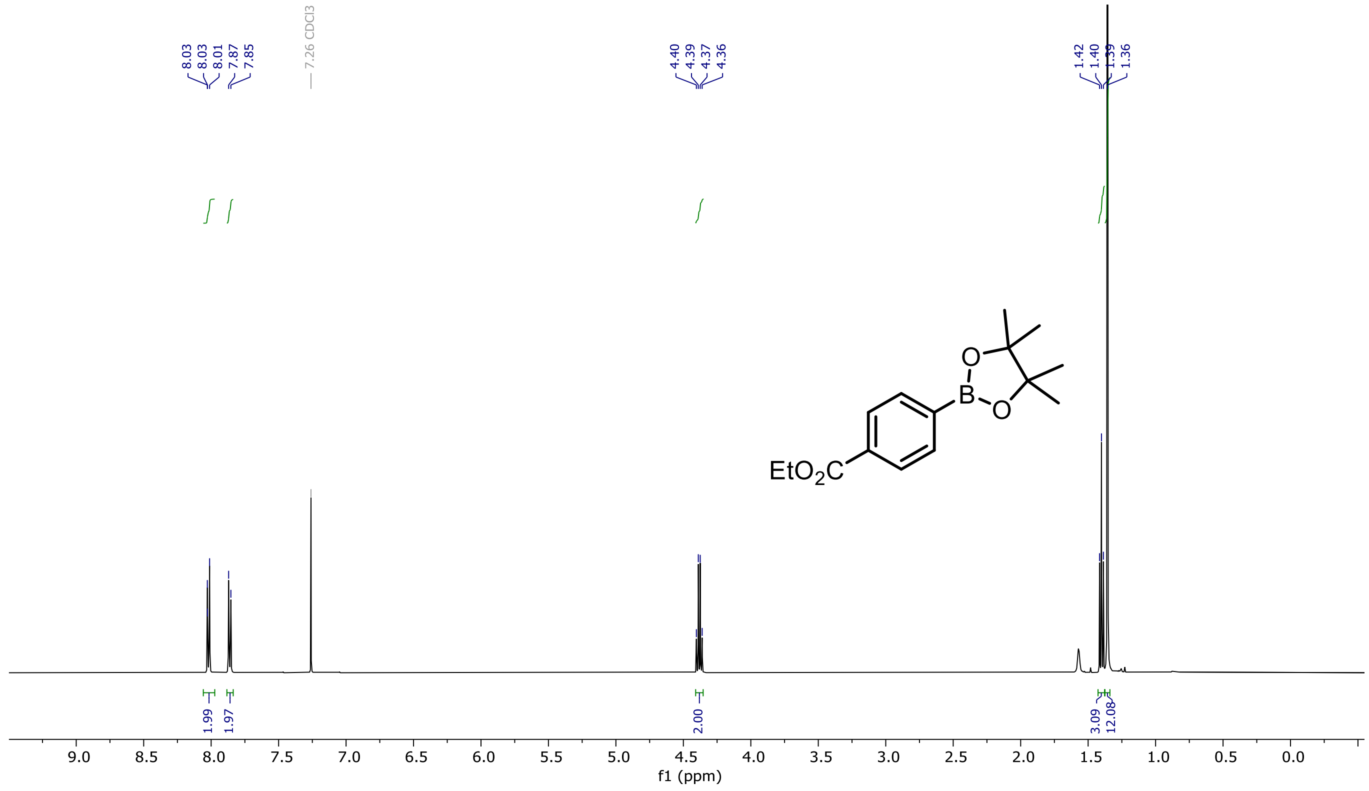

$^{13}C\{^{1}H\}$ NMR (126 MHz, $CDCl_3$)

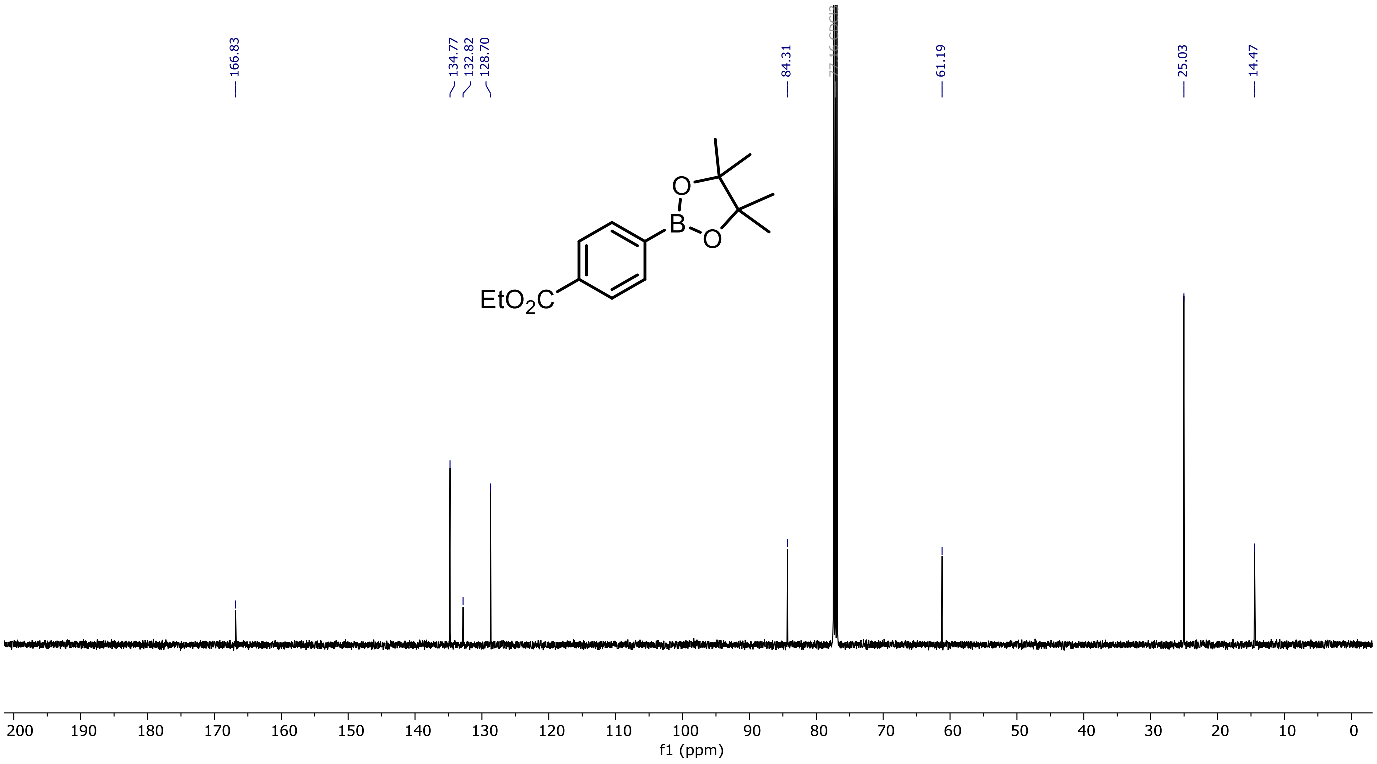

^{11}B NMR (160 MHz, $CDCl_3$)

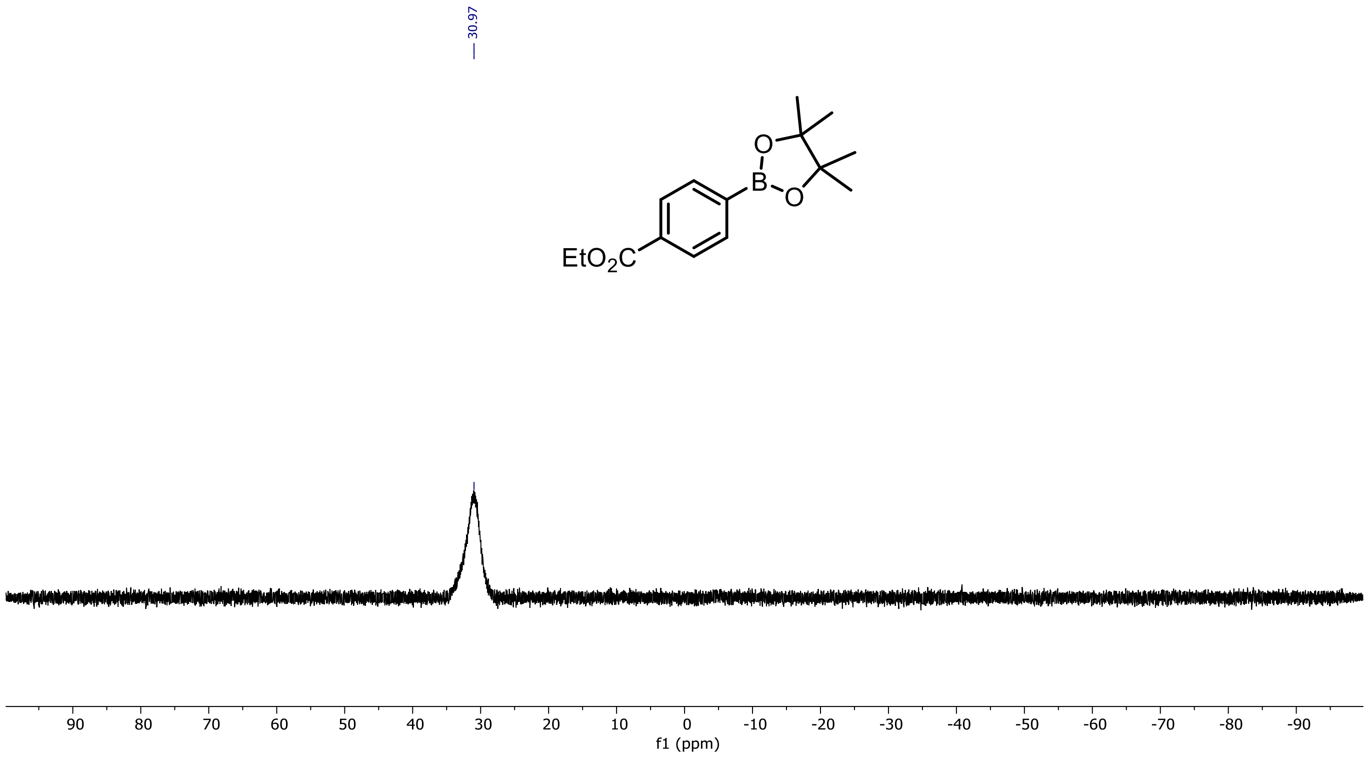

4,4,5,5-Tetramethyl-2-(4-(trifluoromethyl)phenyl)-1,3,2-dioxaborolane (6u)

^{1}H NMR (500 MHz, $CDCl_3$)

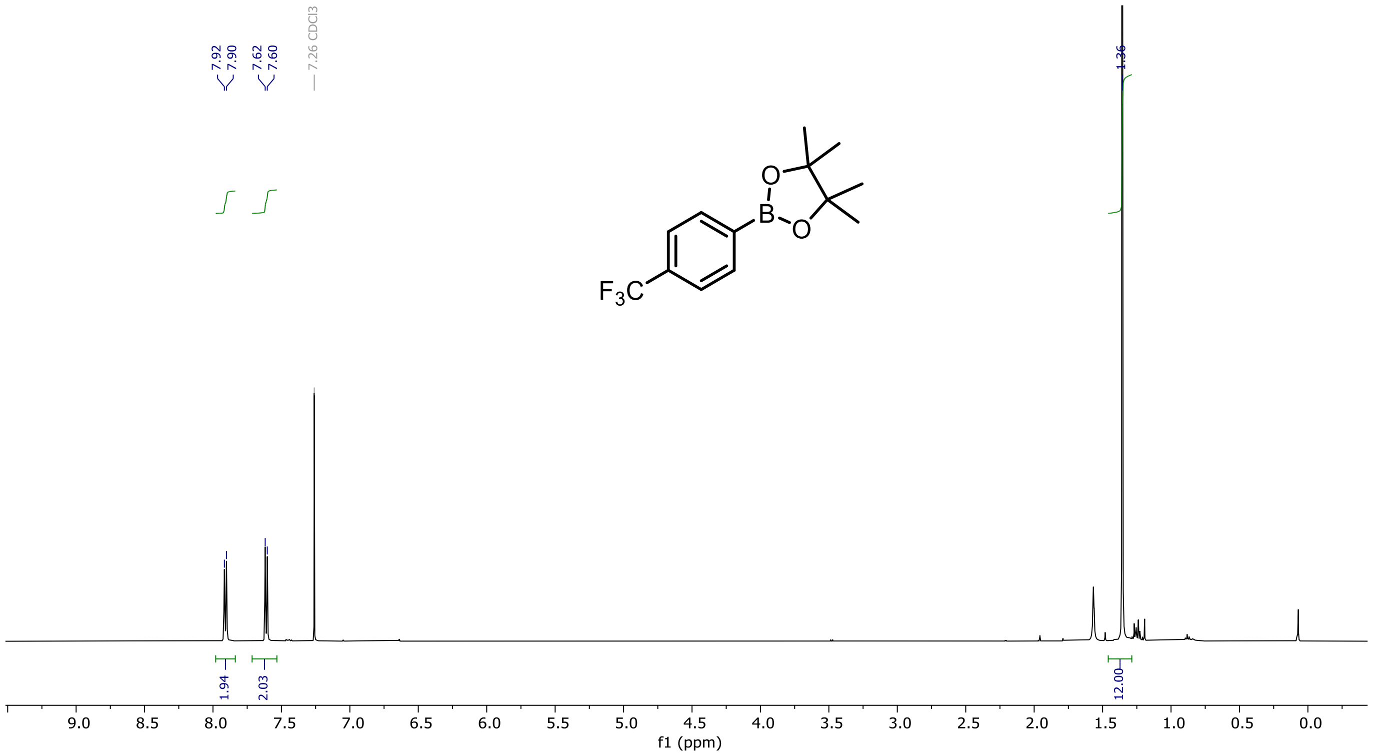

^{19}F NMR (470 MHz, $CDCl_3$)

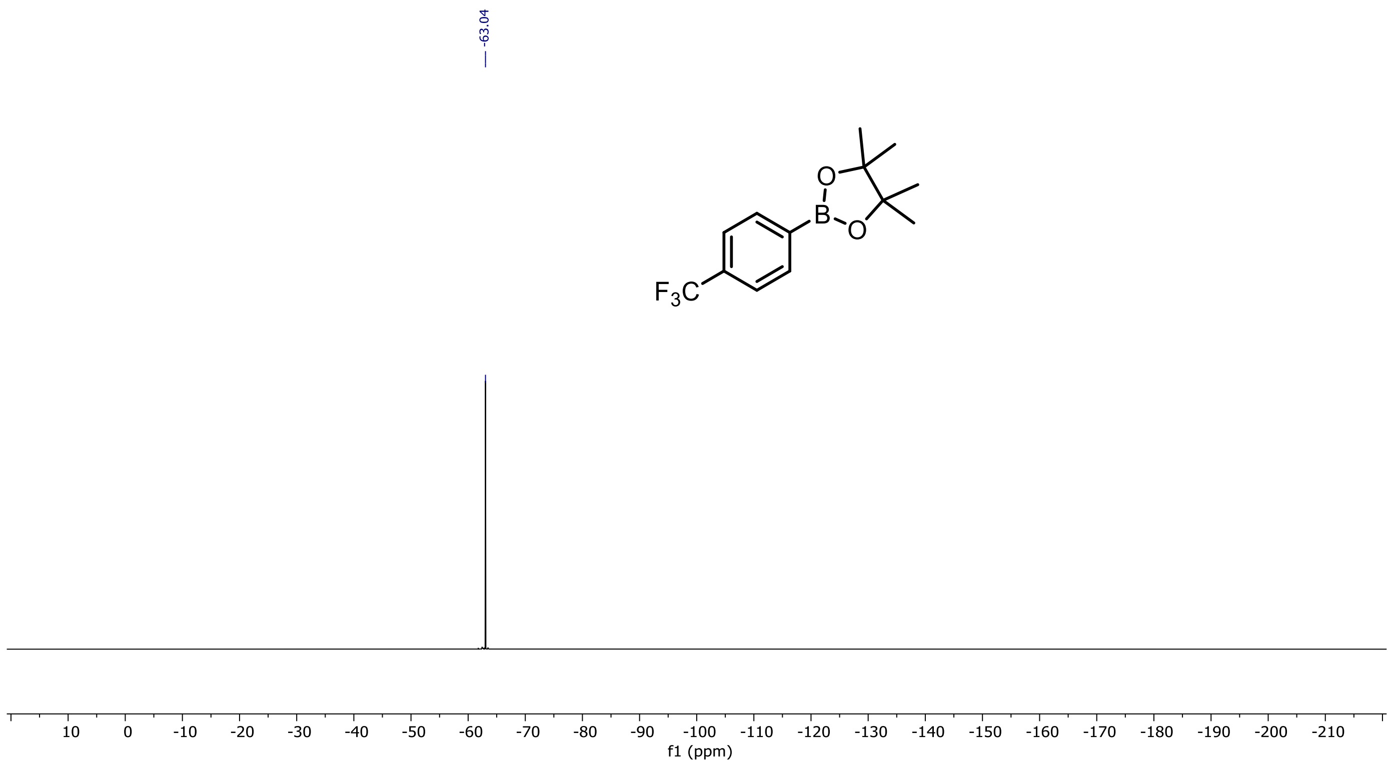

^{11}B NMR (160 MHz, $CDCl_3$)

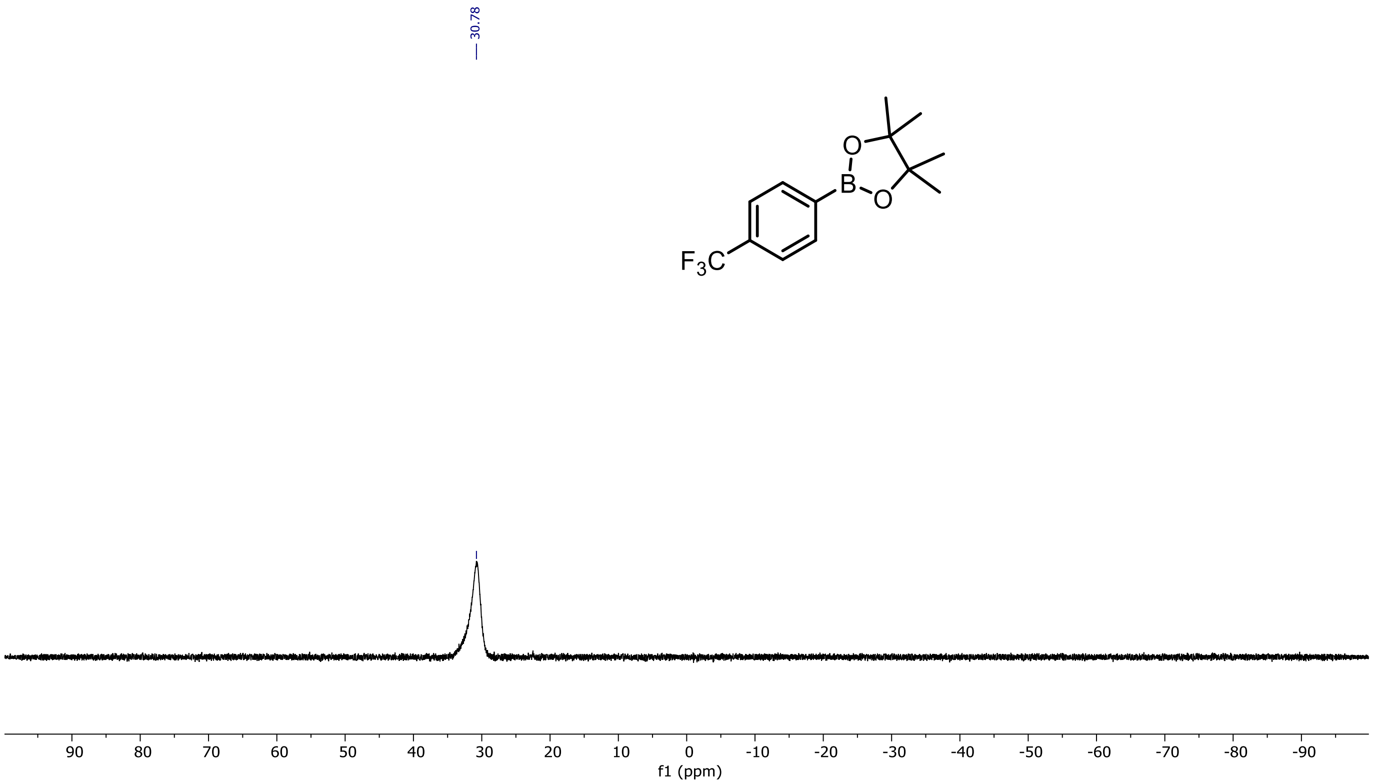

^{13}C{^{1}H} NMR (126 MHz, $CDCl_3$)

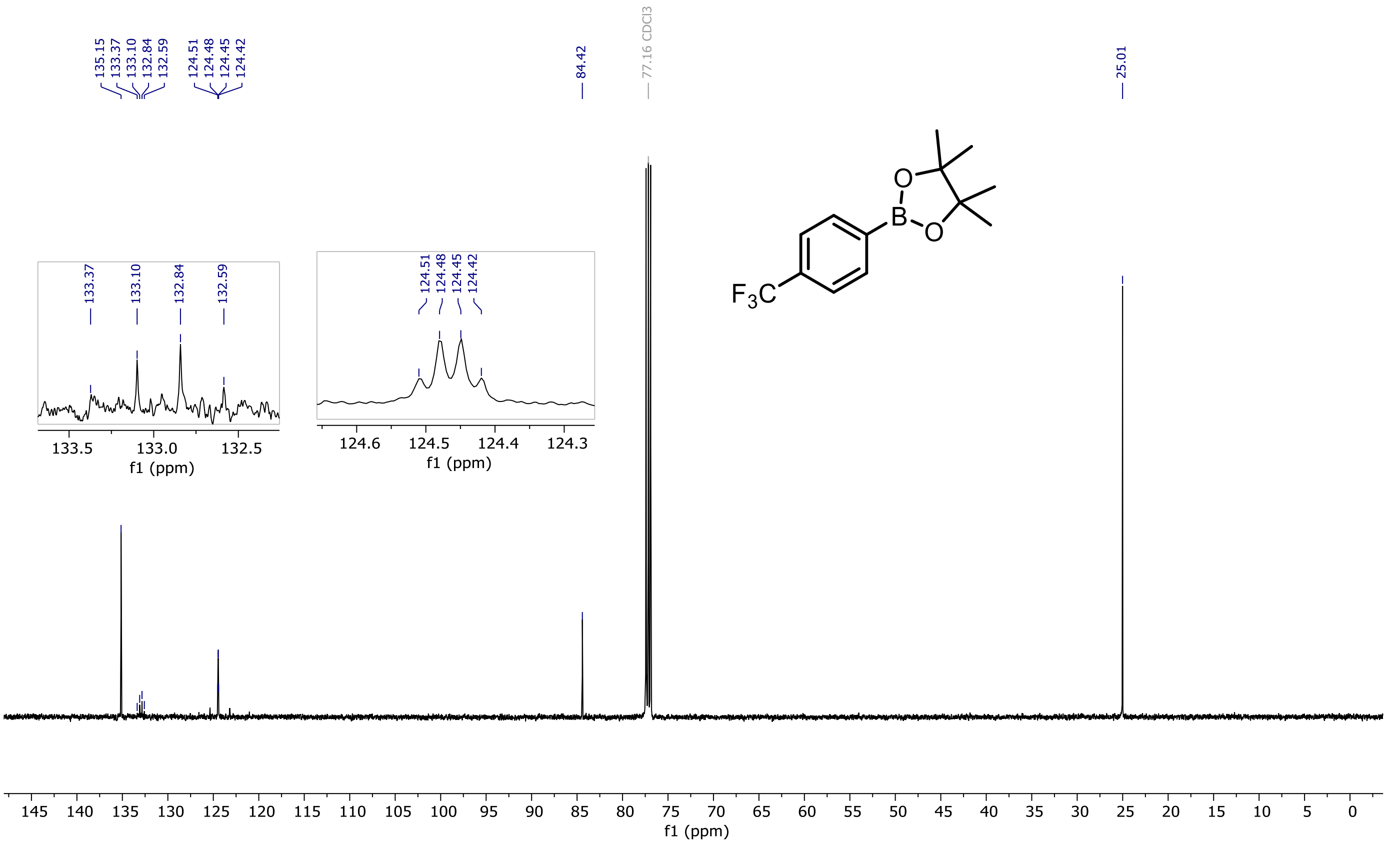

4,4,5,5-Tetramethyl-2-(4-nitrophenyl)-1,3,2-dioxaborolane (6v)

^{1}H NMR (500 MHz, $CDCl_3$)

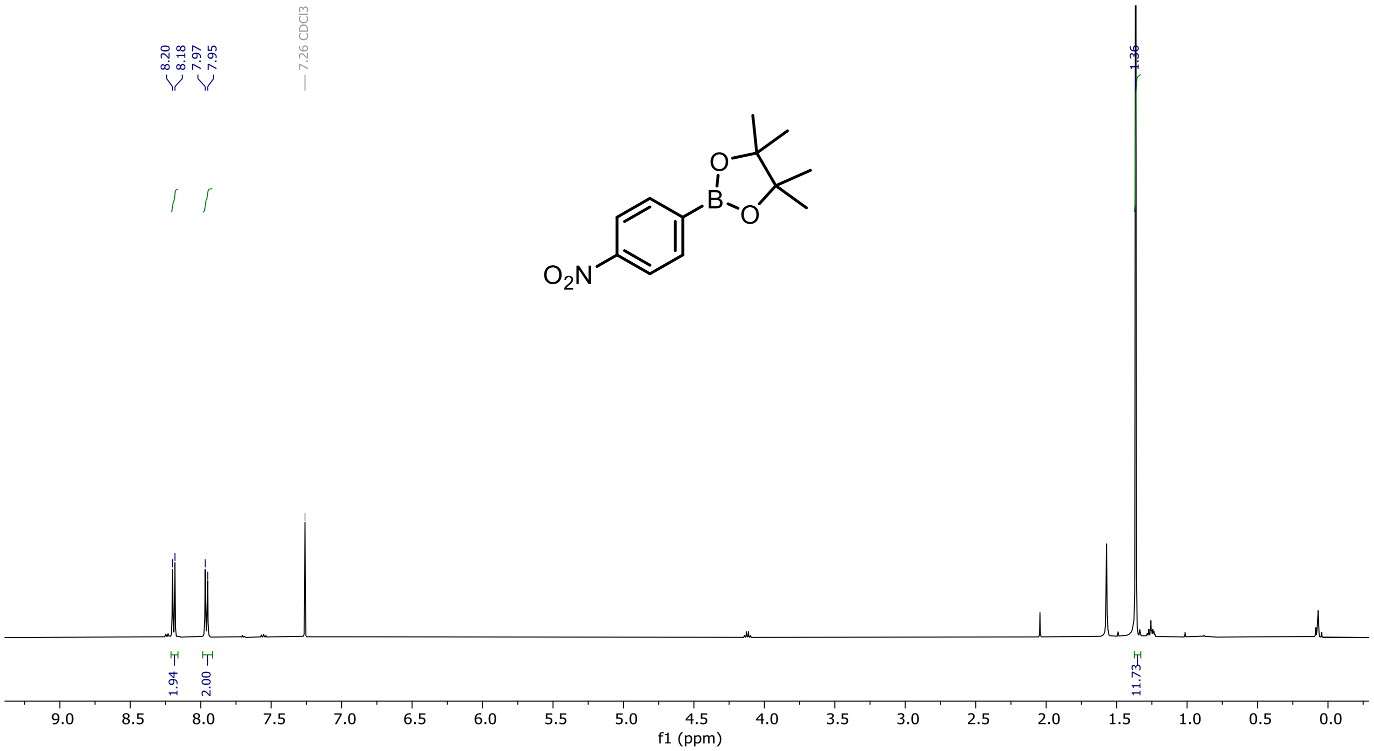

$^{13}C\{^{1}H\}$ NMR (126 MHz, $CDCl_3$)

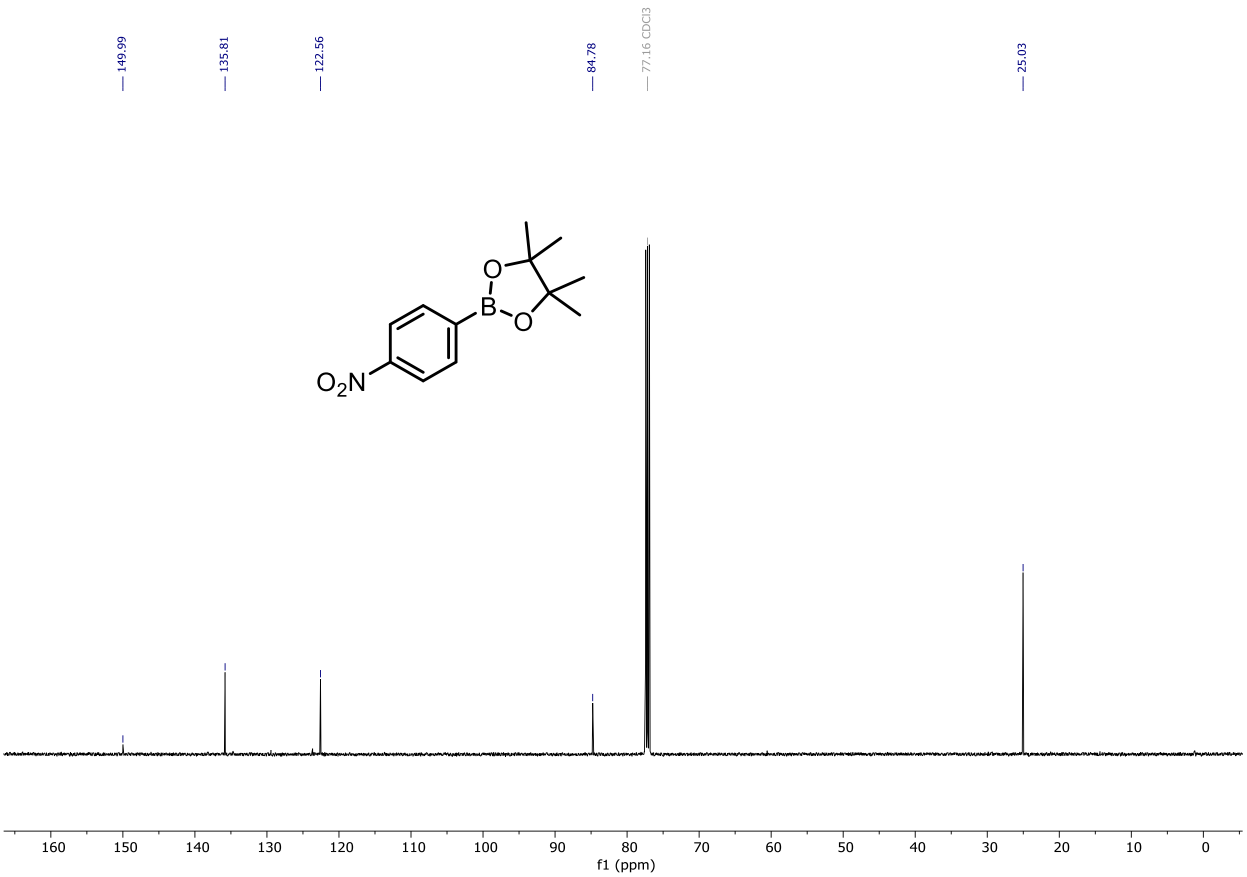

^{11}B NMR (160 MHz, $CDCl_3$)

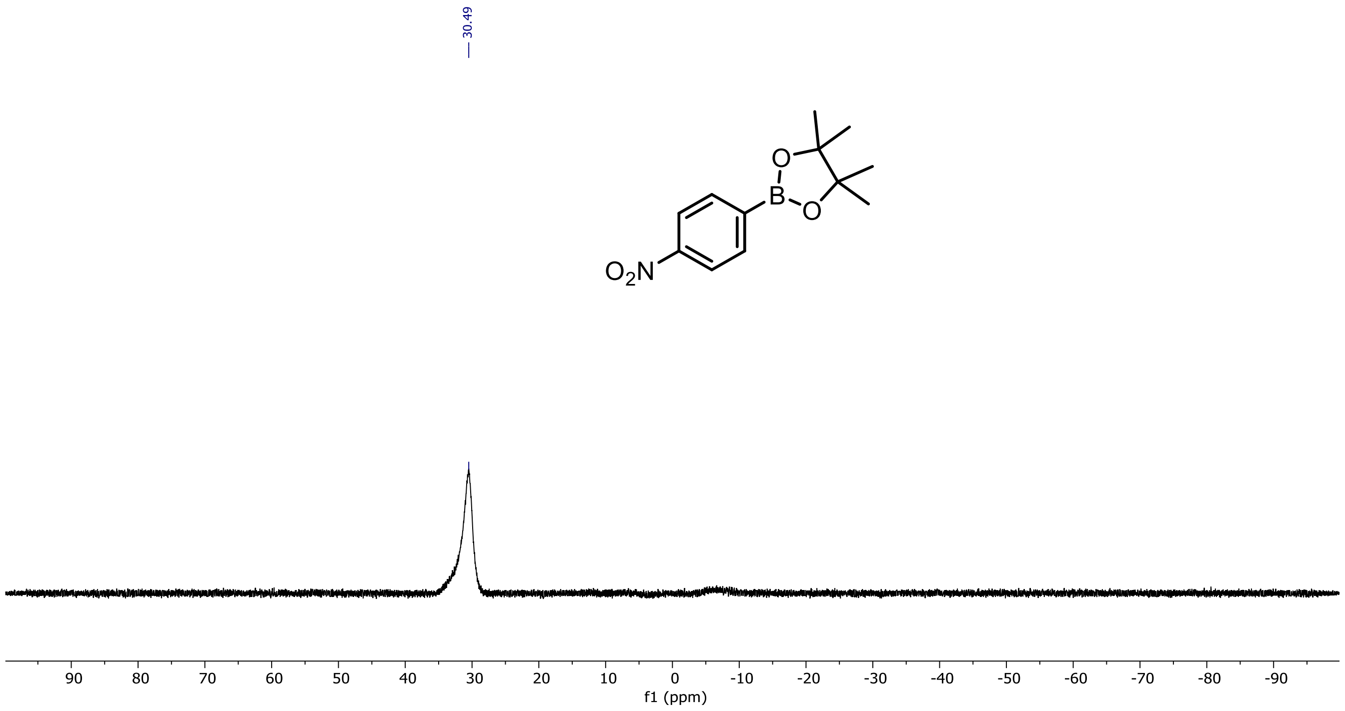

2-(4,4,5,5-Tetramethyl-1,3,2-dioxaborolan-2-yl)benzonitrile (6w)

^{1}H NMR (500 MHz, $CDCl_3$)

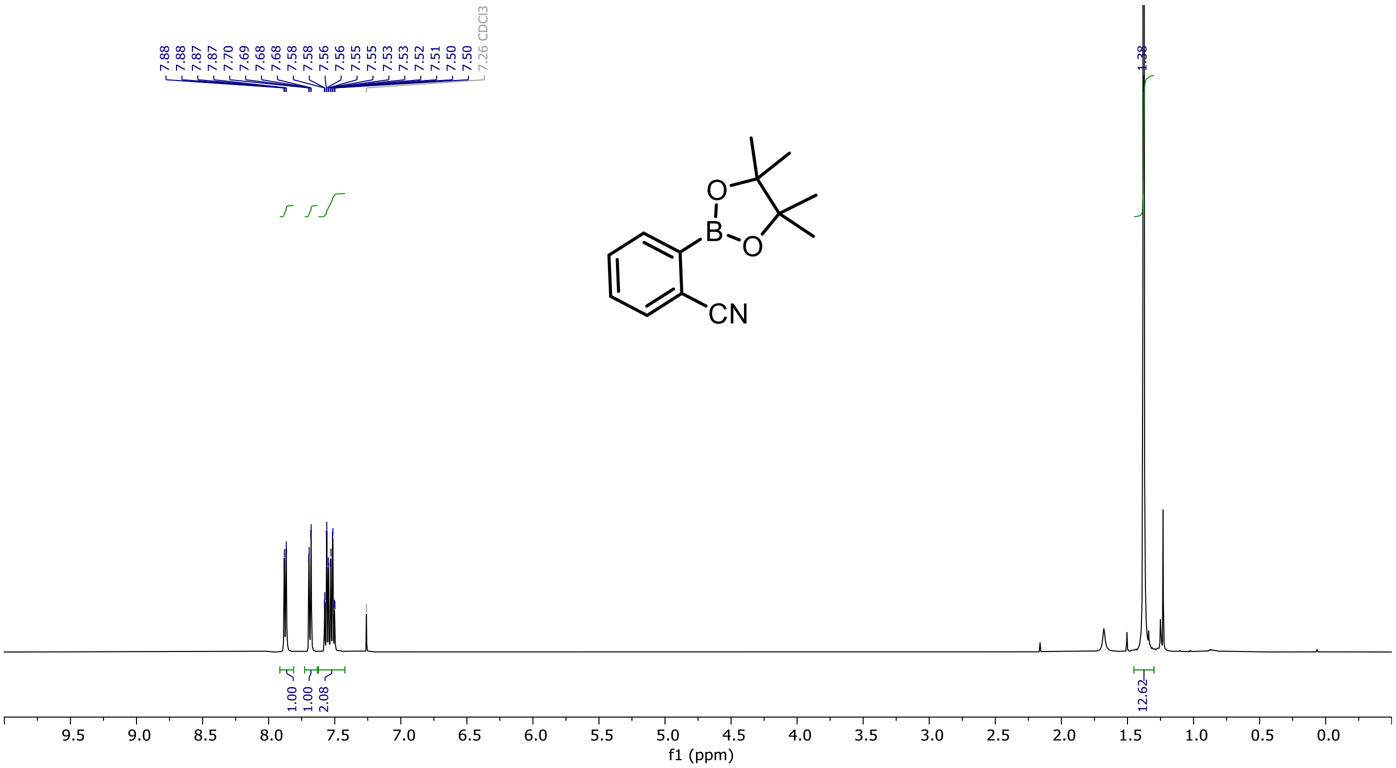

$^{13}C\{^{1}H\}$ NMR (126 MHz, $CDCl_3$)

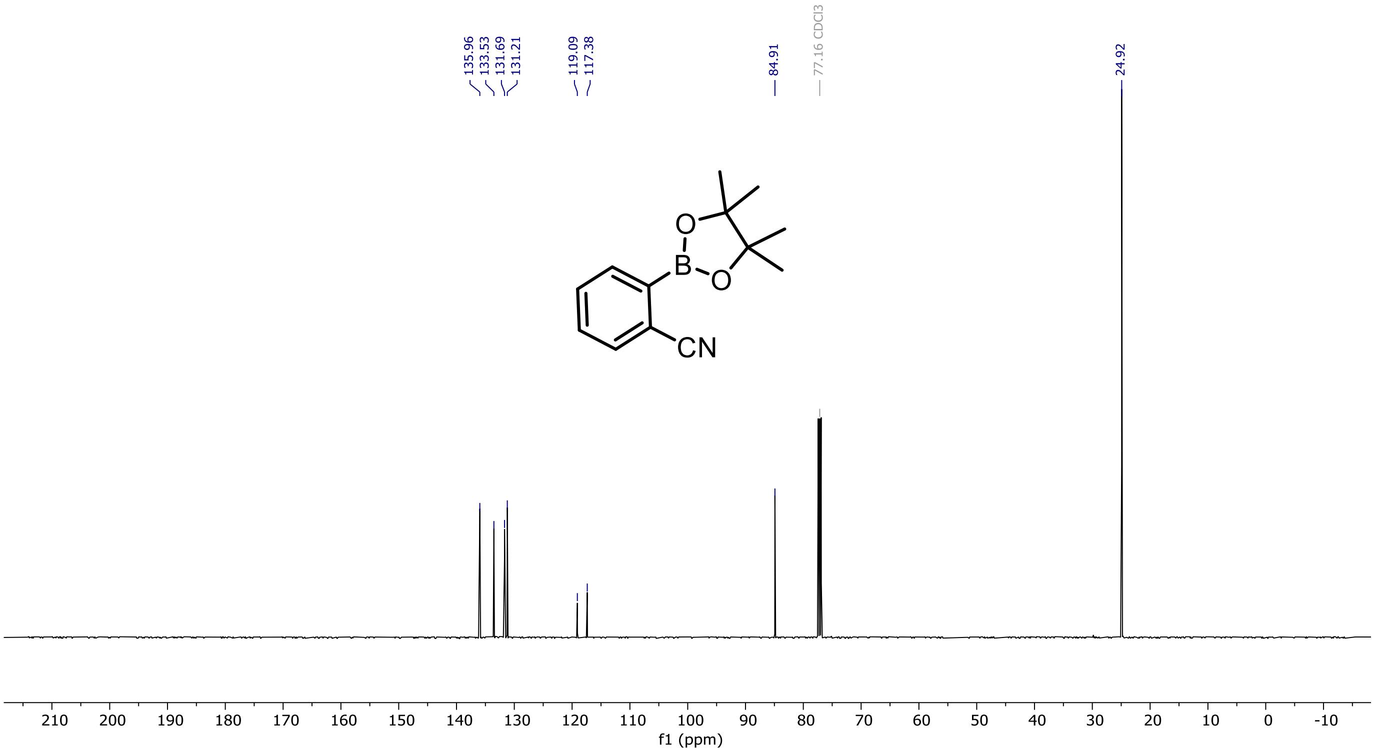

^{11}B NMR (160 MHz, $CDCl_3$)

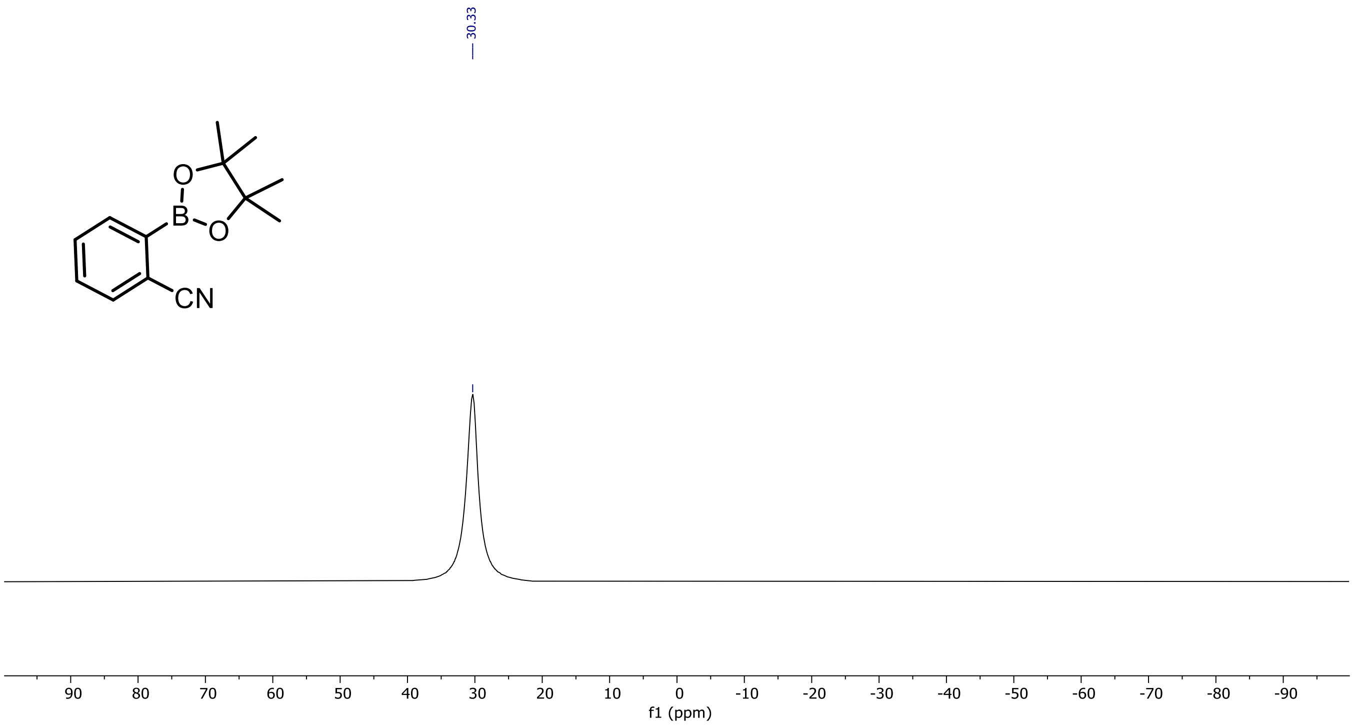

4-(4,4,5,5-Tetramethyl-1,3,2-dioxaborolan-2-yl)benzonitrile (6x)

^{1}H NMR (500 MHz, $CDCl_3$)

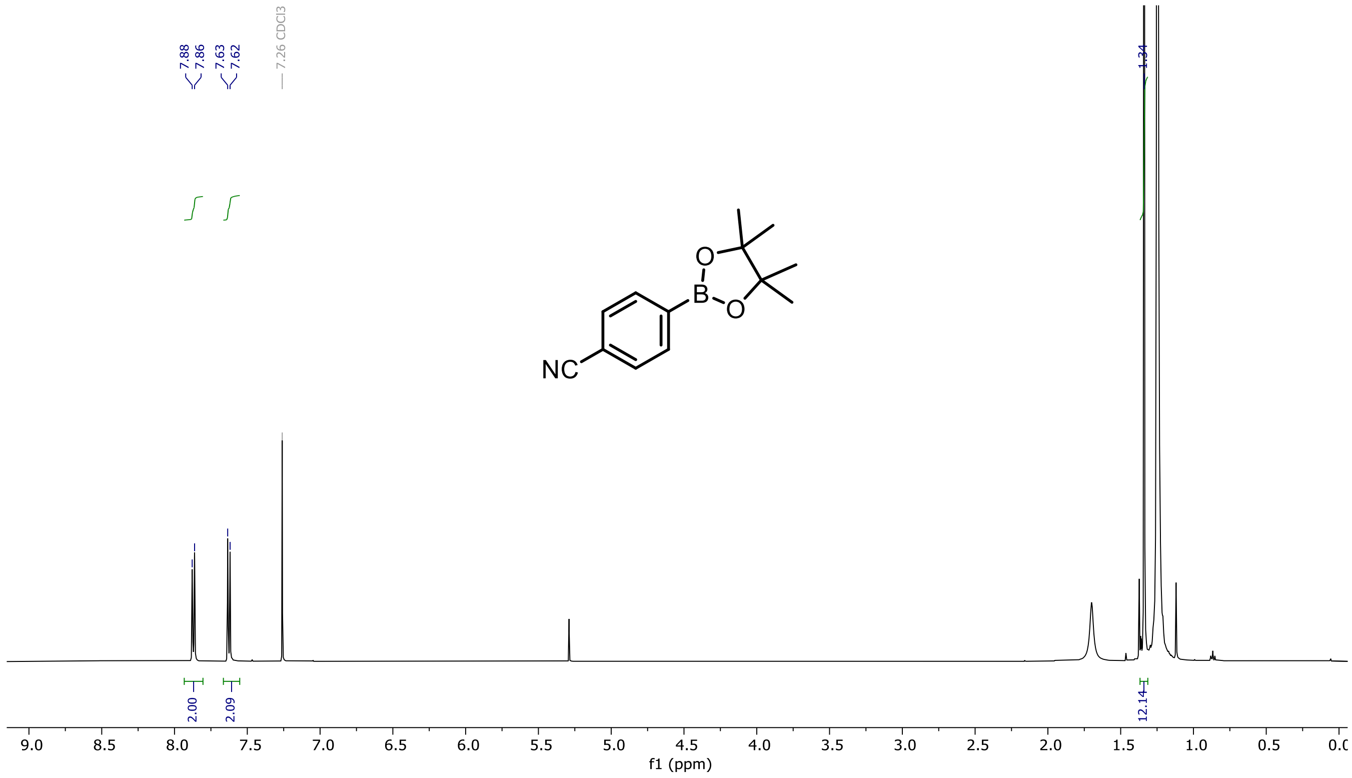

$^{13}C\{^{1}H\}$ NMR (126 MHz, $CDCl_3$)

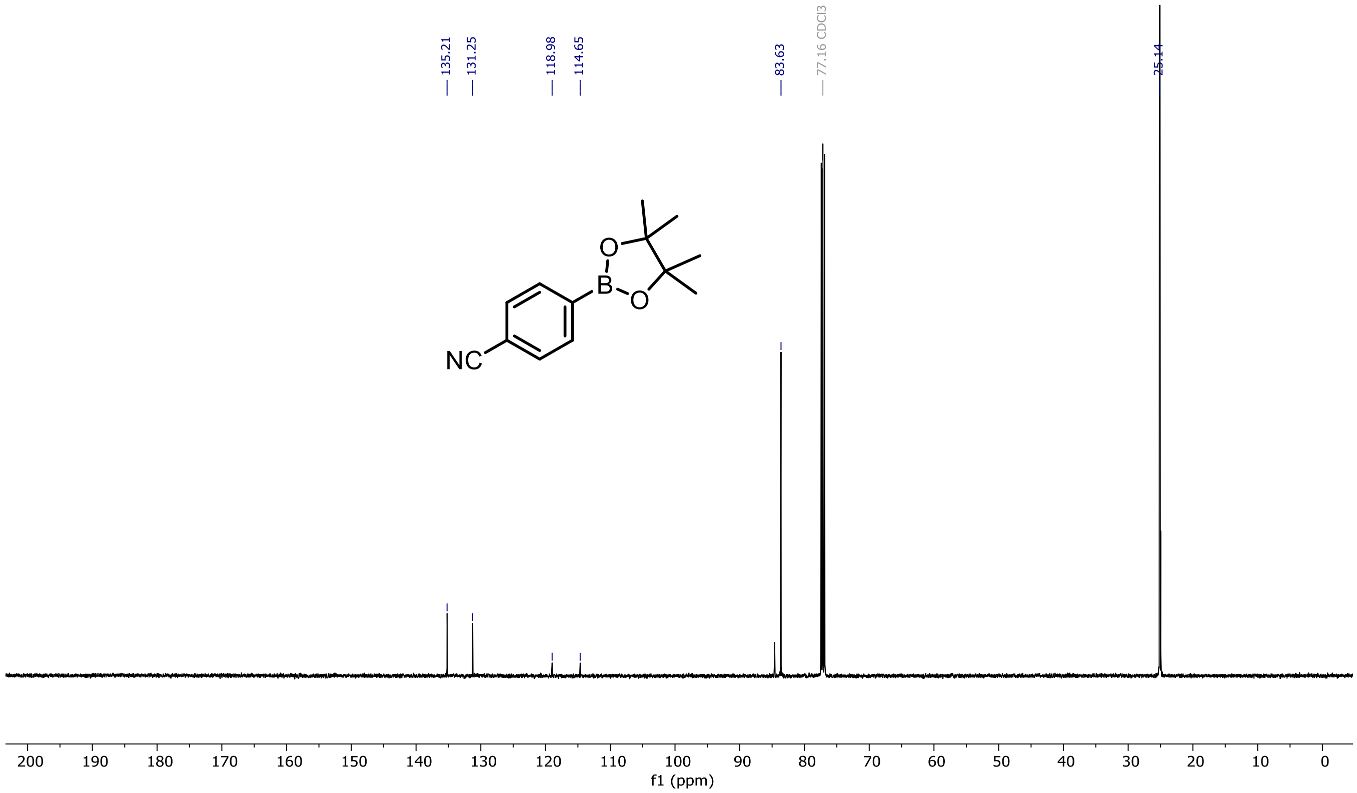

^{11}B NMR (160 MHz, $CDCl_3$)

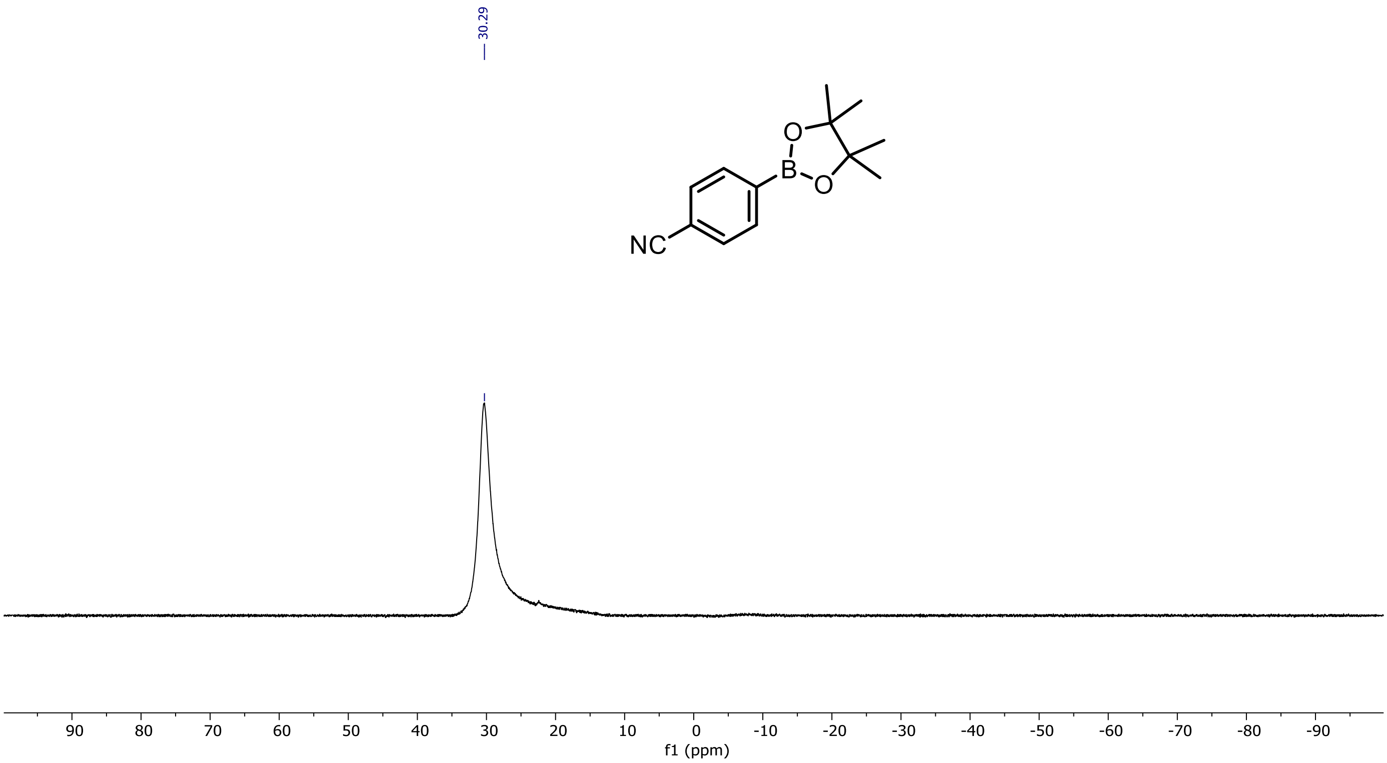

1-(4-(4,4,5,5-Tetramethyl-1,3,2-dioxaborolan-2-yl)phenyl)ethan-1-one (6y)

^{1}H NMR (500 MHz, $CDCl_3$)

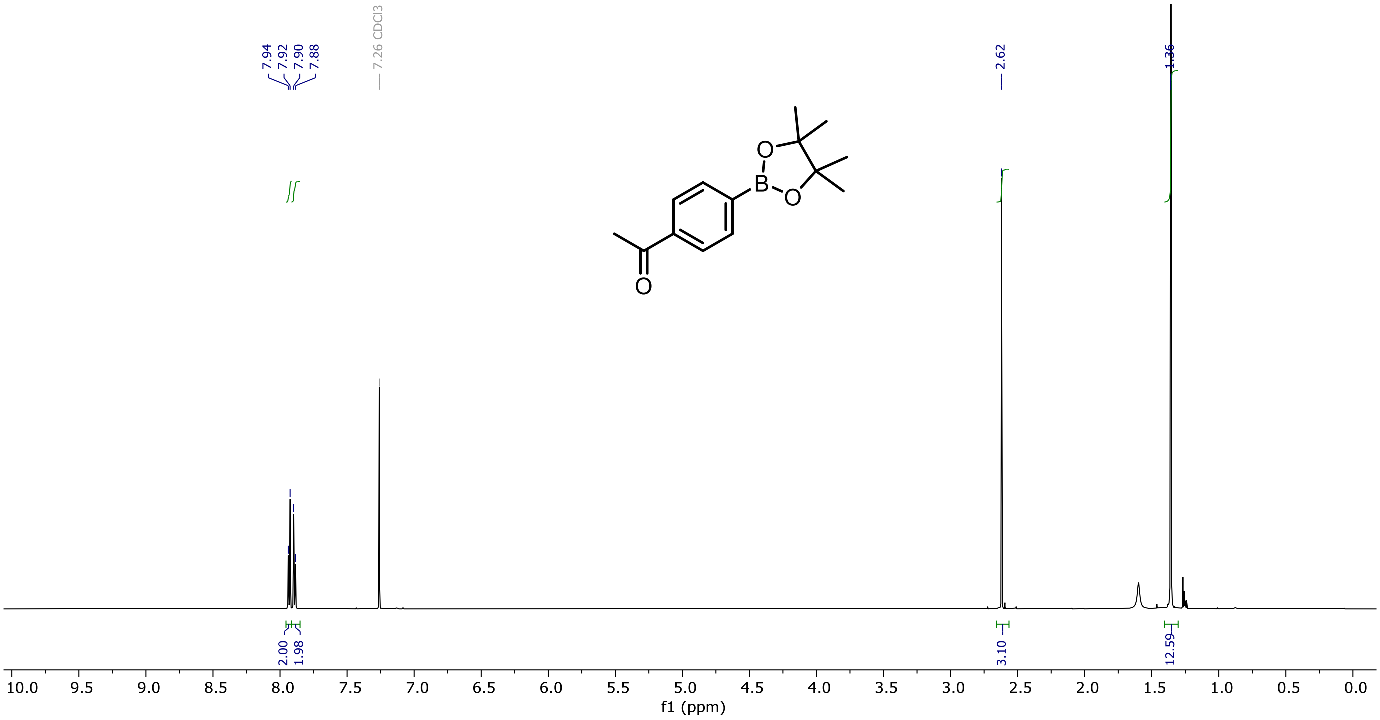

$^{13}C\{^{1}H\}$ NMR (126 MHz, $CDCl_3$)

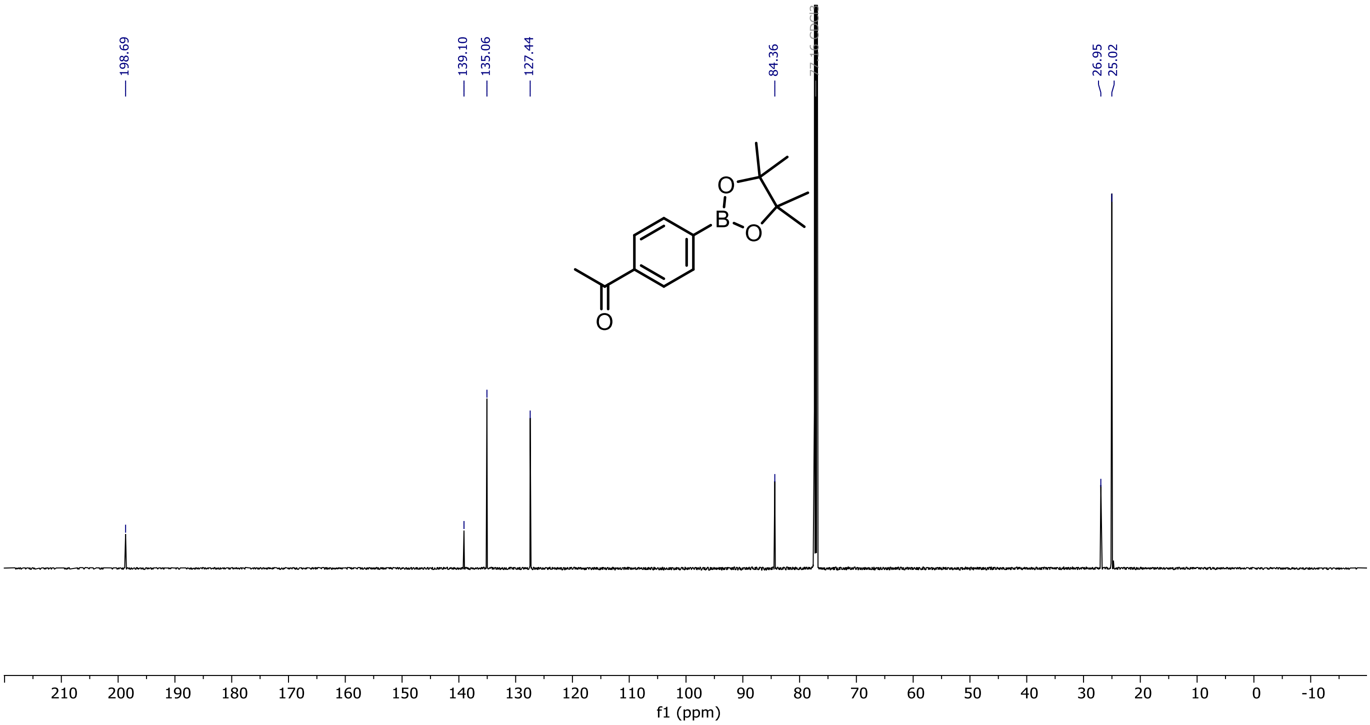

^{11}B NMR (160 MHz, $CDCl_3$)

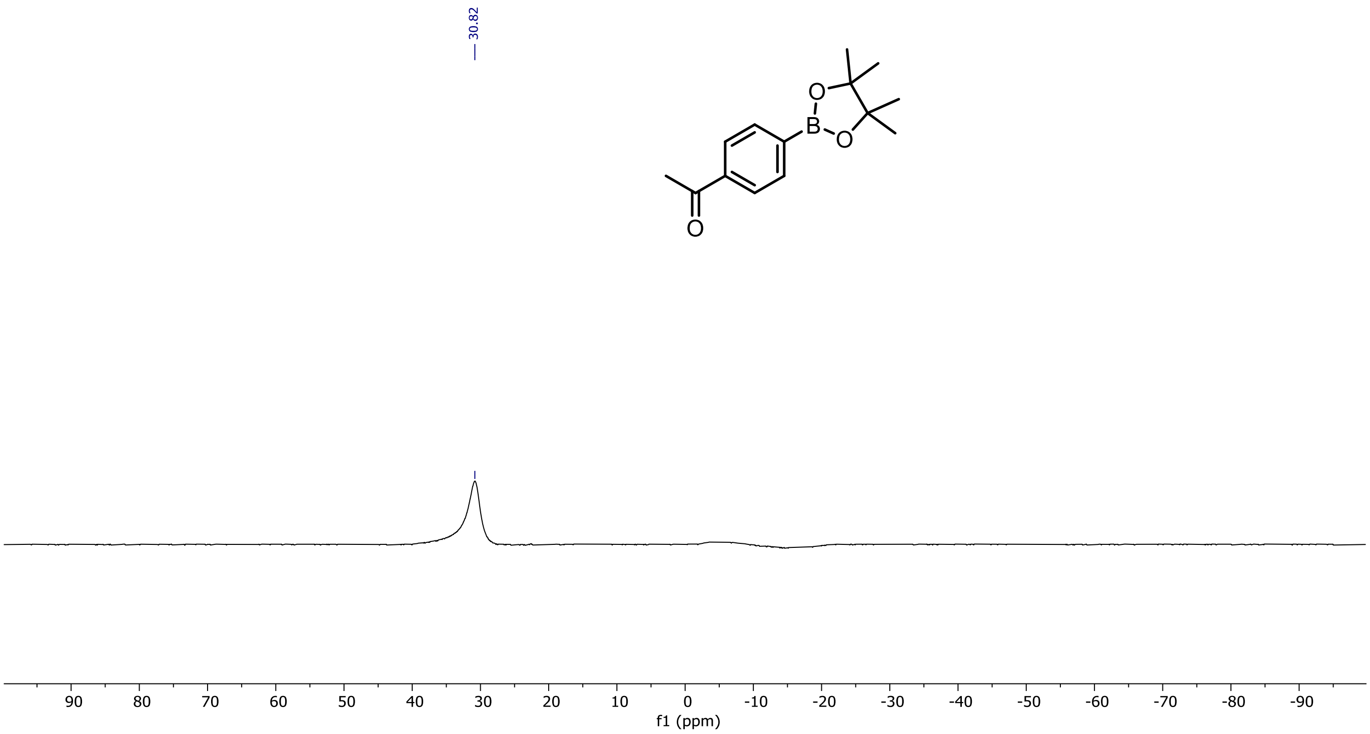

Bis(4-(4,4,5,5-tetramethyl-1,3,2-dioxaborolan-2-yl)phenyl)methanone (6z)

^{1}H NMR (500 MHz, $CDCl_3$)

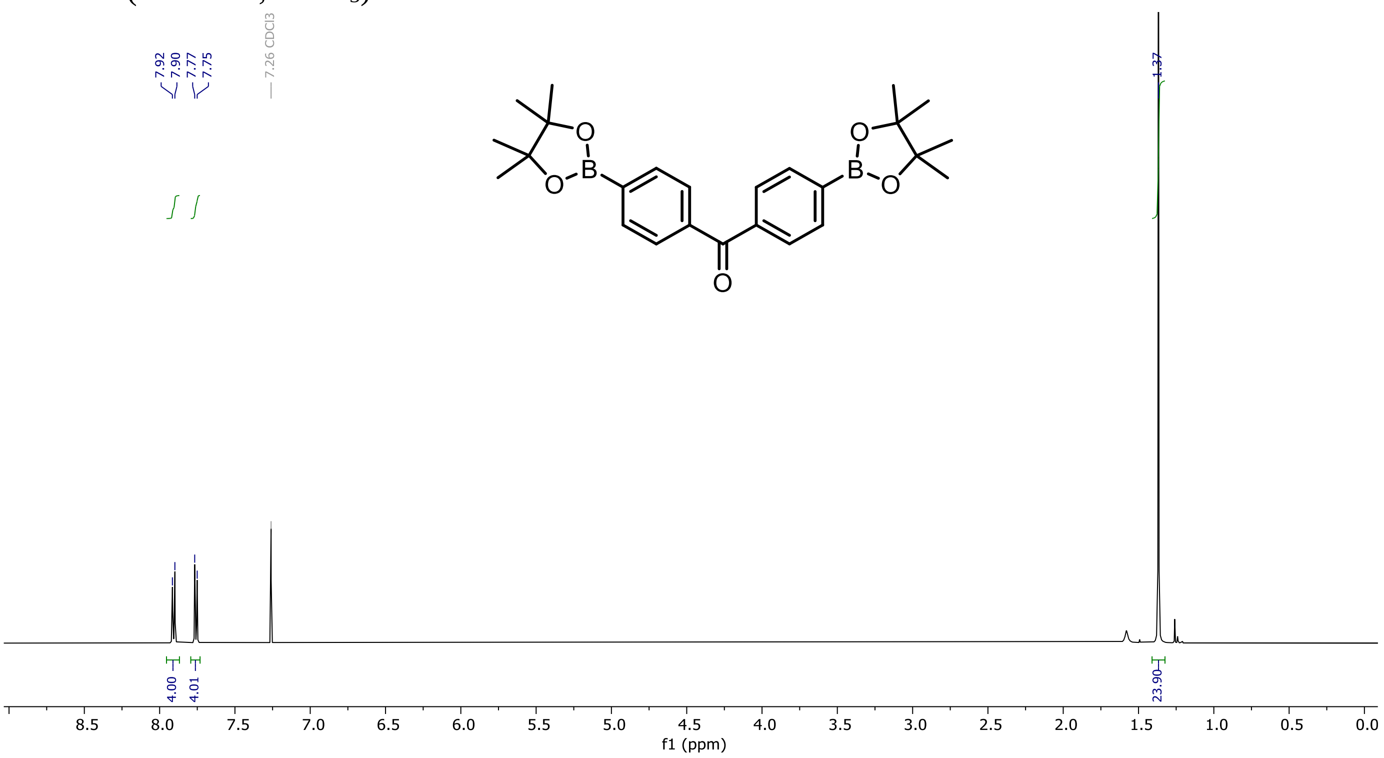

$^{13}C\{^{1}H\}$ NMR (126 MHz, $CDCl_3$)

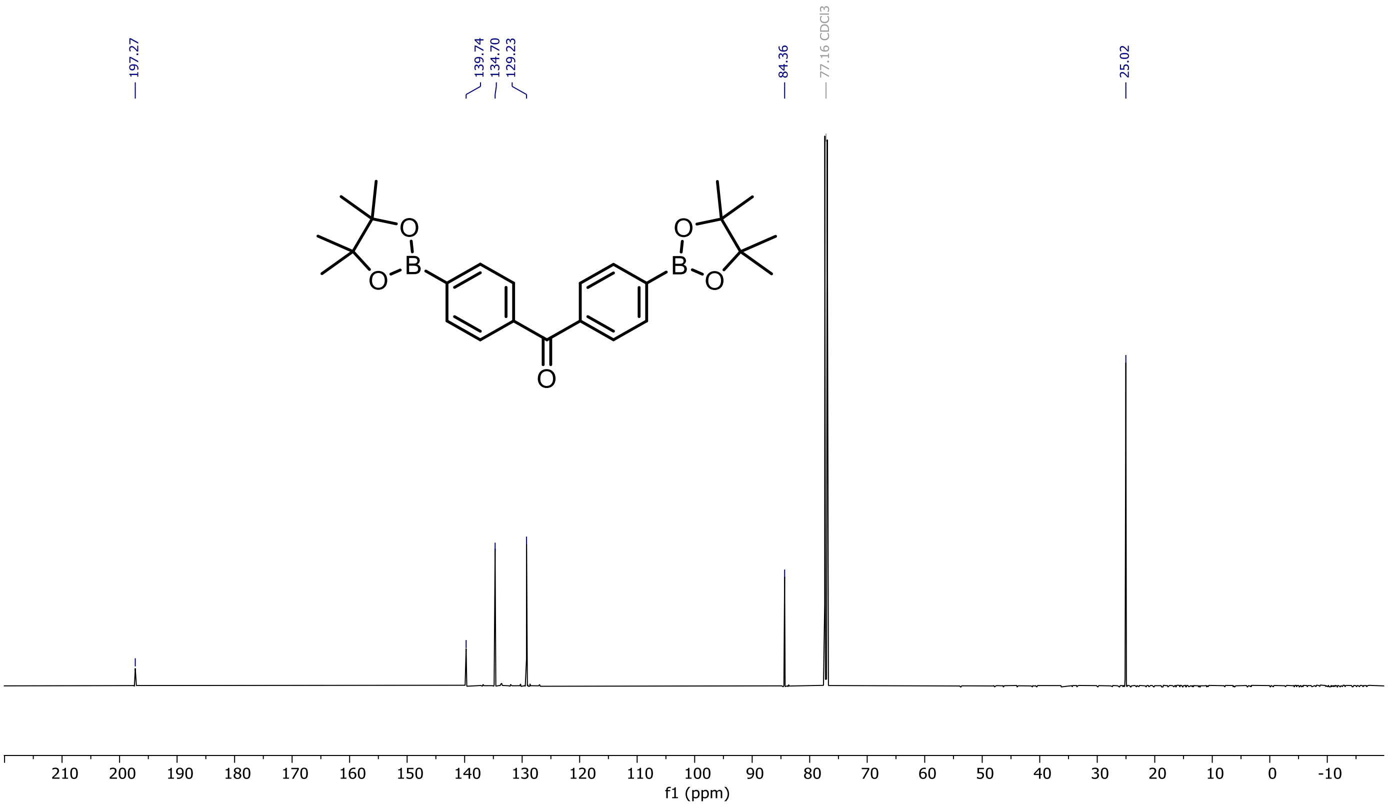

^{11}B NMR (160 MHz, $CDCl_3$)

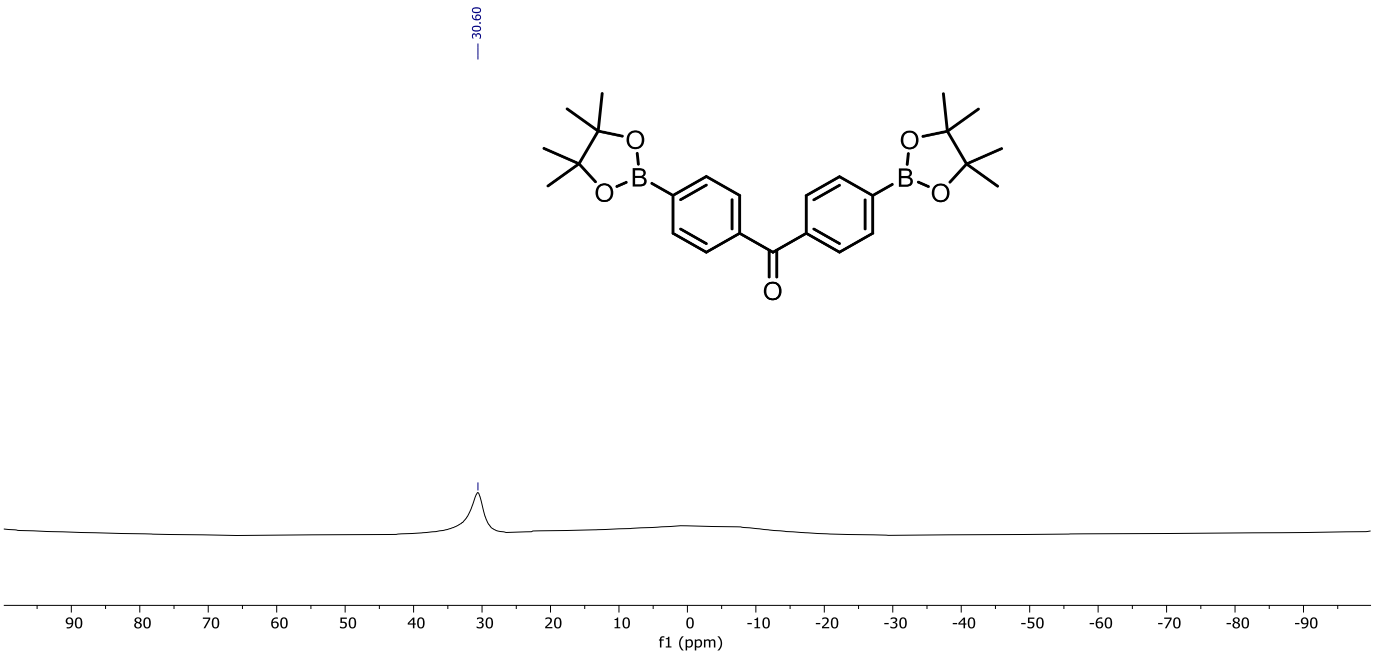

2-(Diethylamino)ethyl 4-(4,4,5,5-tetramethyl-1,3,2-dioxaborolan-2-yl)benzoate (8)

^{1}H NMR (500 MHz, $CDCl_3$)

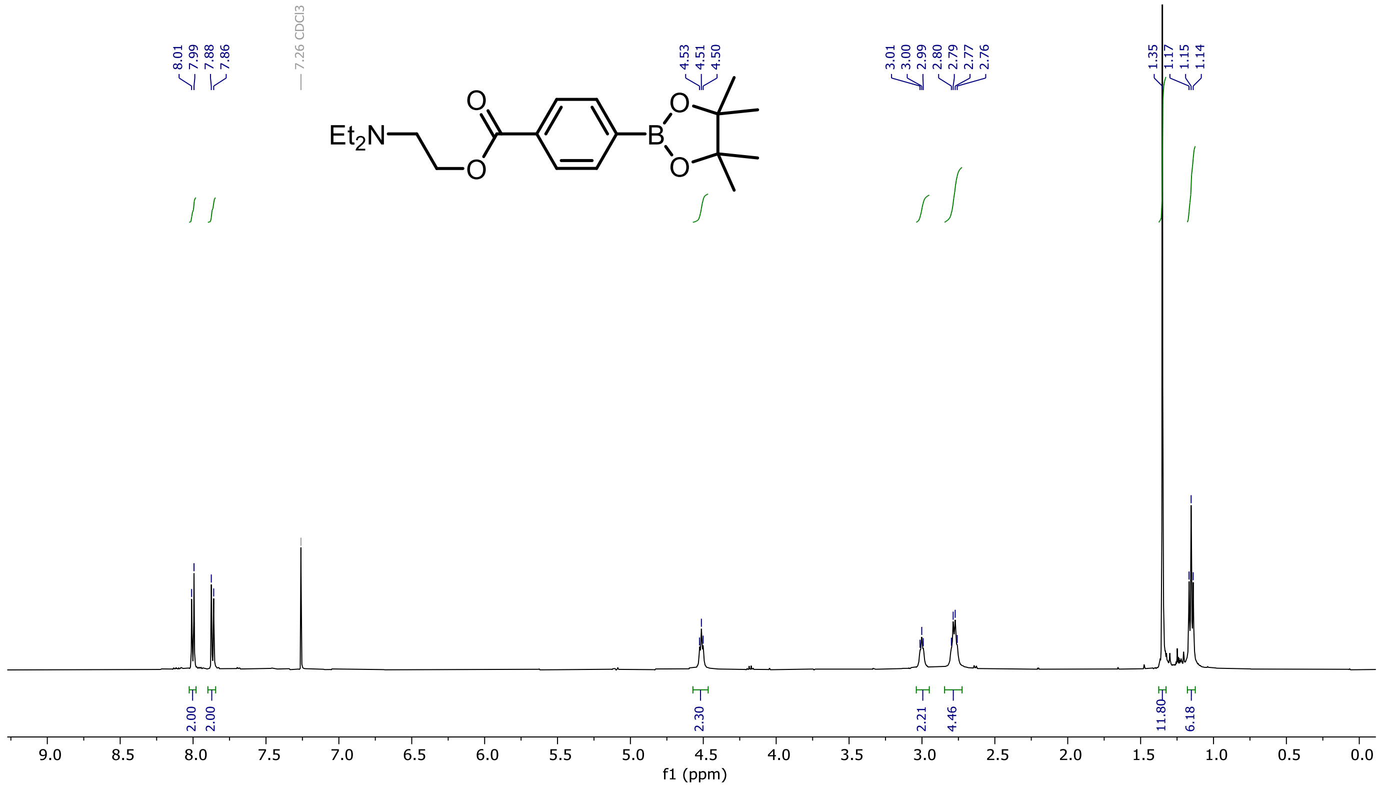

$^{13}C\{^1H\}$ NMR (126 MHz, $CDCl_3$)

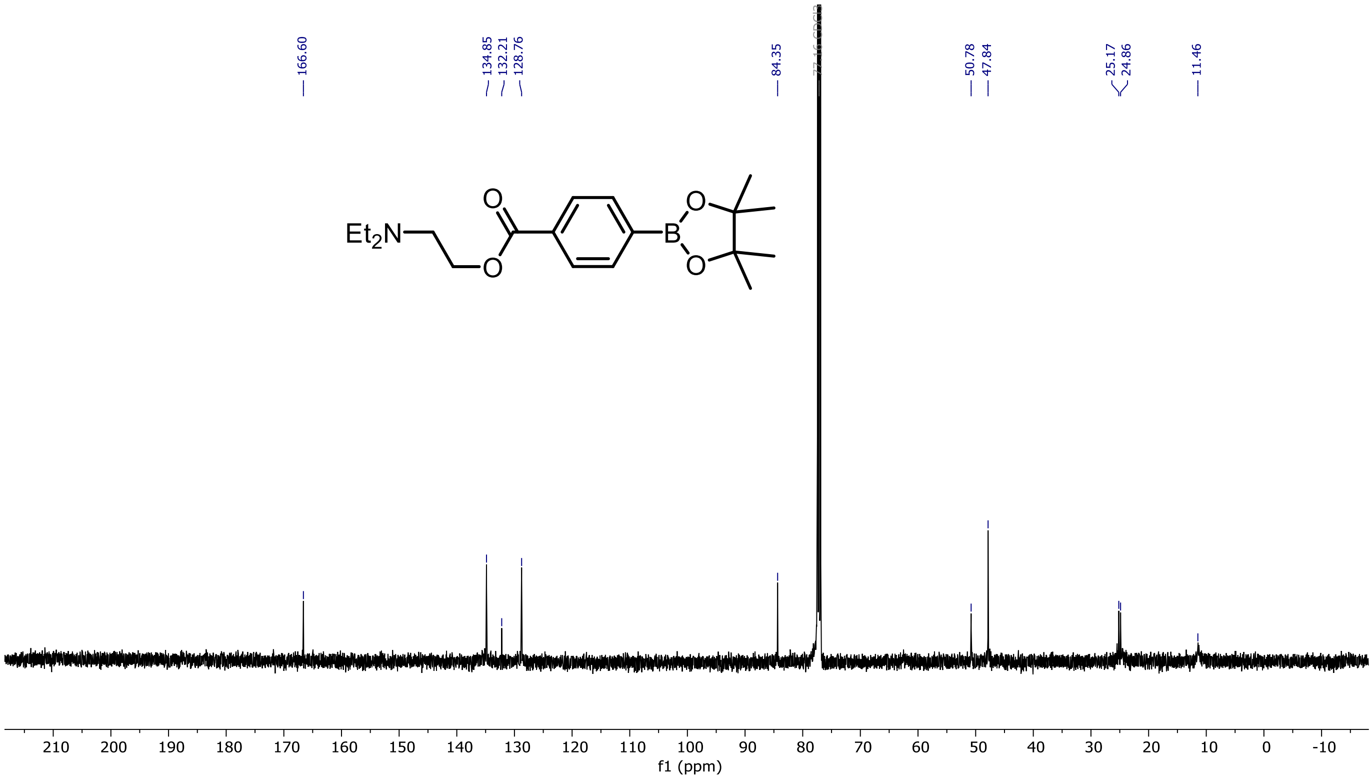

^{11}B NMR (160 MHz, $CDCl_3$)

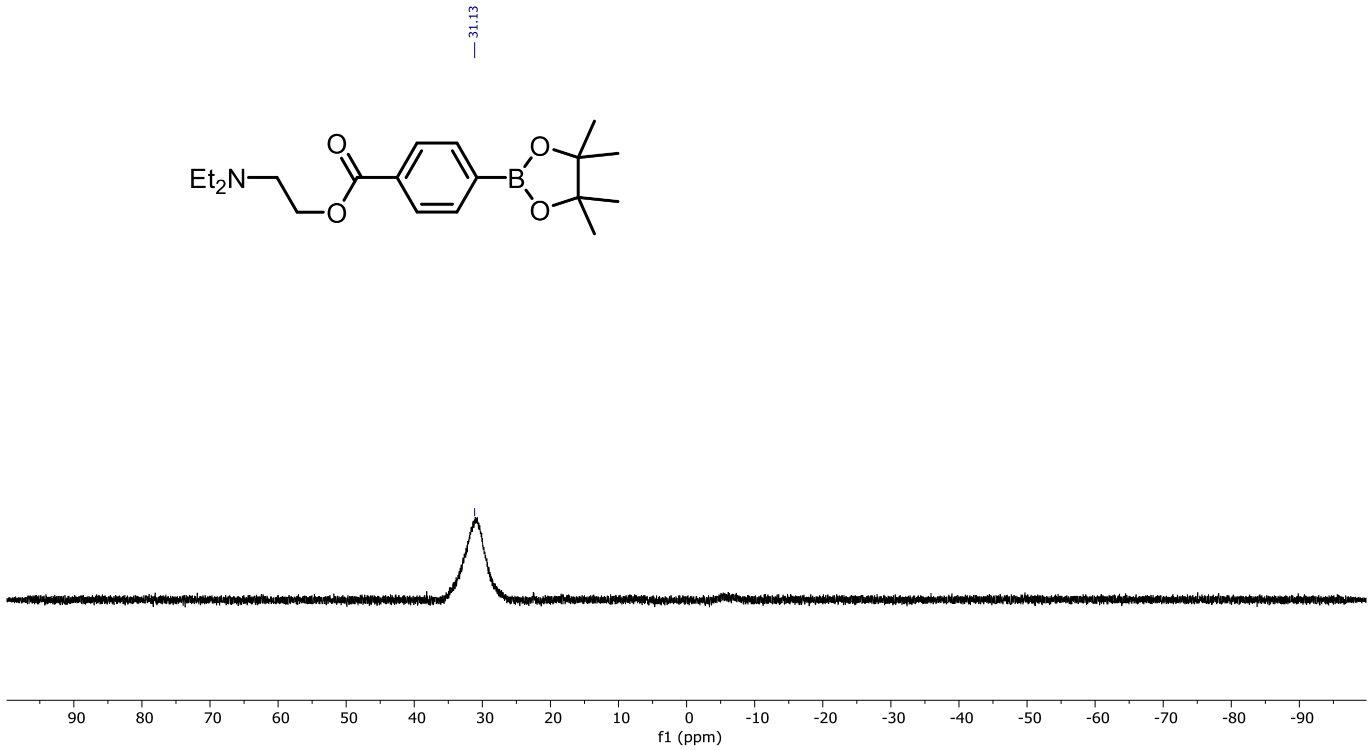

Potassium trifluoro(p-tolyl)borate (S1)

^{1}H NMR (500 MHz, acetone-d_6)

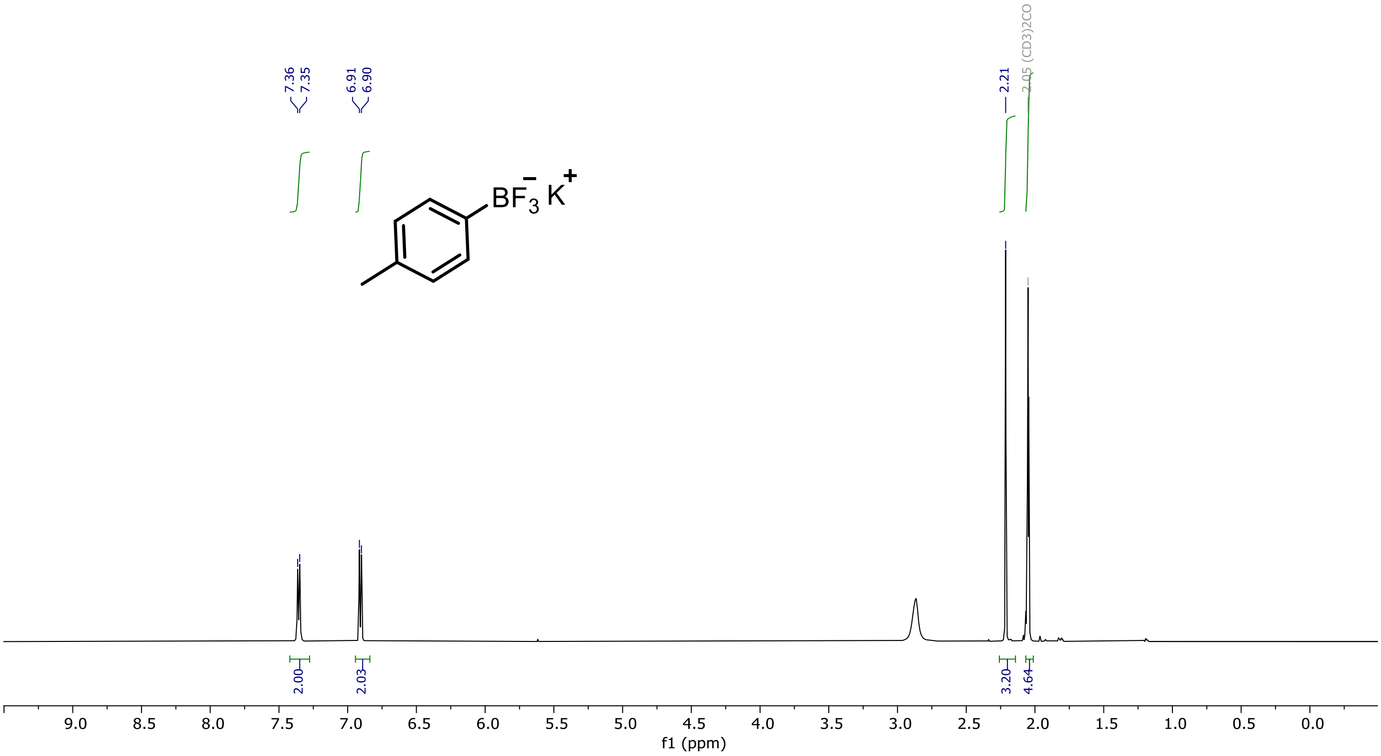

$^{13}C\{^{1}H\}$ NMR (126 MHz, acetone-d_6)

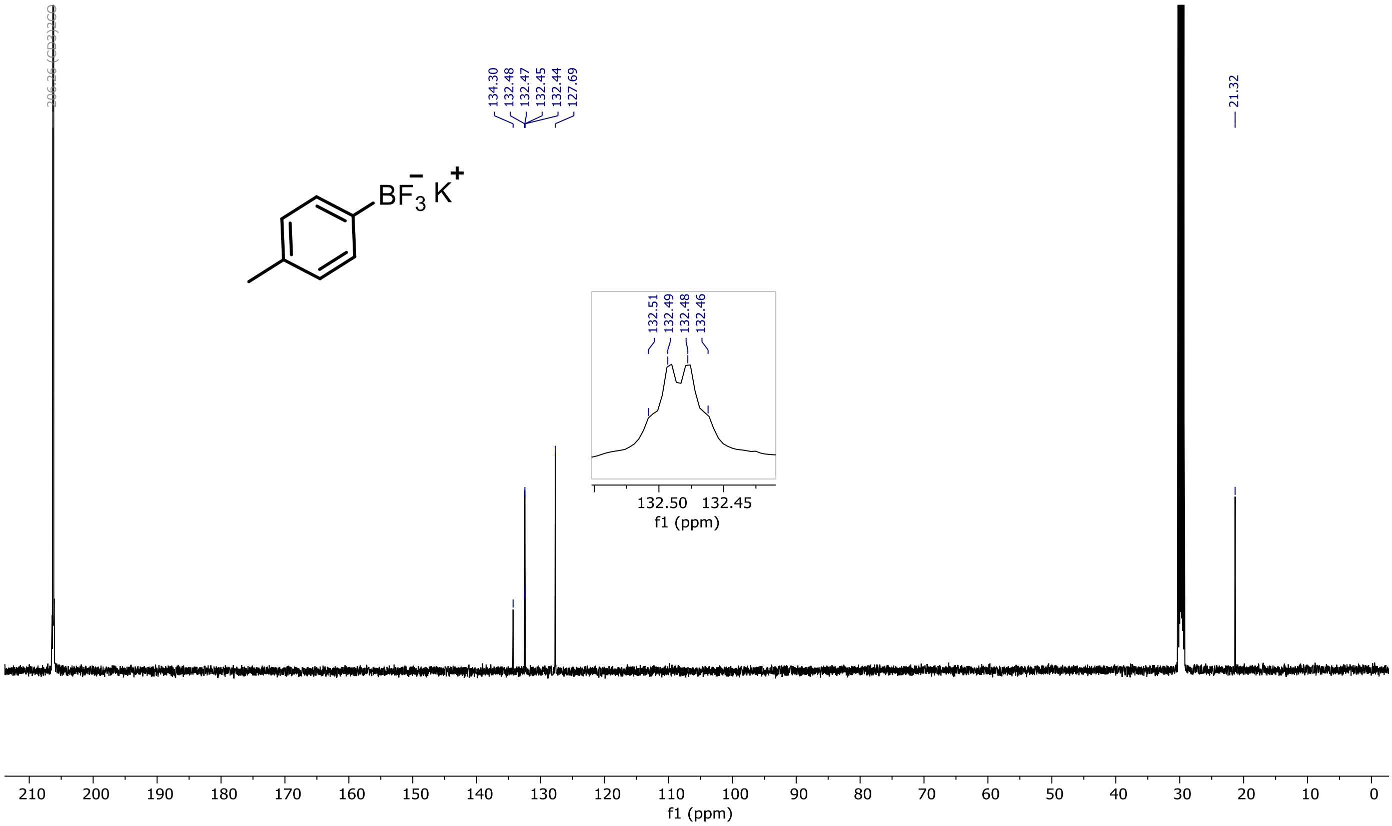

^{11}B NMR (160 MHz, acetone-d_6)

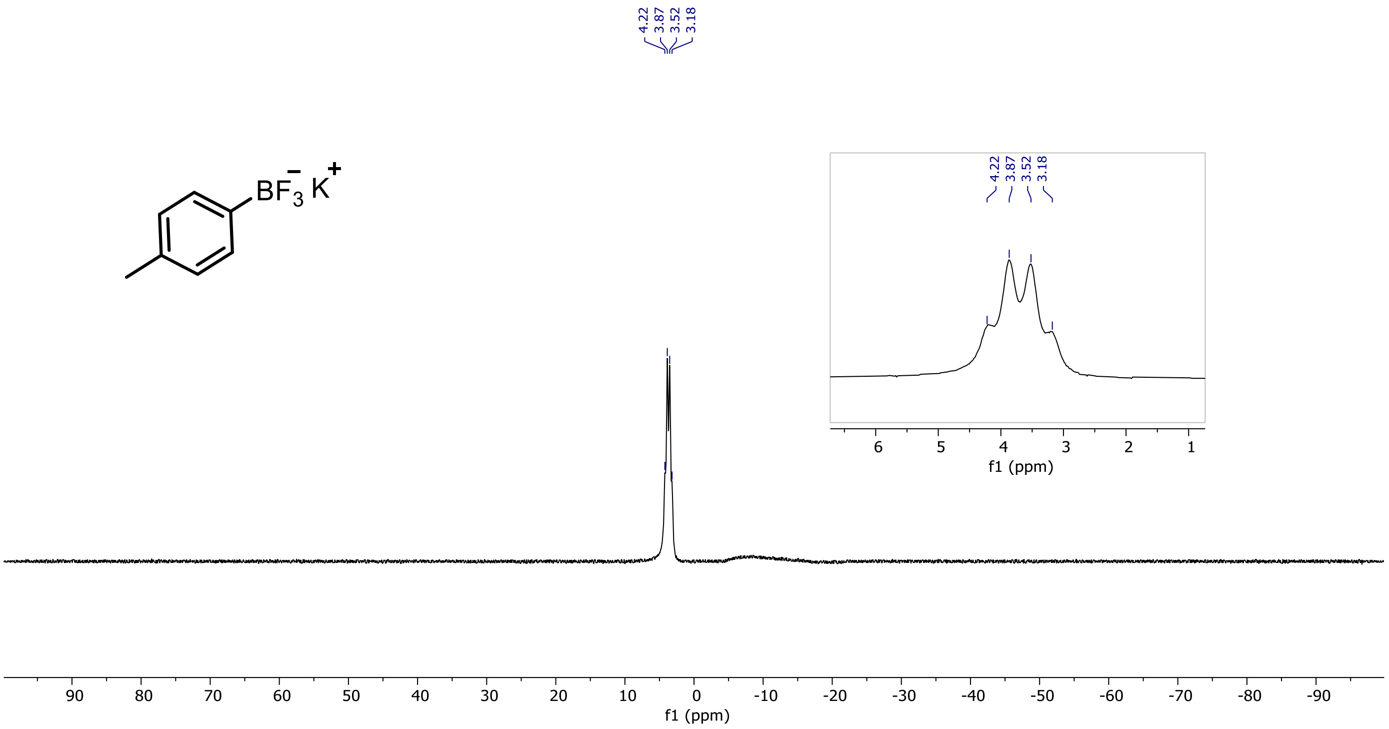

^{19}F NMR (470 MHz, acetone-d_6)

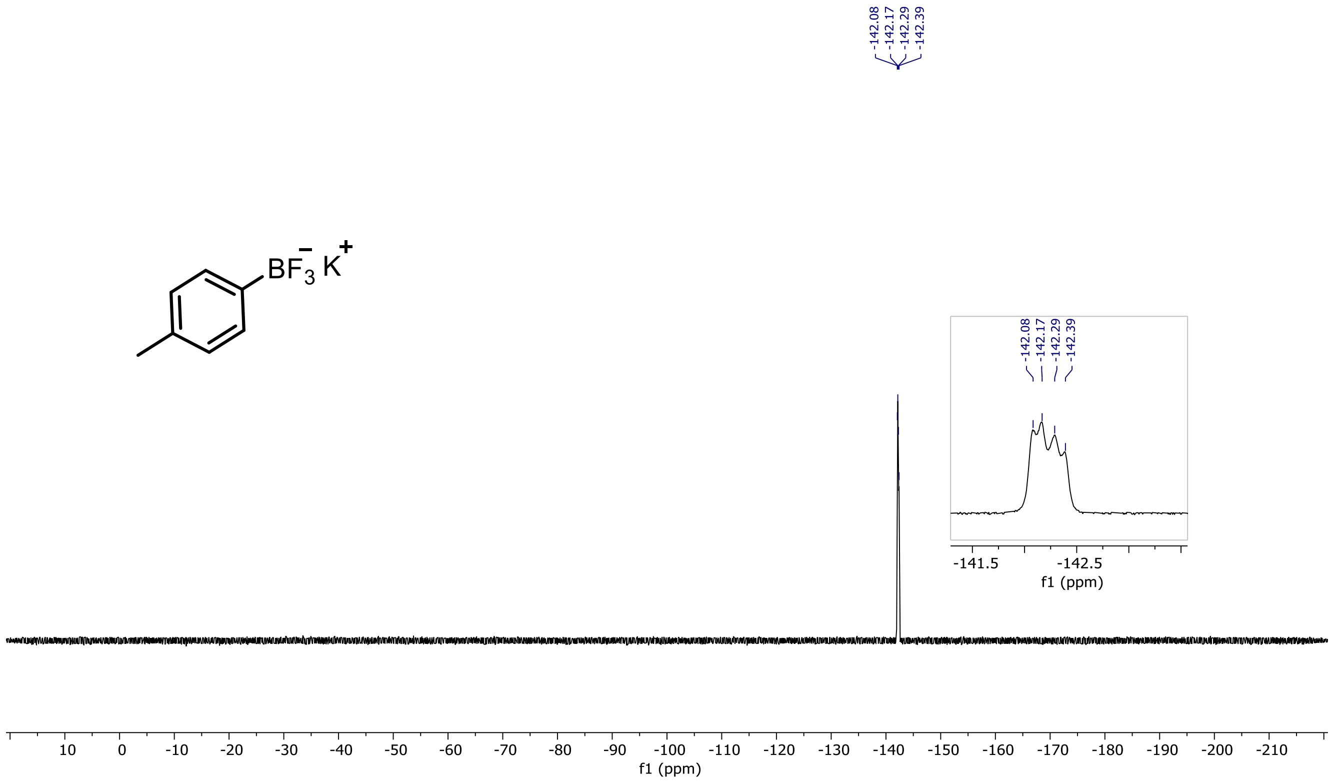

2-([1,1'-Biphenyl]-4-yl)acetic acid (felbinac, 10)

^{1}H NMR (500 MHz, DMSO-d_6)

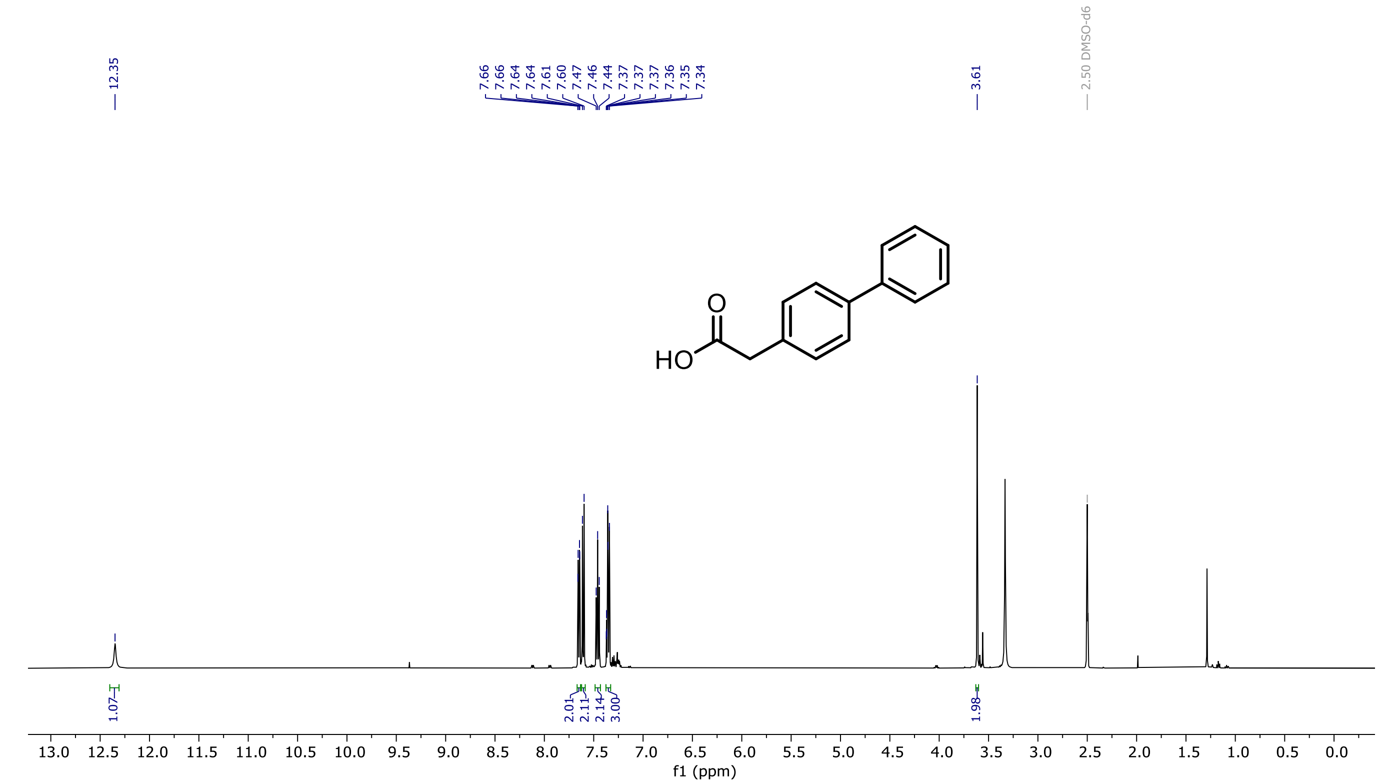

^{13}C{^{1}H} NMR (126 MHz, DMSO-d_6)

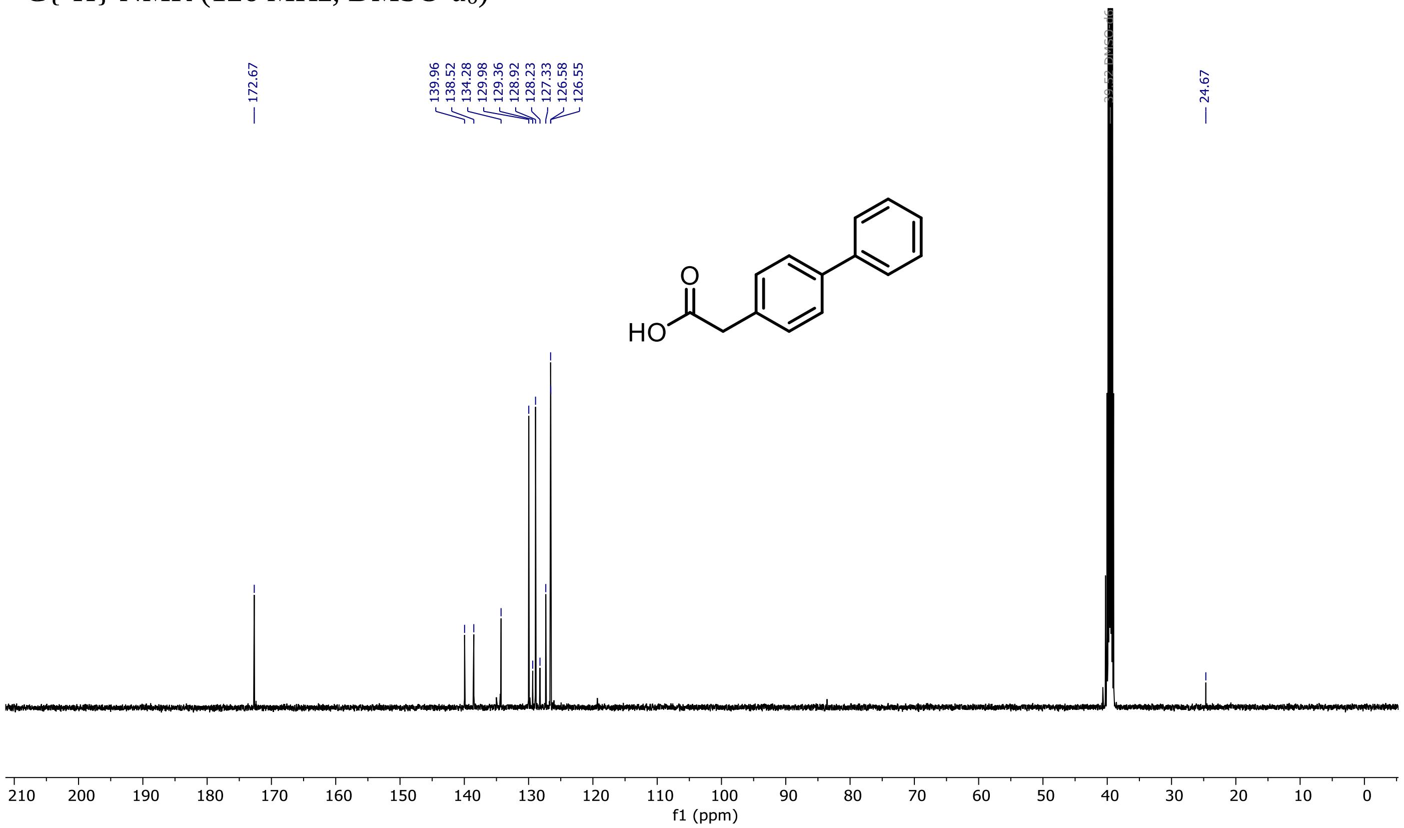

2-Morpholino-2-(p-tolyl)acetic acid (9)

^{1}H NMR (500 MHz, CD_3OD)

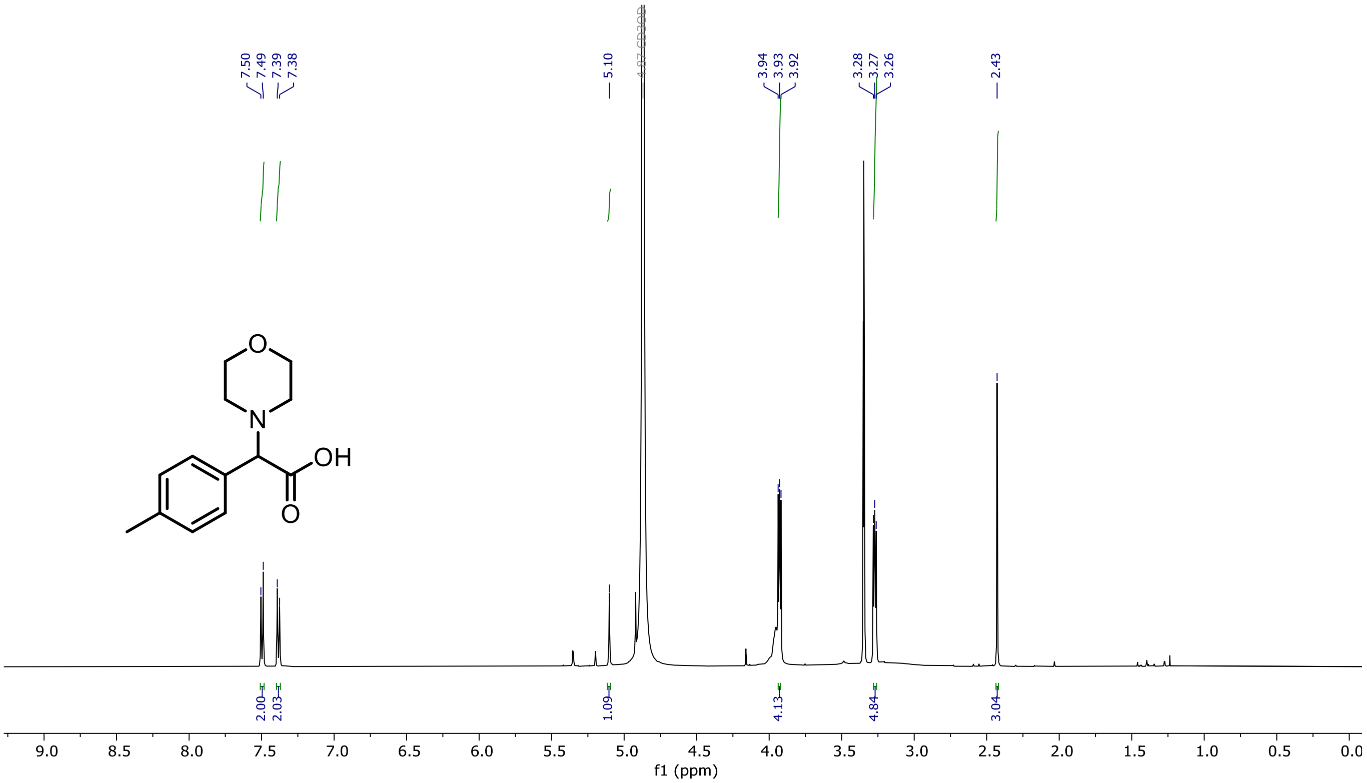

^{13}C{^{1}H} NMR (126 MHz, CD_3OD)

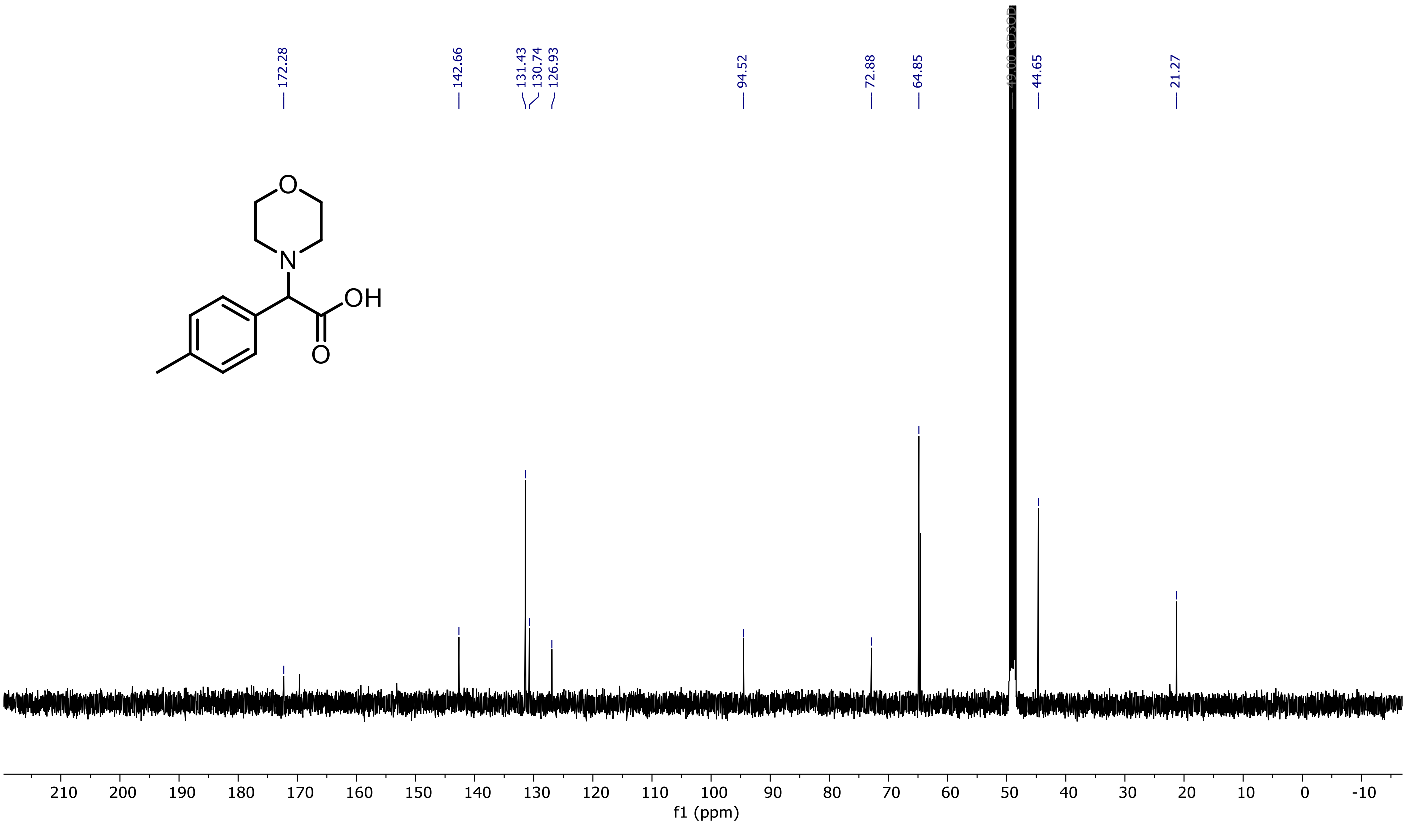

4,4,5,5-Tetraethyl-2-phenyl-1,3,2-dioxaborolane (12)

^{1}H NMR (500 MHz, $CDCl_3$)

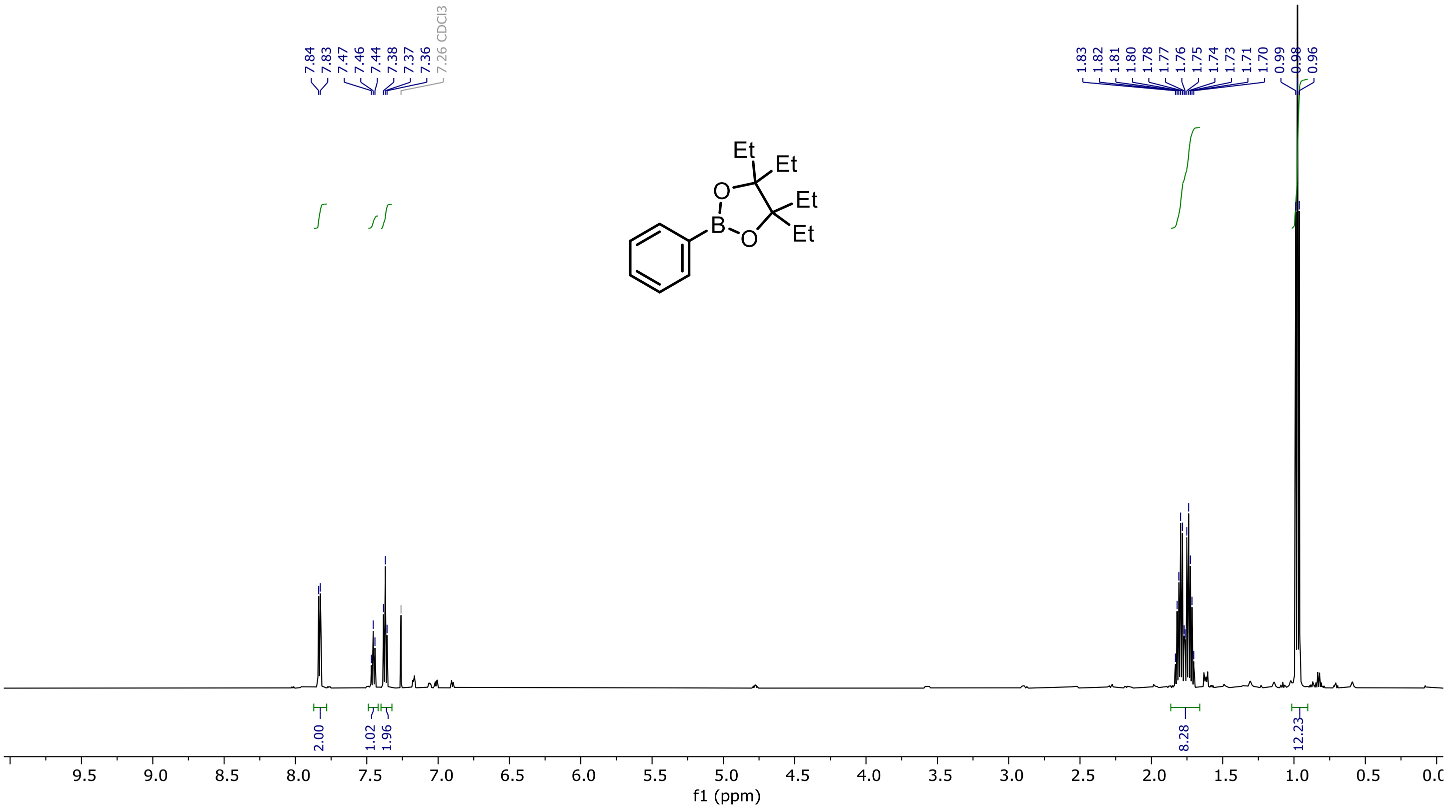

^{13}C{^{1}H} NMR (126 MHz, $CDCl_3$)

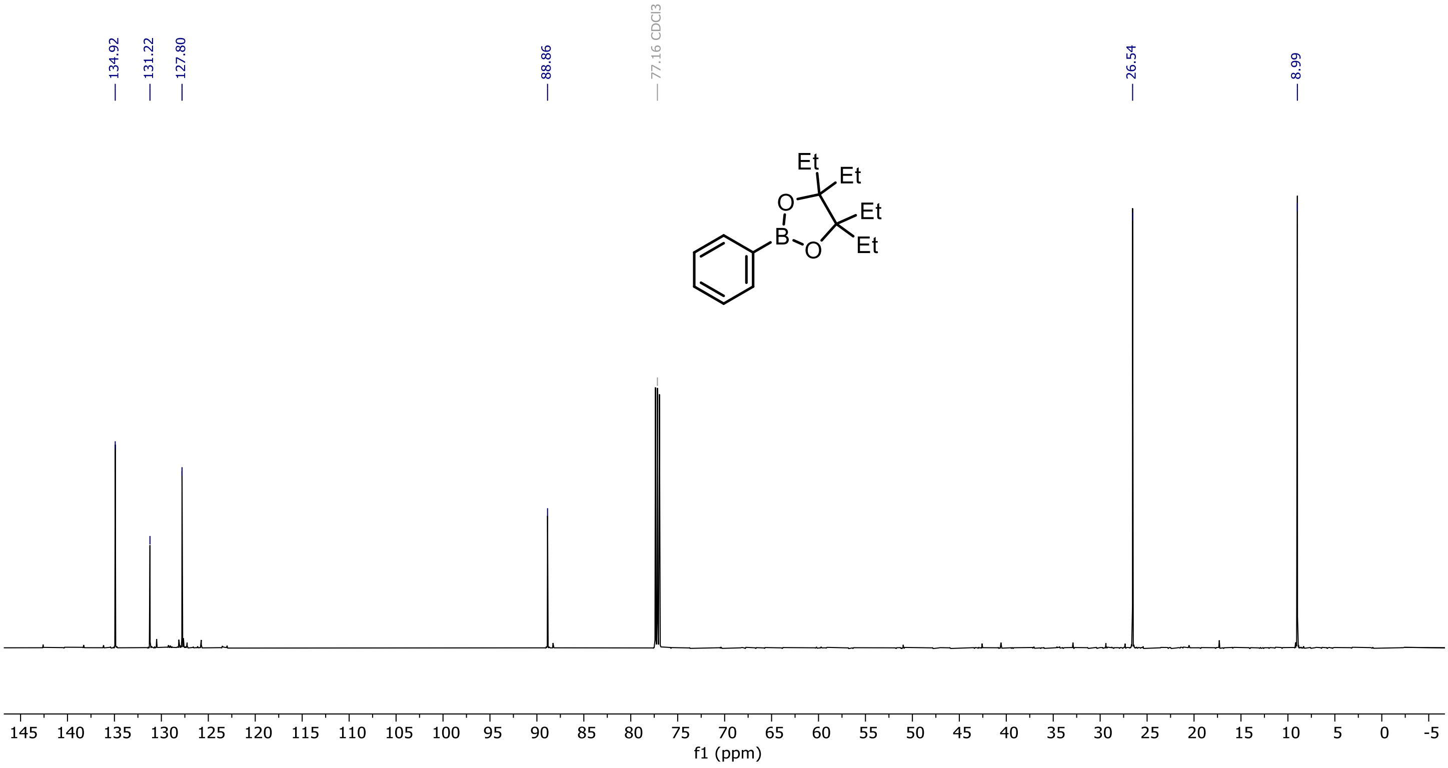

^{11}B NMR (160 MHz, $CDCl_3$)

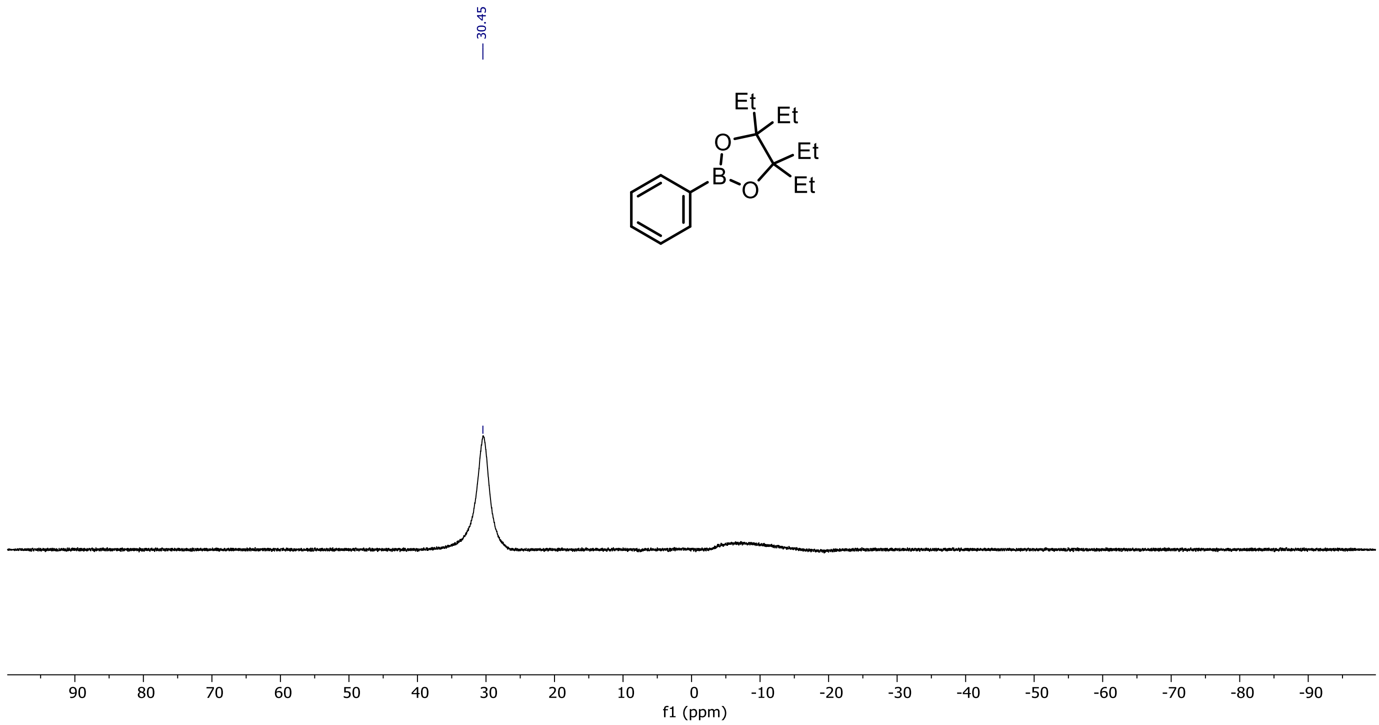

4,4,5,5-Tetraethyl-2-(p-tolyl)-1,3,2-dioxaborolane (13)

^{1}H NMR (500 MHz, $CDCl_3$)

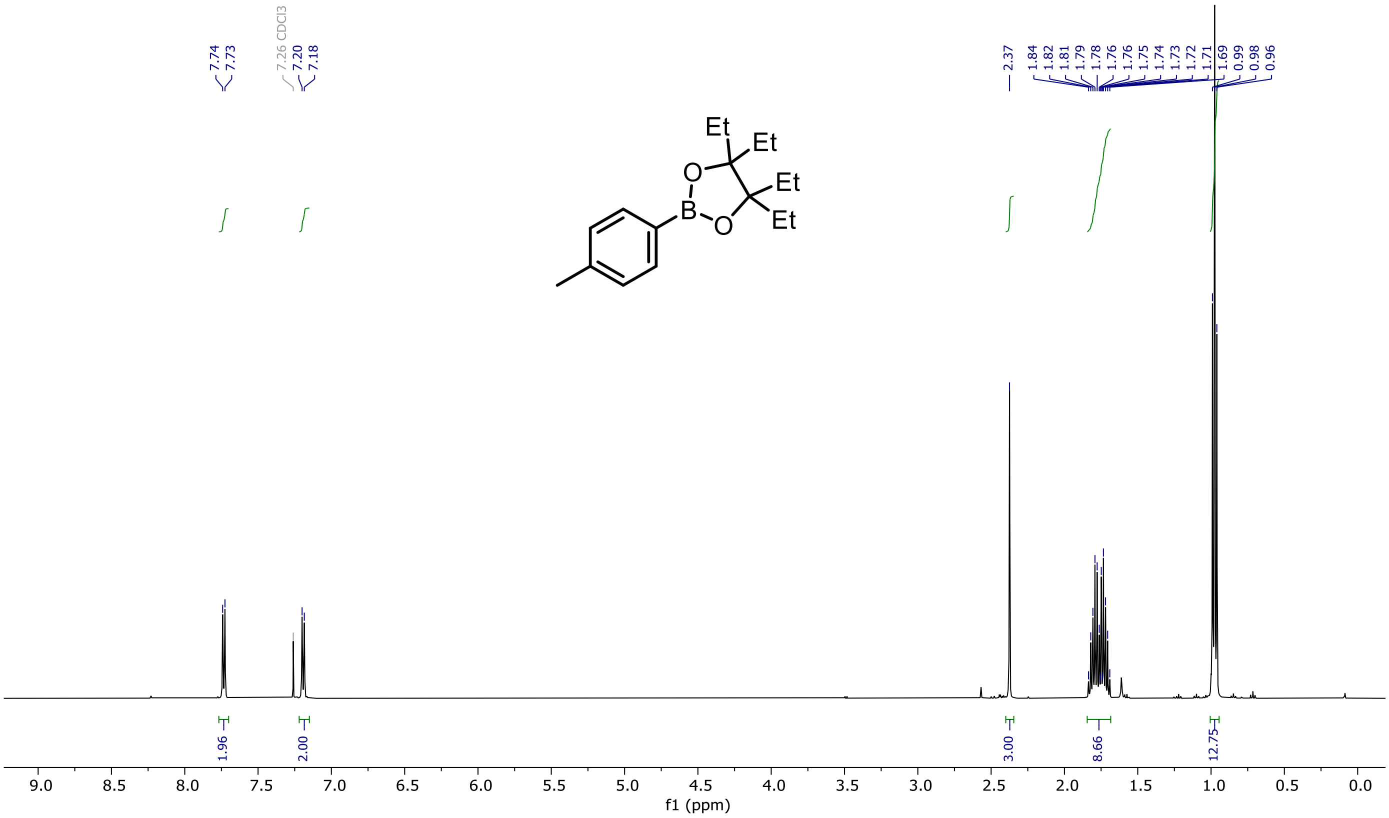

$^{13}C\{^{1}H\}$ NMR (126 MHz, $CDCl_3$)

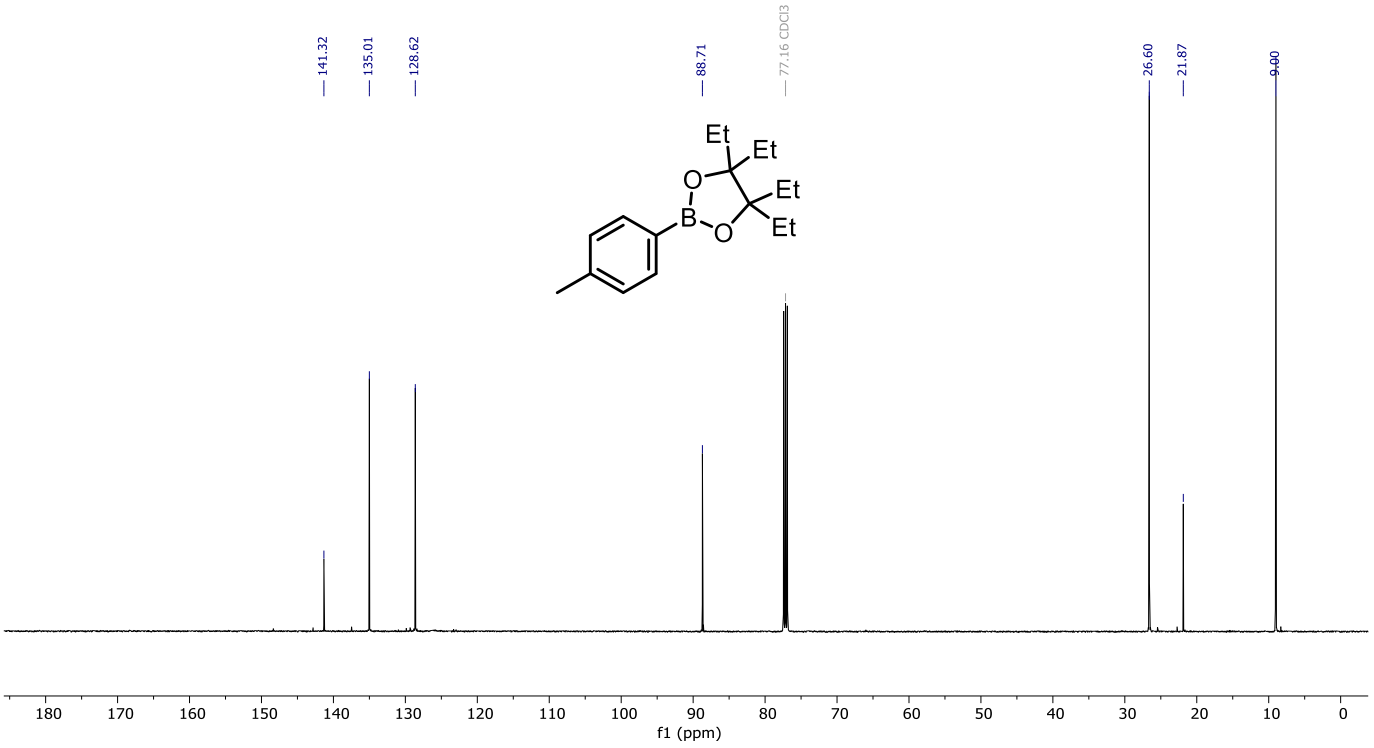

^{11}B NMR (160 MHz, $CDCl_3$)

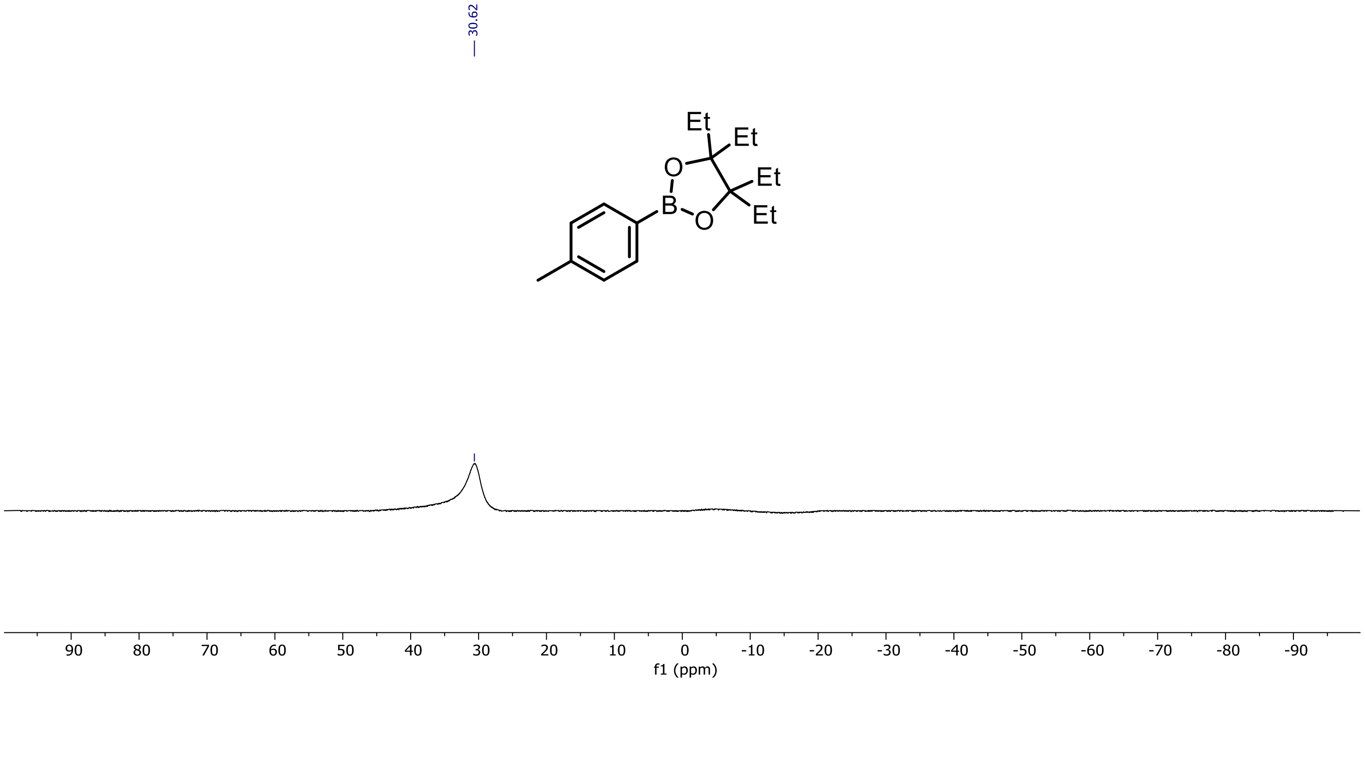

DFT Calculations

All DFT calculations were performed with the Gaussian 16 software package.[18] Geometries were optimized using the unrestricted uM06-2X functional and the 6-311+G(d,p) basis set in the gas phase. All geometry optimized structures were checked for negative frequencies and spin contamination. Single point energies were calculated using uM06-2X and 6-311++G(d,p) with the SMD solvation model in acetonitrile. Reported Gibbs free energies in the solution include thermal corrections computed at 298 K, obtained by frequency analyses using 6-311++g(d,p) basis set.

Comparing the energetic profiles between catalytic and chain transfer pathways. DFT and Marcus theory calculations show that the SET step has a lower activation barrier in catalytic pathway compared to the chain transfer pathway. Additionally, in this step the catalytic pathway is more exergonic.

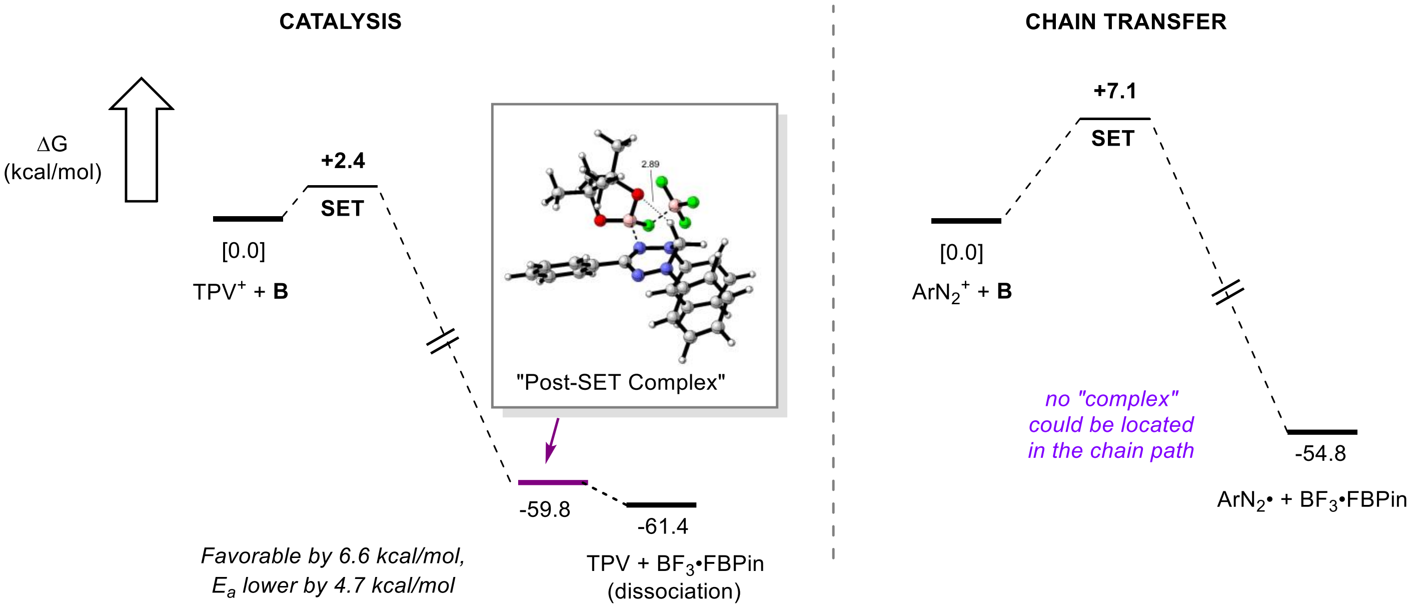

Figure S5. DFT-calculated energetic profiles of catalytic and chain transfer pathways.

Using Marcus Theory to estimate the activation barriers for SET

According to the Marcus equation[19-23], the activation barrier can be estimated as:

$$\Delta G^{\circ\ddagger} = \frac{(\Delta G + \lambda)^2}{4\lambda}$$

Where ΔG is the thermodynamic driving force and λ is the total reorganization energy, which is the sum of inner-sphere reorganization energy (λ_i) and outer-sphere reorganization energy (λ_o).

The internal reorganization energy λ_i can be estimated according to the equation:

$$\lambda_{i1} = [E^{D^{\bullet -}}(Q^P) + E^{A^+}(Q^P)] - [E^{D^{\bullet -}}(Q^R) + E^{A^+}(Q^R)]$$

$$\lambda_{i2} = [E^{D}(Q^R) + E^{A^\bullet}(Q^R)] - [E^{D}(Q^R) + E^{A^\bullet}(Q^R)]$$

$$\lambda_i = \frac{\lambda_{i1} + \lambda_{i2}}{2}$$

where λ_{i1} represents the difference between the energy of the reactants (R) in the geometry characteristic of the products and that in their ground state geometry, λ_{i2} is the difference between the energy of the products (P) in the characteristic geometry of the reactants and that in their ground state equilibrium geometry. Q^R and Q^P denote the optimum geometries of the reactants and products, respectively. E^D and E^A denote the energies of donor and acceptor in the SET reaction $E^{D^{\bullet -}} + E^{A^+} \rightarrow E^D + E^{A^\bullet}$.

In the case of catalytic pathway with post-SET complex, for the calculation of the energies of reactants at the product geometry [$E^{D^{\bullet -}}(Q^P)$ and $E^{A^+}(Q^P)$], the nuclear coordinates used were their geometries adopted in the post-SET complex.

The external reorganization energy λ_o was calculated as:

$$\lambda_o = \left(332\frac{kcal}{mol}\right)\left(\frac{1}{2a_1} + \frac{1}{2a_2} - \frac{1}{R}\right)\left(\frac{1}{\varepsilon_{op}} - \frac{1}{\varepsilon}\right)$$

where a_1 is the radii of the donor, a_2 is the radii of the acceptor and $R = a_1 + a_2$, the distance between two centers. The radii were obtained using the "volume" keyword in gaussian. The solvent parameters ε_{op} is the optical dielectric constant (ε_{op} = 1.78 for acetonitrile) and ε is the static dielectric constant (ε_{op} = 37.5 for acetonitrile).

Additionally, SET activation barrier in the catalytic pathway was calculated via an alternative approach – with modelling the products in their individual ground states. The inner reorganization energies in both cases were very close (λ_i = 72.1 and 72.2 kcal/mol respectively), with the slight difference in thermodynamics being the main factor, resulting in only 0.2 kcal/mol difference in activation barrier for SET at this level of theory.

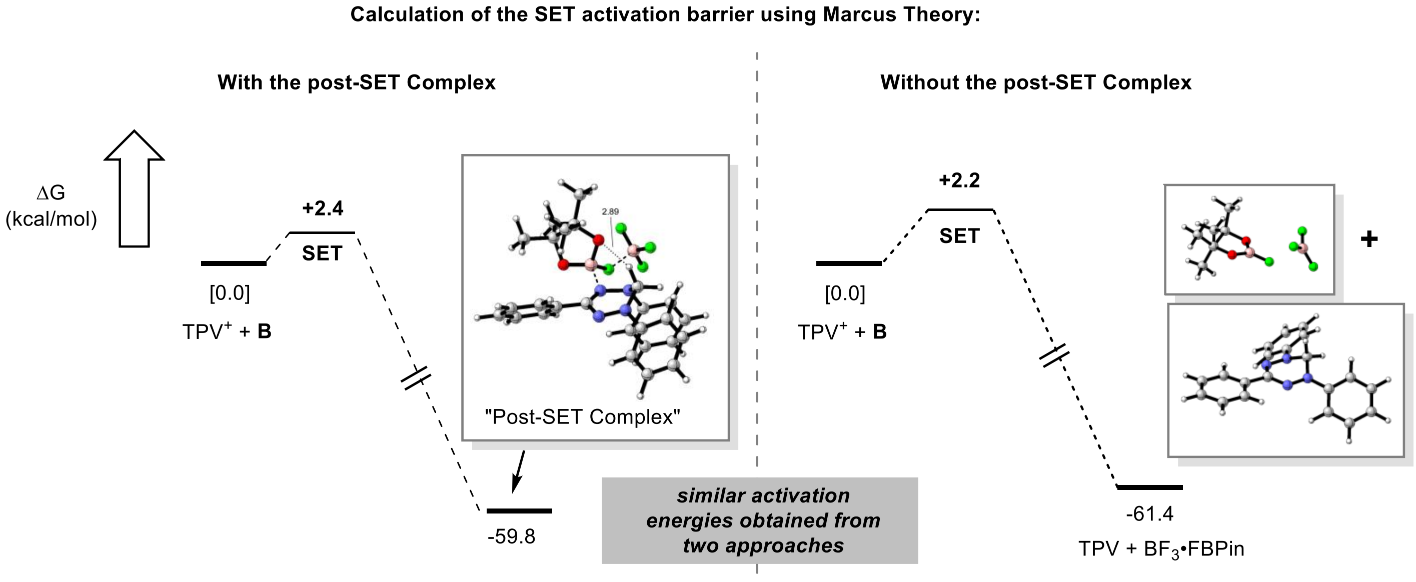

Figure S6. Comparison of activation energies obtain from using Post-SET complex and without

Molecular orbitals and spin density plots

We probed the electronic structure of the postulated ligated boryl anion radical **B**, which indicates significant spin density on the boron atom. We propose that the catalytic pathway benefits from having stabilizing interactions between the singly occupied molecular orbital (SOMO) of the boryl anion radical B and the lowest unoccupied molecular orbital (LUMO) of the verdazylium cation. The structure of the post-SET complex can be an indicator for this.

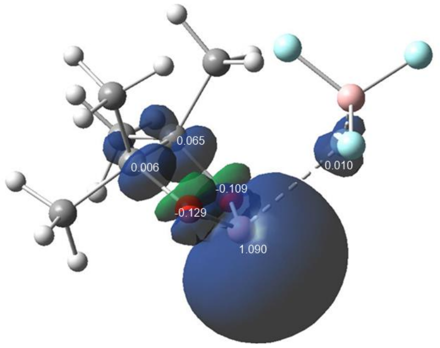

Figure S7. Mulliken spin density plot for boryl anion **B**

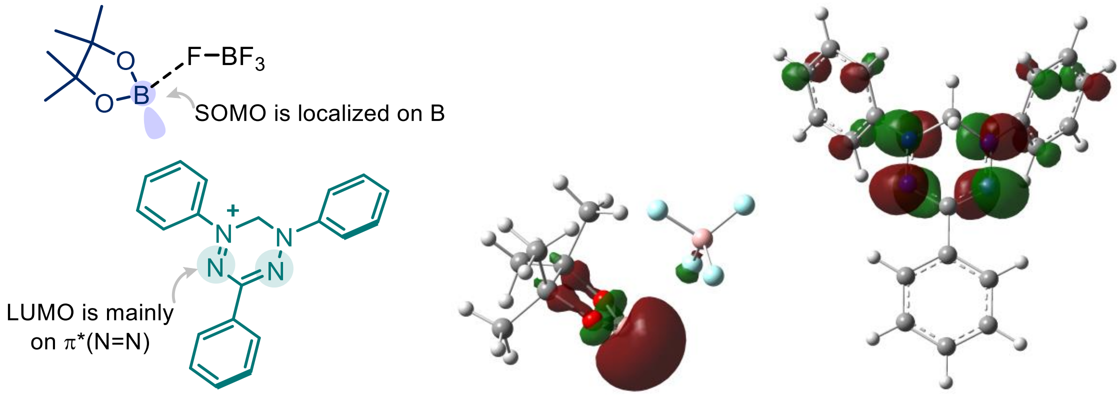

Figure S8. Frontier molecular orbitals of boryl anion and verdazylium.

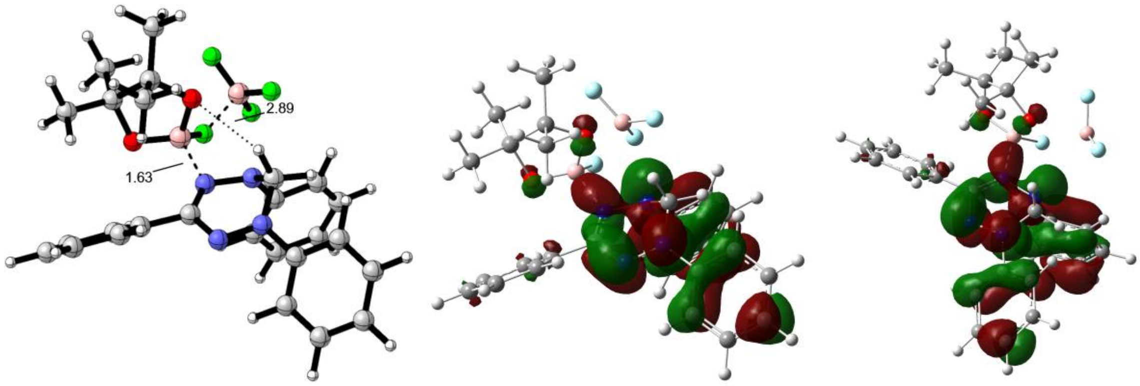

Figure S9. Singly occupied molecular orbital of the resultant complex after SET.

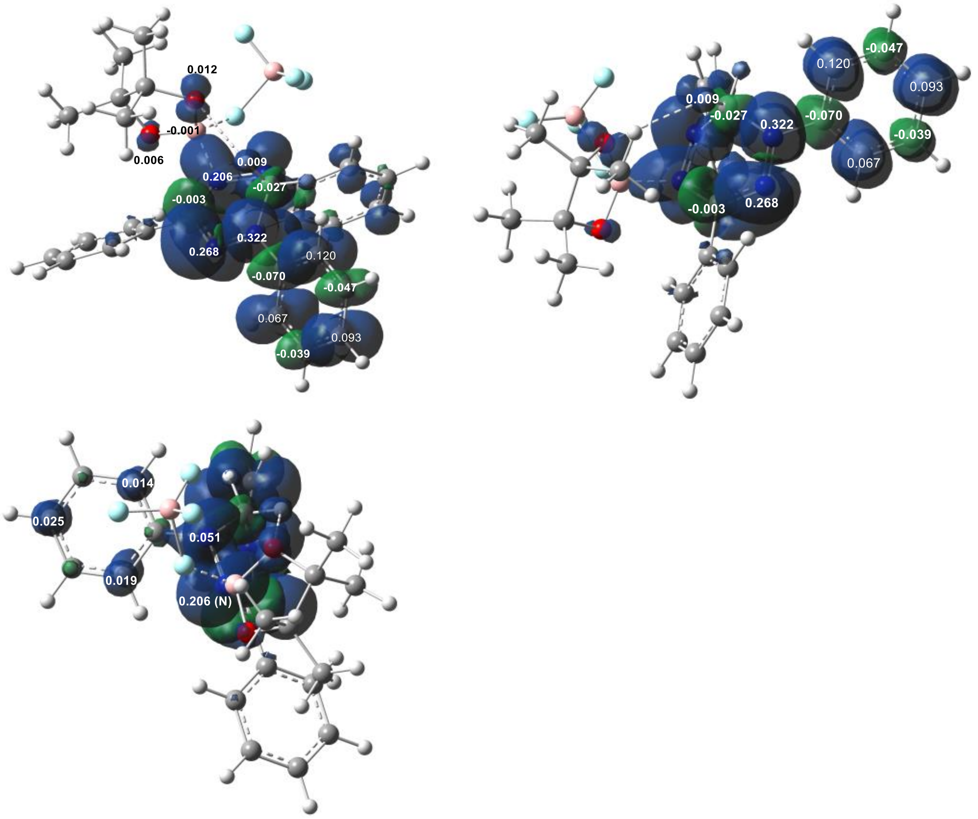

Figure S10. Mulliken spin density plot of the resultant complex after SET (from different angles).

Cartesian coordinates of the optimized structures

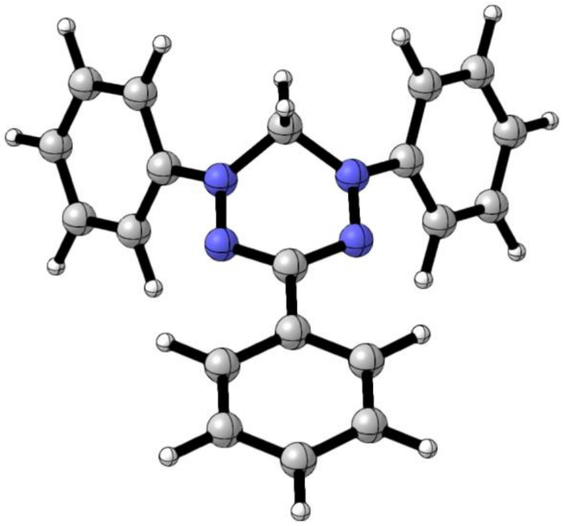

Verdazylium cation (**1a**$^+$)

Parameter	Value
Charge	1
Multiplicity	1
Imaginary Frequencies	0

Electronic Energy in gas phase	-990.903299 Hartrees
Gibbs free Energy in gas phase	-990.619113 Hartrees
Electronic Energy in solution (acetonitrile)	-991.000296 Hartrees
Gibbs Free Energy in solution (acetonitrile)	-990.716078 Hartrees

Cartesian coordinates of the optimized structure

```
N      -0.43200    1.13574    -0.65291
C      -0.82396    0.00000    -1.47531
C      -1.35764    2.11198    -0.17103
N      -0.43200   -1.13574    -0.65291
H      -1.89205    0.00000    -1.65449
H      -0.22870    0.00000    -2.39516
N       0.78811    1.15348    -0.24822
C      -1.14735    2.68123     1.08245
C      -2.42759    2.49148    -0.97956
C      -1.35765   -2.11198    -0.17103
N       0.78811   -1.15348    -0.24822
C       1.47718    0.00000    -0.31415
C      -2.03317    3.65007     1.53026
H      -0.30759    2.36276     1.68670
C      -3.30831    3.45661    -0.51196
H      -2.55791    2.07589    -1.97189
C      -1.14736   -2.68122     1.08245
C      -2.42760   -2.49148    -0.97957
C       2.93380    0.00000    -0.11816
C      -3.11313    4.03427     0.73942
H      -1.88483    4.09946     2.50391
H      -4.13904    3.76739    -1.13246
C      -2.03318   -3.65007     1.53025
H      -0.30760   -2.36276     1.68670
C      -3.30832   -3.45660    -0.51197
H      -2.55791   -2.07588    -1.97189
C       3.62485    1.21078    -0.03431
C       3.62484   -1.21079    -0.03428
H      -3.80364    4.78732     1.09804
C      -3.11315   -4.03426     0.73942
H      -1.88484   -4.09946     2.50391
H      -4.13905   -3.76738    -1.13247
C       5.00032    1.20593     0.15074
H       3.08772    2.14824    -0.11247
C       5.00031   -1.20594     0.15077
H       3.08772   -2.14825    -0.11242
H      -3.80365   -4.78732     1.09804
C       5.68884    0.00000     0.24570
H       5.53629    2.14440     0.21732
H       5.53628   -2.14441     0.21737
```

H	6.76279	-0.00001	0.38615

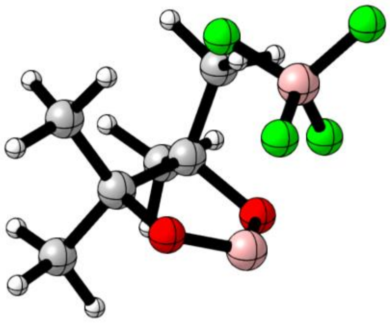

Boryl radical anion **B**

**Parameter**	**Value**
Charge	-1
Multiplicity	2
Imaginary Frequencies	0
Electronic Energy in gas phase	-835.686131 Hartrees
Gibbs free Energy in gas phase	-835.534275 Hartrees
Electronic Energy in solution (acetonitrile)	-835.778299 Hartrees
Gibbs Free Energy in solution (acetonitrile)	-835.626498 Hartrees

Cartesian coordinates of the optimized structure

C	1.69393	-0.78278	0.08002
C	1.61464	0.78421	0.13629
B	0.52322	0.00570	-1.69258
O	0.64454	1.09763	-0.89300
O	1.25385	-1.06906	-1.27579
B	-2.67825	0.00636	0.01580
F	-1.74347	-0.65167	-0.83027
F	-3.75231	-0.85555	0.26987
F	-3.12316	1.18030	-0.59197
F	-2.02515	0.31281	1.23606
C	1.11120	1.33990	1.45775
H	1.77237	1.03532	2.27597
H	1.10413	2.43114	1.40782
H	0.09452	0.99940	1.65488
C	2.91744	1.46713	-0.27770
H	3.70180	1.32210	0.47008
H	3.26758	1.08630	-1.23979

H	2.72645	2.53665	-0.38383
C	3.09464	-1.35100	0.25510
H	3.49395	-1.08193	1.23783
H	3.04752	-2.43999	0.19074
H	3.77623	-0.99041	-0.51538
C	0.72360	-1.47279	1.03177
H	0.70906	-2.53842	0.79257
H	1.04510	-1.34944	2.06995
H	-0.28659	-1.08057	0.92081

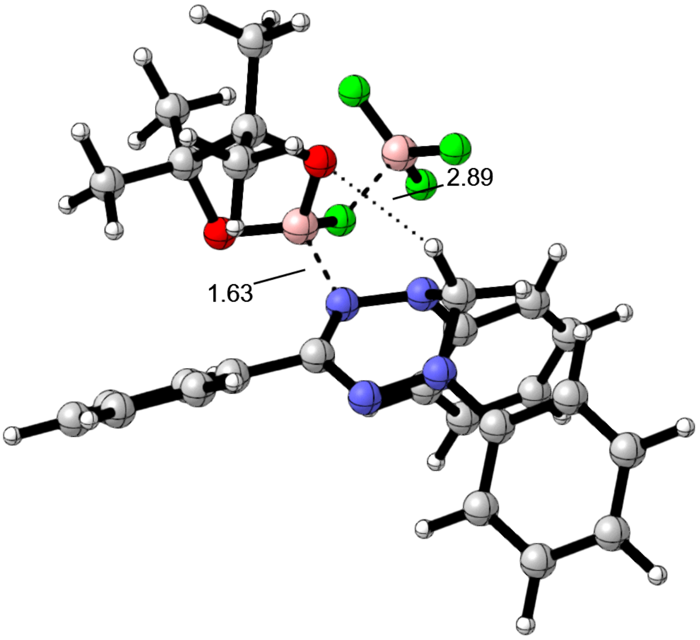

Post-SET complex (**V**)

**Parameter**	**Value**
Charge	0
Multiplicity	2
Imaginary Frequencies	0
Electronic Energy in gas phase	-1826.844498 Hartrees
Gibbs free Energy in the gas phase	-1826.380287 Hartrees
Electronic Energy in solution (acetonitrile)	-1826.902147 Hartrees
Gibbs Free Energy in solution (acetonitrile)	-1826.437936 Hartrees

Cartesian coordinates of the optimized structure

N	-0.79662	0.84112	-0.26457

C	-1.56393	0.28673	-1.33396
C	-1.40716	1.71728	0.67799
N	-2.26172	-0.89111	-0.78803
H	-2.28799	1.01253	-1.68691
H	-0.88464	-0.02747	-2.13046
C	-1.42203	1.42179	2.03889
C	-1.97652	2.90005	0.20446
C	-3.65091	-1.09456	-0.89015
N	-1.55803	-1.72340	-0.04203
C	-0.32232	-1.34150	0.31449
C	-2.01734	2.31369	2.92474
H	-0.96105	0.51211	2.40227
C	-2.58522	3.76958	1.09573
H	-1.88392	3.15545	-0.84394
C	-4.30622	-1.88618	0.05804
C	-4.36336	-0.52071	-1.94784
C	0.50981	-2.40419	0.92103
C	-2.60705	3.48151	2.45848
H	-2.01893	2.08891	3.98442
H	-3.01421	4.69393	0.72882
C	-5.67247	-2.08958	-0.05568
H	-3.73608	-2.32134	0.86709
C	-5.73159	-0.73141	-2.04271
H	-3.85811	0.06098	-2.70823
C	1.25171	-2.15399	2.07567
C	0.49873	-3.68299	0.36124
H	-3.06647	4.17402	3.15282
C	-6.39163	-1.51273	-1.09970
H	-6.18132	-2.69686	0.68281
H	-6.28106	-0.28897	-2.86433
C	1.98880	-3.17653	2.65672
H	1.26000	-1.16100	2.50578
C	1.25361	-4.69808	0.93445
H	-0.09550	-3.87052	-0.52545
H	-7.45975	-1.67217	-1.17841
C	1.99887	-4.44433	2.08262
H	2.56378	-2.97991	3.55320
H	1.25707	-5.68539	0.48908
H	2.58457	-5.23718	2.53284
N	0.11111	-0.07887	0.24623
C	3.66748	-0.49574	-0.38084
C	2.93150	-0.28398	-1.75265
B	1.62103	0.46266	-0.02128
O	1.84836	0.58691	-1.40561
O	2.60597	-0.32499	0.57190
B	1.57691	3.32008	-0.14502
F	1.50260	1.82520	0.60307
F	1.23126	4.11811	0.88867
F	0.66010	3.23178	-1.14019
F	2.86870	3.38652	-0.54524
C	3.77823	0.39634	-2.81756

H	4.67631	-0.19095	-3.02966
H	3.19963	0.48596	-3.73915
H	4.06790	1.39683	-2.49839
C	2.32929	-1.57518	-2.30849
H	3.10157	-2.26407	-2.65836
H	1.73067	-2.08792	-1.55222
H	1.68425	-1.32007	-3.15248
C	4.25530	-1.88619	-0.18833
H	4.99500	-2.10348	-0.96410
H	4.75235	-1.93609	0.78276
H	3.47718	-2.65191	-0.20995
C	4.71922	0.57535	-0.10282
H	5.05378	0.47027	0.93098
H	5.58374	0.46453	-0.76164
H	4.30108	1.57533	-0.22898

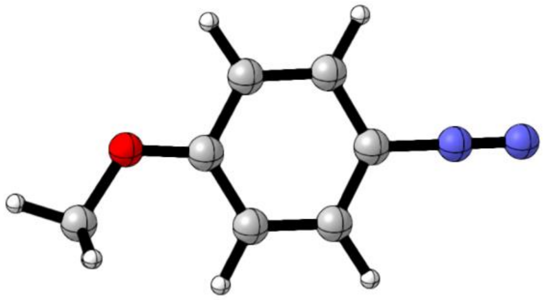

Methoxyphenyldiazonium cation (**5a**$^{+}$)

**Parameter**	**Value**
Charge	1
Multiplicity	1
Imaginary Frequencies	0
Electronic Energy in gas phase	-455.307785 Hartrees
Gibbs free Energy in gas phase	-455.208474 Hartrees
Electronic Energy in solution (acetonitrile)	-455.394856 Hartrees
Gibbs Free Energy in solution (acetonitrile)	-455.295527 Hartrees

Cartesian coordinates of the optimized structure

N	3.64352	-0.81117	0.00030
C	1.51839	0.15081	0.00000
C	0.88766	1.39011	0.00007
C	0.78247	-1.02479	-0.00033

C	-0.49285	1.45381	0.00000
H	1.49213	2.28914	0.00029
C	-0.60592	-0.96783	-0.00052
H	1.29727	-1.97834	-0.00045
C	-1.24644	0.27530	-0.00029
H	-1.01740	2.40073	0.00018
H	-1.17299	-1.88846	-0.00083
O	-2.58897	0.43708	-0.00052
C	-3.39995	-0.72149	0.00075
H	-4.42778	-0.36734	0.00156
H	-3.22339	-1.32650	-0.89346
H	-3.22166	-1.32570	0.89516
N	2.97440	0.14893	0.00020

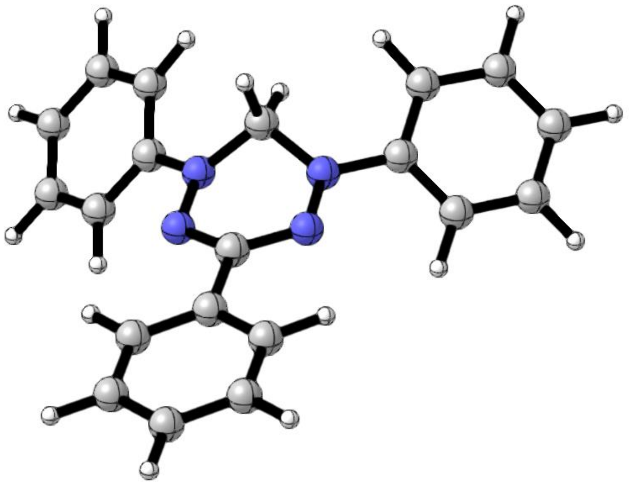

TPV radical (**1a**)

**Parameter**	**Value**
Charge	0
Multiplicity	2
Imaginary Frequencies	0
Electronic Energy in gas phase	-991.132539 Hartrees
Gibbs free Energy in gas phase	-990.852242 Hartrees
Electronic Energy in solution (acetonitrile)	-991.168972 Hartrees
Gibbs Free Energy in solution (acetonitrile)	-990.888714 Hartrees

Cartesian coordinates of the optimized structure

N	0.67880	-1.14416	-0.24416

C	1.24865	0.02786	-0.87440
C	1.41638	-2.32029	-0.03267
N	0.62534	1.17306	-0.24587
H	2.31656	0.05283	-0.68323
H	1.04429	0.02232	-1.95678
N	-0.65308	-1.20223	-0.12037
C	0.87450	-3.32959	0.77217
C	2.67586	-2.50140	-0.61279
C	1.30767	2.38170	-0.03236
N	-0.70760	1.16961	-0.12182
C	-1.28814	-0.03033	-0.15788
C	1.59558	-4.49102	0.99786
H	-0.10248	-3.18028	1.21039
C	3.38993	-3.66905	-0.36624
H	3.09802	-1.75962	-1.27824
C	0.71855	3.36328	0.77351
C	2.55768	2.62238	-0.61143
C	-2.77269	-0.06369	-0.11835
C	2.85932	-4.66878	0.43831
H	1.16844	-5.26343	1.62641
H	4.36400	-3.79739	-0.82292
C	1.38387	4.55706	1.00105
H	-0.25058	3.16771	1.21092
C	3.21574	3.82211	-0.36305
H	3.01444	1.90189	-1.27748
C	-3.45167	-1.28383	-0.09429
C	-3.50508	1.12534	-0.10891
H	3.41875	-5.57690	0.62415
C	2.63812	4.79484	0.44226
H	0.92050	5.30767	1.63032
H	4.18299	3.99671	-0.81887
C	-4.84015	-1.31176	-0.05227
H	-2.88285	-2.20472	-0.11055
C	-4.89340	1.09236	-0.06691
H	-2.97772	2.07030	-0.13666
H	3.15396	5.72817	0.62927
C	-5.56660	-0.12508	-0.03733
H	-5.35639	-2.26437	-0.03305
H	-5.45136	2.02132	-0.05946
H	-6.64944	-0.14883	-0.00643

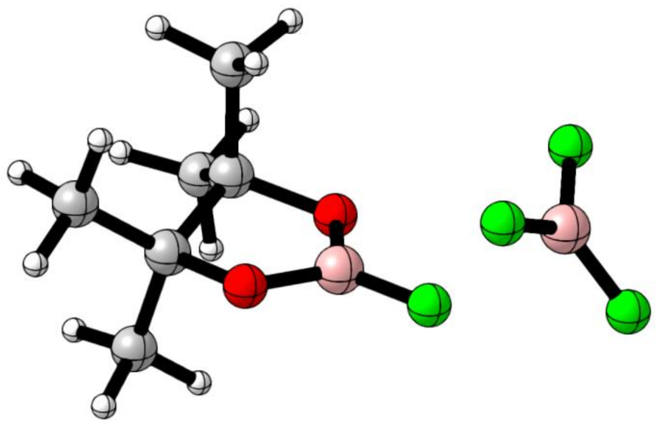

Neutral BF_3•FBPin complex

**Parameter**	**Value**
Charge	0
Multiplicity	1
Imaginary Frequencies	0
Electronic Energy in gas phase	-835.692261 Hartrees
Gibbs free Energy in gas phase	-835.537649 Hartrees
Electronic Energy in solution (acetonitrile)	-835.706259 Hartrees
Gibbs Free Energy in solution (acetonitrile)	-835.551636 Hartrees

Cartesian coordinates of the optimized structure

C	-1.98735	-0.62264	0.17917
C	-1.48872	0.84020	-0.09033
B	0.14216	-0.63188	-0.51439
O	-0.27480	0.61148	-0.85447
O	-0.74280	-1.37208	0.19470
B	3.07059	0.15983	0.20352
F	1.35330	-1.08953	-0.85534
F	3.86167	-0.87770	0.34822
F	2.18661	0.44510	1.14626
F	3.23351	0.98686	-0.80266
C	-2.43549	1.69223	-0.91438
H	-3.39430	1.79692	-0.39977
H	-2.00642	2.68669	-1.04628
H	-2.60756	1.25932	-1.89903
C	-1.07304	1.56787	1.18372
H	-1.94354	1.82998	1.78837
H	-0.39553	0.95686	1.78365
H	-0.54832	2.48350	0.90703

C	-2.69414	-0.81639	1.50763
H	-3.58335	-0.18245	1.55769
H	-3.00826	-1.85686	1.60429
H	-2.04005	-0.57557	2.34456
C	-2.82438	-1.18381	-0.96535
H	-2.96315	-2.25412	-0.80624
H	-3.80497	-0.70569	-1.00688
H	-2.32464	-1.04091	-1.92611

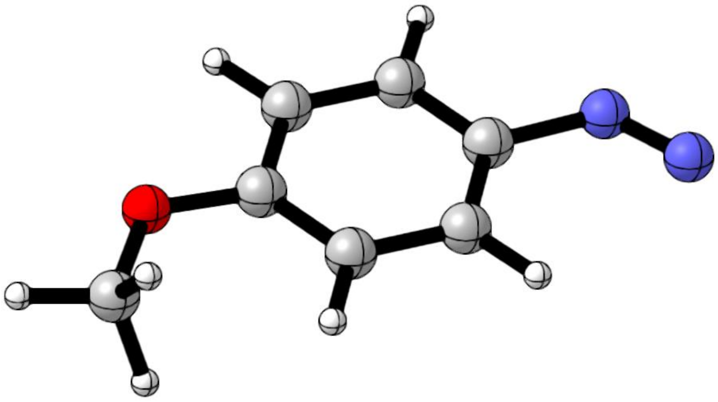

Methoxyphenyl diazinyl radical

**Parameter**	**Value**
Charge	0
Multiplicity	2
Imaginary Frequencies	0
Electronic Energy in gas phase	-455.543020 Hartrees
Gibbs free Energy in gas phase	-455.446891 Hartrees
Electronic Energy in solution (acetonitrile)	-455.553753 Hartrees
Gibbs Free Energy in solution (acetonitrile)	-455.457636 Hartrees

Cartesian coordinates of the optimized structure

N	3.64352	-0.81117	0.00030
C	1.51839	0.15081	0.00000
C	0.88766	1.39011	0.00007
C	0.78247	-1.02479	-0.00033
C	-0.49285	1.45381	0.00000
H	1.49213	2.28914	0.00029
C	-0.60592	-0.96783	-0.00052
H	1.29727	-1.97834	-0.00045

C	-1.24644	0.27530	-0.00029
H	-1.01740	2.40073	0.00018
H	-1.17299	-1.88846	-0.00083
O	-2.58897	0.43708	-0.00052
C	-3.39995	-0.72149	0.00075
H	-4.42778	-0.36734	0.00156
H	-3.22339	-1.32650	-0.89346
H	-3.22166	-1.32570	0.89516
N	2.97440	0.14893	0.00020

References

(1) Gilroy, J. B.; McKinnon, S. D. J.; Koivisto, B. D.; Hicks, R. G. E. *Org. Lett.* **2007**, *9*, 4837-4840.
(2) Firth, J. D.; Fairlamb, I. J. S. *Org. Lett.* **2020**, *22*, 7057-7059.
(3) Gosset, C.; Pellegrini, S.; Jooris, R.; Bousquet, T.; Pelinski, L. *Adv. Synth. Cat.* **2018**, *360*, 3401-3405.
(4) Qi, Z.; Wen, S.-M.; Wu, Q.; Jiang, D.-F.; Hao, W.-J.; Jiang, B. *J. Org. Chem.* **2023**, *88*, 11874-11884.
(5) Xing, B.; Ni, C.; Hu, J. *Angew. Chem. Int.l Ed.* **2018**, *57*, 9896-9900.
(6) Ding, R.; Liu, Q.; Zheng, L. *Chem. Eur. J.* **2023**, *29*, e202203792.
(7) Andrejčák, S.; Kisszékelyi, P.; Májek, M.; Šebesta, R. *Eur. J. Org. Chem.* **2023**, *26*, e202201399.
(8) Marciasini, L. D.; Vaultier, M.; Pucheault, M. *Tet. Lett.* **2014**, *55*, 1702-1705.
(9) Tian, Y.-M.; Guo, X.-N.; Kuntze-Fechner, M. W.; Krummenacher, I.; Braunschweig, H.; Radius, U.; Steffen, A.; Marder, T. B. *J. Am. Chem. Soc.* **2018**, *140*, 17612-17623.
(10) Dzhevakov, P. B.; Topchiy, M. A.; Zharkova, D. A.; Morozov, O. S.; Asachenko, A. F.; Nechaev, M. S.*Adv. Synth. Cat.* **2016**, *358*, 977-983.
(11) Lamola, J. L.; Moshapo, P. T.; Holzapfel, C. W.; Christopher Maumela, M. *Tet. Lett.* **2022**, *88*, 153572.
(12) Zhao, X.; Wu, M.; Liu, Y.; Cao, S. *Org. Lett.* **2018**, *20*, 5564-5568.
(13) Meng, C.-F.; Zhang, B.-B.; Liu, Q.; Chen, K.-Q.; Wang, Z.-X.; Chen, X.-Y. *J. Am. Chem. Soc.* **2024**, *146*, 7210-7215.
(14) Kim, H.; Kim, H.; Lambert, T. H.; Lin, S. *J. Am. Chem. Soc.* **2020**, *142*, 2087-2092.
(15) Yuen, A. K. L.; Hutton, C. A. *Tet. Lett.* **2005**, *46*, 7899-7903.
(16) Ding, Y.; Huang, R.; Zhang, W.; Huang, H. *Org. Lett.* **2022**, *24*, 7972-7977.
(17) Oka, N.; Yamada, T.; Sajiki, H.; Akai, S.; Ikawa, T. *Org. Lett.* **2022**, *24*, 3510–3514.
(18) Gaussian 16, Revision A.03, Frisch, M. J.; Trucks, G. W.; Schlegel, H. B.; Scuseria, G. E.; Robb, M. A.; Cheeseman, J. R.; Scalmani, G.; Barone, V.; Petersson, G. A.; Nakatsuji, H.; Li, X.; Caricato, M.; Marenich, A. V.; Bloino, J.; Janesko, B. G.; Gomperts, R.; Mennucci, B.; Hratchian, H. P.; Ortiz, J. V.; Izmaylov, A. F.; Sonnenberg, J. L.; WilliamsYoung, D.; Ding, F.; Lipparini, F.; Egidi, F.; Goings, J.; Peng, B.; Petrone, A.; Henderson, T.; Ranasinghe, D.; Zakrzewski, V. G.; Gao, J.; Rega, N.; Zheng, G.; Liang, W.; Hada, M.; Ehara, M.; Toyota, K.; Fukuda, R.; Hasegawa, J.; Ishida, M.; Nakajima, T.; Honda, Y.; Kitao, O.; Nakai, H.; Vreven, T.; Throssell, K.; Montgomery, J. A., Jr.; Peralta, J. E.; Ogliaro, F.; Bearpark, M. J.; Heyd, J. J.; Brothers, E. N.; Kudin, K. N.; Staroverov, V. N.; Keith, T. A.; Kobayashi, R.; Normand, J.; Raghavachari, K.; Rendell, A. P.; Burant, J. C.; Iyengar, S. S.; Tomasi, J.; Cossi, M.; Millam, J. M.; Klene, M.; Adamo, C.; Cammi, R.; Ochterski, J. W.; Martin, R. L.; Morokuma, K.; Farkas, O.; Foresman, J. B.; Fox, D. J. Gaussian, Inc., Wallingford CT, 2016.
(19) Marcus, R. A. .*J. Chem. Phys.* **1956,** *24*, 979–989
(20) Marcus, R. A. *J. Chem. Phys.* **1956**, *24*, 966–978
(21) Marcus, R. A. *J. Chem. Phys.* **1957,** *26*, 867–871.
(22) Marcus, R. A. *J. Chem. Phys.* **1957**, 26, 872–877.
(23) López-Estrada, O.; Laguna, H. G.; Barrueta-Flores, C.; Amador-Bedolla, C. *ACS Omega* **2018**, *3*, 2130–2140.
